# TBC1 domain-containing proteins are frequently involved in triple-negative breast cancers in connection with the induction of a glycolytic phenotype

**DOI:** 10.1038/s41419-024-07037-2

**Published:** 2024-09-04

**Authors:** Mariadomenica Lupi, Daniele Avanzato, Stefano Confalonieri, Flavia Martino, Rosa Pennisi, Emanuela Pupo, Valentina Audrito, Stefano Freddi, Giovanni Bertalot, Francesca Montani, Bronislava Matoskova, Sara Sigismund, Pier Paolo Di Fiore, Letizia Lanzetti

**Affiliations:** 1 Department of Oncology, University of Torino Medical School, Turin, Italy; 2 Candiolo Cancer Institute, FPO-IRCCS, Turin, Italy; 3 IEO, European Institute of Oncology IRCCS, Milan, Italy; 4 Department of Science and Technological Innovation (DISIT), University of Eastern Piedmont, Alessandria, Italy; 5 Department of Oncology and Haemato-Oncology, University of Milan, Milan, Italy; 6 Present Address: Department of Veterinary Sciences, Infectious Diseases Unit, University of Torino, Turin, Italy; 7 Present Address: Unità Operativa Multizonale di Anatomia Patologica, APSS, Trento, Italy, and Centre for Medical Sciences – CISMed, University of Trento, Trento, Italy

**Keywords:** Cancer, Breast cancer

## Abstract

Metabolic plasticity is a hallmark of cancer, and metabolic alterations represent a promising therapeutic target. Since cellular metabolism is controlled by membrane traffic at multiple levels, we investigated the involvement of TBC1 domain-containing proteins (TBC1Ds) in the regulation of cancer metabolism. These proteins are characterized by the presence of a RAB-GAP domain, the TBC1 domain, and typically function as attenuators of RABs, the master switches of membrane traffic. However, a number of TBC1Ds harbor mutations in their catalytic residues, predicting biological functions different from direct regulation of RAB activities. Herein, we report that several genes encoding for TBC1Ds are expressed at higher levels in triple-negative breast cancers (TNBC) *vs*. other subtypes of breast cancers (BC), and predict prognosis. Orthogonal transcriptomics/metabolomics analysis revealed that the expression of prognostic TBC1Ds correlates with elevated glycolytic metabolism in BC cell lines. In-depth investigations of the three top hits from the previous analyses (TBC1D31, TBC1D22B and TBC1D7) revealed that their elevated expression is causal in determining a glycolytic phenotype in TNBC cell lines. We further showed that the impact of TBC1D7 on glycolytic metabolism of BC cells is independent of its known participation in the TSC1/TSC2 complex and consequent downregulation of mTORC1 activity. Since TBC1D7 behaves as an independent prognostic biomarker in TNBC, it could be used to distinguish good prognosis patients who could be spared aggressive therapy from those with a poor prognosis who might benefit from anti-glycolytic targeted therapies. Together, our results highlight how TBC1Ds connect disease aggressiveness with metabolic alterations in TNBC. Given the high level of heterogeneity among this BC subtype, TBC1Ds could represent important tools in predicting prognosis and guiding therapy decision-making.

## Introduction

Cancers meet their high metabolic demands, both anabolic and catabolic, through a variety of adaptations, globally defined as “metabolic plasticity” [1]. The best characterized metabolic alteration in cancer is the elevation of aerobic glycolysis, the so-called Warburg effect, a process whereby pyruvate is reduced to lactate even in the presence of oxygen [2]. The Warburg effect is proposed to confer growing advantages to cancer cells mostly by increasing the production of anabolic intermediates, through heightened glycolytic flux [3, 4].

Breast cancer (BC) is the most frequently diagnosed neoplasia worldwide, accounting for ~12% of all cancer diagnoses and ~7% of cancer-related deaths [5]. It is a phenotypically and molecularly heterogenous disease, which can be categorized into molecular subtypes based on the expression of the estrogen and progesterone receptors (ER and PGR, respectively), and the amplification of the *HER2* oncogene (HUGO: *ERBB2*). Luminal BCs are ER+/HER2-; HER2+ BCs display amplification of HER2, regardless of the ER/PGR status; triple-negative BCs (TNBCs) are negative for the expression of ER, PGR and HER2 [6]. Among these subtypes, TNBCs display overall worse prognosis and, in general, scarce response to therapy [7]. TNBCs are also clinically heterogeneous, with a significant proportion characterized by poor prognosis in the first 4-5 years after diagnosis compared to Luminal BCs. However, after 5 years the mortality curve flattens becoming superimposable with that of Luminal BCs [8, 9].

The molecular heterogeneity of the different BC subtypes is associated with distinct metabolic features [10], and metabolic heterogeneity is present also within subtypes. For instance, multi-omics analysis has identified lipogenic, glycolytic and mixed phenotypes within the TNBC subtype, associated with increased sensitivity to specific metabolic inhibitors [11]. Thus, investigations into the molecular basis of the metabolic heterogeneity of TNBC hold promises for this BC subtype in which there is pressing need for novel therapies.

Cellular metabolism is controlled by membrane traffic at several levels. First, membrane trafficking controls the number of nutrient transporters on the cell surface by regulating their delivery to the plasma membrane (PM), and their endocytosis, recycling and degradation [12]. Since most of these carriers operate through facilitative diffusion, their abundance largely determines the extent of nutrient supply. Second, the major scavenging pathways, including autophagy and macropinocytosis, that allow cells to survive under nutrient-deprived conditions are membrane-based [13, 14]. Finally, the core of cell metabolism is represented by the lysosomal vesicular compartment. On lysosomes, proliferative signals, which can be in turn regulated by membrane traffic, and nutrient abundance signals converge to activate, in a cooperative manner, the mTORC1 complex which, in turn, stimulates anabolic pathways while downregulating catabolic ones [15].

Membrane trafficking is regulated by the activity of RAB GTPases (RABs), small GTP-binding proteins that mark distinct vesicular compartments [16–19]. RABs cycle between a GTP-bound active state – in which they recruit downstream effectors to coordinate vesicle budding, movement, tethering and fusion – and a GDP-bound inactive form. The switch between GTP and GDP-bound RAB is controlled by GTPase-activating proteins (RAB-GAPs). RAB-GAPs bind to their RAB target and provide two catalytic residues (RQ) to stimulate hydrolysis of the bound GTP [16, 20]. The catalytic activity of RAB-GAPs resides in an evolutionarily conserved domain named the TBC1 domain [20]. Thus, RAB-GAPs are also referred to as the TBC1 domain-containing proteins (TBC1Ds). Interestingly, some TBC1Ds harbor mutations in the critical catalytic residues and thus are likely to lack GTPase activity [20]. It is hypothesized that at least some of the catalytically inactive TBC1Ds might still be able to bind to the cognate GTP-bound RABs, thereby functioning as effectors, although they might also have diverged to assume functions not immediately traceable to membrane traffic, as in the case of TBC1D7 (see below).

There is evidence that some TBC1Ds are involved in maintaining homeostasis of metabolic pathways and can contribute to the subversion of these pathways in human diseases (see for instance [21–47]). One example is the catalytically inactive TBC1D7 protein that interacts with and regulates the TSC1/TSC2 complex, a GAP for the small GTPase RHEB. In its active GTP-bound form, RHEB binds to and activates the mTORC1 complex, a key regulator of cellular metabolism [41, 48, 49]. By stabilizing the TSC1/TSC2 complex, TBC1D7 promotes the conversion of RHEB-GTP to RHEB-GDP, thereby inhibiting mTORC1.

Based on this background, we hypothesized that subversion of TBC1Ds might lead to metabolic reprogramming of cancer cells, specifically to the activation of the Warburg effect. The present study was undertaken to test this hypothesis, with a particular focus on TNBC in which elevated glycolysis correlates with patient prognosis and resistance to therapy [11, 50, 51].

## Results

### TBC1D genes are frequently expressed at higher levels in TNBCs *vs*. other subtypes of BCs, and predict prognosis

**Fig. 1:**
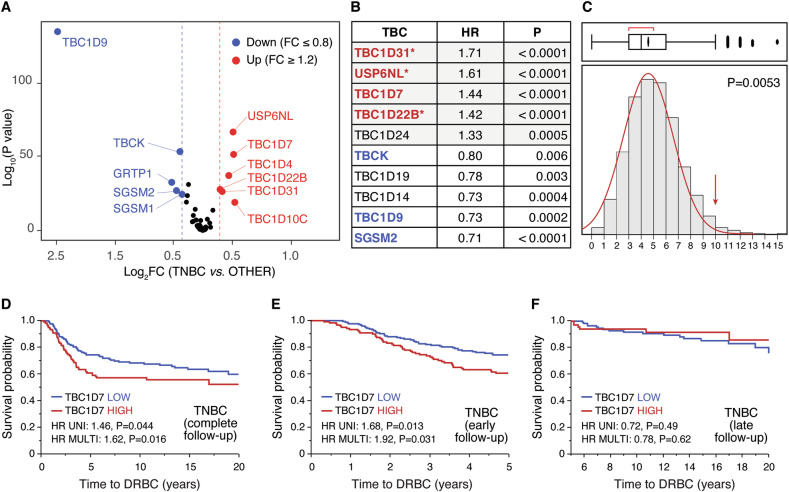
Analysis of TBC1D genes in the METABRIC dataset (N = 1904 cases). **A** For each individual TBC1D gene the level of expression in the TNBCs of the METABRIC dataset (N = 299 TNBCs) *vs*. all other molecular subtypes (N = 1605 cases) was calculated as FC (fold change in TNBCs/non-TNBCs). Results in the Volcano plot are expressed as log_2_ FC *vs*. log_10_ P values. Cut-off values for overexpression (FC ≥ 1.2, red circles) or underexpression (FC ≤ 0.8, blue circles) are indicated by dashed red and blue lines, respectively. Significance threshold was set at P < 0.05. The fold change (FC) was determined based on the mean expression of each TBCs in TNCB versus all other samples. All p-values were derived with the non-parametric Wilcoxon test using JMP version 14.3. The complete set of data is in Supplementary Table 1. **B** TBC1D genes were analyzed for HR (hazard ratio) for death related to BC (DRBC) in univariate analysis. P-values and HR were calculated by Cox proportional hazards regression model analysis using ‘survival’ package in R, version 3.5–5. Only genes displaying HR ≥ 1.2 or ≤ 0.8 at P < 0.01 are shown. Genes associated with worse prognosis are shaded in gray. In red and blue are shown the genes found overexpressed or underexpressed in TNBCs (as per panel A), respectively. Multivariable analysis (variables used for the multivariable analyses were: age, tumor size, nodal status, HR (ER/PGR), HER2, and tumor grade) was also performed, and the genes that remained significant in this type of test are indicated by asterisks. The complete set of data is in Supplementary Table 2. **C** Montecarlo simulation was performed in R to test the probability of finding ≥10 genes predicting poor prognosis in univariate analysis in random sets of 44 genes, from the 24 368 genes present in the METABRIC dataset. Ten thousand random sets were generated and tested, yielding 53 sets containing ≥10 significant genes. Thus, the probability of random occurrence is 0.0053. **D** The expression of TBC1D7 was categorized as HIGH or LOW with respect to the mean expression in the TNBC (n = 299) subtype of samples from the METABRIC dataset. Following this categorization, Kaplan–Meier analyses, univariate and multivariable survival analyses were performed within JMP, employing the Survival platform and the Cox proportional hazards model, as appropriate. **E**, **F** TNBC samples were probed for survival analysis considering early (0–5 years, **E** panel) and late (5–20 years, **F** panel) DRBC. Kaplan-Meier analyses, univariate and multivariable survival analyses were performed as described in **D**.

We conducted a survey of the levels of expression of TBC1D genes in the METABRIC dataset of BCs. Of the 54 genes encoding TBC1Ds, data could be retrieved for 44 genes. By adopting a FC (fold-change) threshold of ± 20% (≥1.2 or ≤0.8 FC), we identified 11 TBC1D genes that displayed significant levels of over- or under-expression in TNBCs *vs*. other molecular subtypes of BC (Fig. 1A and Supplementary Table 1). This was reflected in the ability of TBC1D genes to predict prognosis in BC. By adopting rather stringent cut-off values (HR, at least ±20%, i.e., ≥1.2 or ≤0.8; P < 0.01), we found that 10 TBC1D genes were associated with good or bad prognostic outcome (death related to BC, DRBC), with significant correlation with their status of over- or under-expression in TNBCs (Fig. 1B; Supplementary Fig. S1 and Supplementary Table 2). A Montecarlo simulation revealed that this value is highly significant with respect to a random occurrence (Fig. 1C). Multivariable analysis revealed that three out of the five overexpressed/bad prognosis predictor genes (*TBC1D31*, *USP6NL*, and *TBC1D22B*, indicated by an asterisk in Fig. 1B, see also Supplementary Table 2) remained significant, indicating that they are independent predictors of worse clinical outcome.

Finally, an interesting pattern emerged for *TBC1D7*. While high levels of expression of this gene predicted prognosis in univariate analysis, the correlation was lost in multivariable analysis (Supplementary Table 2). However, when the prognostic power was assessed within the molecular subtypes of BC, *TBC1D7* was prognostic in TNBCs, both in univariate and multivariable analysis (Fig. 1D and Supplementary Table 3). The effect was evident for the risk of early DRBC (0–5 years, Fig. 5E), but not for late risk (≥5 years, Fig. 1F). *TBC1D7* was not prognostic of bad outcome in any other molecular sub-groups (Supplementary Table 3).

We concluded that TBC1D genes are frequently perturbed in BC, in particular in the TNBC molecular subtype. In addition, *TBC1D7* specifically stratifies TNBCs.

### The levels of expression of TBC1D genes correlate with glycolytic metabolism in BC cell lines

The aggressive behavior of TNBCs compared with other BC subtypes has been attributed in part to their enhanced glycolytic metabolism [52]. Given the perturbed expression of TBC1D genes in TNBC, we investigated whether their levels of expression correlated with distinct metabolic phenotypes. For this purpose, we employed a panel of 46 BC cell lines [53] for which metabolomic [54] and transcriptomic [55] data were available (Supplementary Tables 4–6).

**Fig. 2:**
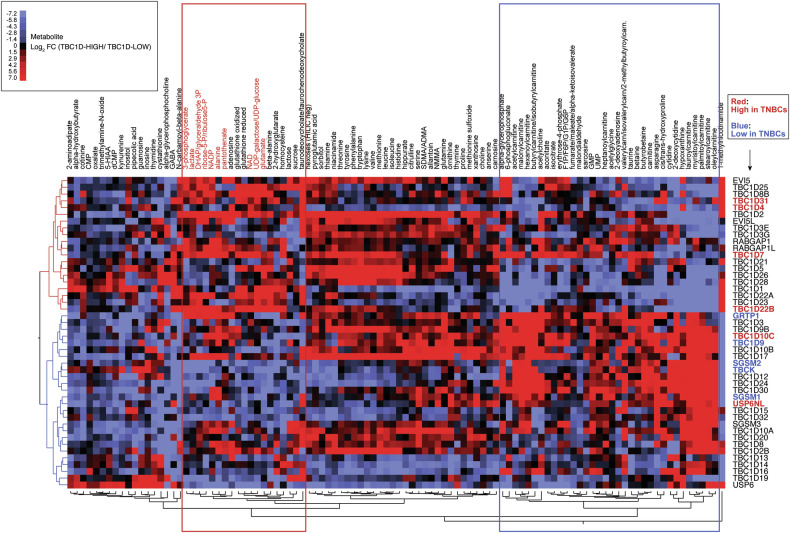
Hierarchical clustering of metabolites stratified by TBC1D gene expression. From the dataset of the 46 BC cell lines (see Supplementary Tables 4, 5, 6), we retrieved expression data for 46 TBC1D genes. For each gene, the 46 cell lines were ranked in order of levels of expression (1 through 46). Then, for each gene, the cell lines received the label TBC1D-HIGH or TBC1D-LOW, depending on whether that cell line fell in the upper or in the lower tertile of expression. In practical terms, given a cell line (e.g., HDQP1) and a TBC1D gene (e.g., EVI5) (first line of Supplementary Table 6), the EVI5 status of that cell line was EVI5-LOW. Then, we calculated the average concentration of each metabolite in the HIGH and LOW groups (for instance: average expression of 1-methylnicotinamide in EVI5-HIGH lines and EVI5-LOW lines, first line of Supplementary Table 7) and the FC between the averages (Supplementary Table 7). Finally, we performed the unsupervised clustering analysis shown in the picture, in which rows represent TBC1D genes and columns represent metabolite FCs identified with a color code indicative of log_2_ FC values (color code is in the inset). All Distance-based Dendrograms were created using the Ward’s method in cluster analysis within JMP. Only metabolites showing significant differences in at least 2 comparisons (TBC1D-HIGH *vs*. TBC1D-LOW) are shown. The complete dataset is in Supplementary Table 7. TBC1D genes associated with glycolytic and FAO metabolites are depicted in red and blue respectively in the dendrogram on the left. Red and blue boxes contain metabolites preferentially associated with glycolysis and FAO, respectively. TBC1D genes overexpressed and underexpressed in TNBC are shown in red and blue, respectively, in the list on the right.

For each TBC1D gene, the BC cell lines were categorized as TBC1D-HIGH or TBC1D-LOW, defined as the upper or lower tertiles of mRNA expression of that TBC1D gene (Supplementary Table 6, see also legend to Fig. 2). The FC in the average levels of each metabolite in TBC1D-HIGH *vs*. TBC1D-LOW cell lines was then determined for each TBC1D gene (Supplementary Table 7). By hierarchical clustering analysis, two groups of TBC1D genes could be readily identified (Fig. 2). One group (depicted in red in the dendrogram on the left of Fig. 2) correlated with high levels of metabolites enriched in products of glycolytic metabolism or connected pathways (red box in Fig. 2). The other group (depicted in blue in the dendrogram on the left of Fig. 2) showed enrichment of metabolites connected with fatty acid oxidation (FAO) (blue box in Fig. 2). Interestingly, the groups of TBC1D genes associated with glycolytic or FAO metabolism were also enriched in genes overexpressed or underexpressed in TNBCs, respectively (Fig. 2). These findings suggest a connection between the levels of expression of certain TBC1D genes, the TNBC subtype, and enhanced glycolytic metabolism.

### Specific TBC1Ds drive a glycolytic phenotype in TNBC cells

**Fig. 3:**
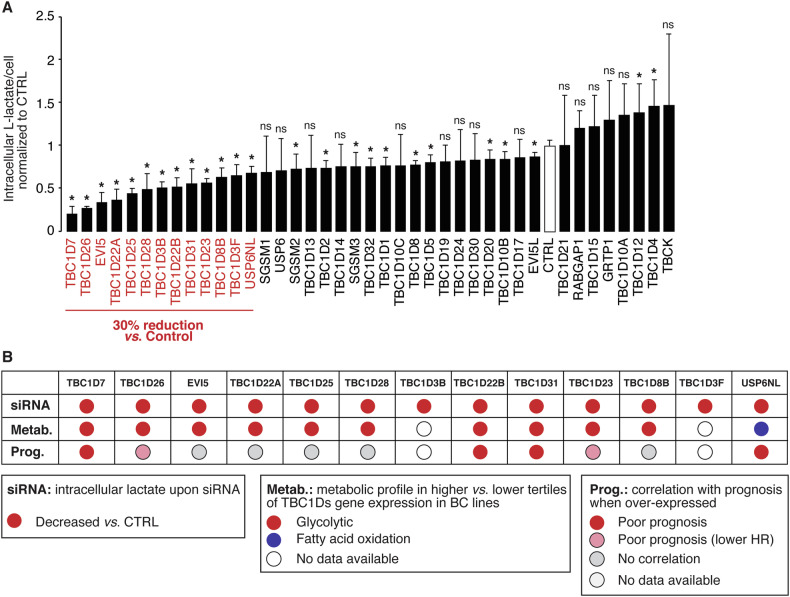
TBC1D gene expression and lactate production. **A** Levels of intracellular L-lactate upon silencing of the indicated TBC1D genes in MDA-MB-468 TNBC cells. Genes whose silencing induced a decrease in lactate production of at least 30% relative to silencing control (CTRL) are indicated in red. Data are expressed as mean ± SD (n = 4). *P < 0.05; ns, not-significant (see also Supplementary Table 8). **B** The characteristics of the “red” genes in panel A are shown, including the results of the siRNA experiment (panel A), the metabolic profiles (from Fig. 2), and the correlation with prognosis (from Fig. 1B). In the category “Prognosis”, red circles indicate genes strongly correlating with prognosis in univariate analysis (HR ≥ 1.2; P < 0.01, as from Fig. 1B), pink circles indicate genes significantly correlating with prognosis (P < 0.05) but with lower HRs (~1.2, see Supplementary Table 2).

To obtain mechanistic evidence of the link between TBC1D gene expression and enhanced glycolytic metabolism in TNBC, we performed a global siRNA-based screening of TBC1D genes. Since lactate production is the most indicative feature of glycolytic metabolism elevation, we analyzed the intracellular lactate levels following the silencing of a panel of 40 TBC1D genes in the TNBC cell line MDA-MB-468 (Fig. 3A and Supplementary Table 8). Using a threshold of at least a 30% reduction, we identified 13 TBC1D genes whose silencing reduced lactate production. Of note, there was a good correlation between the involvement of TBC1D genes in lactate production, their association with a glycolytic metabolic profile and their ability to predict poor prognostic outcome (Fig. 3B). In particular, three genes – *TBC1D7*, *TBC1D22B* and *TBC1D31* – exhibited consistent behavior across all assays (Fig. 3B).

**Fig. 4:**
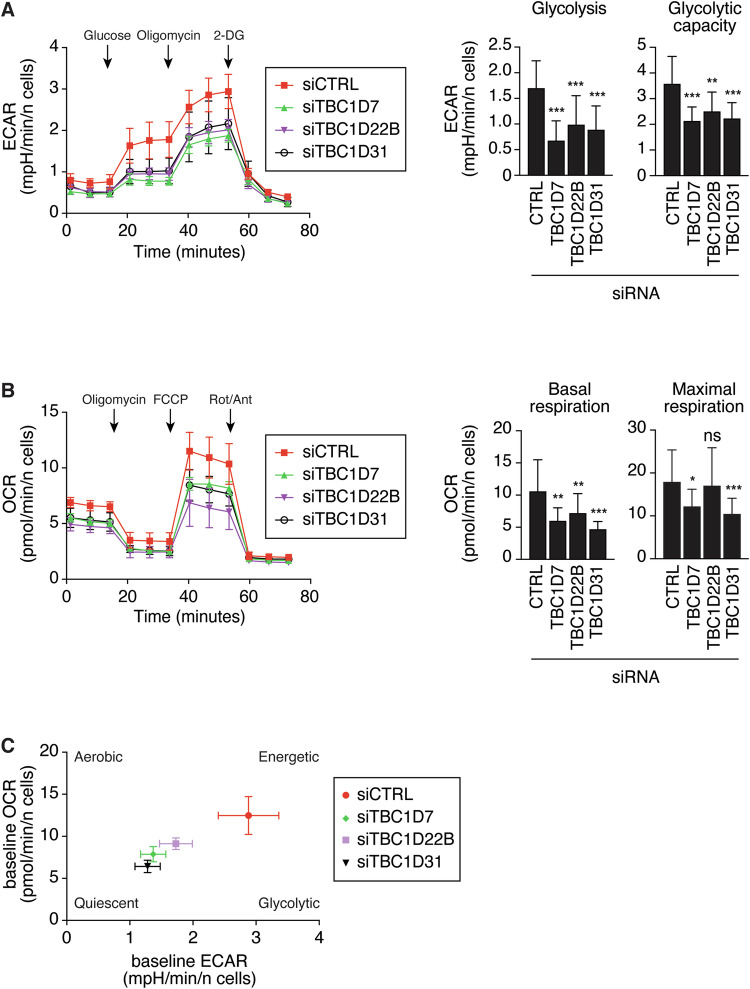
Seahorse analysis of MDA-MB-468 cells silenced with various TBC1D genes. **A** Extracellular acidification rate (ECAR) was measured in MDA-MB-468 silenced with control oligos or siRNAs for TBC1D genes ECAR data were normalized on the number of cells. 2-DG: 2-Deoxy-D-glucose. The shown profiles (left) are representative of 3 independent experiments. Values are the mean ± SD n = 6. The bar graphs (right) report the parameters glycolysis and glycolytic capacity in cells silenced as indicated (data represent mean ± SD of 3 independent experiments, n = 18). **B** O_2_ consumption rate (OCR) was measured in MDA-MB-468 silenced with control oligos or siRNAs for TBC1D genes. The shown profiles (left) are representative of 3 independent experiments. OCR data were normalized on the number of cells. FCCP: carbonyl cyanide-4-(trifluoromethoxy)phenylhydrazone, Rot/Ant: rotenone + antimycin. Values are the mean ± SD n = 6. The bar graphs (right) report the parameters basal respiration and maximal respiration in the cells silenced as on bottom (data represent mean ± SD of 3 independent experiments, n = 18). In **A**, **B**, *P < 0.05; **P < 0.01; ***P < 0.001; ns not-significant. **C** Energy map. Relative baseline ECAR and OCR data were plotted simultaneously to reveal overall relative metabolic profiles of MDA-MB-468 cells, variously silenced as indicated in the legend on the right. Data represent mean ± sem of 3 independent experiments, n = 18.

We evaluated the impact of these three genes on the metabolic status by conducting a Seahorse analysis in MDA-MB-468 cells silenced for their expression. A significant reduction in both the basal and maximal glycolytic rates was observed following silencing of the three genes, as determined by measuring the extracellular acidification rate (ECAR, Fig. 4A). Similarly, the basal and maximal mitochondrial respiration were reduced in silenced cells, as determined by measuring the oxygen consumption rate (OCR, Fig. 4B). Together, these alterations in metabolic profiles determine a shift from a high energetic condition towards a quiescent state (Fig. 4C).

**Fig. 5:**
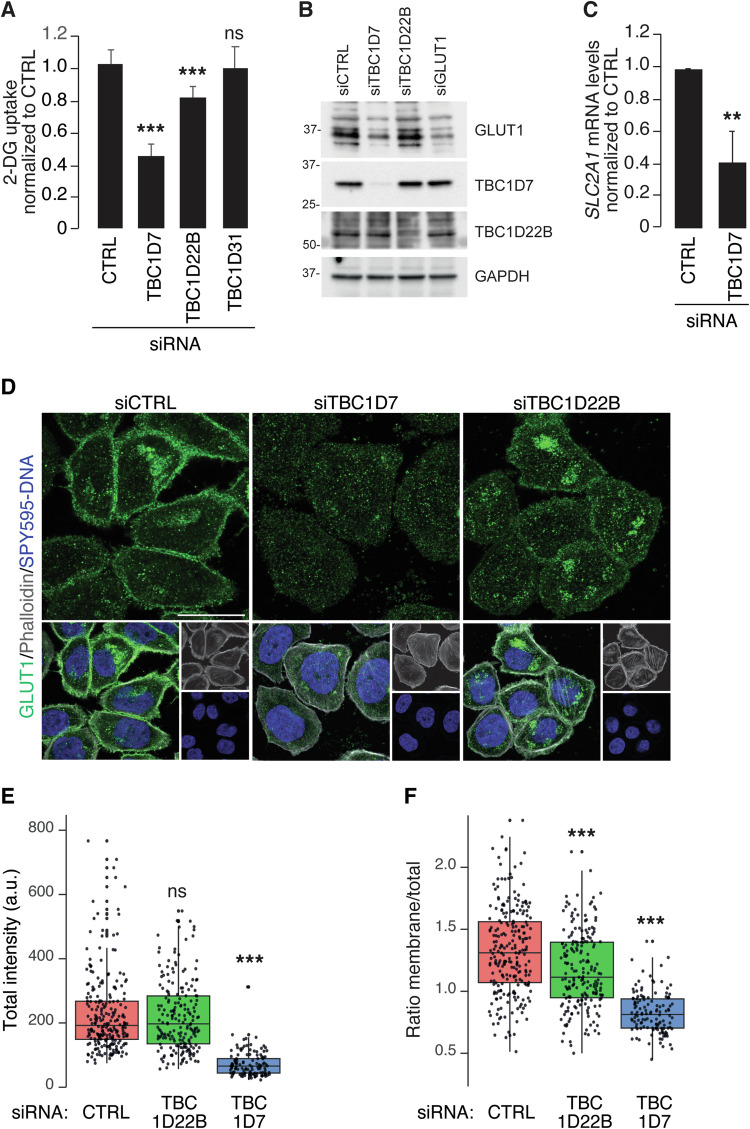
Effects of TBC1Ds depletion on glycolysis. **A** Bar graph of 2-deoxyglucose (2-DG) uptake measured in MDA-MB-468 cells silenced with control siRNA (siCTRL), TBC1D7 siRNA (siTBC1D7), TBC1D22B siRNA (siTBC1D22B), TBC1D31 siRNA (siTBC1D31). Values are the mean ± SD of 3 independent experiments, n = 18. **B** IB analysis of GLUT1 expression levels in deglycosylated lysates from MDA-MB-468 cells silenced as indicated on top; GAPDH, loading control. MW markers are shown in KDa. Because GLUT1 is highly glycosylated, we performed deglycosylation of the lysates to better compare the total amount of GLUT1 in the various samples. **C** Real-time PCR measuring the *SLC2A1* (encoding GLUT1) mRNA levels in MDA-MB-468 cells silenced as indicated on bottom. The bar graph is the mean ± SD of 3 independent experiments, n = 9. **D** Representative confocal images of cells, silenced as indicated on the top, and stained with anti-GLUT1 antibody (in green in the top row and in the merged images in the bottom row), and 647-Phalloidin for actin detection (in gray, bottom row) and SPY595-DNA to reveal the nuclei (in blue, bottom row). Images are projection of 4 Z-stacks. Bar 20 μm. **E**, **F** Quantitative analysis of GLUT1 localization in the silenced cells. The indicated number of cells, was analyzed as described in Materials and Methods. The box plot in **E** shows the total intensity/cell, expressed in arbitrary units (a.u.). The box plot in **F** shows the ratio of mean GLUT1 intensity at the plasma membrane/total. In **A**, **C**, **E**, **F**: **P < 0.01; ***P < 0.001; ns not-significant.

To further investigate the role of the three TBC1D genes in glycolysis, we measured the effects of their silencing on glucose uptake, which was reduced by depletion of *TBC1D7* and *TBC1D22B*, but not of *TBC1D31* (Fig. 5A).

This effect was, at least in part, due to alterations in the proper membrane localization of GLUT1, the major transporter of glucose across the PM (encoded by the *SLC2A1* gene). We found that the silencing of *TBC1D7*, but not of *TBC1D22B*, significantly reduced the cellular content of GLUT1 (Fig. 5B). Interestingly, this effect was due to reduced levels of *SLC2A1* mRNA (Fig. 5C). These results were confirmed by IF with anti-GLUT1, which revealed decreased overall levels of GLUT1 in *TBC1D7*-silenced cells, but not in *TBC1D22B*-silenced ones (Fig. 5D,E). By IF, we also noticed that, while in control cells a significant fraction of the GLUT1 staining was localized at the cell periphery, most likely in association with the PM, this localization was reduced both in *TBC1D7*- and *TBC1D22B*-silenced cells (Fig. 5D). Indeed, a quantitation of the membrane/total localization of GLUT1 revealed that the protein was relatively less present on the PM of *TBC1D7*- and *TBC1D22B*-silenced cells (Fig. 5F).

Altogether these findings indicate that TBC1D7, TBC1D22B and TBC1D31 are required to sustain the high energetic metabolism in MDA-MB-468 cells. Mechanistically, TBC1D7 and TBC1D22B participate in the early steps of glycolysis by regulating GLUT1 availability at the cell surface.

### TBC1D7 is needed to maintain metabolic functions and a glycolytic phenotype in TNBC cell lines

In addition to being the strongest regulator of lactate production (Fig. 3A), *TBC1D7* was overexpressed in TNBC at the mRNA level and predicted poor prognostic outcome (Fig. 1). TBC1D7 has been shown to regulate metabolism through negative regulation of the mTORC1 complex [41]. Thus, we investigated further the involvement of TBC1D7 in the regulation of glycolytic metabolism.

**Fig. 6:**
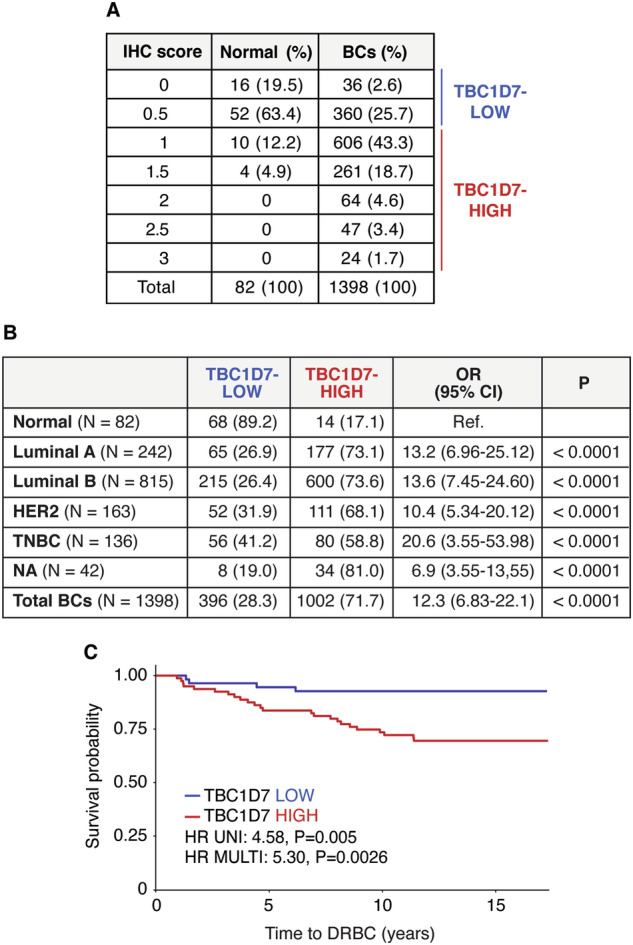
TBC1D7 is overexpressed at the protein level in BCs compared to normal breast tissues and predicts prognosis in TNBCs. **A** FFPE samples of normal breast tissue (n = 82) and BC cases from the IEO consecutive cohort (n = 1398) were evaluated for TBC1D7 expression levels. The number of cases displaying each IHC score is shown (percentage in parentheses). Samples were divided into two subgroups: TBC1D7-LOW (IHC score < 1) and TBC1D7-HIGH (IHC score ≥ 1). **B** The number and percentage of TBC1D7-LOW and TBC1D7-HIGH normal and BC samples are reported in the table. The two-class model was used to calculate the odds ratio (OR) with 95% confidence intervals (CI). The p-value was calculated using Pearson’s Chi-Squared Test. **C** Kaplan–Meier analysis of time to DRBC in the TNBC subgroup of the IEO cohort, stratified using the two-class model of TBC1D7 protein expression. Univariate (UNI) and multivariable (MULTI) hazard ratios (HRs) were calculated using the Cox proportional hazards regression model in the ‘survival’ package of R, version 3.5–5. The multivariable analysis was adjusted for age, tumor size, tumor grade, nodal status, and Ki-67.

Initially, we validated the correlation between prognosis and TBC1D7 expression at the protein level using a large consecutive BC cohort, collected at the European Institute of Oncology (the IEO cohort) [56–58]. TBC1D7 levels in FFPE samples were analyzed by immunohistochemistry (IHC) on tissue microarrays (TMA) (Supplementary Fig. S2). Using a two-class score model (TBC1D7-LOW, IHC score < 1; TBC1D7-HIGH, IHC score ≥ 1), TBC1D7 was clearly overexpressed in BC samples, of all molecular subtypes, compared to normal breast tissues (Fig. 6A, B). In agreement with the transcriptomic data, when the two-class model was used to predict prognosis, it readily distinguished TNBCs with good prognosis from those with poor prognosis (Fig. 6C) while it had no prognostic power in the other molecular subgroups (Supplementary Table 9). Thus, TBC1D7 expression stratifies TNBCs based on their prognostic outcome in two independent cohorts (METABRIC and IEO), assessed using different technological platforms at the mRNA and protein levels, respectively.

**Fig. 7:**
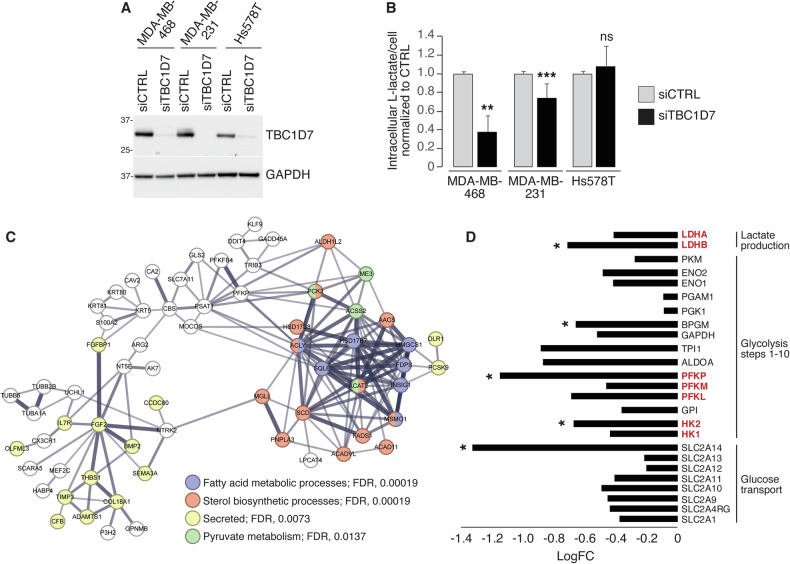
Expression of TBC1D7 is needed to maintain a glycolytic phenotype in TBC1D7-HIGH TNBC lines. **A** IB analysis of TBC1D7 expression levels in the indicated TNBC cell lines, in the presence of control siRNA (siCTRL) or TBC1D7 siRNA (siTBC1D7). GAPDH, loading control. MW markers are shown in KDa. **B** Intracellular L-lactate levels were measured in the indicated TNBC cell lines, silenced for TBC1D7 (siTBC1D7) or transfected with control siRNA CTRL. Data are expressed as mean ± SD of l-lactate per cell, normalized to the respective siCTRL in each cell line (n = 6 technical replicates from 3 independent experiments except for MDA-MB-231 cells where n = 8 technical replicates from 4 independent experiments). **C** STRING network analysis of genes classified as downregulated following TBC1D7 silencing in MDA-MB-468 cells, using a cut-off of FC ± 2.0, FDR < 0.05. The search was performed at a high-confidence setting (0.7). In the network, the thickness of the edges indicates the strength of supporting data. Non-connected nodes were not included. The “analysis” tool of STRING was used to select GO, KEGG, and Wiki terms/pathways. Selected terms are highlighted indicated by a color code. **D** Expression analysis of glycolytic genes in TBC1D7-silenced MDA-MB-468 cells. Values are plotted as the FC in TBC1D7-KD *vs*. CTRL siRNA cells (details are in Supplementary Table 10). In red, genes encoding rate-limiting enzymes. Note that in the entire RNAseq dataset, 17,329 genes were present of which 8054 were positively regulated and 9 275 negatively regulated following TBC1D7 KD (regardless of significance). Thus, for a single random gene the probability of being downregulated in the dataset was 0.535, and the probability of 24 random genes being simultaneously downregulated was 0.535^24^, i.e., 3.02 × 10^−7^. In B: **p < 0.01; ***p < 0.001; ns, not-significant. In D, *, significantly regulated at FC < 1.5, FDR < 0.05.

**Fig. 8:**
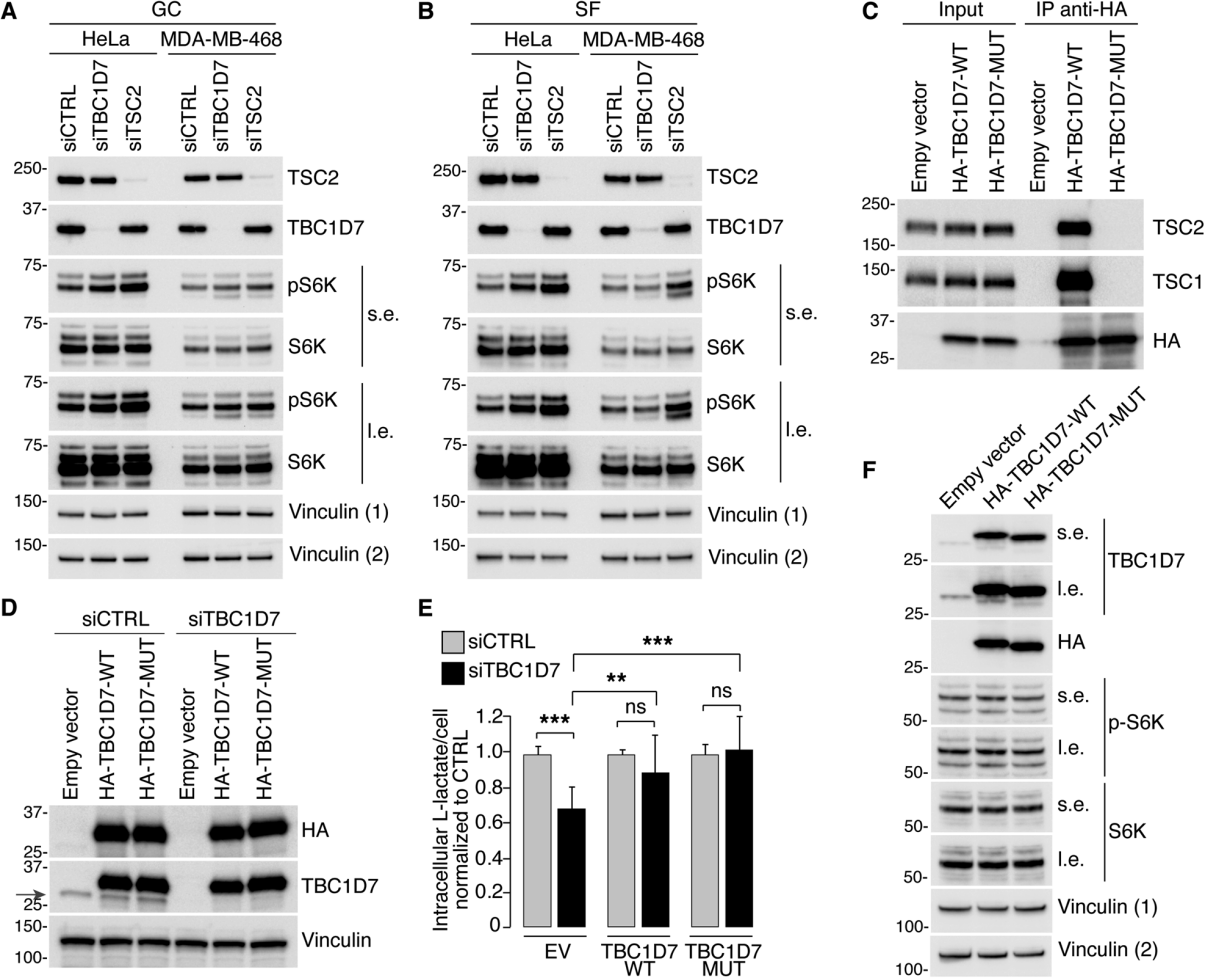
The interaction with the TSC1/TSC2 complex is not required for the effects of TBC1D7 on glycolysis in BC cells. **A**, **B**. HeLa and MDA-MB-468 cells were transfected with 10 nM of siCTRL, siTBC1D7 or siTSC2 (as shown on top). Silenced cells were left in complete medium, growing conditions (GC) (**A**), or serum starved for 16 h (SF) (**B**) before harvesting. Total cellular lysates (15 μg) were IB with the antibodies indicated on the right (s.e., short exposure; l.e., long exposure, in these and in the other panels). Vinculin (1), loading control for pS6K and TBC1D7; Vinculin (2), loading control for TSC2 and S6K. MW markers are shown in KDa in these and subsequent panels. **C** Total cellular lysates (1 mg) from MDA-MB-468 cells, stably expressing empty vector, HA-tagged TBC1D7 wild type (TBC1D7-WT) or TBC1D7-MUT (a TBC1D7 mutant in which Arg81, Gln84 and Arg121 were mutagenized to Ala) were immunoprecipitated with the anti-HA antibody and blotted as indicated on the right. Input, 15 μg of total lysates. **D** Stable MDA-MB-468 cells expressing the empty vector, HA-tagged TBC1D7-WT or TBC1D7-MUT were silenced as in **A**, and IB with the antibodies indicated on the right. The red arrow points to endogenous TBC1D7 which is effectively silenced. **E** MDA-MB-468 cells, treated as in **D** were assayed for intracellular content of L-lactate. Data are expressed as mean ± SD of l-lactate per cell, normalized to the respective siCTRL in each transfectant, and represent 15 technical replicates from 2 independent experiments. **P < 0.01; ***P < 0.001; ns, not-significant. **F** MDA-MB-468 cells stably expressing empty vector, HA-tagged TBC1D7-WT or TBC1D7-MUT were analyzed with the indicated antibodies. Vinculin (1), loading control for pS6K and TBC1D7; Vinculin (2), loading control for S6K and HA.

To dissect the molecular mechanisms underlying the role of TBC1D7 overexpression in TNBCs, we employed TNBC cell lines showing different levels of TBC1D7 protein: MDA-MB-468 (used also in the screening in Fig. 3A) and MDA-MB-231, displayed higher levels of TBC1D7, when compared to Hs578T (Fig. 7A). Following *TBC1D7* silencing, intracellular L-lactate levels were significantly decreased in the TBC1D7-HIGH cell lines (MDA-MB-468 and MDA-MB-231), while they were unaffected in TBC1D7-LOW Hs578T cells (Fig. 7B, for a rescue experiment, see Fig. 8E). Thus, in TNBC cell lines, high levels of TBC1D7 expression are needed to maintain active glycolysis.

This conclusion was strengthened by the analysis of global gene expression changes in MDA-MB-468 cells silenced for *TBC1D7*. In these experiments, using a cut-off of FC ± 2.0 (log_2_FC ± 1.0; FDR < 0.05), we identified 122 upregulated genes and 158 downregulated genes in *TBC1D7* knockdown (KD) conditions (Supplementary Fig. S3A). Pathway analysis of the upregulated genes did not reveal any significant major enrichment. Conversely, the group of the downregulated genes was significantly enriched in gene networks related to metabolism, particularly sterol biosynthetic processes, fatty acid metabolic processes, and pyruvate metabolism (Fig. 7C). In addition, a heterogenous group of genes encoding secreted proteins was downregulated (Fig. 7C). Using a less stringent cut-off of FC ± 1.5 (FDR < 0.05), results were even more striking: 250 upregulated and 568 downregulated genes were detected (Supplementary Fig. S3B). Again, no significant major enrichments of gene networks were observed among the upregulated genes. However, the downregulated genes created an extensive network of genes involved in the control of cellular metabolic processes and a smaller network of extracellular (secreted) genes (Supplementary Fig. S3C). These data strongly suggest that TBC1D7 is needed to maintain metabolic functions in the cell.

Since *TBC1D7* KD has a major impact on glycolysis and lactate production, we investigated its effects on the expression levels of genes encoding glycolytic pathway enzymes. From RNAseq data, we extracted the expression data of glucose transporters, key glycolytic enzymes, and lactate dehydrogenase (LDH) isoforms (Supplementary Table 10). Of the 24 genes analyzed, five were found to be significantly downregulated in *TBC1D7*-KD MDA-MB-468 cells compared to control cells (FC < 1.5, FDR < 0.05): *SLC2A14*, *HK2*, *PFKP*, *BPGM* and *LDHB* (Fig. 7D). Among these, HK2, PFKP, and LDHB control critical rate-limiting steps in the glycolytic pathway. Notably, all 24 genes were found to be downregulated in *TBC1D7*-KD MDA-MB-468 cells, despite significance not being reached for several of them.

### TBC1D7 induces a glycolytic phenotype independently of its regulation of the TSC1/TSC2 complex

We investigated whether the decreased glycolytic phenotype induced by silencing *TBC1D7* resulted from its known function of regulating mTORC1 activity [41]. In principle, *TBC1D7* KD should enhance the activity of the mTORC1 complex, leading to increased phosphorylation of specific substrates of this kinase complex [41, 49]. In HeLa cells, in which the TBC1D7/TSC1/TSC2 complex has been previously extensively characterized [41], *TBC1D7* KD resulted in an increase in pSK6 levels, (*TSC2* KD cells were used as positive controls) (Fig. 8A). This effect was more noticeable in serum-free conditions compared with growth conditions (Fig. 8B), consistent with previous studies [41]. In contrast, in TNBC (MDA-MB-468) cells, although *TSC2* KD increased pSK6 levels, TBC1D7 KD did not have appreciable effects in either growth or serum-free conditions (Fig. 8A, B). Moreover, in HeLa cells the production of lactate, the uptake of glucose, and the levels of GLUT1 were not reduced upon *TBC1D7* silencing (Supplementary Fig. S4). These results suggest that in the MDA-MB-468 model cell line, the function of TBC1D7 might proceed through mechanisms other than the modulation of TSC1/TSC2 and mTORC1 activity.

To investigate this possibility, we engineered a TBC1D7 mutant (TBC1D7-MUT), which has previously been described and shown to be devoid of TSC1-binding ability [59, 60]. Co-immunoprecipitation experiments confirmed that TBC1D7-MUT did not bind to the TSC1/TSC2 complex in vivo (Fig. 8C). However, this mutant was able to rescue the effects of *TBC1D7* silencing on lactate production as efficiently as WT TBC1D7 (Fig. 8D, E). Similar to TBC1D7 depletion, also overexpression of either TBC1D7 wild type or its mutant did not affect mTORC1 signaling in MDA-MB-468 cells (Fig. 8F). These results suggest that certain metabolic functions of TBC1D7, particularly its role in sustaining glycolysis, may be mediated by mechanisms independent of its function in the TSC1/TSC2 complex.

## Discussion

Herein, we report the frequent involvement of the TBC1-domain family of proteins in BC. This family plays a pivotal role in the regulation of endocytic/trafficking circuitries through its ability to modulate the function of RAB GTPases. We found that several TBC1-domain family members were highly expressed in aggressive BCs, particularly in the TNBC subtype. Their expression correlated with an aggressive disease course, and mechanistically they were able to sustain a glycolytic phenotype in BC cell lines. These results suggest that subversion of endocytic/trafficking routes is involved in driving the metabolic alterations observed in BC cells which contribute to malignant conversion.

Notably, TBC1D genes that were overexpressed in TNBCs, compared to other molecular subtypes, correlated with worse prognosis and also stratified BC lines based on their high glycolytic phenotype. In contrast, TBC1D genes that were underexpressed in TNBCs and associated with a better prognosis, appeared to be involved in sustaining FAO metabolism. These results are consistent with the findings of Gong *et al*., who showed that TNBCs can be classified as lipogenic, glycolytic and mixed phenotypes, each associated with distinct sensitivities to metabolic inhibitors [11]. Our results support the idea that these phenotypes, identified through orthogonal multiomics approaches, might result from alterations in the endocytic/trafficking machinery.

TBC1Ds endowed with RAB-GAP activity might impact directly on metabolic control through various mechanisms (reviewed in the Introduction). In the case of the three TBC1Ds herein characterized (TBC1D22B, TBC1D31 and TBC1D7), the mechanisms through which they sustain enhanced glycolytic metabolism in BC, and in TNBC in particular, remain to be elucidated.

In the case of TBC1D22B, which is catalytically competent, our findings point to its involvement in the proper localization of the glucose transporter to the PM, since silencing of *TBC1D22B* caused decreased PM levels of GLUT1. This might be the result of reduced trafficking to the PM, or of increased removal from it. Thus, TBC1D22B might sustain glycolytic flux, through maintenance of appropriate glucose transport across the PM, at least in part.

Also in the case of the catalytically-incompetent TBC1D7, there was reduced membrane/total expression of GLUT1 upon its silencing. In this case, the effect was accompanied with a substantial reduction in the total levels of GLUT1, which were due to reduced mRNA levels. In addition, silencing of *TBC1D7* affected the levels of mRNA expression of a number of key glycolytic enzymes (Fig. 7D).

Therefore, the effect of TBC1D7 on glycolytic flux, seems to be pleiotropic. On the one hand, some trafficking component must be involved (transport of GLUT1 to the PM or its endocytosis/removal, as in the case of TBC1D22B). On the other, TBC1D7 seems to regulate (directly or indirectly) transcriptional events (or mRNA stability) of the *SLC2A1* gene (encoding GLUT1) and of several other glycolytic genes.

How TBC1D7 might control transcriptional events remains unresolved, especially since the most obvious explanation, i.e., regulation of the mTORC1 pathway, was ruled out. A speculative scenario can be offered, which might also shed some light on the mechanisms involved in the regulation of glycolysis by TBC1D31. Both TBC1D31 and TBC1D7 have been implicated in ciliogenesis [61, 62]. The primary cilium is a PM organelle that is capable of receiving and interpreting signals from the extracellular environment [63]. Several signaling pathways are activated through the primary cilium and play crucial roles in modulating cell proliferation, differentiation, polarization, metabolism, and immune responses [64]. Experimental evidence suggests that ciliogenesis and glycolysis are co-regulated, and the activity of primary cilia seems to be necessary to sustain glycolysis, probably through their signaling activities [65]. This would be consistent with our findings that TBC1D31 and TBC1D7 are needed to sustain glycolytic metabolism. Indeed, an investigation into the publicly available interactomes of TBC1D31 and TBC1D7 supports mechanistically their involvement in cilium assembly (Supplementary Fig. S5). If so, the metabolic reprogramming supported by TBC1D7 and TBC1D31 might ultimately rely on regulation of critical glycolytic effectors, originating from ciliar-activated signal transduction. This regulation might in part be transcriptional, as supported by our transcriptomic analysis of TBC1D7-KD cells. In this contention, it is of note that TBC1D7 and TBC1D31 interactomes are enriched in regulators of the NFkB pathway (Supplementary Fig. S5).

In conclusion, we showed that overexpression of a number of TBC1D proteins occurs in TNBCs and correlates with the induction of a glycolytic phenotype. Clinically, TBC1D7 harbor the greatest promises. TNBCs are clinically heterogeneous and, while characterized by overall worse prognosis *vs*. the other molecular subtypes, clearly include a subgroup of patients with good prognosis that could be spared aggressive chemotherapy [8, 9]. TBC1D7 sharply stratifies the two groups, projecting its utility in patient management. In addition, high-TBC1D7 TNBCs might constitute a suitable target population for the use of anti-glycolytic therapies [66].

## Materials and methods

### Cell culture, antibodies and reagents

MDA-MB-468 and Hs578T were grown in RPMI 1640 (ECB9006L, Euroclone, Pero, Italy), MDA-MB-231 and HeLa were grown in DMEM (ECM0749L, Euroclone). All cell lines were supplemented with 10% fetal bovine serum (ECS1800D, Euroclone), 1% L-glutamine (ECB3000D, Euroclone) and 1% penicillin-streptomycin (ECB3001D, Euroclone). Cell lines were from ATCC, and were authenticated by STR profiling (StemElite ID System, Promega, Madison WI, USA) and periodically tested for mycoplasma with Venor GM Kit (56-1010, Minerva Biolabs, Berlin, Germany).

Antibodies were:

(i) from Cell Signaling Technology (Danvers, MA, USA): anti-HA-tag (C29F4) (#3724, 1:1000 for IB); anti-TBC1D7 (D8K1Y) (#14949, 1:1000 for IB); anti-TSC1/Hamartin (D43E2) (#6935, 1:1000 for IB); anti-TSC2/Tuberin (D93F12) (#4308, 1:1000 for IB); anti-p70 S6 Kinase (#9202, 1:1000 for IB); anti-phospho-p70 S6 Kinase (Thr389) (D5U1O) (#97596, 1:1000 for IB);
(ii) from Santa Cruz (Dallas, TX, USA): anti-GAPDH (6C5) (sc-32233 from 1:1000 for IB);
(iii) from Sigma (St. Louis, MO, USA): anti-Vinculin (#V9131, 1:10 000 for IB); anti-TBC1D22B (HPA027908, 1:500 for IB);
(iv) from Abcam (Cambridge, UK): anti-GLUT1/SLC2A1 (ab15309, 1:120 for IF and 1:500 for IB).
  Alexa Fluor 647 phalloidin (#A22287, 1:2000 for IF) was from Thermo Fisher Scientific (Waltham, MA, USA). SPY595-DNA (#CY-SC301 1:1000 for IF) was from Cytoskeleton (Denver, CO, USA).

### Engineering of vectors, siRNA experiments and RNAseq experiments

Silencing-resistant cDNAs for TBC1D7 and its mutant unable to bind to TSC1 (TBC1D7-MUT) were synthetized by Vector Builder (Chicago, IL, USA), tagged with HA, cloned in the lentiviral pLV vector and sequence verified (sequences are available upon request). To generate TBC1D7-MUT the following residues: Arginine 81, Glutamine 84 and Arginine 121 were substituted with Alanine. MDA-MB-468 cell populations stably expressing the empty vector (EV), HA-TBC1D7 or HA-TBC1D7-MUT were generated by lentiviral infection and selected with puromycin (P8833, Sigma).

Silencing was performed by transiently transfecting cells with 20 nM of pools of 4 ON-TARGETplus siRNA oligos, from Dharmacon (Horizon, Cambridge, UK), using Lipofectamine RNAiMAX (13778075, Invitrogen, Thermo Fisher Scientific) according to the manufacturer’s instructions. For USP6NL a previously validated siRNA oligo was employed [47]. A second round of transfection was performed 24 h after the first transfection, and cells were processed for the appropriate assay 72 h after the second transfection. Gene ID and catalog number of siRNA oligos used in the study are in Supplementary Table 11. In the assays measuring L-lactate, silencing was performed by plating cells in 24-well plates (MDA-MB-468 2.5x10^4^ cells/well, MDA-MB-231 1.5 × 10^4^ cells/well, Hs578T 1.2x10^4^ cells/well). For protein evaluation by IB or for mRNA quantitation by Real-Time PCR, cells were plated in 6-well plates (MDA-MB-468 1.5x10^5^ cells/well, HeLa 1.0 × 10^5^ cells/well). For the IB of Fig. 8B, cells were starved in medium without serum for 16 h.

For RNAseq, total cellular RNA was extracted from MDA-MB-468 silenced cells using the Maxwell RSC miRNA Tissue kit (AS1460, Promega) with the Maxwell RSC Instrument (AS4500, Promega) and the quality was assessed using the BioAnalyzer 2100 (Agilent, Santa Clara, CA, USA). Total RNA was depleted of ribosomal RNA and the RNAseq libraries were prepared with the TruSeq Stranded Total RNA kit from Illumina (San Diego, CA, USA). Following adapter ligation, libraries were amplified by PCR, checked on a Bioanalyzer 2100 (Agilent), quantified with picogreen reagent (P11495, Invitrogen, Thermo Fisher Scientific), and sequenced for 50 bases in the paired-end mode with 35 million reads coverage on a Novaseq 6000 sequencer (Illumina). Raw data were acquired for all datasets, and the human reference genome (hg38) was employed as the alignment template for mapping the reads through Bowtie2 (version 2.4.5) [67]. The estimation of gene expression abundance was carried out using RSEM (version 1.3.3) with default parameters [68].

### Lactate and glucose measurements

Intracellular lactate was extracted from the silenced cells and evaluated using the Lactate-Glo Assay (J5022, Promega), according to the manufacturer’s instruction. Briefly, culture medium was removed, and cells were washed with PBS. Inactivation solution (0.6 N HCl) and neutralization buffer (1 M Tris Base) were sequentially added to the cells before adding the detection reagent. Samples were incubated for 1 h at RT. The luminescent signal was recorded by GloMax Discover (Promega). The intracellular lactate concentration was obtained from a calibration curve, after background subtraction, and normalized over the number of cells in each well. For the normalization, after lactate extraction, cells were fixed with paraformaldehyde 4% for 10 min at room temperature and nuclei were counterstained with DAPI. Images were acquired using the LIPSI automated workstation (Nikon, Minato, Tokyo, Japan) and the number of cells were counted using the ImageJ software.

Glucose uptake was measured with the Glucose Uptake-Glo Assay (Promega) following the manufacturer’s instruction. MDA-MB-468 cells were plated and silenced in 24 well plates (3.0 × 10^4^ cells/well) as described above. Seventy-two h after the second silencing transfection, cells were incubated in glucose free medium for 4 h then incubated for 10 minutes in glucose free medium supplemented with 1 mmol/L 2-Deoxy-D-Glucose (2DG). Rate of glucose uptake (fmol/cell/min) was calculated as follows: ([2DG6P]x(volume of sample))/((number of cells)x(time of uptake)).

### Seahorse measurements

Real-time measurements of oxygen consumption rate (OCR) and extracellular acidification rate (ECAR) on MDA-MB-468 cells silenced with siRNA oligos against TBC1D7, TBC1D22B, TBC1D31 or control (CTRL), were performed with the XFe96 Extracellular Flux Analyzer (Agilent). MDA-MB-468 cells were silenced in 6-well plates (2.0 × 10^5^ cells/well) as described above and, 24 h after the second silencing transfection, they were seeded in the XF Cell Culture Microplate (1.2 × 10^4^ cells/well). Forty-two h later, cells were incubated for OCR detection in Seahorse XF DMEM (Agilent, 103575-100) containing 25 mM glucose (Agilent, 103577-100), 2 mM pyruvate (Agilent, 103578-100) and 2 mM glutamine (Agilent, 103579-100). OCR was measured using the Mito Stress Test Kit (Agilent, 103015-100) following the manufacturer’s protocol in basal conditions and in response to oligomycin (1 μM), carbonylcyanide-4-(trifluoromethoxy)-phenylhydrazone (FCCP, 1 μM) and Rotenone/Antimycin A (0.5 μM). ECAR detection was carried out in Seahorse XF DMEM containing only 2 mM pyruvate and 1 mM glutamine. ECAR was measured using the Glycolysis Stress Test kit (Agilent, 103020-100) following the manufacturer’s protocol in basal conditions and in response to glucose (10 mM), oligomycin (1 μM) and 2-Deoxy-D-glucose (2-DG, 50 mM). All reagents were from Agilent. Measurements were recorded using the WAVE software (version 2.6.1, Agilent), normalized on the number of cells in each well, and analyzed through the Mito Stress Test Report Generator, Glycolysis Stress Test Report Generator and Cell Energy phenotype Test Report Generator (Agilent) to obtain the metabolic parameters.

### Immunofluorescence

MDA-MB-468 cells were plated on coverslips coated with 0.5% gelatin in six well plates (18x10^4^ cells/well) and silenced as indicated above. Cells were fixed in 4% paraformaldehyde for 10 min and permeabilized with 0,1% Triton X-100 in PBS for 10 min. A blocking step of 30 min with 5% BSA + 0,1% Triton X-100 was performed prior to incubation with primary and secondary antibodies diluted in 5% BSA for 1 h at RT. Confocal Z-stacks (8 sections, 0.1 μm thickness) were acquired with a Leica SP8 AOBS microscope (Leica Microsystems, Wetzlar, Germany) using a 63x objective.

### Real-Time PCR

Total RNA was extracted from silenced cells as indicated above. cDNA preparation was performed using the i-Script reverse transcription Supermix RT-qPCR (1708841, Biorad, Hercules, CA, USA) according to manufacturer’s instructions with the Veriti 96 well Thermal Cycler (Applied Biosystems, Thermo Fisher Scientific). Quantitative Real-Time PCR was performed on 30 ng cDNA/reaction by applying the iTaq Universal SYBR Green Supermix (#1725121 Biorad) and 500 nM of the following primers:

TBP (Prime Time, Hs.PT.58 v.39858774, Integrated DNA Technologies, Iowa, USA), GLUT1 F (5′-GGCCATCTTTTCTGTTGGGG-3′), GLUT1 R (5′-GCTGATGATGAACCTGCTGG-3′), (Thermo Fisher Scientific), and run in QuantStudio 7 Pro Real-Time PCR system.

### Protein studies

Deglycosylation of total lysates (Fig. 5B and Supplementary Fig. S4) was performed using the Enzymatic deglycosylation kit for N-linked and simple O-linked glycans from Agilent (GK80110). Briefly, cells were lysed in 60 µl of ice-cold lysis buffer (50 mM Tris HCl pH 7.4, 300 mM NaCl, 2 mM EDTA pH 8, 2% NP-40, 10% glycerol) supplemented with protease inhibitors (P8340, Sigma-Aldrich) and phosphatase inhibitors (04906837001, Roche, Basilea, Switzerland) by scraping them on ice. Cell lysate was incubated on ice for 20 min and then centrifuged (10,000 × *g*, 10 min, 4 °C). Proteins were quantified with BCA Protein Assay Kit (23225, Thermo Fisher Scientific). One hundred μg of proteins were dissolved in 30 µl of deionized water plus 10 μl of 5× Incubation Buffer (0.25 M sodium phosphate, pH 7.0) and 2.5 µl of denaturation solution (4% sodium dodecyl sulfate and 1 M beta-mercaptoethanol) and heated at 100 °C for 5 min. After addition of 2.5 μl of detergent solution (15% NP-40), the samples were incubated with 1 µl of N-Glycanase (≥5 U/ml), Sialidase A (≥5 U/ml) and O-Glycanase (≥5 U/ml) for 3 h at 37 °C.

Immunoprecipitation experiments were performed by using Pierce Magnetic HA-Tag IP/Co-IP Kit (88838, Thermo Fisher Scientific). MDA-MB-468 were plated in 100 mm dishes (1.5 × 10^6^ cells/plate) two days before the experiment. Cells were washed twice with ice-cold PBS and lysed in 800 μl of ice-cold lysis buffer (50 mM Tris HCl pH7.4, 300 mM NaCl, 2 mM EDTA pH 8, 2% NP-40, 10% glycerol) supplemented with protease and phosphatase inhibitors, by scraping on ice. The lysates were centrifuged at 13,000 × *g* for 10 min. For immunoprecipitation, 1 mg of total lysate was incubated with 25 μl of pre-washed Pierce Anti-HA Magnetic Beads for 30 min at RT with gentle shaking. Beads were collected using a magnetic stand and washed three times with 500 μl of lysis buffer. Protein complexes were eluted by boiling the samples in LDS sample buffer (B0007, Thermo Fisher Scientific, supplemented with 50 mM DTT) at 96 °C for 10 min.

For the IB shown in Figs. 7A and 8D, total lysates were obtained by scraping cells in hot lysis buffer (125 mM Tris HCl pH 6.8 and 2.5% SDS) and boiled for 10 min at 96 °C. Samples were sonicated and centrifuged at 14 000 rpm for 5 min. For the IB shown in Fig. 8A, B, F cells were lysed in ice-cold RIPA buffer containing 50 mM Tris HCl pH 7.4, 150 mM NaCl, 1 mM EDTA, 1% NP-40, 0.5% sodium deoxycholate 0.1% SDS supplemented with protease and phosphatase inhibitors. Proteins were separated on 4-12% NuPAGE Bis-Tris or 4–12% Novex Tris Glycine or 12% Novex Tris Glycine gels (Thermo Fisher Scientific), transferred to nitrocellulose membrane with Trans-Blot Turbo 0.2 µm Nitrocellulose Transfer Packs **(**#1704158, Biorad) and revealed with the indicated antibodies.

Uncropped version of the IB shown in this study are in Supplementary Fig. S6.

### Immunohistochemistry

For TBC1D7 IHC, we employed samples arrayed on tissue microarrays. The rabbit monoclonal antibody anti-TBC1D7 (D8K1Y) (#14949S from Cell Signaling) was used at a final dilution of 1:100 and was unmasked with EDTA pH 9.0 (Bond Epitope Retrieval Sol2, Leica Biosystems, Nussloch, Germany, AR9640). IHC were performed using Bond III IHC auto-stainer (Leica Biosystems) and were acquired with an Aperio ScanScope XT instrument. Samples were scored on a semi-quantitative scale from 0 to 3 (Supplementary Fig. S2), and classified as TBC1D7-HIGH (IHC score ≥ 1) and TBC1D7-LOW (IHC score < 1). IHC data were available for 82 normal breast and 1398 breast cancers.

### Publicly available datasets

The METABRIC dataset (1904 samples) was obtained through the cBioPortal (2019 freeze, available at https://github.com/cBioPortal/datahub/tree/master/public/brca_metabric) [69, 70]. Data were available as normalized log_2_ intensity values.

BC cell lines RNASeq and metabolomics data were obtained from the Cancer Cell Line Encyclopedia (CCLE) collection (https://sites.broadinstitute.org/ccle/datasets) [55] and from [54]. Cell line metabolomics data were available as log_10_ transformed data.

When raw RNASeq data were available, they were processed with RSEM (version 1.3.3) using Bowtie2 (version 2.4.5) as aligner and the human genome (hg38) as reference.

### Data analysis and statistical methods

Data presented in Fig. 1A and Supplementary Table 1 were obtained via the non-parametric Wilcoxon test using JMP to calculate FC and p-values. The Volcano plot (Fig. 1A) was generated using the ggplot() function in R with the package ggplot2 v. 3.4.4.

Kaplan–Meier analyses depicted in Fig. 1D–F, Fig. 6C, and in Supplementary Fig. S1 were performed within JMP, utilizing the Survival platform. Univariable and multivariable HR and p-values were obtained employing the Cox proportional hazards model, as appropriate within JMP.

The Monte Carlo simulation presented in Fig. 1C was conducted employing the R function replicate().

Hierarchical clustering and Distance-based Dendrograms (Fig. 2) were created using the Ward’s method in cluster analysis within JMP.

In Supplementary Fig. S3A, B, RNAseq data were analyzed with EdgeR package within the R software (version3.40.2). Using default parameters, after filtering for not expressed or low expressed genes, library sizes were normalized and statistical analyses between groups were performed with the quasi-likelihood F-tests (QLF). Differentially expressed genes were obtained and the p-value adjusted with the Benjamini and Hochberg methodology to obtain the FDR (False Discovery Rate). Volcano plots were generated using the ggplot() function in R with the package ggplot2 v. 3.4.4. Data provided in Supplementary Tables 2 and 3 were analyzed using the ‘coxph’ function to ascertain HR (Hazard ratio) and p-values for univariate analysis, utilizing the ‘survival’ package in R, version 3.5–5.

In Fig. 6B, the odds ratio and confidence interval were calculated using the JMP software; p-value was evaluated by Pearson’s Chi Squared test.

Quantification of GLUT1 localization shown in Fig. 5E, F was performed on average Z- stack projections using the Cellpose software which identifies single cells (https://github.com/MouseLand/cellpose) [71]. To calculate the average GLUT1 signal present on the membrane, a 600 nm thick “corona” was created starting from the edge of the cell, cell edges were identified based on the actin staining revealed by phalloidin. The ratio between the average signal in the membrane and in the entire cell was calculated. The intensity analyses were performed using the ImageJ software.

For the L-lactate measurements (Figs. 3A, 7B and 8E), Seahorse experiments (Fig. 4A, B), GLUT1 localization (Fig. 5D) by immunofluorescence and glucose uptake experiments (Fig. 5A), statistical analyses were performed using the Mann-Whitney test, for Real-Time PCR (Fig. 5C) the two-tailed t-test was employed. Unless, otherwise indicated, the results are expressed as mean ± standard deviation (SD).

Supplementary information is available at *Cell Death and Disease’s* website.

## Supplementary information

Supplementary Information Tables and Legends

Supplementary Figure S1

Supplementary Figure S2

Supplementary Figure S3

Supplementary Figure S4

Supplementary Figure S5

Raw data western blot

## Data Availability

All data generated or analyzed during this study are included in this published article (and its supplementary information files). Raw RNA-seq data of MDA-MB-468 cells silenced for TBC1D7 have been deposited at GEO under accession number GSE273321.
