# Supplementary material for: TBC1 domain-containing proteins are frequently involved in triple-negative breast cancers in connection with the induction of a glycolytic phenotype
Source: Cell Death Dis. 2024 Sep 4;15(9):647. doi: 10.1038/s41419-024-07037-2 (PMC11375060; doi:10.1038/s41419-024-07037-2)
Supplement: Supplementary file 1 — Supplementary Information Tables and Legends [file 41419_2024_7037_MOESM1_ESM.doc]

**SUPPLEMENTARY INFORMATION**

**LEGENDS TO SUPPLEMENTARY FIGURES**

**Supplementary Figure S1. KM analysis of the TBCD1 genes identified in Fig. 1B.** The expression of each gene was categorized as HIGH or LOW with respect to the mean expression across all samples. Following this categorization, Kaplan-Meier analyses were performed within JMP, employing the Survival platform.

**Supplementary Figure S2. Intensity scale for the IHC experiments displayed in Fig. 6 of the main text.** Tumors of the IEO cohort were arrayed on tissue microarrays (TMA) and analyzed by IHC. Normal breast tissue samples were included in the TMAs to allow a comparison with tumor samples. In the pictures, representative images of normal breast tissues (two examples, left) and BC FFPE samples (right) stained for TBC1D7 in IHC on TMAs. The IHC scores used for the semi-quantitative evaluation of TBC1D7 levels, in Fig. 6 of the main text, are indicated. Bar, 200 μm.

**Supplementary Figure S3. Schematic summary of the analysis of RNAseq data in MDA-MB-468 silenced for TBCD7 *vs*. control. A.** Volcano plot of the genes over-expressed (red) or under-expressed (blue) at FC ± 2.0, FDR, 0.05. **B.** Volcano plot of the genes over-expressed (red) or under-expressed (blue) at FC ± 1.5, FDR, 0.05. **C.** STRING network analysis of genes whose expression was down-regulated by silencing of TBC1D7 in MDA-MB-468 cells at FC ± 1.5, FDR < 0.05. The search was performed at high-confidence setting (0.7). In the network, the grey scale of the edges indicates the strength of data support. Non-connected nodes are not included in the representation. The “analysis” tool of STRING was used to select GO, KEGG, and Wiki terms/pathways; selected terms are highlighted indicated by a color code. Note the high representation of genes involved in metabolic processes and also a distinct cluster of genes encoding for extracellular matrix (secreted) proteins.

**Supplementary Figure S4. Depletion of TBC1D7 in HeLa cells does not affect glycolysis. A.** Intracellular L-lactate levels were measured in HeLa cells, silenced with TBC1D7 siRNA oligos (siTBC1D7) or with control oligos (siCTRL). Data are expressed as mean ± SD of L-lactate per cell, normalized to the respective siCTRL from 2 independent experiments n=12 technical replicates. **B.** Rate of 2-deoxyglucose (2-DG) uptake measured in HeLa cells silenced with TBC1D7 siRNA oligos (siTBC1D7) or with control oligos (siCTRL). Data are expressed as mean ± SD of 2-DG per cell, normalized to the respective siCTRL from 2 independent experiments, n=12 technical replicates. **C.** IB analysis of TBC1D7 expression levels in the indicated cell lines, silenced with control siRNA (siCTRL) or TBC1D7 siRNA (siTBC1D7). Vinculin, loading control. MW markers are shown in KDa.

**Supplementary Figure S5. The TBC1D7 and TBC1D31 interactomes.** The interactomes of TBC1D7 and TBC1D31 were downloaded from the BIOGRID database [1]. They consisted of 60 and 63 unique proteins, respectively. Proteins present in the Crapome database [2] (http://www.crapome.org/) were eliminated from the two lists (cut-off 25%, i.e., present in 179/716 instances in the Crapome database). This yielded 49 and 57 proteins for TBC1D7 and TBC1D31, respectively. There was no overlap between the two lists, suggesting that the proximal effectors of the two TBC1D proteins are different. We reasoned, however, that a more detailed network analysis might unveil similar pathways regulated by the two TBC1Ds. To this purpose, we performed a STRING analysis [3] (https://string-db.org/) of the two interactomes together.

The combined analysis of the two interactomes is shown in the figure, with TBC1D7-interacting proteins in red, and TBC1D31-interacting proteins in blue. As evident, the two interactomes tended to cluster together individually. The major cluster of TBC1D31 interactors (cluster 1) was enriched in proteins belonging to the ontologies “cilium” and “microtubule organizing center”. This confirmed available evidence that TBC1D31 is involved in the assembly of a centrosomal complex composed of TBC1D31, protein kinase A, the E3 ubiquitin ligase PJA2, and OFD1, which is required for ciliogenesis [4].

TBC1D7 interactors, conversely tended to cluster together in a subnetwork (cluster 2) enriched in the ontologies “regulation of autophagy” and “regulation of TOR signaling”. This confirms the known participation of TBC1D7 to the regulation of mTORC1 activity, through its interaction with the TSC1/TSC2 complex [5-7], and further suggests possible regulation of membrane-associated autophagic pathways. Interestingly, cluster 1 and cluster 2 were networked together by a third subnetwork (Cluster 3), which was enriched in “microtubule organizing center” proteins, created by interactors of both TBC1D7 and TB1D31. Therefore, while the two TBC1D proteins probably regulate ciliogenesis through different molecular mechanisms, they do so by impinging on the same network of protein:protein interactions and regulations. It is also of interest that a small subnetwork (Cluster 4) enriched in “Regulation of NFkB signaling” was identified. This cluster also contained both TBC1D7 and TBC1D31 interactors and would have escaped detection by network analysis of individual sets of interactors (with TBC1D7 or TBC1D31). While the hypothetical impact of TBC1D7 and TBC1D31 on NFkB signaling remains to be experimentally validated, it is noteworthy that there is evidence for regulation of energy metabolism by NFkB [8].

**Supplementary Figure S6. Uncropped IBs.**

**LEGENDS TO SUPPLEMENTARY TABLES**

**Supplementary Table 1. Expression of TBC1D genes in TNBC *vs*. other molecular subtypes of BC.** Complete dataset used to prepare Fig. 1A. Descriptions are as in Fig. 1A. Genes of relevance in Fig. 1A are shaded in red and blue in boldface.

**Supplementary Table 2. Univariate and multivariable analyses for prognostic prediction by TBC1D genes.** Complete dataset used to prepare Fig. 1B. Descriptions are as in Fig. 1B. TBC1 gene expression levels were categorized as either HIGH or LOW relative to the mean expression across all samples in the METABRIC dataset (n=1904). After this classification, the data were subjected to analysis using the 'survival' package in R, version 3.5-5. The ‘coxph’ function was employed to determine HRs and p-values for the univariate and multivariable analyses. Variables used for the multivariable analysis were: age, tumor size, tumor grade, nodal status, ER/PGR status, and HER2 status.

**Supplementary Table 3. Univariate and multivariable analyses for prognostic prediction by TBC1D genes in the different BC molecular subtypes.** The METABRIC dataset was divided into four main BC subtypes: Luminal (n=1370), HER2-amplified (n=127), Luminal B/HER2-amplified (n=108), and TNBC (n=299). TBC1 gene expression levels were categorized as either HIGH or LOW relative to the mean expression across all samples within each group. After this classification, the data were subjected to analysis using the 'survival' package in R, version 3.5-5. The ‘coxph’ function was employed to determine HR and p-values for the univariate and multivariable analyses. Variables used for the multivariable analysis were: age, tumor size, tumor grade, nodal status, ER/PGR status, and HER2 status.

**Supplementary Table 4. Biological characteristics of the 46 BC cell lines used for the orthogonal transcriptomics/metabolomics analysis in Fig. 2.** The 46 BC cell lines for which transcriptomic and metabolomic data are available in the public domain [9, 10] (https://sites.broadinstitute.org/ccle/datasets) are listed, together with their biological properties: ER, estrogen receptor; PR, progesterone receptor; HER2, state of amplification of the *ErbB2* oncogene; Classification (Luminal A, HER2, Luminal B-HER2, Basal-like/TNBC) according to [11].

**Supplementary Table 5. Metabolite data in the 46 BC cell lines used for the orthogonal transcriptomics/metabolomics analysis in Fig. 2.** Metabolite concentrations in the 46 BC cell lines are shown and recorded as log_10_ normalized values. Data are from [10].

**Supplementary Table 6. Transcriptomics data in the 46 BC cell lines and stratification in tertiles based on TBC1D gene expression.** Transcriptomics data for the 46 BC cell lines are from [9]. In the table, the TMM normalized RNASeq data (obtained with the EdgeR R package (version 3.40.2) for each TBC1D gene is provided, along with its classification in Upper or Lower tertiles used for the analysis in Fig. 2 of the main text.

**Supplementary Table 7. Metabolite concentrations in various BC lines, expressed as the FC between the upper and lower tertiles for each TBC1 protein.** Complete dataset used to prepare Fig. 2 (see also Supplementary Tables 4-6 and legend to Fig. 2 for details). All p-values were derived with the non-parametric Wilcoxon test using JMP.

**Supplementary Table 8. Lactate production in MDA-MB-468 TNBC cells silenced for various TBC1D genes.** Data used to prepare Fig. 3A. The TBC1 genes are ranked in order of L-lactate production in the siRNA experiment from the lowest to the highest. The mean L-lactate production normalized to CTRL, the standard deviation (SD) and the p-value calculated using the Mann-Whitney test are reported for each gene (n=2, two independent biological replicates each in duplicate).

**Supplementary Table 9. Univariate and multivariable analyses for prognostic prediction by TBC1D7, analyzed by IHC, in various BC molecular subtypes of the IEO cohort.** Different BC molecular subtypes in the IEO cohort were considered for this analysis: Luminal A (ER+/HER2- Ki-67<14%), Luminal B (ER+/HER2- Ki-67≥14%), HER2+ (HER2-positive) and TNBC (ER-/HER2-). BCs were classified as TBC1D7-HIGH or -LOW based on the two-class model shown in Fig. 6. The data were analyzed using the 'survival' package in R, version 3.5-5. The ‘coxph’ function was employed to determine hazard ratios (HRs) and p-values for the univariate and multivariable analyses. Variables used for the multivariable analysis were: age, tumor size, tumor grade, nodal status, and Ki-67.

**Supplementary Table 10. List of genes encoding glucose transporters, key glycolytic enzymes and lactate dehydrogenase isoforms analyzed in TBC1D7-KD MDA-MB-468 cells.** Data used to prepare Fig. 7D. The expression of 24 genes involved in glucose transport, the indicated steps of the glycolytic pathway, and the conversion of pyruvate to lactate was analyzed in TBC1D7-KD *vs*. control MDA-MB-468 cells. The FC in expression, p-values and FDR values are reported. Some glycolysis-related genes including a number of SLC2A genes (most notably SLC2A4) HK3, GCK, ALDOB, ALDOC, GAPDHS, PGK2, PGAM2, ENO3, PKLR, LDHC, and LDHAL6B were not included in the analysis because they were either not-expressed or expressed at negligible levels (cut-off at least 3 TMMs in at least one replicate, out of three, in either control or TBC1D7-KD cells). Genes with significant FDR and p-values (< 0.05) are highlighted in yellow. Genes with significant p-values (< 0.05) but FDR values > 0.05 are highlighted in gray.

**Supplementary Table 11. Gene ID and siRNA oligos.** Gene ID and catalogue number of siRNA oligos employed in the study.

**REFERENCES TO SUPPLEMENTARY INFORMATION**

1. Oughtred R, Rust J, Chang C, Breitkreutz BJ, Stark C, Willems A, et al. The BioGRID database: A comprehensive biomedical resource of curated protein, genetic, and chemical interactions. Protein Sci. 2021;30:187-200.

2. Mellacheruvu D, Wright Z, Couzens AL, Lambert JP, St-Denis NA, Li T, et al. The CRAPome: a contaminant repository for affinity purification-mass spectrometry data. Nat Methods. 2013;10:730-36.

3. Szklarczyk D, Kirsch R, Koutrouli M, Nastou K, Mehryary F, Hachilif R, et al. The STRING database in 2023: protein-protein association networks and functional enrichment analyses for any sequenced genome of interest. Nucleic Acids Res. 2023;51: D638-D646.

4. Senatore E, Chiuso F, Rinaldi L, Intartaglia D, Delle Donne R, Pedone E, et al. The TBC1D31/praja2 complex controls primary ciliogenesis through PKA-directed OFD1 ubiquitylation. EMBO J. 2021;40:e106503.

5. Dibble CC, Elis W, Menon S, Qin W, Klekota J, Asara JM, et al. TBC1D7 is a third subunit of the TSC1-TSC2 complex upstream of mTORC1. Mol Cell. 2012;47:535-46.

6. Schrotter S, Yuskaitis CJ, MacArthur MR, Mitchell SJ, Hosios AM, Osipovich M, et al. The non-essential TSC complex component TBC1D7 restricts tissue mTORC1 signaling and brain and neuron growth. Cell Rep. 2022;39:110824.

7. Menon S, Dibble CC, Talbott G, Hoxhaj G, Valvezan AJ, Takahashi H, et al. Spatial control of the TSC complex integrates insulin and nutrient regulation of mTORC1 at the lysosome. Cell. 2014;156:771-85.

8. Eluard B, Thieblemont C, Baud V. NF-kappaB in the New Era of Cancer Therapy. Trends Cancer. 2020;6:677-87.

9. Ghandi M, Huang FW, Jane-Valbuena J, Kryukov GV, Lo CC, McDonald ER, 3rd, et al. Next-generation characterization of the Cancer Cell Line Encyclopedia. Nature. 2019;569: 503-8.

10. Li H, Ning S, Ghandi M, Kryukov GV, Gopal S, Deik A, et al. The landscape of cancer cell line metabolism. Nat Med. 2019;25:850-60.

11. Jiang G, Zhang S, Yazdanparast A, Li M, Pawar AV, Liu Y, et al. Comprehensive comparison of molecular portraits between cell lines and tumors in breast cancer. BMC Genomics. 2016;17 Suppl 7: 525.

**Supplementary Table 1**

| **Symbol** | **FC** | **LogFC** | **P_value** |
| --- | --- | --- | --- |
| **TBC1D10C** | 1.45 | 0.536 | 1.0E-19 |
| **TBC1D7** | 1.43 | 0.516 | 4.0E-52 |
| **USP6NL** | 1.42 | 0.506 | 3.0E-67 |
| **TBC1D4** | 1.35 | 0.433 | 8.0E-38 |
| **TBC1D31** | 1.25 | 0.322 | 5.0E-27 |
| **TBC1D22B** | 1.22 | 0.287 | 2.0E-28 |
| RABGAP1L | 1.12 | 0.164 | 2.0E-14 |
| TBC1D24 | 1.09 | 0.124 | 0.006 |
| TBC1D23 | 1.08 | 0.111 | 9.2E-06 |
| TBC1D1 | 1.05 | 0.070 | 2.3E-08 |
| TBC1D22A | 1.03 | 0.043 | 0.004 |
| TBC1D25 | 1.03 | 0.043 | 0.052 |
| SGSM3 | 1.03 | 0.043 | 0.061 |
| TBC1D2B | 1.02 | 0.029 | 0.289 |
| TBC1D10B | 1.01 | 0.014 | 0.376 |
| TBC1D26 | 1 | 0.000 | 0.514 |
| TBC1D28 | 1 | 0.000 | 0.683 |
| TBC1D21 | 1 | 0.000 | 0.735 |
| TBC1D5 | 0.99 | -0.015 | 0.242 |
| USP6 | 0.99 | -0.015 | 0.778 |
| TBC1D2 | 0.98 | -0.029 | 0.013 |
| RABGAP1 | 0.98 | -0.029 | 0.339 |
| TBC1D12 | 0.97 | -0.044 | 1.8E-07 |
| TBC1D8B | 0.97 | -0.044 | 0.018 |
| TBC1D10A | 0.96 | -0.059 | 3.0E-04 |
| TBC1D20 | 0.96 | -0.059 | 0.025 |
| EVI5 | 0.96 | -0.059 | 0.158 |
| TBC1D32 | 0.95 | -0.074 | 0.017 |
| EVI5L | 0.94 | -0.089 | 3.0E-04 |
| TBC1D15 | 0.94 | -0.089 | 0.004 |
| TBC1D16 | 0.93 | -0.105 | 0.001 |
| TBC1D13 | 0.92 | -0.120 | 7.6E-08 |
| TBC1D17 | 0.91 | -0.136 | 5.0E-15 |
| TBC1D14 | 0.89 | -0.168 | 7.0E-11 |
| TBC1D3C | 0.89 | -0.168 | 5.6E-07 |
| TBC1D3 | 0.88 | -0.184 | 1.4E-06 |
| TBC1D19 | 0.84 | -0.252 | 8.0E-32 |
| TBC1D9B | 0.83 | -0.269 | 3.0E-24 |
| TBC1D8 | 0.82 | -0.286 | 7.0E-20 |
| **SGSM1** | **0.78** | -0.359 | 4.0E-25 |
| **TBCK** | **0.76** | -0.396 | 5.0E-54 |
| **SGSM2** | **0.73** | -0.454 | 1.0E-27 |
| **GRTP1** | **0.69** | -0.535 | 3.0E-33 |
| **TBC1D9** | **0.18** | -24.739 | 5.0E-135 |

**Supplementary Table 2**

|  | **Univariate** | | | **Multivariable** | | |
| --- | --- | --- | --- | --- | --- | --- |
| **Gene** | **HR** | **95% CI** | **P_value** | **HR** | **95% CI** | **P_value** |
| TBC1D31 | 1.710 | 1.459-2.004 | 3.740E-11 | 1.343 | 1.134-1.590 | 0.001 |
| USP6NL | 1.607 | 1.369-1.886 | 6.603E-09 | 1.218 | 1.009-1.469 | 0.040 |
| TBC1D7 | 1.438 | 1.226-1.686 | 8.085E-06 | 1.059 | 0.886-1.265 | 0.529 |
| TBC1D22B | 1.416 | 1.208-1.660 | 1.770E-05 | 1.274 | 1.080-1.503 | 0.004 |
| TBC1D24 | 1.332 | 1.134-1.565 | 4.952E-04 | 1.133 | 0.959-1.338 | 0.142 |
| TBC1D26 | 1.209 | 1.032-1.417 | 0.019 | 1.207 | 1.029-1.415 | 0.020 |
| TBC1D23 | 1.198 | 1.022-1.404 | 0.026 | 1.097 | 0.934-1.288 | 0.258 |
| RABGAP1 | 1.181 | 1.008-1.384 | 0.040 | 1.243 | 1.458-1.059 | 0.008 |
| TBC1D10B | 1.160 | 0.988-1.361 | 0.069 | 1.128 | 0.960-1.325 | 0.144 |
| TBC1D25 | 1.159 | 0.989-1.359 | 0.068 | 1.144 | 0.976-1.342 | 0.097 |
| TBC1D20 | 1.157 | 0.986-1.356 | 0.075 | 1.257 | 1.070-1.475 | 0.005 |
| SGSM1 | 1.150 | 0.970-1.364 | 0.108 | 1.053 | 0.885-1.475 | 0.559 |
| EVI5L | 1.079 | 0.920-1.265 | 0.352 | 1.071 | 0.912-1.475 | 0.403 |
| TBC1D16 | 1.069 | 0.907-1.260 | 0.427 | 0.989 | 0.837-1.167 | 0.894 |
| SGSM3 | 1.061 | 0.903-1.247 | 0.469 | 0.873 | 0.737-1.035 | 0.118 |
| TBC1D15 | 1.061 | 0.903-1.246 | 0.472 | 1.044 | 0.888-1.228 | 0.602 |
| EVI5 | 1.045 | 0.887-1.230 | 0.594 | 1.116 | 0.946-1.315 | 0.192 |
| TBC1D4 | 1.024 | 0.872-1.204 | 0.769 | 0.921 | 0.778-1.315 | 0.345 |
| TBC1D2 | 1.020 | 0.868-1.198 | 0.806 | 1.018 | 0.866-1.196 | 0.825 |
| TBC1D9B | 1.016 | 0.866-1.192 | 0.845 | 1.121 | 0.952-1.319 | 0.171 |
| TBC1D22A | 1.008 | 0.860-1.182 | 0.918 | 1.056 | 0.899-1.239 | 0.508 |
| TBC1D32 | 1.007 | 0.858-1.181 | 0.936 | 1.104 | 0.939-1.298 | 0.230 |
| TBC1D28 | 1.004 | 0.856-1.177 | 0.961 | 0.975 | 0.831-1.144 | 0.756 |
| TBC1D1 | 1.001 | 0.854-1.174 | 0.988 | 0.961 | 0.818-1.128 | 0.623 |
| TBC1D21 | 0.992 | 0.847-1.163 | 0.925 | 0.970 | 0.827-1.138 | 0.711 |
| TBC1D8B | 0.963 | 0.818-1.133 | 0.647 | 1.001 | 0.849-1.179 | 0.995 |
| TBC1D10C | 0.954 | 0.801-1.136 | 0.595 | 0.766 | 0.636-0.922 | 0.005 |
| TBC1D13 | 0.952 | 0.812-1.116 | 0.543 | 1.067 | 0.908-1.254 | 0.431 |
| TBC1D12 | 0.952 | 0.812-1.117 | 0.547 | 0.970 | 0.826-1.140 | 0.715 |
| TBC1D3 | 0.945 | 0.799-1.118 | 0.508 | 0.976 | 0.823-1.156 | 0.776 |
| RABGAP1L | 0.942 | 0.799-1.110 | 0.477 | 0.849 | 0.719-1.003 | 0.055 |
| TBC1D10A | 0.937 | 0.797-1.101 | 0.427 | 0.860 | 0.731-1.012 | 0.069 |
| TBC1D5 | 0.936 | 0.798-1.097 | 0.413 | 1.013 | 0.862-1.190 | 0.874 |
| TBC1D2B | 0.919 | 0.783-1.079 | 0.304 | 0.944 | 0.803-1.110 | 0.485 |
| TBC1D3C | 0.905 | 0.772-1.062 | 0.223 | 0.915 | 0.780-1.074 | 0.277 |
| USP6 | 0.861 | 0.730-1.016 | 0.077 | 0.886 | 0.750-1.046 | 0.153 |
| TBC1D8 | 0.859 | 0.729-1.012 | 0.069 | 0.948 | 0.802-1.120 | 0.529 |
| GRTP1 | 0.831 | 0.705-0.980 | 0.028 | 0.963 | 0.812-1.143 | 0.669 |
| TBC1D17 | 0.808 | 0.686-0.952 | 0.011 | 0.917 | 0.776-1.143 | 0.312 |
| TBCK | 0.800 | 0.681-0.939 | 0.006 | 0.931 | 0.785-1.105 | 0.416 |
| TBC1D19 | 0.781 | 0.664-0.918 | 0.003 | 0.895 | 0.752-1.065 | 0.210 |
| TBC1D14 | 0.732 | 0.624-0.860 | 1.417E-04 | 0.894 | 0.758-1.053 | 0.179 |
| TBC1D9 | 0.729 | 0.617-0.861 | 1.956E-04 | 0.947 | 0.785-1.142 | 0.571 |
| SGSM2 | 0.706 | 0.598-0.861 | 3.950E-05 | 0.944 | 0.793-1.124 | 0.519 |

**Supplementary Table 3**

| **Gene** | **TNBC** | | | | | |
| --- | --- | --- | --- | --- | --- | --- |
|  | **Univariate** | | | **Multivariable** | | |
|  | **HR** | **95% CI** | **P_value** | **HR** | **95% CI** | **P_value** |
| TBC1D31 | 1.145 | 0.788-1.663 | 0.478 | 1.123 | 0.762-1.653 | 0.558 |
| TBC1D22B | 1.109 | 0.764-1.609 | 0.587 | 1.132 | 0.770-1.663 | 0.528 |
| TBC1D24 | 1.013 | 0.687-1.494 | 0.948 | 0.956 | 0.641-1.425 | 0.824 |
| TBC1D7 | 1.465 | 1.010-2.126 | 0.044 | 1.620 | 1.093-2.400 | 0.016 |
| USP6NL | 0.807 | 0.547-1.190 | 0.279 | 0.725 | 0.485-1.085 | 0.118 |
| SGSM2 | 0.983 | 0.674-1.432 | 0.928 | 1.075 | 0.731-1.582 | 0.714 |
| TBC1D14 | 0.717 | 0.490-1.051 | 0.088 | 0.721 | 0.486-1.072 | 0.106 |
| TBC1D19 | 1.321 | 0.910-1.917 | 0.143 | 1.298 | 0.883-1.908 | 0.184 |
| TBC1D9 | 0.942 | 0.632-1.405 | 0.769 | 1.022 | 0.668-1.564 | 0.920 |
| TBCK | 1.209 | 0.835-1.752 | 0.315 | 1.302 | 0.890-1.564 | 0.174 |

| **Gene** | **HER2** | | | | | |
| --- | --- | --- | --- | --- | --- | --- |
|  | **Univariate** | | | **Multivariable** | | |
|  | **HR** | **95% CI** | **P_value** | **HR** | **95% CI** | **P_value** |
| TBC1D31 | 1.000 | 0.597-1.676 | 0.999 | 0.778 | 0.453-1.335 | 0.362 |
| TBC1D22B | 1.327 | 0.812-2.167 | 0.258 | 1.101 | 0.663-1.826 | 0.710 |
| TBC1D24 | 0.889 | 0.524-1.509 | 0.663 | 1.013 | 0.589-1.742 | 0.963 |
| TBC1D7 | 1.311 | 0.797-2.156 | 0.286 | 1.232 | 0.732-2.076 | 0.432 |
| USP6NL | 1.064 | 0.651-1.738 | 0.806 | 0.792 | 0.474-1.322 | 0.372 |
| SGSM2 | 0.787 | 0.473-1.308 | 0.355 | 0.689 | 0.407-1.164 | 0.164 |
| TBC1D14 | 0.622 | 0.379-1.021 | 0.061 | 0.576 | 0.346-0.959 | 0.034 |
| TBC1D19 | 1.530 | 0.935-2.503 | 0.091 | 1.671 | 0.999-2.795 | 0.050 |
| TBC1D9 | 0.809 | 0.447-1.464 | 0.483 | 0.825 | 0.448-1.517 | 0.536 |
| TBCK | 1.130 | 0.692-1.846 | 0.624 | 1.128 | 0.682-1.866 | 0.639 |

| **Gene** | **LUMINAL** | | | | | |
| --- | --- | --- | --- | --- | --- | --- |
|  | **Univariate** | | | **Multivariable** | | |
|  | **HR** | **95% CI** | **P_value** | **HR** | **95% CI** | **P_value** |
| TBC1D31 | 1.822 | 1.491-2.228 | 4.724E-09 | 1.632 | 1.321-2.016 | 5.426E-06 |
| TBC1D22B | 1.401 | 1.146-1.712 | 0.001 | 1.371 | 1.115-2.016 | 0.003 |
| TBC1D24 | 1.313 | 1.073-1.607 | 0.008 | 1.250 | 1.014-1.541 | 0.036 |
| TBC1D7 | 1.282 | 1.048-1.567 | 0.016 | 1.063 | 0.858-1.317 | 0.575 |
| USP6NL | 1.234 | 1.009-1.508 | 0.040 | 1.172 | 0.953-1.440 | 0.132 |
| SGSM2 | 0.810 | 0.659-0.995 | 0.044 | 0.932 | 0.752-1.155 | 0.520 |
| TBC1D14 | 0.861 | 0.704-1.052 | 0.143 | 1.017 | 0.824-1.255 | 0.875 |
| TBC1D19 | 0.903 | 0.738-1.106 | 0.325 | 0.905 | 0.734-1.116 | 0.350 |
| TBC1D9 | 0.915 | 0.744-1.125 | 0.398 | 0.890 | 0.720-1.102 | 0.285 |
| TBCK | 0.964 | 0.788-1.179 | 0.720 | 1.009 | 0.820-1.242 | 0.933 |

| **Gene** | **LUMINAL B-HER2** | | | | | |
| --- | --- | --- | --- | --- | --- | --- |
|  | **Univariate** | | | **Multivariable** | | |
|  | **HR** | **95% CI** | **P_value** | **HR** | **95% CI** | **P_value** |
| TBC1D31 | 1.236 | 0.714-2.139 | 0.449 | 1.182 | 0.668-2.092 | 0.565 |
| TBC1D22B | 1.329 | 0.770-2.294 | 0.307 | 1.527 | 0.859-2.715 | 0.149 |
| TBC1D24 | 1.475 | 0.845-2.575 | 0.172 | 1.326 | 0.739-2.380 | 0.344 |
| TBC1D7 | 0.791 | 0.452-1.384 | 0.411 | 0.712 | 0.391-1.298 | 0.268 |
| USP6NL | 1.343 | 0.775-2.327 | 0.294 | 1.190 | 0.647-2.189 | 0.577 |
| SGSM2 | 0.905 | 0.519-1.579 | 0.726 | 1.016 | 0.559-1.845 | 0.960 |
| TBC1D14 | 1.144 | 0.663-1.976 | 0.628 | 1.430 | 0.786-2.602 | 0.241 |
| TBC1D19 | 0.577 | 0.328-1.016 | 0.057 | 0.620 | 0.346-1.109 | 0.107 |
| TBC1D9 | 0.655 | 0.372-1.153 | 0.143 | 0.718 | 0.397-1.109 | 0.273 |
| TBCK | 0.906 | 0.519-1.578 | 0.726 | 0.801 | 0.452-1.419 | 0.448 |

**Supplementary Table 4. Biological characteristics of the 46 BC cell lines used for the orthogonal transcriptomics/metabolomics analysis in Fig. 2.**

| **Cell line** | **ER** | **PR** | **HER2** | **Classification** |
| --- | --- | --- | --- | --- |
| HDQP1 | - | - | - | Basal-like/TNBC |
| HCC1937 | - | - | - | Basal-like/TNBC |
| MDAMB361 | + | +/- | + | Luminal B-HER2 |
| HCC1143 | - | - | - | Basal-like/TNBC |
| HCC1599 | - | - | - | Basal-like/TNBC |
| HCC202 | - | - | + | HER2 |
| BT20 | - | - | - | Basal-like/TNBC |
| MDAMB468 | - | - | - | Basal-like/TNBC |
| HCC1569 | - | - | + | HER2 |
| HCC1954 | - | - | + | HER2 |
| HCC1806 | - | - | - | Basal-like/TNBC |
| MDAMB134VI | + | - |  | Luminal A |
| HCC1419 | - | - | + | HER2 |
| HCC1187 | - | - | - | Basal-like/TNBC |
| T47D | + | + | - | Luminal A |
| HCC38 | - | - | - | Basal-like/TNBC |
| BT483 | + | +/- | - | Luminal A |
| HCC2218 | - | - | + | HER2 |
| MDAMB175VII | + | - | - | Luminal A |
| EFM19 | + | + | - | Luminal A |
| CAMA1 | + | +/- | - | Luminal A |
| HCC70 | - | - | - | Basal-like/TNBC |
| UACC893 | - | - | + | HER2 |
| MDAMB415 | + | +/- | - | Luminal A |
| HCC1428 | + | + | - | Luminal A |
| BT549 | - | - | - | Basal-like/TNBC |
| SKBR3 | - | - | + | HER2 |
| CAL851 | - | - | - | Basal-like/TNBC |
| CAL148 | - | - | - | Basal-like/TNBC |
| DU4475 | - | - | - | Basal-like/TNBC |
| ZR7530 | + | - | + | Luminal B HER2 |
| CAL120 | - | - | - | Basal-like/TNBC |
| EFM192A | + | + | + | Luminal B-HER2 |
| CAL51 | - | - | - | Basal-like/TNBC |
| AU565 | - | - | + | HER2 |
| BT474 | + | + | + | Luminal B-HER2 |
| ZR751 | + | +/- | - | Luminal A |
| MCF7 | + | + | - | Luminal A |
| JIMT1 | - | - | + | HER2 |
| MDAMB231 | - | - | - | Basal-like/TNBC |
| MDAMB157 | - | - | - | Basal-like/TNBC |
| MDAMB453 | - | - | + | HER2 |
| UACC812 | + | +/- | + | Luminal B-HER2 |
| HMC18 | + | - | - | Luminal A |
| Hs_578T | - | - | - | Basal-like/TNBC |
| HCC1395 | - | - | - | Basal-like/TNBC |

**Supplementary Table 5. Metabolite data in the 46 BC cell lines used for the orthogonal transcriptomics/metabolomics analysis in Fig. 2**

| **Cell line** | **2-aminoadipate** | **3-phosphoglycerate** | **alpha-glycerophosphate** | **4-pyridoxate** | **aconitate** |
| --- | --- | --- | --- | --- | --- |
| HDQP1 | 6.2050036 | 5.9245007 | 5.4558829 | 5.9206584 | 5.9992836 |
| HCC1937 | 6.2001239 | 5.8289575 | 4.4268051 | 7.2222478 | 5.1295171 |
| MDAMB361 | 5.9174547 | 5.1693048 | 5.8677371 | 6.168407 | 5.6565935 |
| HCC1143 | 5.8406197 | 5.1160598 | 5.1802391 | 5.8375498 | 5.6509495 |
| HCC1599 | 5.993698 | 5.6185771 | 5.5189781 | 6.087417 | 5.1178652 |
| HCC202 | 5.7478788 | 5.6858871 | 6.3414695 | 6.2562316 | 5.8926193 |
| BT20 | 6.1878535 | 5.8906493 | 5.9300342 | 6.1741784 | 6.1102248 |
| MDAMB468 | 5.6326833 | 6.0047395 | 6.0224974 | 5.8803712 | 5.7233149 |
| HCC1569 | 5.5015127 | 6.3350544 | 5.1752049 | 5.8601683 | 5.4622635 |
| HCC1954 | 5.850535 | 6.1878567 | 6.0411352 | 5.8871547 | 6.0626892 |
| HCC1806 | 5.7582304 | 6.6153894 | 5.8913195 | 5.5593753 | 6.2732059 |
| MDAMB134VI | 6.0857591 | 5.950295 | 5.0516787 | 5.5988827 | 5.5879331 |
| HCC1419 | 5.3957414 | 6.074301 | 6.1999301 | 6.3559646 | 5.9532328 |
| HCC1187 | 5.400939 | 5.8243835 | 5.5232104 | 5.9330821 | 5.0781915 |
| T47D | 5.5957775 | 5.9552412 | 7.0280981 | 5.4274208 | 5.9935464 |
| HCC38 | 5.7345491 | 5.7412427 | 5.7269337 | 5.5580139 | 5.8373192 |
| BT483 | 5.6412655 | 5.8298271 | 5.6566102 | 6.4909711 | 6.0835659 |
| HCC2218 | 5.8445544 | 5.3810362 | 6.7330424 | 5.8484218 | 5.4550303 |
| MDAMB175VII | 5.613213 | 5.8748001 | 5.9623989 | 6.2580572 | 5.729863 |
| EFM19 | 5.7013517 | 5.6989503 | 6.0870458 | 6.1434253 | 5.818466 |
| CAMA1 | 5.8596956 | 5.7282315 | 6.3723412 | 6.3574195 | 5.7895814 |
| HCC70 | 5.8128008 | 6.0955769 | 5.6250341 | 6.0022976 | 5.7984217 |
| UACC893 | 5.9332516 | 5.5121157 | 5.6321923 | 5.4591183 | 5.3392609 |
| MDAMB415 | 5.773913 | 6.0145419 | 6.3696571 | 6.4698687 | 5.9329369 |
| HCC1428 | 5.8444312 | 5.9963311 | 6.9954677 | 5.882257 | 6.2477777 |
| BT549 | 6.1573476 | 5.6846086 | 5.7714803 | 6.2221549 | 5.732538 |
| SKBR3 | 5.9930355 | 5.9611823 | 6.2394435 | 5.9776763 | 5.8878468 |
| CAL851 | 5.7754076 | 5.9721834 | 5.7336958 | 6.1627616 | 5.5160936 |
| CAL148 | 5.8670333 | 5.4555085 | 5.6432987 | 5.5153609 | 5.5098787 |
| DU4475 | 5.8712732 | 5.6948866 | 6.0426028 | 6.109664 | 5.7490339 |
| ZR7530 | 5.9081323 | 5.8588124 | 6.5147601 | 6.0942251 | 5.5470106 |
| CAL120 | 5.5956755 | 5.8619824 | 5.8839763 | 5.4988265 | 5.9629962 |
| EFM192A | 5.8631621 | 5.9425659 | 6.0331119 | 6.3172582 | 5.8624812 |
| CAL51 | 6.0286516 | 5.852998 | 5.9149614 | 6.3361487 | 5.7998921 |
| AU565 | 5.5852186 | 5.9004505 | 6.1794771 | 6.2088946 | 6.2313726 |
| BT474 | 5.5371003 | 5.7286179 | 5.7359337 | 5.9833485 | 5.6789317 |
| ZR751 | 5.666087 | 6.2128096 | 6.2231168 | 6.1166083 | 6.0215988 |
| MCF7 | 5.8008271 | 5.8371813 | 5.8600887 | 6.1057 | 5.8003542 |
| JIMT1 | 5.8641372 | 6.5619078 | 6.2507909 | 5.6799012 | 6.110031 |
| MDAMB231 | 5.6712545 | 6.0389753 | 5.9560394 | 6.0549252 | 5.7885404 |
| MDAMB157 | 5.9738029 | 5.8100213 | 6.5992324 | 5.4544503 | 5.8859053 |
| MDAMB453 | 5.6738042 | 5.983045 | 6.5334485 | 6.0817591 | 5.9335927 |
| UACC812 | 5.9849992 | 5.3710441 | 6.388998 | 6.4060514 | 5.8127056 |
| HMC18 | 6.2610398 | 5.7663056 | 4.8729745 | 6.2984637 | 5.0599287 |
| Hs_578T | 6.0622178 | 5.7744449 | 5.7661035 | 6.1233815 | 5.4406446 |
| HCC1395 | 6.083912 | 5.3869397 | 5.5029844 | 6.2209261 | 5.0851674 |

| **Cell line** | **adenine** | **adipate** | **alpha-ketoglutarate** | **AMP** | **citrate** | **isocitrate** | **CMP** | **cystathionine** |
| --- | --- | --- | --- | --- | --- | --- | --- | --- |
| HDQP1 | 5.4982906 | 5.6978448 | 5.8795035 | 5.5503957 | 6.1267945 | 6.1449642 | 5.5578974 | 6.7345022 |
| HCC1937 | 5.7420075 | 6.0944082 | 5.3984364 | 6.0347302 | 4.8018254 | 4.8408473 | 5.7619536 | 5.7054331 |
| MDAMB361 | 6.4376014 | 5.8379522 | 5.3239582 | 5.5086462 | 5.7671702 | 5.7179016 | 5.6742913 | 5.3261172 |
| HCC1143 | 5.4325614 | 5.9749462 | 5.5244609 | 5.4929729 | 5.3517562 | 5.3948032 | 5.7043914 | 5.7579299 |
| HCC1599 | 6.1474527 | 5.9458005 | 5.3630334 | 6.2741239 | 5.2608395 | 5.226794 | 6.5040716 | 6.3764693 |
| HCC202 | 5.7422681 | 5.7764063 | 5.3815946 | 5.7608082 | 5.7139141 | 5.8925562 | 5.8573228 | 5.9582016 |
| BT20 | 5.972076 | 6.0742882 | 6.3532011 | 6.1283346 | 6.1979999 | 6.0795971 | 5.9558546 | 7.0558405 |
| MDAMB468 | 5.6458228 | 5.8385227 | 6.0494434 | 5.6956799 | 5.7903062 | 5.8287001 | 5.6135931 | 5.8784187 |
| HCC1569 | 5.5496852 | 5.7200442 | 5.9839495 | 6.4108089 | 5.532499 | 5.5756701 | 6.3227149 | 6.210004 |
| HCC1954 | 5.2496252 | 5.9050183 | 6.1193312 | 5.7738744 | 6.0646505 | 6.0560855 | 5.7861337 | 5.9820875 |
| HCC1806 | 5.745104 | 5.8986434 | 5.8404068 | 5.8035033 | 6.4002404 | 6.365772 | 5.8752774 | 6.1916021 |
| MDAMB134VI | 5.6941173 | 6.2259229 | 5.5692705 | 6.009754 | 5.6759957 | 5.6663044 | 6.3513453 | 6.4869686 |
| HCC1419 | 6.242203 | 5.6835432 | 5.8345508 | 6.1147068 | 5.7408131 | 5.8620382 | 6.1799086 | 6.9470828 |
| HCC1187 | 5.5288895 | 5.9335621 | 5.4249165 | 5.8562367 | 4.7179293 | 4.9872707 | 5.9232066 | 5.4472728 |
| T47D | 5.7512361 | 5.7364901 | 6.0937785 | 6.0535054 | 5.8541053 | 5.8649837 | 6.1423536 | 5.4443633 |
| HCC38 | 6.1745419 | 5.922278 | 5.7510826 | 5.7789048 | 5.6754929 | 5.9979777 | 5.9935177 | 5.6971193 |
| BT483 | 6.0103862 | 5.8214044 | 5.3219474 | 6.1303232 | 6.039078 | 5.8575073 | 5.785997 | 6.5268058 |
| HCC2218 | 5.6608082 | 5.7023592 | 5.6819803 | 6.5462429 | 5.6858947 | 5.5301055 | 6.3945315 | 6.1302285 |
| MDAMB175VII | 5.4322757 | 5.9434162 | 5.7673683 | 5.6806464 | 5.7596912 | 5.5867789 | 5.8720004 | 5.9651578 |
| EFM19 | 5.5145142 | 5.7864224 | 5.7059484 | 5.8317059 | 5.7682436 | 5.7019375 | 5.8096903 | 5.1318472 |
| CAMA1 | 5.0904377 | 5.9406808 | 5.5597898 | 6.2511525 | 5.8948514 | 5.9013888 | 5.9568675 | 6.0805847 |
| HCC70 | 5.8989909 | 5.9748393 | 5.8017947 | 6.0688671 | 5.6491524 | 5.7376778 | 5.9054919 | 5.3470102 |
| UACC893 | 5.6673876 | 6.1003684 | 5.6016808 | 5.9487195 | 5.0977188 | 5.2207807 | 6.2819859 | 4.8657219 |
| MDAMB415 | 5.8259666 | 5.8001348 | 5.8537345 | 6.1323436 | 5.9537783 | 5.9644823 | 5.9143964 | 5.1330625 |
| HCC1428 | 6.0479291 | 5.9003246 | 6.1453559 | 6.2320467 | 6.1243835 | 6.232446 | 5.7114285 | 5.3274363 |
| BT549 | 5.9447193 | 6.096567 | 5.8284149 | 5.6668427 | 5.7725887 | 5.8821695 | 5.5654117 | 6.1536886 |
| SKBR3 | 5.7043758 | 5.9475389 | 5.824698 | 5.5638499 | 5.7441205 | 5.7101585 | 5.7938727 | 5.0963522 |
| CAL851 | 5.1400076 | 5.7881559 | 5.9361959 | 5.6071627 | 5.6898636 | 5.7401248 | 5.7323983 | 5.8218044 |
| CAL148 | 5.8299808 | 6.2582045 | 5.5419128 | 5.8364455 | 5.2614249 | 5.3725068 | 6.1151484 | 5.9542344 |
| DU4475 | 6.5727223 | 5.9429215 | 5.852313 | 6.4049428 | 5.7843095 | 5.6766189 | 6.3456258 | 5.6486174 |
| ZR7530 | 5.2880152 | 6.0462492 | 5.5937348 | 6.099759 | 5.6280892 | 5.6188417 | 5.5962041 | 5.3669999 |
| CAL120 | 5.8879702 | 5.7939302 | 6.0604592 | 5.7166574 | 5.9116307 | 5.8940643 | 5.9753709 | 6.4465935 |
| EFM192A | 5.951045 | 5.9695199 | 5.8494994 | 5.6041232 | 5.8112087 | 5.8545291 | 5.9059263 | 5.6123582 |
| CAL51 | 5.6093793 | 5.9965518 | 5.9357611 | 5.5763583 | 5.8773758 | 5.7636696 | 5.6844887 | 6.4785807 |
| AU565 | 5.5550944 | 5.8495591 | 6.0230032 | 6.0441129 | 6.1210318 | 6.203298 | 5.9400683 | 5.1843234 |
| BT474 | 5.6033143 | 5.9848891 | 5.7825448 | 6.0502065 | 5.7126049 | 5.7322608 | 5.6729431 | 6.3196927 |
| ZR751 | 6.0185049 | 5.8753821 | 6.0976035 | 6.6082363 | 5.9402451 | 6.0796969 | 6.0144209 | 5.3561163 |
| MCF7 | 5.7537761 | 5.7839539 | 6.1606413 | 5.9170566 | 5.86988 | 5.9168327 | 5.9593059 | 6.8672043 |
| JIMT1 | 5.7670684 | 5.8792183 | 6.3279291 | 5.8839763 | 5.944206 | 6.010346 | 6.0578948 | 6.4240871 |
| MDAMB231 | 5.8736606 | 5.9250106 | 5.8780166 | 5.4495678 | 5.8477867 | 5.8998311 | 5.6619553 | 5.0357524 |
| MDAMB157 | 5.6509554 | 5.9189016 | 5.5778585 | 5.7571266 | 5.6897533 | 5.7082455 | 6.0296067 | 6.2723704 |
| MDAMB453 | 5.4465847 | 5.9184212 | 5.9224041 | 5.9882742 | 5.896858 | 5.983143 | 5.9855267 | 6.9985504 |
| UACC812 | 6.7071705 | 5.8275351 | 6.0628038 | 5.5867496 | 5.8762704 | 5.8266853 | 5.5639624 | 5.9714651 |
| HMC18 | 6.4301558 | 5.9602735 | 5.2856766 | 5.8105653 | 4.8673669 | 5.1154414 | 6.1077174 | 6.5087512 |
| Hs_578T | 5.9785138 | 6.0380017 | 5.8970425 | 5.5014454 | 5.5886783 | 5.592453 | 5.7384289 | 6.1227042 |
| HCC1395 | 5.7687994 | 5.9204546 | 5.6484655 | 5.4966255 | 5.5499341 | 5.6966437 | 6.0586942 | 5.1187749 |

| **Cell line** | **cytidine** | **dCMP** | **DHAP/ glyceraldehyde 3P** | **erythrose-4-phosphate** | **F1P/F6P/G1P/G6P** | **hexoses (HILIC neg)** |
| --- | --- | --- | --- | --- | --- | --- |
| HDQP1 | 5.030569 | 5.4744363 | 5.9394292 | 5.8137466 | 6.1844685 | 6.0316104 |
| HCC1937 | 5.4826917 | 6.2790503 | 5.6456237 | 5.4236407 | 4.9004841 | 6.2195738 |
| MDAMB361 | 6.5318538 | 5.8002177 | 5.4611465 | 5.7554385 | 5.7398708 | 5.9743336 |
| HCC1143 | 5.851742 | 5.474059 | 5.7481176 | 5.4660079 | 5.1525954 | 6.1917335 |
| HCC1599 | 6.5099778 | 6.162269 | 5.1757087 | 5.7160443 | 5.3428312 | 4.9219052 |
| HCC202 | 6.3567841 | 5.81091 | 5.7299601 | 5.6969285 | 5.8583234 | 6.0263114 |
| BT20 | 5.7127976 | 5.8544552 | 5.894793 | 5.8813495 | 5.6861049 | 5.3216912 |
| MDAMB468 | 5.5420025 | 5.9058901 | 6.2067624 | 6.0126974 | 6.2069197 | 6.1153476 |
| HCC1569 | 5.4658441 | 5.9592105 | 6.0432341 | 5.6839537 | 5.6466074 | 4.8521366 |
| HCC1954 | 5.8665686 | 5.6013921 | 6.1605375 | 5.8158452 | 5.9643682 | 5.7249431 |
| HCC1806 | 4.6084618 | 5.7873934 | 6.6225643 | 5.9608209 | 6.0954604 | 5.8910811 |
| MDAMB134VI | 5.5433131 | 6.7971454 | 5.3723137 | 5.8839763 | 6.1427505 | 5.2253766 |
| HCC1419 | 6.5994851 | 5.8392162 | 6.003221 | 6.0103347 | 5.9698243 | 5.8042141 |
| HCC1187 | 6.0675214 | 6.1270894 | 5.9815809 | 5.7082868 | 5.6981967 | 5.1558648 |
| T47D | 6.1585449 | 5.7534696 | 5.9994586 | 5.6921326 | 5.7284399 | 5.8420389 |
| HCC38 | 6.4661537 | 5.8958182 | 5.5494152 | 5.8380079 | 5.649581 | 5.9070708 |
| BT483 | 6.822771 | 5.7271687 | 5.4625055 | 5.3891685 | 5.5622876 | 5.8661595 |
| HCC2218 | 5.3233526 | 6.129552 | 6.1232294 | 6.3060667 | 6.2453722 | 4.6454762 |
| MDAMB175VII | 6.3348627 | 5.6916035 | 5.5377402 | 6.0138378 | 5.6500967 | 6.0231553 |
| EFM19 | 6.1523155 | 5.9206501 | 5.77912 | 5.8627994 | 5.6660418 | 6.0156105 |
| CAMA1 | 5.5865952 | 5.9914023 | 5.8527157 | 6.0296424 | 6.1472912 | 5.3288906 |
| HCC70 | 5.3835846 | 5.4016787 | 5.933612 | 5.7845943 | 5.7678061 | 5.9304246 |
| UACC893 | 5.4514407 | 5.8864386 | 5.6041879 | 6.1249839 | 6.0308122 | 5.1309834 |
| MDAMB415 | 5.247131 | 5.8070267 | 6.0362432 | 5.9689071 | 5.990141 | 5.897339 |
| HCC1428 | 5.5792631 | 6.1674596 | 6.0558439 | 6.3918277 | 6.2702426 | 5.7651696 |
| BT549 | 6.5380051 | 5.643745 | 6.1261087 | 5.6919903 | 5.6869773 | 6.1624182 |
| SKBR3 | 6.5205911 | 5.4538162 | 5.8810374 | 6.1333462 | 6.0045896 | 6.2129365 |
| CAL851 | 4.9878843 | 5.9065768 | 6.0633028 | 5.8642542 | 6.0728738 | 6.0801796 |
| CAL148 | 5.4657349 | 5.9509626 | 5.4267287 | 5.8839763 | 5.5209044 | 5.7695557 |
| DU4475 | 5.6515692 | 6.1113443 | 5.2557814 | 5.9061285 | 5.6576883 | 5.0638056 |
| ZR7530 | 5.4729364 | 5.434923 | 5.8282438 | 5.8839763 | 5.9634475 | 6.03104 |
| CAL120 | 5.4423942 | 5.8011697 | 5.9347166 | 6.0488458 | 6.1157833 | 6.1053593 |
| EFM192A | 5.1331943 | 5.7657762 | 5.8175446 | 5.9101819 | 5.8469944 | 6.009901 |
| CAL51 | 6.4304472 | 5.8480037 | 5.9541462 | 5.7014643 | 5.9680939 | 6.1436978 |
| AU565 | 5.8110518 | 5.9230645 | 5.7840923 | 6.366342 | 6.3277778 | 6.0401223 |
| BT474 | 5.8562729 | 5.1693786 | 5.9217357 | 5.8850258 | 5.8755521 | 6.234461 |
| ZR751 | 6.2642676 | 5.7145668 | 6.1306664 | 6.0234556 | 6.0228441 | 5.5832036 |
| MCF7 | 6.9147336 | 6.1336103 | 5.5950743 | 5.8114172 | 5.6409937 | 5.7855879 |
| JIMT1 | 4.8071989 | 5.7614739 | 6.672814 | 6.2769305 | 6.0599567 | 6.0468417 |
| MDAMB231 | 5.2919798 | 5.8675504 | 6.3107413 | 5.7431265 | 5.8886653 | 5.9877007 |
| MDAMB157 | 6.2338087 | 5.7903947 | 5.827161 | 5.6448042 | 5.5956644 | 5.9708679 |
| MDAMB453 | 6.3204308 | 5.8840224 | 5.798838 | 6.0927023 | 6.2395141 | 6.0430889 |
| UACC812 | 5.9833028 | 5.4392724 | 5.3096498 | 5.8336224 | 5.6585558 | 6.0033497 |
| HMC18 | 5.5304525 | 6.3890155 | 5.4425237 | 5.9359196 | 5.3297833 | 4.9746118 |
| Hs_578T | 5.7419251 | 5.9109165 | 6.357838 | 5.6759065 | 5.8839763 | 6.1956511 |
| HCC1395 | 5.5404313 | 6.2808652 | 5.5765959 | 5.8239595 | 5.5517257 | 6.0372287 |

| **Cell line** | **fumarate/maleate/ alpha-ketoisovalerate** | **glucuronate** | **glutathione oxidized** | **glutathione reduced** | **GMP** | **guanosine** |
| --- | --- | --- | --- | --- | --- | --- |
| HDQP1 | 5.7604585 | 5.9969807 | 6.0417591 | 5.9894478 | 5.7940339 | 5.9794028 |
| HCC1937 | 5.5904898 | 6.4459147 | 5.0029491 | 5.0459701 | 5.8890452 | 5.9244078 |
| MDAMB361 | 5.4608479 | 5.9064728 | 5.3478031 | 5.4181393 | 5.6719247 | 5.9417519 |
| HCC1143 | 5.6499008 | 6.1155856 | 5.7350968 | 5.3778589 | 5.3649851 | 5.4001972 |
| HCC1599 | 5.7140307 | 5.6280462 | 5.7845975 | 5.9904015 | 6.0839581 | 6.5386318 |
| HCC202 | 5.6270253 | 5.6768512 | 5.2266875 | 5.2915536 | 5.8584672 | 6.1716674 |
| BT20 | 6.1796808 | 5.7820272 | 6.379209 | 6.3674836 | 6.148981 | 5.3003853 |
| MDAMB468 | 5.8964732 | 5.5945864 | 5.9569222 | 5.7973835 | 5.7633207 | 5.5282911 |
| HCC1569 | 5.7142127 | 6.0953623 | 5.5714067 | 5.7070802 | 6.4059269 | 5.9053299 |
| HCC1954 | 6.0738859 | 6.1311099 | 6.3258238 | 6.2693956 | 5.9690535 | 5.9840541 |
| HCC1806 | 5.9637956 | 6.0877465 | 6.3286356 | 6.4231243 | 5.7298002 | 5.6955379 |
| MDAMB134VI | 5.6211211 | 5.3000035 | 5.1769457 | 5.3949982 | 5.7698586 | 6.4686443 |
| HCC1419 | 5.8899686 | 6.0282476 | 6.3103053 | 6.00603 | 6.6095346 | 6.1530267 |
| HCC1187 | 5.7624082 | 5.67942 | 5.1800985 | 5.3154536 | 5.509735 | 5.9106925 |
| T47D | 6.1568471 | 5.7060254 | 5.7587906 | 5.8552016 | 5.7173761 | 5.6519945 |
| HCC38 | 5.8022657 | 5.6362736 | 5.708556 | 5.5350515 | 5.8288292 | 5.5711092 |
| BT483 | 5.5612919 | 5.8887803 | 6.0993986 | 5.7824925 | 6.057543 | 6.1917811 |
| HCC2218 | 5.9002142 | 5.7997737 | 5.6774444 | 5.7513123 | 6.2960084 | 5.7874478 |
| MDAMB175VII | 5.6191925 | 5.9743567 | 5.8429858 | 5.2393273 | 6.1276982 | 6.2910184 |
| EFM19 | 5.4162134 | 5.5772264 | 5.0941862 | 5.1373863 | 5.9825349 | 5.5371378 |
| CAMA1 | 5.8544012 | 5.6085033 | 5.6156803 | 5.7650696 | 6.3353239 | 5.7358978 |
| HCC70 | 5.9014451 | 5.6417342 | 5.7620134 | 5.9123472 | 5.945663 | 5.8142843 |
| UACC893 | 5.749963 | 5.2577057 | 5.0681846 | 5.5597649 | 6.1213681 | 6.1679574 |
| MDAMB415 | 5.6532126 | 5.9297582 | 6.3607821 | 5.7710811 | 6.141118 | 6.1202521 |
| HCC1428 | 6.1715569 | 5.8564573 | 5.9905879 | 6.1312058 | 5.9657285 | 5.7836737 |
| BT549 | 5.7325093 | 5.9332489 | 5.5546869 | 5.3903481 | 5.6312044 | 5.8084001 |
| SKBR3 | 5.752199 | 5.6417614 | 5.8613217 | 5.2444303 | 6.1431233 | 6.0413674 |
| CAL851 | 5.9541464 | 5.7410284 | 5.9007042 | 5.9934741 | 5.5606108 | 5.5633021 |
| CAL148 | 5.9844891 | 5.6839667 | 5.3170844 | 5.5766804 | 5.6677462 | 6.1262491 |
| DU4475 | 6.2690438 | 5.7446867 | 6.5637508 | 6.3261186 | 6.027652 | 5.9608242 |
| ZR7530 | 5.5571064 | 5.7459695 | 5.8298354 | 5.6621205 | 6.1077588 | 5.5023995 |
| CAL120 | 5.7697584 | 5.7651344 | 5.9892815 | 5.8571703 | 5.7718319 | 6.7836343 |
| EFM192A | 5.7014584 | 6.0479975 | 5.8518106 | 5.5861485 | 6.0215469 | 5.656364 |
| CAL51 | 5.8374464 | 6.043112 | 5.8544537 | 5.8879638 | 5.6081156 | 6.4632741 |
| AU565 | 5.8292271 | 5.8034933 | 5.8707702 | 5.6370432 | 6.096262 | 5.6558463 |
| BT474 | 5.8090068 | 5.7669798 | 5.7691206 | 5.699332 | 5.8008745 | 6.2959611 |
| ZR751 | 5.9920792 | 5.5911192 | 5.8325934 | 6.0376663 | 6.4330666 | 5.8998819 |
| MCF7 | 6.0878216 | 5.9226987 | 6.0399069 | 6.0397732 | 6.0734662 | 6.9049907 |
| JIMT1 | 6.1273975 | 5.7966515 | 6.3646129 | 6.2311137 | 5.9349771 | 5.7099885 |
| MDAMB231 | 5.8563946 | 6.0568811 | 5.7747702 | 5.8839763 | 5.2993264 | 6.1551494 |
| MDAMB157 | 5.9825711 | 5.8102849 | 5.295952 | 5.4098042 | 5.5522366 | 5.6437799 |
| MDAMB453 | 5.9062457 | 5.5894358 | 6.1990102 | 6.0690694 | 5.9745024 | 5.5879644 |
| UACC812 | 5.7874599 | 6.0915343 | 4.768428 | 4.9909205 | 5.7438652 | 5.8024214 |
| HMC18 | 4.9136563 | 5.498894 | 5.4980682 | 5.4101383 | 5.7411453 | 6.6123283 |
| Hs_578T | 5.5745579 | 6.0541972 | 5.8514619 | 5.8830907 | 5.9173078 | 6.0937124 |
| HCC1395 | 5.6756062 | 5.548716 | 6.069344 | 5.9928515 | 5.606908 | 5.9759767 |

| **Cell line** | **hippurate** | **hypoxanthine** | **inosine** | **kynurenine** | **lactate** | **lactose** | **malate** |
| --- | --- | --- | --- | --- | --- | --- | --- |
| HDQP1 | 5.8899994 | 5.0777782 | 6.0662019 | 5.9504326 | 5.9980995 | 6.1437655 | 5.8275083 |
| HCC1937 | 6.1991428 | 5.8826539 | 6.5018931 | 6.323844 | 6.6315858 | 5.6760307 | 5.5037345 |
| MDAMB361 | 6.1557547 | 6.7187428 | 6.1443691 | 5.5178802 | 5.6305866 | 7.9821226 | 5.3126875 |
| HCC1143 | 6.2531186 | 6.3467748 | 5.9122747 | 5.6290791 | 5.9588522 | 6.4917194 | 5.6661898 |
| HCC1599 | 5.2341721 | 6.0673053 | 6.2888769 | 5.4703808 | 5.6100173 | 5.0879359 | 5.7484212 |
| HCC202 | 6.1534211 | 6.6280858 | 5.9301433 | 5.2354944 | 5.9031304 | 5.9044306 | 5.6774407 |
| BT20 | 5.9163804 | 5.0357041 | 5.3297376 | 5.0363846 | 6.0721187 | 5.8132664 | 6.1817544 |
| MDAMB468 | 5.7651066 | 6.2578656 | 5.7264475 | 6.0304368 | 5.7777581 | 6.1112157 | 5.9456271 |
| HCC1569 | 5.1771478 | 5.1815951 | 5.863515 | 5.7564808 | 5.8616226 | 5.2527297 | 5.7682452 |
| HCC1954 | 5.7592467 | 6.0674794 | 5.7831689 | 6.506444 | 5.9824393 | 6.357128 | 6.0757567 |
| HCC1806 | 5.9121165 | 4.9215658 | 5.5945084 | 5.9182789 | 6.1888266 | 6.1234139 | 6.0109378 |
| MDAMB134VI | 5.5594768 | 5.9238181 | 5.638499 | 6.2347662 | 4.9825523 | 5.1883011 | 5.5897761 |
| HCC1419 | 5.7228078 | 6.5445671 | 5.7320738 | 5.1533534 | 5.6953076 | 5.8847745 | 6.0308714 |
| HCC1187 | 5.0576176 | 5.7626687 | 5.6061258 | 6.264731 | 5.7588887 | 5.6632676 | 4.9924893 |
| T47D | 5.6648963 | 5.8231582 | 5.8301025 | 5.9082301 | 5.8237887 | 5.7294347 | 6.1548992 |
| HCC38 | 5.8527397 | 5.3986654 | 5.658465 | 6.0442197 | 5.7036279 | 5.7800502 | 5.7710223 |
| BT483 | 6.1995128 | 6.8370124 | 6.0023438 | 5.506247 | 5.6369116 | 6.0994919 | 5.6404863 |
| HCC2218 | 5.2353341 | 6.5452892 | 6.6004652 | 6.0764448 | 5.9177847 | 4.9878079 | 5.9217191 |
| MDAMB175VII | 6.1253307 | 6.573741 | 6.2230695 | 5.2470177 | 5.8026488 | 5.6101493 | 5.656063 |
| EFM19 | 5.9078494 | 5.9137616 | 5.8400529 | 5.7116064 | 5.633259 | 5.3495286 | 5.3820763 |
| CAMA1 | 5.8062082 | 5.7926513 | 5.8894317 | 5.6139916 | 5.7804211 | 6.2420895 | 5.8594725 |
| HCC70 | 5.6316994 | 4.7829366 | 5.5181972 | 5.8027996 | 5.9661161 | 5.4842518 | 5.9210285 |
| UACC893 | 5.3113564 | 5.6920666 | 5.4737764 | 6.3184561 | 5.083441 | 5.4165106 | 5.7098622 |
| MDAMB415 | 6.1792125 | 6.5375436 | 6.041859 | 5.3965107 | 6.0175613 | 5.8116091 | 5.6880029 |
| HCC1428 | 5.9192556 | 5.2780049 | 5.6552587 | 5.3078986 | 5.9526142 | 5.3572922 | 6.1929177 |
| BT549 | 6.3021107 | 6.7024072 | 6.3280526 | 6.0007456 | 5.8018518 | 5.8098069 | 5.6340995 |
| SKBR3 | 6.1559198 | 6.4644747 | 6.2041172 | 5.5638942 | 5.9222056 | 6.4046757 | 5.7570693 |
| CAL851 | 5.9642354 | 5.3921799 | 5.935416 | 5.565036 | 5.8707598 | 6.2052979 | 6.0044 |
| CAL148 | 5.5098016 | 5.7984684 | 6.5015562 | 6.235573 | 5.0285972 | 5.2050786 | 5.8958117 |
| DU4475 | 4.866842 | 7.06508 | 6.2846185 | 6.2831084 | 5.2816933 | 5.2297969 | 6.248542 |
| ZR7530 | 5.9631313 | 6.107319 | 5.7425102 | 5.0854072 | 5.8362544 | 6.0317479 | 5.5259392 |
| CAL120 | 5.7301569 | 6.3763386 | 6.0813371 | 5.8336384 | 5.8733574 | 6.1453492 | 5.8338765 |
| EFM192A | 6.236391 | 6.4556125 | 6.085253 | 5.407432 | 5.8421372 | 5.8778209 | 5.6342148 |
| CAL51 | 6.2939763 | 6.3612783 | 6.1790109 | 6.1861659 | 5.8398152 | 5.8778718 | 5.8497182 |
| AU565 | 6.0767909 | 5.6058632 | 5.6726407 | 5.2443589 | 5.8289133 | 6.3488045 | 5.861401 |
| BT474 | 5.9345213 | 6.6904312 | 6.1753742 | 5.9287997 | 5.6868433 | 6.0238344 | 5.7844343 |
| ZR751 | 5.8085003 | 5.9547395 | 5.7113444 | 5.160258 | 5.9844945 | 5.7559191 | 6.0200071 |
| MCF7 | 5.9595591 | 6.0518599 | 6.2403322 | 5.5705707 | 5.8605426 | 5.892106 | 6.0613468 |
| JIMT1 | 5.9217784 | 5.0709295 | 5.6405078 | 5.9759896 | 6.2177076 | 6.0088828 | 6.2055994 |
| MDAMB231 | 6.0695958 | 5.5387649 | 5.7780071 | 5.4957832 | 5.9419258 | 5.926027 | 5.8509207 |
| MDAMB157 | 5.6983603 | 5.8195806 | 5.8453838 | 5.5780877 | 5.5445625 | 6.4248171 | 5.9437792 |
| MDAMB453 | 5.9320033 | 5.6962275 | 5.5512172 | 5.8391071 | 5.4620707 | 5.6553552 | 5.8438425 |
| UACC812 | 6.4424141 | 6.8520723 | 6.2389552 | 5.8899685 | 5.7122036 | 5.8589737 | 5.7678321 |
| HMC18 | 5.2128126 | 5.8963739 | 5.9225859 | 6.5760251 | 5.6449091 | 5.3704434 | 4.9959832 |
| Hs_578T | 6.0261923 | 6.4961771 | 6.027466 | 5.5872901 | 5.9864954 | 6.2779195 | 5.5027311 |
| HCC1395 | 5.8470559 | 6.3958346 | 5.6913257 | 6.2875485 | 5.6454829 | 5.762598 | 5.6004894 |

| **Cell line** | **NAD** | **NADP** | **oxalate** | **pantothenate** | **PEP** | **ribose-5-P/ ribulose5-P** | **sorbitol** |
| --- | --- | --- | --- | --- | --- | --- | --- |
| HDQP1 | 6.2190141 | 5.5465469 | 5.7146375 | 5.7054207 | 6.0483996 | 6.6487344 | 6.009386 |
| HCC1937 | 4.9272147 | 5.5770179 | 6.2812135 | 6.3007798 | 5.9255862 | 5.5359554 | 6.181923 |
| MDAMB361 | 5.5043372 | 5.411885 | 5.8909311 | 6.0269664 | 5.4160993 | 5.5192799 | 5.9017256 |
| HCC1143 | 5.1620107 | 5.575468 | 5.6164061 | 5.4044487 | 5.4198719 | 5.279944 | 6.0716769 |
| HCC1599 | 5.8151602 | 6.0148026 | 6.1703199 | 5.4134177 | 5.8074865 | 4.8974078 | 4.8879813 |
| HCC202 | 5.5860372 | 5.4646307 | 5.822611 | 5.6665216 | 5.6143698 | 5.6698143 | 5.842177 |
| BT20 | 6.2639146 | 6.2241914 | 5.9928446 | 6.2309401 | 5.7838065 | 5.7167525 | 6.2751045 |
| MDAMB468 | 5.6090101 | 6.0029182 | 6.028514 | 6.2230166 | 5.7679002 | 6.0541406 | 5.9466172 |
| HCC1569 | 5.8525285 | 5.4459671 | 6.0202714 | 5.1910803 | 6.655521 | 5.3790552 | 5.0625691 |
| HCC1954 | 6.2408824 | 6.0010189 | 5.88313 | 5.8170641 | 6.1910207 | 6.0492749 | 5.7986728 |
| HCC1806 | 6.1441235 | 6.5949641 | 5.7380345 | 5.991144 | 6.4027447 | 6.6644007 | 5.9218666 |
| MDAMB134VI | 5.2553599 | 5.671683 | 6.2467134 | 5.9768923 | 6.0930728 | 5.1385282 | 5.4152435 |
| HCC1419 | 5.8214415 | 6.5498741 | 5.864972 | 5.8839763 | 6.263271 | 5.9529234 | 5.7172738 |
| HCC1187 | 5.2865665 | 5.6450813 | 6.2727813 | 5.7024695 | 6.0464429 | 5.8156601 | 5.3518016 |
| T47D | 5.7517056 | 5.4772335 | 5.6602224 | 5.648359 | 6.1090164 | 6.2812962 | 5.7475484 |
| HCC38 | 5.5849228 | 5.67886 | 5.9124635 | 5.3157434 | 5.9466406 | 5.5994799 | 5.8218524 |
| BT483 | 5.558515 | 5.5200997 | 5.7156103 | 5.778148 | 6.134154 | 5.3421578 | 5.8954996 |
| HCC2218 | 5.741724 | 5.0734339 | 6.0702413 | 6.1607316 | 5.8228332 | 5.8183002 | 4.9251013 |
| MDAMB175VII | 5.6966622 | 6.4811889 | 5.5920892 | 5.4294827 | 6.2291327 | 5.9199372 | 5.8752049 |
| EFM19 | 5.2413497 | 5.3105511 | 5.9170596 | 5.8040415 | 5.5187271 | 5.6409849 | 5.9076766 |
| CAMA1 | 6.1162891 | 6.4841886 | 6.0251847 | 6.6527916 | 5.9639137 | 5.7270035 | 5.4744918 |
| HCC70 | 6.0061513 | 5.9715256 | 5.7925247 | 5.9728221 | 5.9535327 | 6.0353157 | 6.0336663 |
| UACC893 | 5.4571563 | 5.666712 | 5.9619759 | 5.4804975 | 5.906912 | 5.1303735 | 5.3423525 |
| MDAMB415 | 5.9345496 | 6.143499 | 5.6838263 | 5.4398742 | 6.2321805 | 6.1192867 | 5.9794764 |
| HCC1428 | 5.9689696 | 5.6479925 | 5.881848 | 5.9179946 | 6.3388922 | 6.1967577 | 5.929372 |
| BT549 | 5.5589543 | 5.2621182 | 5.8787447 | 5.5784922 | 5.7849084 | 5.9016934 | 6.1140133 |
| SKBR3 | 5.7246074 | 6.4049601 | 5.8413243 | 5.8031497 | 6.1822685 | 6.1858142 | 5.8731825 |
| CAL851 | 6.1215332 | 5.8694172 | 5.9124556 | 6.2605656 | 5.8539001 | 6.0413376 | 6.1925565 |
| CAL148 | 5.6408108 | 5.6458407 | 6.2068674 | 5.0676653 | 5.4517579 | 5.1199411 | 5.5099181 |
| DU4475 | 5.7331637 | 6.0722345 | 6.1371525 | 6.2287359 | 5.6859176 | 5.7520778 | 5.0934655 |
| ZR7530 | 5.7559399 | 5.9509615 | 5.8617152 | 6.0056704 | 5.9884735 | 5.8944233 | 5.8364513 |
| CAL120 | 5.9220794 | 6.0046752 | 5.7113932 | 5.929216 | 6.2115409 | 6.0369083 | 5.784812 |
| EFM192A | 5.7686211 | 6.2690918 | 5.8235391 | 5.921542 | 6.0661297 | 5.9097251 | 6.0557062 |
| CAL51 | 5.6695964 | 5.8076616 | 5.860977 | 6.3544078 | 5.8839763 | 5.737504 | 5.9392438 |
| AU565 | 5.8577556 | 5.8720102 | 5.9113378 | 6.2197733 | 6.1149279 | 5.7842812 | 5.8189882 |
| BT474 | 5.8839763 | 6.0108502 | 6.0487825 | 6.0431267 | 5.9782826 | 5.8725586 | 5.953167 |
| ZR751 | 5.997198 | 5.8671924 | 5.8668148 | 5.5759765 | 6.2539484 | 6.2886288 | 5.6202743 |
| MCF7 | 5.8867566 | 5.8160828 | 5.8444173 | 5.765612 | 6.0128132 | 5.8839763 | 5.8649225 |
| JIMT1 | 6.1926874 | 6.2017111 | 5.7657741 | 6.2679535 | 6.2688324 | 6.37897 | 5.8206251 |
| MDAMB231 | 6.044976 | 5.4493111 | 5.9535264 | 5.9311884 | 6.0371552 | 5.9103991 | 5.8882246 |
| MDAMB157 | 5.3807141 | 5.5232371 | 5.8631096 | 5.2956815 | 5.7360328 | 5.556896 | 5.8709798 |
| MDAMB453 | 5.5919651 | 5.9791748 | 5.8656838 | 6.2067678 | 6.3309176 | 6.078146 | 5.7393958 |
| UACC812 | 5.5504974 | 4.888097 | 5.6391896 | 5.7667491 | 5.544803 | 5.410925 | 6.024339 |
| HMC18 | 4.9593098 | 5.8417021 | 6.3552136 | 5.0150914 | 5.8129799 | 5.0650695 | 5.0085859 |
| Hs_578T | 5.6386465 | 5.5327821 | 5.990777 | 5.9435402 | 5.6752597 | 6.2281154 | 6.0325638 |
| HCC1395 | 6.0151006 | 6.1183726 | 6.0573902 | 6.0053589 | 5.794082 | 5.5713856 | 6.0931407 |

| **Cell line** | **succinate/ methylmalonate** | **sucrose** | **thymine** | **UMP** | **UDP-galactose/ UDP-glucose** | **uracil** | **urate** |
| --- | --- | --- | --- | --- | --- | --- | --- |
| HDQP1 | 5.8772164 | 6.1543097 | 6.2435612 | 5.9269918 | 6.0517566 | 6.0251231 | 5.9429854 |
| HCC1937 | 5.7616091 | 5.6035381 | 6.8851797 | 6.0735092 | 3.9531737 | 6.4179683 | 5.8463894 |
| MDAMB361 | 6.0226778 | 7.2568678 | 6.190811 | 5.4829937 | 5.2336639 | 6.0062652 | 6.1512913 |
| HCC1143 | 6.1715783 | 6.303451 | 6.6053688 | 5.5426239 | 5.5749593 | 6.3478712 | 6.1395001 |
| HCC1599 | 5.9728752 | 5.1493646 | 5.3792981 | 6.2727764 | 5.2005731 | 4.8052602 | 4.7429423 |
| HCC202 | 5.9312224 | 5.9025 | 5.9993024 | 5.8227556 | 5.3311154 | 5.9672858 | 4.8398546 |
| BT20 | 5.8080979 | 5.6995113 | 6.1421851 | 5.9001344 | 6.2872442 | 5.9331084 | 6.1846081 |
| MDAMB468 | 6.0146574 | 6.3808997 | 5.8700643 | 5.8301892 | 5.9409381 | 5.7198384 | 5.706454 |
| HCC1569 | 5.1515858 | 6.4756355 | 5.3282782 | 6.4207915 | 5.9364877 | 5.111684 | 4.882075 |
| HCC1954 | 5.7465598 | 6.4840011 | 5.9666317 | 5.7374205 | 6.0535143 | 5.8316907 | 6.0069211 |
| HCC1806 | 5.8737139 | 5.8321138 | 5.3297164 | 5.9039149 | 6.0976968 | 5.8184534 | 6.0909425 |
| MDAMB134VI | 5.4206125 | 5.625284 | 5.8700331 | 5.9001425 | 5.066402 | 5.5633251 | 5.5975513 |
| HCC1419 | 6.0676505 | 5.5149788 | 5.7639048 | 6.4600827 | 5.8139682 | 5.8191763 | 5.71862 |
| HCC1187 | 5.2230882 | 5.6446627 | 6.1383096 | 5.7193063 | 4.8253821 | 5.0365867 | 5.0636166 |
| T47D | 5.7721785 | 5.8437257 | 5.896336 | 6.1308502 | 5.868408 | 5.9537101 | 5.7832632 |
| HCC38 | 5.9804587 | 5.7504148 | 6.249992 | 5.7638525 | 5.9910985 | 5.9486149 | 5.7770237 |
| BT483 | 5.9431038 | 5.8839763 | 6.2672573 | 5.7739945 | 5.191247 | 6.1833738 | 6.4878025 |
| HCC2218 | 5.2317019 | 5.5364645 | 5.3128138 | 6.3801891 | 5.2809011 | 5.0849239 | 4.5815464 |
| MDAMB175VII | 5.9251959 | 5.876352 | 6.3137916 | 6.3258534 | 5.5629681 | 6.0481758 | 5.8432156 |
| EFM19 | 6.1662172 | 5.7998212 | 6.0880694 | 5.8705892 | 5.2792174 | 5.9396966 | 4.2742105 |
| CAMA1 | 5.7962723 | 5.8070727 | 5.9085886 | 5.9047431 | 5.8218047 | 5.6313678 | 5.62694 |
| HCC70 | 5.7618886 | 5.6697705 | 5.7791344 | 5.9337852 | 6.1315183 | 5.6630625 | 5.6720284 |
| UACC893 | 5.5313471 | 5.6531646 | 5.8903911 | 6.1111253 | 5.2600366 | 5.4128106 | 5.2449506 |
| MDAMB415 | 6.2646201 | 5.9046343 | 5.7349198 | 5.8724681 | 5.5238292 | 6.2892172 | 6.0720214 |
| HCC1428 | 5.949765 | 5.5010129 | 5.7458448 | 6.144121 | 5.7957954 | 5.8839763 | 5.8436081 |
| BT549 | 6.1062455 | 5.919205 | 6.2387894 | 5.5859166 | 5.3029879 | 6.0971436 | 6.3947399 |
| SKBR3 | 6.0125985 | 6.3358404 | 6.6409911 | 6.2880435 | 5.7271923 | 6.2699656 | 6.227791 |
| CAL851 | 5.7115254 | 6.1945407 | 5.8997179 | 5.7649797 | 6.0938438 | 5.8066094 | 5.3244156 |
| CAL148 | 5.788086 | 6.0096591 | 6.0600159 | 6.2493689 | 5.3122501 | 5.6617658 | 5.4114729 |
| DU4475 | 5.5067157 | 5.7444315 | 6.0541484 | 6.5139033 | 6.1000465 | 5.3498337 | 5.2712394 |
| ZR7530 | 6.3479802 | 6.1422645 | 5.9916169 | 6.0047105 | 5.6138299 | 5.9571711 | 5.9799754 |
| CAL120 | 5.8638242 | 6.1399745 | 5.9462369 | 5.8137835 | 6.144641 | 5.7664457 | 5.7256245 |
| EFM192A | 6.13362 | 5.6748401 | 5.8022971 | 5.8756846 | 5.4600529 | 6.1303457 | 6.1538379 |
| CAL51 | 6.1325502 | 6.033412 | 5.6163541 | 5.9042924 | 6.1175092 | 5.988727 | 6.0748077 |
| AU565 | 6.0629419 | 6.1586932 | 6.2298901 | 5.7096408 | 5.7244056 | 6.0457664 | 6.0255225 |
| BT474 | 6.0981708 | 6.0262361 | 5.5284721 | 5.717889 | 5.5798096 | 6.0126217 | 6.0666813 |
| ZR751 | 5.7934747 | 6.0111959 | 6.2559991 | 6.3368516 | 5.9046554 | 5.8852121 | 5.3616499 |
| MCF7 | 5.9151269 | 5.7648136 | 5.9586222 | 6.0831304 | 5.7969848 | 5.878978 | 6.0505919 |
| JIMT1 | 5.8035793 | 5.9622774 | 5.1815376 | 6.0667341 | 6.30849 | 5.2961742 | 6.2180167 |
| MDAMB231 | 5.889796 | 5.9196942 | 5.3988277 | 5.4039549 | 5.9355895 | 5.5793912 | 5.8976101 |
| MDAMB157 | 5.8861696 | 6.1091513 | 6.0251713 | 5.8700844 | 5.6320096 | 5.9149891 | 5.7608186 |
| MDAMB453 | 5.885125 | 5.6699004 | 5.8752349 | 6.1027447 | 5.7526679 | 5.8822753 | 5.3782949 |
| UACC812 | 6.0197719 | 5.9466013 | 6.4048301 | 5.7165341 | 5.1288856 | 6.1957318 | 6.2687075 |
| HMC18 | 5.2347193 | 5.6872764 | 6.3444435 | 5.8652248 | 4.6524594 | 5.0345116 | 4.5303534 |
| Hs_578T | 5.9057606 | 6.3151303 | 6.0123838 | 5.7937593 | 5.7268725 | 5.9026596 | 5.6357397 |
| HCC1395 | 5.7438572 | 5.8225715 | 6.091429 | 5.5379941 | 5.7685196 | 5.7549402 | 5.8330524 |

| **Cell line** | **uridine** | **xanthine** | **taurocholate** | **glycodeoxycholate/ glycochenodeoxycholate** | **taurodeoxycholate/ taurochenodeoxycholate** |
| --- | --- | --- | --- | --- | --- |
| HDQP1 | 6.1958063 | 5.9032537 | 6.1427134 | 5.8482206 | 6.1363971 |
| HCC1937 | 6.2809887 | 6.2045653 | 6.4038978 | 6.1576093 | 6.8495436 |
| MDAMB361 | 6.2596616 | 5.9902967 | 5.6287184 | 6.215178 | 6.1364928 |
| HCC1143 | 5.8162977 | 6.3585626 | 6.6202203 | 6.0491575 | 6.6139351 |
| HCC1599 | 5.9593811 | 4.9572677 | 5.3146192 | 5.5977047 | 5.383296 |
| HCC202 | 5.8705902 | 5.9790834 | 5.9322084 | 6.1470832 | 6.6338104 |
| BT20 | 5.3885681 | 5.8047377 | 5.7953893 | 5.9194592 | 3.5297187 |
| MDAMB468 | 5.4013095 | 5.6711984 | 5.8539536 | 5.8348137 | 5.9314262 |
| HCC1569 | 5.4513585 | 5.2162399 | 5.9003438 | 5.7704846 | 5.510848 |
| HCC1954 | 5.7212721 | 5.7151492 | 5.8490132 | 5.8313989 | 6.1935044 |
| HCC1806 | 5.5512051 | 5.9377809 | 6.1658285 | 6.2301563 | 6.4270263 |
| MDAMB134VI | 5.4681105 | 5.3734051 | 6.0743355 | 5.5863193 | 3.2005123 |
| HCC1419 | 6.3778697 | 5.7185013 | 5.9051885 | 5.6915152 | 6.0350503 |
| HCC1187 | 5.2159018 | 4.9767658 | 6.2915815 | 5.7056866 | 5.3695389 |
| T47D | 5.9128181 | 5.8680622 | 6.3463367 | 5.6765494 | 5.9341556 |
| HCC38 | 6.002019 | 5.9320286 | 6.0549592 | 6.0221281 | 5.9245241 |
| BT483 | 6.1504062 | 6.0187673 | 5.5932824 | 5.9868985 | 6.2638549 |
| HCC2218 | 5.4661811 | 4.7268005 | 6.0453558 | 5.442311 | 5.388571 |
| MDAMB175VII | 6.2196431 | 6.1095271 | 6.0857994 | 5.9991239 | 6.4936023 |
| EFM19 | 6.3173289 | 5.9446521 | 5.9109457 | 5.730509 | 6.0686982 |
| CAMA1 | 5.1741932 | 5.3724342 | 6.1082519 | 5.7940903 | 3.6581445 |
| HCC70 | 6.2132748 | 5.8030426 | 6.2569372 | 5.9434171 | 6.1543623 |
| UACC893 | 5.30642 | 5.0034398 | 6.1221569 | 5.5899643 | 2.9760308 |
| MDAMB415 | 6.5081285 | 6.1419606 | 5.9443422 | 6.2457075 | 6.4001303 |
| HCC1428 | 5.4349997 | 5.826503 | 6.1210065 | 5.9664023 | 5.9199659 |
| BT549 | 6.1719951 | 6.1254721 | 6.2853668 | 6.2340691 | 6.4247823 |
| SKBR3 | 6.1598447 | 6.3289288 | 6.2792943 | 6.6988883 | 4.3731 |
| CAL851 | 5.5417405 | 5.7363816 | 5.9127936 | 5.8231196 | 6.0673986 |
| CAL148 | 5.4959977 | 5.3302477 | 5.8966495 | 5.5986985 | 4.6461984 |
| DU4475 | 4.9377267 | 5.0826526 | 4.905645 | 5.6096731 | 4.0728015 |
| ZR7530 | 5.2612591 | 5.9454854 | 6.0143059 | 5.9511728 | 6.447765 |
| CAL120 | 5.8727456 | 5.740159 | 6.415096 | 5.5691826 | 5.7653235 |
| EFM192A | 4.889537 | 6.140656 | 5.9063118 | 6.0142462 | 6.1029148 |
| CAL51 | 5.9991528 | 6.0587168 | 5.8834841 | 6.0844884 | 6.2408068 |
| AU565 | 5.4403687 | 5.9362375 | 5.7814612 | 6.1470897 | 6.4351217 |
| BT474 | 5.695896 | 6.0436471 | 5.8096683 | 5.988762 | 6.1491971 |
| ZR751 | 5.6488404 | 5.6529188 | 5.8234399 | 5.7556306 | 6.1541728 |
| MCF7 | 6.6417185 | 5.9041055 | 5.7211068 | 5.8892192 | 3.658398 |
| JIMT1 | 5.2967575 | 4.7645113 | 6.0749478 | 5.7337049 | 6.1656107 |
| MDAMB231 | 5.7239215 | 5.7125671 | 6.0382141 | 5.9216302 | 6.292503 |
| MDAMB157 | 6.1219334 | 5.9079233 | 6.2824427 | 5.7763511 | 5.8700886 |
| MDAMB453 | 6.0406334 | 5.7814468 | 5.7764373 | 5.6885035 | 6.1078159 |
| UACC812 | 5.565046 | 6.2430219 | 5.9190539 | 6.2014311 | 6.3637353 |
| HMC18 | 5.4631726 | 4.6079593 | 6.2143337 | 5.5909463 | 5.408518 |
| Hs_578T | 5.4023737 | 5.8458304 | 6.1550753 | 5.882902 | 6.350972 |
| HCC1395 | 5.2187156 | 5.6814072 | 5.808082 | 5.9758701 | 6.1143167 |

| **Cell line** | **phosphocreatine** | **3-methyladipate/ pimelate** | **6-phosphogluconate** | **alpha-hydroxybutyrate** | **2-hydroxyglutarate** |
| --- | --- | --- | --- | --- | --- |
| HDQP1 | 5.3534654 | 6.0706307 | 6.0059553 | 5.8671424 | 5.7980074 |
| HCC1937 | 4.2138571 | 6.2578838 | 6.2531756 | 6.2790778 | 5.8414762 |
| MDAMB361 | 6.2303895 | 5.9809259 | 5.9831666 | 6.0242516 | 5.6225227 |
| HCC1143 | 5.8002067 | 5.8839763 | 4.8153457 | 6.0212146 | 5.78839 |
| HCC1599 | 5.7607466 | 5.9832567 | 6.2857229 | 6.2085655 | 7.1752309 |
| HCC202 | 5.8245146 | 5.8785199 | 6.6495552 | 5.8178442 | 5.4640949 |
| BT20 | 6.0580949 | 5.7916082 | 6.0542966 | 5.7840411 | 6.3127963 |
| MDAMB468 | 5.345307 | 5.9403694 | 6.163206 | 5.9888959 | 6.020321 |
| HCC1569 | 6.3057323 | 5.9074387 | 5.2137964 | 5.9725946 | 6.671675 |
| HCC1954 | 6.1706142 | 5.5804808 | 6.2791621 | 5.6823905 | 6.3660214 |
| HCC1806 | 6.5607532 | 5.8254957 | 5.9697286 | 5.601494 | 5.9533061 |
| MDAMB134VI | 5.2112112 | 6.1807683 | 6.1240266 | 6.260512 | 5.9633413 |
| HCC1419 | 6.1532308 | 5.6242778 | 6.5033345 | 5.5239962 | 6.0178172 |
| HCC1187 | 5.2652082 | 5.8491632 | 6.1997608 | 5.7794337 | 6.2382597 |
| T47D | 6.5409934 | 5.7577982 | 5.6094344 | 5.8613513 | 6.0625927 |
| HCC38 | 6.2369726 | 5.8532878 | 5.7481975 | 6.0044888 | 6.2066037 |
| BT483 | 6.0814792 | 5.6953392 | 6.2181308 | 5.8224407 | 5.5398252 |
| HCC2218 | 5.8708541 | 5.9353105 | 6.0368172 | 5.8816563 | 5.811304 |
| MDAMB175VII | 6.000231 | 5.820225 | 6.906791 | 5.5492837 | 5.5797088 |
| EFM19 | 5.8428277 | 5.9543428 | 5.86571 | 5.8071386 | 5.4906979 |
| CAMA1 | 6.0387222 | 5.8746021 | 5.3635222 | 5.7902905 | 5.523334 |
| HCC70 | 6.0663945 | 5.9347392 | 5.430624 | 5.6593198 | 6.3895865 |
| UACC893 | 5.370338 | 6.0734778 | 5.000535 | 6.0941984 | 5.6382632 |
| MDAMB415 | 6.4269344 | 5.6249262 | 5.6206825 | 5.8248526 | 5.7618194 |
| HCC1428 | 6.2378312 | 5.8896929 | 6.1201355 | 5.5361745 | 5.8005702 |
| BT549 | 6.1292639 | 6.1535166 | 5.6108563 | 5.7119005 | 5.6824698 |
| SKBR3 | 6.4134474 | 6.1121121 | 6.8004948 | 5.589908 | 5.9944636 |
| CAL851 | 6.2043282 | 5.9529599 | 6.0777381 | 5.9795671 | 5.6134246 |
| CAL148 | 5.6536847 | 6.0740789 | 5.5610873 | 6.1895227 | 5.5331479 |
| DU4475 | 6.699741 | 6.2547584 | 6.1481158 | 6.2039531 | 5.4759122 |
| ZR7530 | 5.815158 | 6.1755047 | 5.908981 | 5.7945964 | 6.0507211 |
| CAL120 | 6.3005334 | 5.678508 | 5.8059855 | 6.0279108 | 5.8513155 |
| EFM192A | 6.3003007 | 6.1465746 | 7.0144752 | 5.8153045 | 5.7068943 |
| CAL51 | 5.8428122 | 5.9099486 | 5.3421111 | 5.8517712 | 5.7167839 |
| AU565 | 6.084733 | 5.6250482 | 7.1632988 | 5.5698493 | 6.3397036 |
| BT474 | 6.3515911 | 6.0678367 | 5.6983057 | 5.8679088 | 5.5612695 |
| ZR751 | 6.8886571 | 5.6245738 | 6.4971365 | 5.7982666 | 5.4611047 |
| MCF7 | 6.2910712 | 5.8882801 | 6.1298769 | 5.8409227 | 5.4883922 |
| JIMT1 | 6.6003351 | 5.8892635 | 6.4747322 | 5.8987462 | 6.5779734 |
| MDAMB231 | 5.3184952 | 5.6398173 | 6.0697914 | 5.6683101 | 6.1017016 |
| MDAMB157 | 6.1521818 | 5.8839763 | 5.3110123 | 5.8706682 | 6.4385999 |
| MDAMB453 | 6.1981483 | 5.844504 | 6.5580511 | 5.92867 | 6.4019984 |
| UACC812 | 5.5464365 | 5.9241969 | 5.8385394 | 6.031499 | 5.5999206 |
| HMC18 | 4.5564464 | 6.3234332 | 6.6685418 | 6.4293915 | 5.6098831 |
| Hs_578T | 5.6921621 | 5.9710055 | 6.1217228 | 5.8719649 | 5.8697555 |
| HCC1395 | 5.316057 | 5.8408476 | 5.652081 | 6.1242042 | 5.7747938 |

| **Cell line** | **inositol** | **malondialdehyde** | **glycine** | **alanine** | **serine** | **threonine** | **methionine** | **aspartate** |
| --- | --- | --- | --- | --- | --- | --- | --- | --- |
| HDQP1 | 5.7924478 | 6.0901289 | 6.1850715 | 6.1101731 | 6.1141421 | 6.3232599 | 5.9991709 | 5.4879569 |
| HCC1937 | 5.5154354 | 6.4743673 | 5.5713589 | 6.2615604 | 6.1095758 | 5.755058 | 6.1257023 | 5.348522 |
| MDAMB361 | 5.7171854 | 5.5585502 | 5.715009 | 5.5763498 | 6.0858057 | 5.73262 | 5.6748211 | 5.7881116 |
| HCC1143 | 5.9954616 | 5.9761386 | 5.8825226 | 5.7830017 | 6.3391376 | 5.9708871 | 6.2059962 | 6.2415044 |
| HCC1599 | 5.4158798 | 5.7678733 | 5.8766745 | 5.416515 | 5.2290607 | 5.1783252 | 5.0008005 | 5.1748439 |
| HCC202 | 5.6989112 | 5.8839763 | 5.8088941 | 5.7453391 | 6.510347 | 5.9859032 | 6.0979377 | 6.0940609 |
| BT20 | 5.875699 | 6.1529448 | 5.5962146 | 6.3079267 | 6.1055546 | 6.3304469 | 5.8944341 | 5.953665 |
| MDAMB468 | 5.3088262 | 5.7862922 | 6.1904917 | 5.756895 | 6.1361148 | 6.2915878 | 6.0131285 | 5.0820502 |
| HCC1569 | 5.7602458 | 6.009902 | 5.7798727 | 5.4899264 | 5.1568303 | 5.1794895 | 5.0174726 | 5.1882538 |
| HCC1954 | 5.7322877 | 6.0328212 | 5.8589463 | 5.8074832 | 5.752376 | 5.5272983 | 5.6196302 | 5.6018438 |
| HCC1806 | 5.911405 | 6.2447332 | 5.709338 | 6.0102329 | 5.9973149 | 5.7709271 | 6.0952712 | 5.9754801 |
| MDAMB134VI | 5.208592 | 5.7887484 | 5.921576 | 5.88501 | 6.169783 | 6.1141925 | 5.7389211 | 5.4053623 |
| HCC1419 | 5.4865132 | 5.5694495 | 5.5063405 | 6.1200077 | 6.3755073 | 5.8658914 | 5.8604977 | 6.1286453 |
| HCC1187 | 5.29138 | 5.8419454 | 5.7389152 | 5.524164 | 5.8102071 | 5.4203907 | 5.0177455 | 5.0708994 |
| T47D | 5.6384803 | 5.8839763 | 5.8593876 | 5.6607533 | 6.0637918 | 5.6328576 | 5.7429027 | 5.8321468 |
| HCC38 | 5.728306 | 5.8222638 | 5.7629928 | 5.5627515 | 6.0741183 | 5.6760722 | 5.8337167 | 6.0404972 |
| BT483 | 5.6018404 | 5.7092398 | 5.8891326 | 5.7073445 | 6.1722439 | 5.7681619 | 5.7916417 | 5.8224116 |
| HCC2218 | 5.0704237 | 6.0340482 | 5.7732178 | 5.570843 | 5.8836015 | 5.6055042 | 5.2299649 | 5.727945 |
| MDAMB175VII | 5.7431327 | 5.8064083 | 5.703911 | 6.0719332 | 6.2940178 | 5.9481311 | 6.0695364 | 5.9168535 |
| EFM19 | 5.6680463 | 5.660615 | 5.8206069 | 5.3595556 | 6.1435257 | 5.9750684 | 5.8983751 | 5.8174435 |
| CAMA1 | 5.3201353 | 5.8241935 | 5.8096824 | 5.987556 | 5.5391569 | 6.1271302 | 5.6872081 | 5.8837809 |
| HCC70 | 5.9109358 | 5.8839763 | 6.4297656 | 6.1493616 | 6.1147741 | 5.826892 | 5.8751087 | 5.800643 |
| UACC893 | 5.8600994 | 5.868692 | 5.9352695 | 5.8836734 | 6.2389655 | 6.2002368 | 5.787714 | 5.4538743 |
| MDAMB415 | 5.8555635 | 5.812567 | 5.8487045 | 5.9383659 | 6.1833656 | 5.7683974 | 5.7934368 | 5.9876749 |
| HCC1428 | 5.7310412 | 6.0876399 | 5.6780229 | 5.6806268 | 5.8695242 | 5.752832 | 5.734006 | 5.5046847 |
| BT549 | 5.9184338 | 5.8850187 | 5.7541892 | 5.5598389 | 6.293933 | 6.0624095 | 6.0567632 | 5.9862179 |
| SKBR3 | 5.8035185 | 5.9409429 | 6.2714583 | 6.2300282 | 6.3335405 | 5.9426572 | 6.0967123 | 5.9913141 |
| CAL851 | 5.5610494 | 5.8680351 | 6.2246917 | 5.8167984 | 6.1216297 | 6.3044722 | 5.9815023 | 6.2500333 |
| CAL148 | 5.388701 | 5.9792816 | 5.4780217 | 5.4156105 | 5.8389249 | 5.8660796 | 5.4414958 | 6.0838373 |
| DU4475 | 5.8498794 | 5.953737 | 5.7496266 | 5.3409814 | 5.5605903 | 5.4011207 | 5.4307798 | 6.3722076 |
| ZR7530 | 5.6808695 | 5.8004664 | 5.8716304 | 5.6591005 | 6.2727644 | 5.8849767 | 5.9192557 | 5.9300936 |
| CAL120 | 5.9286127 | 5.9772992 | 6.2376823 | 5.8919431 | 6.1708485 | 6.3811557 | 6.1295737 | 5.7651122 |
| EFM192A | 5.6462633 | 5.7792706 | 5.8994927 | 5.6786514 | 6.1852778 | 5.8111381 | 5.8708183 | 5.7444352 |
| CAL51 | 5.9886795 | 5.9191648 | 5.9749055 | 5.7807021 | 6.2313069 | 6.3802408 | 6.1028718 | 5.811906 |
| AU565 | 5.8588593 | 5.8527965 | 5.836588 | 5.8640433 | 6.2148128 | 5.8173803 | 5.919311 | 5.8800518 |
| BT474 | 5.486468 | 5.863416 | 6.2040171 | 5.9200032 | 6.4376023 | 6.5317738 | 6.2091183 | 5.8689394 |
| ZR751 | 5.6556027 | 6.0587442 | 6.0556223 | 5.7463773 | 5.8427909 | 5.6092494 | 5.6393065 | 5.8567852 |
| MCF7 | 5.4916524 | 5.9877469 | 5.8143113 | 5.9631298 | 5.7971686 | 6.3508179 | 6.0508889 | 6.2535606 |
| JIMT1 | 5.9314865 | 6.2577701 | 6.1319527 | 6.3534907 | 5.6345448 | 6.3938054 | 6.2128458 | 5.7279834 |
| MDAMB231 | 5.8695036 | 6.0034765 | 5.6101497 | 5.1898112 | 5.8986529 | 5.6226777 | 5.7385233 | 5.8058832 |
| MDAMB157 | 5.7661504 | 5.7277603 | 5.7067444 | 5.4131576 | 6.0221445 | 5.6259251 | 5.7862753 | 6.1232229 |
| MDAMB453 | 5.4788135 | 5.5440706 | 5.998036 | 5.3515302 | 6.2197945 | 5.8576074 | 5.9357915 | 6.0640179 |
| UACC812 | 5.8375973 | 5.8912583 | 5.6049938 | 5.8673459 | 6.1036441 | 5.7620765 | 5.8831838 | 5.8821828 |
| HMC18 | 6.3107154 | 5.883215 | 5.5278718 | 4.4147474 | 5.0599778 | 4.7988552 | 4.4379505 | 5.6124605 |
| Hs_578T | 5.6564266 | 5.8867504 | 5.9583159 | 5.4634117 | 6.1951562 | 6.4265921 | 6.153095 | 5.2727725 |
| HCC1395 | 5.6652742 | 5.6355916 | 5.8665105 | 5.4896937 | 6.0793555 | 5.7498132 | 5.7849689 | 6.0388741 |

| **Cell line** | **glutamate** | **asparagine** | **glutamine** | **histidine** | **arginine** | **lysine** | **valine** | **leucine** |
| --- | --- | --- | --- | --- | --- | --- | --- | --- |
| HDQP1 | 5.6593082 | 5.2885277 | 6.1271318 | 5.9840493 | 5.5041611 | 6.2467319 | 6.2837015 | 5.9740465 |
| HCC1937 | 5.3207606 | 5.8416824 | 6.102046 | 5.7468592 | 6.2316149 | 5.6259674 | 5.9151324 | 5.8980724 |
| MDAMB361 | 5.3519974 | 5.8745236 | 5.7842788 | 6.0254687 | 5.9670297 | 5.8995636 | 5.8450474 | 5.7804124 |
| HCC1143 | 5.8507136 | 6.3931847 | 6.1754973 | 6.15794 | 6.3964115 | 6.3596763 | 6.0676276 | 6.2074151 |
| HCC1599 | 5.429716 | 5.5163668 | 5.0839499 | 4.9574307 | 5.1831726 | 5.1203009 | 5.2739578 | 4.9986011 |
| HCC202 | 5.5019405 | 6.3242953 | 6.1789605 | 6.0963141 | 6.2556134 | 6.1568249 | 6.0329212 | 6.0496614 |
| BT20 | 6.0909357 | 5.6815388 | 5.8968891 | 6.2689959 | 5.7921445 | 6.004784 | 6.2039415 | 5.9291182 |
| MDAMB468 | 5.6464768 | 4.576656 | 6.1309971 | 5.9890944 | 5.7128463 | 6.3076778 | 6.2722335 | 5.996508 |
| HCC1569 | 5.8139249 | 5.5403351 | 4.6646352 | 4.9246833 | 5.2652379 | 5.1042298 | 5.2200608 | 5.0029378 |
| HCC1954 | 5.6447451 | 5.9769308 | 5.8210825 | 5.6280098 | 5.903824 | 5.5657753 | 5.607882 | 5.6110926 |
| HCC1806 | 5.9267505 | 6.2956731 | 5.6239057 | 5.9640676 | 6.2224872 | 5.9026481 | 5.8930022 | 6.0137912 |
| MDAMB134VI | 4.8384412 | 5.9899145 | 5.1510166 | 6.2513157 | 5.6716521 | 5.3458041 | 5.6416731 | 5.4739427 |
| HCC1419 | 5.7493993 | 6.134415 | 5.9843097 | 5.7628822 | 5.9037881 | 5.6889804 | 5.7551489 | 5.700549 |
| HCC1187 | 5.2672284 | 5.277458 | 4.9830896 | 5.0259103 | 5.4008948 | 5.1592731 | 5.3300076 | 5.1539025 |
| T47D | 5.6887801 | 6.1210527 | 5.8913218 | 5.637716 | 6.0233965 | 5.599679 | 5.5330631 | 5.6929939 |
| HCC38 | 5.8316 | 6.092629 | 5.8045218 | 5.9696164 | 6.1257595 | 5.8839763 | 5.7023359 | 5.870622 |
| BT483 | 5.5344685 | 6.0474524 | 6.0108373 | 5.7566212 | 5.9756049 | 5.7659932 | 5.7505869 | 5.7184868 |
| HCC2218 | 5.494262 | 5.8154442 | 5.8839763 | 5.0645703 | 5.4259996 | 5.1074249 | 5.4195916 | 5.2337718 |
| MDAMB175VII | 5.4495131 | 6.2630019 | 6.1908648 | 6.0091211 | 6.1486286 | 6.1286328 | 5.9351537 | 5.9245712 |
| EFM19 | 5.3640152 | 6.0555133 | 6.1289033 | 6.044798 | 6.1437033 | 6.001802 | 5.9116835 | 5.9052523 |
| CAMA1 | 5.8066249 | 5.5382434 | 5.9900318 | 5.9033412 | 5.74837 | 5.8974356 | 6.0348252 | 5.6621931 |
| HCC70 | 5.5447498 | 6.1556708 | 5.8904506 | 5.868526 | 6.0955412 | 5.8391063 | 5.8085193 | 5.8563626 |
| UACC893 | 4.7845034 | 6.113371 | 5.3191834 | 6.350622 | 5.9832222 | 5.4745715 | 5.6496777 | 5.4814278 |
| MDAMB415 | 5.6684914 | 6.0890384 | 6.0257827 | 5.8323851 | 5.9514916 | 5.7880972 | 5.7182255 | 5.7046507 |
| HCC1428 | 5.534834 | 5.9396608 | 6.0105802 | 5.8009391 | 5.8637762 | 5.6855795 | 5.7029747 | 5.6892192 |
| BT549 | 5.766288 | 6.1781866 | 6.1889163 | 6.1188424 | 6.1053693 | 6.1848569 | 6.0464105 | 6.0501001 |
| SKBR3 | 5.5643397 | 6.2854082 | 5.9801993 | 6.0990464 | 5.8822834 | 6.1822351 | 6.0195682 | 5.9551579 |
| CAL851 | 6.0071782 | 4.9000348 | 6.0787519 | 5.7950143 | 5.6958446 | 6.193646 | 6.3114236 | 6.0021352 |
| CAL148 | 5.5694057 | 4.7141749 | 5.7448232 | 5.7047599 | 5.3963976 | 5.766797 | 5.7051594 | 5.5182761 |
| DU4475 | 6.0587122 | 5.8307376 | 5.5390934 | 5.5641841 | 5.5106176 | 4.9885762 | 5.4037316 | 5.4832705 |
| ZR7530 | 5.4849308 | 6.2604014 | 6.0667383 | 5.9606189 | 6.1735203 | 5.9053506 | 5.8500184 | 5.8910287 |
| CAL120 | 5.9880785 | 4.9728443 | 6.112035 | 5.9747867 | 5.7492361 | 6.4086888 | 6.2540597 | 6.0871714 |
| EFM192A | 5.2413395 | 6.0101675 | 6.0894481 | 5.8357896 | 5.9094879 | 5.9002483 | 5.8488974 | 5.8051607 |
| CAL51 | 5.8807475 | 4.7894225 | 6.1293616 | 5.9708436 | 5.5066516 | 6.3122356 | 6.3371308 | 6.0152476 |
| AU565 | 5.4613739 | 6.2321962 | 6.068695 | 5.9083221 | 6.2318342 | 5.9690102 | 5.8561997 | 5.9249249 |
| BT474 | 5.6466278 | 5.1647205 | 6.3248504 | 6.0504185 | 5.7554555 | 6.4938353 | 6.5147705 | 6.1888876 |
| ZR751 | 5.5453002 | 6.1264699 | 6.0414666 | 5.4874044 | 5.9413064 | 5.4390737 | 5.5148726 | 5.6552936 |
| MCF7 | 5.7670029 | 5.859017 | 6.2101545 | 6.2297015 | 6.1070558 | 6.2838009 | 6.3094652 | 5.9937056 |
| JIMT1 | 5.981456 | 4.6654415 | 5.8040944 | 5.9583411 | 5.6343975 | 6.3864077 | 6.4548849 | 6.1304942 |
| MDAMB231 | 5.8113017 | 6.0814739 | 5.9101064 | 5.9171237 | 6.1035964 | 5.7263248 | 5.672226 | 5.7448507 |
| MDAMB157 | 5.9212365 | 6.0637779 | 5.8980952 | 5.7916336 | 6.0583222 | 5.7752872 | 5.6432896 | 5.8240346 |
| MDAMB453 | 5.8898664 | 6.206278 | 6.2310834 | 6.0053353 | 6.1716228 | 5.9440813 | 5.8525971 | 5.9146401 |
| UACC812 | 5.5621974 | 5.9842086 | 5.9972852 | 5.8570602 | 5.8809366 | 5.9560893 | 5.8839763 | 5.8364226 |
| HMC18 | 5.1733975 | 5.0784204 | 4.8238593 | 4.72471 | 5.2524187 | 4.9872808 | 5.1434349 | 4.6406112 |
| Hs_578T | 5.9524271 | 4.6965446 | 6.1743803 | 6.1054087 | 5.8107316 | 6.475352 | 6.4222556 | 6.1566953 |
| HCC1395 | 5.9255012 | 6.0774207 | 5.8456908 | 5.9436694 | 6.1939102 | 5.7990961 | 5.8214198 | 5.8180594 |

| **Cell line** | **isoleucine** | **phenylalanine** | **tyrosine** | **tryptophan** | **proline** | **cis/trans-hydroxyproline** | **ornithine** |
| --- | --- | --- | --- | --- | --- | --- | --- |
| HDQP1 | 5.9773727 | 6.254395 | 6.1981833 | 6.2325618 | 5.4376652 | 5.1665375 | 5.6259746 |
| HCC1937 | 6.1912496 | 6.2218713 | 6.119966 | 6.5452726 | 6.0406352 | 6.0309809 | 5.7773344 |
| MDAMB361 | 5.6814049 | 5.7819535 | 5.763896 | 5.8285818 | 5.6041281 | 5.871747 | 6.1801113 |
| HCC1143 | 6.2017385 | 6.1366682 | 6.1146482 | 6.1290644 | 5.9468123 | 6.2007933 | 6.9281449 |
| HCC1599 | 4.9798782 | 4.9749945 | 5.1004145 | 5.1716075 | 5.7110854 | 5.9571784 | 4.9440655 |
| HCC202 | 6.0288258 | 6.0028863 | 6.0342595 | 5.946571 | 5.900567 | 6.1216769 | 6.683355 |
| BT20 | 5.9082653 | 6.1693553 | 6.1422654 | 6.2407495 | 5.7995992 | 5.5275375 | 5.8434175 |
| MDAMB468 | 6.0130386 | 6.2196988 | 6.1802584 | 6.0664117 | 5.2352749 | 4.9133346 | 5.4981502 |
| HCC1569 | 4.9766509 | 4.9854553 | 5.0475986 | 5.137444 | 5.366651 | 5.8839763 | 5.0061078 |
| HCC1954 | 5.6306809 | 5.5263354 | 5.5364864 | 5.4120265 | 5.7688994 | 6.0605896 | 5.8839763 |
| HCC1806 | 6.0365691 | 5.9483303 | 5.9818943 | 6.0203105 | 6.1430407 | 6.1760015 | 5.8834207 |
| MDAMB134VI | 5.7423931 | 5.8464272 | 6.1561923 | 5.7505188 | 5.2341566 | 4.775312 | 5.638797 |
| HCC1419 | 5.7220543 | 5.8217092 | 5.792929 | 5.8158249 | 5.7814224 | 6.0760971 | 5.8571777 |
| HCC1187 | 5.1341393 | 5.174298 | 5.1090008 | 5.3590118 | 5.4083587 | 5.6363761 | 5.7953152 |
| T47D | 5.715066 | 5.602184 | 5.6499555 | 5.68522 | 5.8852689 | 6.1093745 | 6.1244982 |
| HCC38 | 5.852983 | 5.7582878 | 5.8088683 | 5.8524598 | 5.8327971 | 6.0429417 | 6.3245354 |
| BT483 | 5.6900457 | 5.7303097 | 5.6940684 | 5.8948076 | 5.7519005 | 6.0572046 | 6.090004 |
| HCC2218 | 5.2250769 | 5.349153 | 5.2885082 | 5.5554529 | 5.7441332 | 6.150515 | 4.9877698 |
| MDAMB175VII | 5.9320673 | 5.9350432 | 5.9068514 | 5.9078188 | 5.8012448 | 6.1125512 | 6.6703989 |
| EFM19 | 5.90107 | 5.8699643 | 5.8169733 | 5.8794077 | 5.7761832 | 6.0233623 | 6.0735168 |
| CAMA1 | 5.6014764 | 5.9763383 | 5.9314015 | 6.1029904 | 5.683596 | 5.3335958 | 5.4561277 |
| HCC70 | 5.8883768 | 5.7608945 | 5.8396094 | 5.8697016 | 5.8424431 | 6.0271532 | 5.9543565 |
| UACC893 | 5.7743954 | 5.8305585 | 6.1069272 | 5.7134339 | 5.315411 | 4.3178964 | 5.5977446 |
| MDAMB415 | 5.6900747 | 5.7082686 | 5.6871645 | 5.6557495 | 5.7300776 | 6.1433441 | 6.4178767 |
| HCC1428 | 5.69892 | 5.7876383 | 5.7657301 | 5.7118308 | 5.8302257 | 5.9828738 | 6.3861021 |
| BT549 | 6.0567818 | 6.0454088 | 6.0157358 | 6.0300161 | 5.8030529 | 6.0604715 | 6.7114619 |
| SKBR3 | 5.8911677 | 6.122618 | 5.9775052 | 5.8433293 | 6.0249586 | 6.4913685 | 5.8776284 |
| CAL851 | 6.0131636 | 6.2387257 | 6.1915 | 6.0558856 | 5.4326269 | 5.019029 | 5.5226828 |
| CAL148 | 5.4969325 | 5.6503107 | 5.547469 | 5.7035701 | 5.2672848 | 5.0373548 | 5.587675 |
| DU4475 | 5.5002146 | 5.4048746 | 5.4942389 | 5.5732462 | 5.8107617 | 5.998431 | 5.5381429 |
| ZR7530 | 5.8771134 | 5.8273886 | 5.8132743 | 5.9415045 | 5.8610432 | 6.1333572 | 6.285376 |
| CAL120 | 6.0858198 | 6.3538055 | 6.2828557 | 6.2822259 | 5.8674388 | 5.0135879 | 5.6457229 |
| EFM192A | 5.8139003 | 5.8592547 | 5.7776821 | 5.7944597 | 5.7535993 | 5.9758231 | 6.4851984 |
| CAL51 | 6.0634374 | 6.2638617 | 6.2018477 | 6.1166837 | 5.6379673 | 5.2655574 | 5.7866537 |
| AU565 | 5.9167224 | 5.852985 | 5.8475388 | 5.8884165 | 5.8117662 | 6.0618574 | 6.1036516 |
| BT474 | 6.1562517 | 6.4297632 | 6.3161919 | 6.3339122 | 5.5242191 | 5.1441265 | 5.9709854 |
| ZR751 | 5.6585675 | 5.5164816 | 5.662435 | 5.4770604 | 5.9641093 | 6.1828899 | 5.9557799 |
| MCF7 | 6.0150203 | 6.2804053 | 6.2818236 | 6.2059312 | 5.9125468 | 5.5029117 | 5.8328982 |
| JIMT1 | 6.1313923 | 6.4477251 | 6.3706439 | 6.3450487 | 6.0129938 | 4.8812439 | 5.5988589 |
| MDAMB231 | 5.7451719 | 5.6223977 | 5.6338626 | 5.6620907 | 5.8912153 | 6.0200425 | 6.0621962 |
| MDAMB157 | 5.8129451 | 5.7269013 | 5.7182081 | 5.745143 | 5.6876607 | 6.0253564 | 6.2199481 |
| MDAMB453 | 5.9172961 | 5.8430505 | 5.886691 | 5.8641576 | 5.6703667 | 6.1268521 | 5.8988572 |
| UACC812 | 5.8023482 | 5.8378269 | 5.7910771 | 5.9410285 | 5.7196021 | 5.9163452 | 6.5575896 |
| HMC18 | 4.7156586 | 4.6709562 | 4.7926819 | 4.8419851 | 4.8465982 | 5.408486 | 5.1298445 |
| Hs_578T | 6.1499092 | 6.3838085 | 6.2831825 | 6.318126 | 5.3559596 | 5.0069253 | 5.80401 |
| HCC1395 | 5.8467812 | 5.7257886 | 5.7829379 | 5.6845092 | 5.8143487 | 6.1454982 | 6.2053275 |

| **Cell line** | **citrulline** | **taurine** | **5-HIAA** | **serotonin** | **GABA** | **acetylglycine** | **dimethylglycine** |
| --- | --- | --- | --- | --- | --- | --- | --- |
| HDQP1 | 5.8841353 | 6.010648 | 5.5881883 | 6.5614637 | 6.5279314 | 5.6247386 | 5.9232885 |
| HCC1937 | 5.7764374 | 5.1099669 | 6.258659 | 6.149373 | 6.4548638 | 6.3033212 | 5.9798529 |
| MDAMB361 | 5.8632342 | 5.6956897 | 5.5801988 | 6.068505 | 4.7966708 | 5.7538438 | 5.8477224 |
| HCC1143 | 6.1012341 | 5.8119318 | 5.8755462 | 5.6813688 | 6.1540495 | 5.9541147 | 6.1066466 |
| HCC1599 | 5.1368812 | 5.2080135 | 6.1635097 | 6.119353 | 6.3376651 | 6.1596339 | 5.1216245 |
| HCC202 | 6.235661 | 5.2453635 | 5.7622186 | 5.7285511 | 5.8579516 | 5.8126514 | 6.0524584 |
| BT20 | 5.5826745 | 6.3279168 | 6.0838494 | 6.4701271 | 5.2062295 | 5.5839066 | 5.7410807 |
| MDAMB468 | 5.6710094 | 4.875111 | 5.8165207 | 5.5830829 | 5.7281904 | 5.8532373 | 5.7172191 |
| HCC1569 | 4.6270259 | 5.013439 | 6.2190844 | 5.0524417 | 5.9965389 | 5.274247 | 5.10734 |
| HCC1954 | 5.7225872 | 6.0472589 | 5.7019466 | 5.7469565 | 5.5973403 | 5.8369937 | 5.7771297 |
| HCC1806 | 6.0663702 | 5.8976661 | 6.0377498 | 6.0494044 | 6.2684344 | 5.7919402 | 6.0236085 |
| MDAMB134VI | 5.3941938 | 5.8581968 | 6.2230059 | 6.1947539 | 4.6872017 | 5.709578 | 5.8523984 |
| HCC1419 | 6.0001274 | 5.2134486 | 5.5978796 | 5.8947083 | 5.4883549 | 6.1319795 | 5.6835929 |
| HCC1187 | 4.9045882 | 5.3578722 | 6.1225869 | 4.7023403 | 7.3142095 | 5.9220085 | 5.275015 |
| T47D | 5.6404435 | 6.3546883 | 5.5828074 | 5.8889484 | 5.3997379 | 6.183861 | 5.7632015 |
| HCC38 | 6.0457061 | 6.2592463 | 5.9834552 | 5.8802008 | 6.5030923 | 5.7480344 | 5.8823589 |
| BT483 | 6.0024165 | 5.7828435 | 6.3030768 | 6.7024348 | 4.9412984 | 6.3060161 | 6.0485456 |
| HCC2218 | 5.3211317 | 5.5778684 | 5.9587873 | 6.1882192 | 5.8975131 | 6.1238978 | 5.1067321 |
| MDAMB175VII | 6.1001196 | 5.8692336 | 5.6792972 | 5.6171291 | 4.9524119 | 6.1826572 | 5.9757691 |
| EFM19 | 6.0489964 | 5.8839763 | 5.7013119 | 5.9414049 | 4.618966 | 5.7554575 | 6.0198573 |
| CAMA1 | 5.3798054 | 6.2552052 | 6.0883586 | 6.3765464 | 5.0757951 | 5.8839763 | 5.6537281 |
| HCC70 | 5.667355 | 5.5712437 | 5.6686025 | 4.961982 | 5.2057004 | 5.9703848 | 6.0015281 |
| UACC893 | 5.222257 | 5.6058825 | 6.2746348 | 6.1285628 | 5.7445416 | 5.9530297 | 5.2038341 |
| MDAMB415 | 6.1556172 | 6.399903 | 5.8762306 | 5.552258 | 5.6414746 | 5.962437 | 6.0065947 |
| HCC1428 | 5.4493485 | 6.3441647 | 5.5165928 | 5.2754296 | 5.4062135 | 5.9140652 | 5.7097272 |
| BT549 | 5.9593661 | 5.4918613 | 5.9441253 | 6.6286388 | 6.0364801 | 5.7143683 | 6.0034057 |
| SKBR3 | 5.9677395 | 5.1824458 | 5.8536064 | 6.3878924 | 5.2817933 | 5.8132535 | 6.0735134 |
| CAL851 | 5.7271157 | 5.2839341 | 5.4764829 | 5.8031779 | 6.1504082 | 5.9852693 | 5.7014663 |
| CAL148 | 5.5359349 | 5.1873449 | 6.3029558 | 5.650904 | 5.890774 | 5.7867116 | 5.4926861 |
| DU4475 | 5.4160351 | 6.5292376 | 6.1531254 | 5.4534993 | 7.5698276 | 6.2362826 | 5.5149652 |
| ZR7530 | 5.8810788 | 5.466832 | 5.5150177 | 5.6540038 | 5.0604738 | 5.8471105 | 5.8837206 |
| CAL120 | 5.797246 | 5.890963 | 5.9809678 | 5.7777414 | 5.2643491 | 5.5889713 | 5.8613963 |
| EFM192A | 6.0391256 | 5.6902546 | 5.9119214 | 6.0576814 | 5.8547426 | 5.8833928 | 6.120305 |
| CAL51 | 6.0321952 | 5.2838674 | 5.8793912 | 6.1789738 | 5.3546361 | 5.8573145 | 5.9445953 |
| AU565 | 5.9247123 | 5.4441679 | 5.7565377 | 6.4146639 | 5.9284938 | 5.6917804 | 5.8839763 |
| BT474 | 6.0679464 | 5.5680457 | 5.8479466 | 6.3124476 | 5.7478998 | 6.5811865 | 6.0504026 |
| ZR751 | 5.4834249 | 5.9483948 | 5.8700932 | 5.8401269 | 5.1816848 | 6.5000859 | 5.6620466 |
| MCF7 | 5.9356024 | 6.2080688 | 6.0763792 | 6.4744301 | 5.7068146 | 6.2420192 | 5.8965551 |
| JIMT1 | 5.5400462 | 6.0766851 | 6.0908759 | 4.6716287 | 5.2400094 | 5.9502784 | 5.8292664 |
| MDAMB231 | 5.9031852 | 5.953439 | 5.6775835 | 6.1734941 | 6.106124 | 5.7022645 | 5.7819347 |
| MDAMB157 | 5.7801252 | 5.6493863 | 5.766509 | 5.7939347 | 6.353417 | 5.437211 | 5.8037225 |
| MDAMB453 | 5.8084232 | 5.6753008 | 5.7909199 | 4.4793148 | 5.3547871 | 5.9233368 | 5.8831799 |
| UACC812 | 6.1331516 | 5.4234543 | 5.7035549 | 6.1290085 | 4.5542119 | 5.9208393 | 6.190904 |
| HMC18 | 5.2116678 | 4.4946484 | 6.5119435 | 6.210332 | 5.6859345 | 6.3914329 | 5.1332267 |
| Hs_578T | 5.8765202 | 5.2207009 | 5.9866881 | 4.9249323 | 5.7030389 | 5.6094971 | 5.8042853 |
| HCC1395 | 5.8221421 | 5.9634969 | 6.1377741 | 6.1281803 | 5.8151255 | 6.1424438 | 5.8084805 |

| **Cell line** | **homocysteine** | **SDMA/ADMA** | **NMMA** | **allantoin** | **anthranilic acid** | **kynurenic acid** |
| --- | --- | --- | --- | --- | --- | --- |
| HDQP1 | 6.0193984 | 5.8974612 | 6.0393723 | 5.9120529 | 5.7396747 | 5.8344056 |
| HCC1937 | 6.1426434 | 6.01931 | 5.8014196 | 6.4867552 | 6.2571904 | 5.572536 |
| MDAMB361 | 5.5488572 | 5.9233437 | 5.9084244 | 5.9188762 | 6.2425254 | 5.7887954 |
| HCC1143 | 5.1595771 | 6.2191347 | 6.3063137 | 6.2516834 | 6.4490839 | 6.7148659 |
| HCC1599 | 5.9616071 | 5.2702637 | 5.1889146 | 5.2252098 | 5.8839763 | 4.4591688 |
| HCC202 | 4.9337606 | 6.096308 | 6.3048738 | 6.1825801 | 6.4218032 | 5.9256759 |
| BT20 | 6.0286669 | 5.8977944 | 5.962575 | 5.7964669 | 5.7408198 | 5.6807826 |
| MDAMB468 | 5.5190687 | 5.6981012 | 5.7801386 | 5.8380756 | 5.8061561 | 5.8689174 |
| HCC1569 | 6.0243763 | 5.2440299 | 5.2716744 | 5.2198616 | 5.7405433 | 4.4369207 |
| HCC1954 | 6.1707045 | 5.8062119 | 5.5513247 | 5.8148022 | 6.5296197 | 5.5983379 |
| HCC1806 | 6.5941124 | 5.9845844 | 6.0639292 | 5.978268 | 6.6278283 | 6.064163 |
| MDAMB134VI | 5.8988504 | 5.3321426 | 4.9505581 | 5.2415122 | 5.6323712 | 5.6658655 |
| HCC1419 | 6.347437 | 5.7696933 | 5.9597805 | 5.6574468 | 5.4407036 | 5.7230209 |
| HCC1187 | 6.0292384 | 5.2129058 | 5.1323167 | 5.1612496 | 5.5811571 | 4.6642 |
| T47D | 5.6282674 | 5.7319038 | 5.5755274 | 5.7972089 | 6.0695013 | 5.8776914 |
| HCC38 | 5.4159701 | 5.963824 | 5.9066622 | 5.972953 | 6.566457 | 6.2168026 |
| BT483 | 5.5725854 | 6.0523578 | 6.0622591 | 5.9830165 | 6.0215635 | 5.8634306 |
| HCC2218 | 5.9275114 | 5.4164482 | 5.4268555 | 5.1703886 | 5.9375475 | 4.3513624 |
| MDAMB175VII | 5.375434 | 6.1046525 | 6.357804 | 6.1045472 | 6.0304565 | 5.8839763 |
| EFM19 | 5.9406135 | 5.8723926 | 5.8340108 | 6.0536941 | 6.6515805 | 6.1564477 |
| CAMA1 | 6.3639558 | 5.685936 | 5.318653 | 5.5261751 | 5.8590795 | 4.5027396 |
| HCC70 | 6.0910032 | 5.9927313 | 6.1137544 | 5.9759068 | 6.1524451 | 6.0921926 |
| UACC893 | 5.8547672 | 5.273679 | 5.306284 | 5.0289609 | 5.4991172 | 5.2979193 |
| MDAMB415 | 5.5421778 | 6.0393148 | 6.1145141 | 5.9966359 | 5.9408892 | 5.7792962 |
| HCC1428 | 5.9283271 | 5.8135827 | 5.8176632 | 5.7228528 | 5.7264003 | 5.5186429 |
| BT549 | 5.8634468 | 6.0668227 | 5.8962352 | 5.9721956 | 6.7630215 | 6.0281371 |
| SKBR3 | 5.4142785 | 6.0837259 | 6.1193382 | 6.064768 | 6.1937296 | 6.1130126 |
| CAL851 | 5.9243556 | 5.7235306 | 5.6695866 | 5.8455885 | 5.8848931 | 5.3063941 |
| CAL148 | 6.2334988 | 5.4998926 | 5.4124001 | 5.3809478 | 5.9160863 | 5.6335352 |
| DU4475 | 6.1091941 | 5.301607 | 5.2857919 | 5.0977641 | 5.6569153 | 4.7561093 |
| ZR7530 | 5.5932401 | 5.8809521 | 5.8324965 | 5.9238544 | 5.8152783 | 6.0329769 |
| CAL120 | 6.0928694 | 5.7927849 | 5.9582158 | 5.7749656 | 5.6114826 | 6.1901493 |
| EFM192A | 5.7761864 | 6.0437503 | 6.0596571 | 6.0659516 | 5.8603695 | 6.2338986 |
| CAL51 | 5.6822954 | 5.8687647 | 5.9318221 | 6.0362514 | 5.84053 | 6.0005062 |
| AU565 | 5.7141949 | 5.9913597 | 5.7780352 | 5.9305941 | 6.2771136 | 5.9911394 |
| BT474 | 5.746111 | 5.9186694 | 5.950764 | 6.078442 | 5.7575874 | 6.1040297 |
| ZR751 | 5.9022979 | 5.8407816 | 5.8481442 | 5.7620958 | 5.8623009 | 5.467223 |
| MCF7 | 6.1167784 | 6.1514655 | 6.2402497 | 5.8863499 | 6.8562555 | 5.8114851 |
| JIMT1 | 6.4910114 | 5.863668 | 6.0855279 | 5.902109 | 5.5792918 | 5.8448099 |
| MDAMB231 | 5.8158838 | 5.8413407 | 5.6515367 | 5.7944534 | 6.1285142 | 5.5971721 |
| MDAMB157 | 5.3577375 | 5.7820489 | 5.6293385 | 5.9057659 | 6.1672964 | 6.1772019 |
| MDAMB453 | 6.1486431 | 5.7930145 | 5.8253181 | 5.9486372 | 6.108545 | 5.8101526 |
| UACC812 | 4.9343112 | 6.1425243 | 6.2068508 | 6.2794792 | 6.3415491 | 6.0956675 |
| HMC18 | 6.2725481 | 4.8117526 | 5.102016 | 5.0761711 | 6.2216309 | 4.5801516 |
| Hs_578T | 5.8160611 | 5.7935356 | 5.9741002 | 5.8848637 | 5.6549925 | 5.6057249 |
| HCC1395 | 5.5382583 | 5.857286 | 5.6575849 | 5.8277172 | 6.5680019 | 5.4667341 |

| **Cell line** | **5-adenosyl homocysteine** | **carnosine** | **N-carbamoyl-beta-alanine** | **thiamine** | **niacinamide** | **betaine** |
| --- | --- | --- | --- | --- | --- | --- |
| HDQP1 | 5.7638327 | 6.1471323 | 5.8020844 | 6.1765894 | 6.2094685 | 5.837936 |
| HCC1937 | 5.925404 | 5.7663379 | 7.1693195 | 6.2362185 | 6.6557655 | 6.1456804 |
| MDAMB361 | 5.5344945 | 5.9960169 | 5.4502138 | 5.5047255 | 5.6156095 | 5.8362912 |
| HCC1143 | 5.4609626 | 6.2809816 | 6.2576978 | 6.1969792 | 6.2975854 | 6.0411486 |
| HCC1599 | 5.9208194 | 5.7649982 | 5.5997907 | 5.1301736 | 5.3230238 | 5.513473 |
| HCC202 | 5.4421533 | 6.1367732 | 5.5652759 | 6.007244 | 6.1108162 | 5.9206031 |
| BT20 | 6.3834254 | 6.1856035 | 5.807483 | 5.8356263 | 5.6050329 | 5.7868153 |
| MDAMB468 | 5.3520908 | 5.8951074 | 5.7445491 | 6.2531406 | 6.3533495 | 5.7436268 |
| HCC1569 | 5.7357217 | 5.1723598 | 5.7519785 | 5.2342931 | 5.4902822 | 5.3746321 |
| HCC1954 | 5.9894912 | 5.7556104 | 5.888571 | 5.7619761 | 5.6346364 | 5.7621772 |
| HCC1806 | 5.9649429 | 6.0455523 | 6.5696218 | 5.9935423 | 6.0612661 | 5.8846734 |
| MDAMB134VI | 5.9359734 | 5.3537237 | 5.7521697 | 4.7093885 | 4.7960664 | 5.8605157 |
| HCC1419 | 6.2064744 | 5.7670704 | 4.5567845 | 5.8320897 | 5.8077243 | 5.6217641 |
| HCC1187 | 5.6411521 | 5.5682745 | 5.2070601 | 5.0483238 | 5.1868581 | 5.8466878 |
| T47D | 5.6520052 | 5.7453087 | 5.1731496 | 5.6492037 | 5.7755637 | 5.8363369 |
| HCC38 | 5.6531983 | 6.3079211 | 5.3609002 | 5.8939469 | 5.915163 | 5.980215 |
| BT483 | 6.0971525 | 6.0389547 | 5.1750481 | 5.6504713 | 5.7658404 | 6.0180206 |
| HCC2218 | 6.2032459 | 4.7286184 | 5.560178 | 5.5237242 | 5.3441186 | 6.0119909 |
| MDAMB175VII | 5.6681868 | 6.1241885 | 5.8789813 | 5.92118 | 6.1208055 | 5.9969586 |
| EFM19 | 5.3939692 | 6.0857267 | 5.5244577 | 5.8611741 | 6.0689012 | 6.0999531 |
| CAMA1 | 6.2900874 | 5.3978966 | 5.7122406 | 5.5112782 | 5.5610876 | 6.1544685 |
| HCC70 | 5.9542391 | 6.3380716 | 6.9232587 | 5.994654 | 5.9185292 | 5.8990152 |
| UACC893 | 5.5897729 | 5.4858167 | 5.8699964 | 4.9877075 | 4.9327518 | 5.6898632 |
| MDAMB415 | 5.7213942 | 6.1866786 | 6.7516636 | 5.795349 | 5.8839763 | 6.2163118 |
| HCC1428 | 5.916426 | 6.4899433 | 5.5267539 | 5.9627049 | 5.7472958 | 6.0379416 |
| BT549 | 5.67841 | 6.2546997 | 6.0636421 | 6.0841981 | 6.0839875 | 5.9690791 |
| SKBR3 | 5.6746364 | 6.1304083 | 5.7329492 | 5.3551371 | 5.9747984 | 5.8380246 |
| CAL851 | 5.758959 | 5.9088731 | 6.1240164 | 6.2286425 | 6.2823904 | 5.8627283 |
| CAL148 | 5.8609582 | 6.193861 | 5.378062 | 5.7955429 | 5.5830144 | 6.3843516 |
| DU4475 | 6.8340407 | 6.4872755 | 5.4818825 | 5.5300391 | 4.9971092 | 6.0792849 |
| ZR7530 | 5.3852412 | 6.0023355 | 5.3017303 | 5.8166577 | 5.9639229 | 5.8555611 |
| CAL120 | 5.6891056 | 5.7532399 | 6.3928404 | 6.35261 | 6.3259694 | 5.8628447 |
| EFM192A | 5.8663365 | 6.2476245 | 5.048738 | 5.8693712 | 6.0066771 | 5.8923144 |
| CAL51 | 5.8224439 | 5.9998926 | 6.5449773 | 6.1925334 | 6.3688682 | 5.9185015 |
| AU565 | 5.8268708 | 6.098129 | 5.3743658 | 5.9531364 | 6.0006167 | 5.81962 |
| BT474 | 5.8468509 | 6.1700387 | 5.0560153 | 6.3518684 | 6.5518277 | 6.2766561 |
| ZR751 | 5.718425 | 5.8230345 | 5.4692822 | 5.8856921 | 5.7165886 | 5.7752221 |
| MCF7 | 6.3343216 | 5.8393233 | 5.8167516 | 5.9266852 | 5.9368089 | 5.8883739 |
| JIMT1 | 6.0915622 | 6.0322654 | 6.9030628 | 6.3030995 | 6.5923228 | 5.7680955 |
| MDAMB231 | 5.7275765 | 5.8415386 | 6.8010154 | 5.7988881 | 5.6955732 | 5.8361866 |
| MDAMB157 | 5.6870054 | 5.8578165 | 5.7622713 | 5.6701575 | 5.6382598 | 5.885934 |
| MDAMB453 | 5.7249942 | 5.8509933 | 5.4317255 | 6.0138398 | 6.0080826 | 5.8219735 |
| UACC812 | 5.5969267 | 6.3268087 | 5.2030645 | 5.6508915 | 5.9224539 | 6.1940915 |
| HMC18 | 4.9570672 | 4.5852217 | 6.02956 | 4.5986412 | 5.1526402 | 5.0947913 |
| Hs_578T | 5.6853539 | 5.8695111 | 5.9833059 | 6.346702 | 6.424918 | 5.8134636 |
| HCC1395 | 5.8475208 | 5.8698289 | 6.1879802 | 5.7211388 | 5.8012572 | 5.9032325 |

| **Cell line** | **choline** | **alpha-glycero phosphocholine** | **acetylcholine** | **creatine** | **creatinine** | **thyroxine** |
| --- | --- | --- | --- | --- | --- | --- |
| HDQP1 | 5.9503104 | 6.5312604 | 5.3108451 | 5.1168319 | 5.8283883 | 5.9903068 |
| HCC1937 | 6.2833748 | 6.1592807 | 5.1481616 | 5.6535779 | 6.0578639 | 6.2125344 |
| MDAMB361 | 5.8534715 | 6.0160083 | 4.2801331 | 5.6293535 | 5.9655199 | 6.1404705 |
| HCC1143 | 6.1684654 | 6.0381758 | 4.157951 | 5.8026443 | 6.1876793 | 6.2974235 |
| HCC1599 | 5.1515409 | 6.0601001 | 5.195008 | 5.6212818 | 5.1973937 | 5.2861433 |
| HCC202 | 6.0406183 | 6.2409106 | 4.1950635 | 5.7764007 | 6.0021013 | 6.0770258 |
| BT20 | 5.8906842 | 5.5089855 | 6.3243662 | 5.4613272 | 5.781174 | 5.8817231 |
| MDAMB468 | 5.8787565 | 5.7339898 | 5.4679032 | 5.149785 | 5.6625638 | 5.8678782 |
| HCC1569 | 5.7790314 | 5.1607365 | 4.6229823 | 5.9204617 | 5.6098171 | 5.3724988 |
| HCC1954 | 5.7042675 | 4.8601646 | 3.797354 | 5.8130202 | 5.9068989 | 5.8137816 |
| HCC1806 | 5.896559 | 6.1794307 | 5.8474431 | 6.1435326 | 6.0562108 | 6.2987168 |
| MDAMB134VI | 5.727629 | 6.1372744 | 4.5104316 | 4.9315059 | 5.3066682 | 5.5740681 |
| HCC1419 | 5.8376926 | 5.2865026 | 6.1691857 | 5.8064372 | 5.7643469 | 5.7967187 |
| HCC1187 | 5.6014193 | 6.0276639 | 6.1607949 | 5.9642693 | 5.2656254 | 5.2599288 |
| T47D | 5.5265444 | 6.3037408 | 4.375991 | 6.1439477 | 5.9986875 | 5.863571 |
| HCC38 | 6.0285778 | 5.6689144 | 3.9349412 | 6.3410812 | 5.9259601 | 5.9174617 |
| BT483 | 6.0315439 | 6.1611533 | 5.6770986 | 5.7924442 | 5.9961609 | 6.1678972 |
| HCC2218 | 5.7872662 | 4.6650346 | 6.3279735 | 6.0356489 | 5.199038 | 5.5467342 |
| MDAMB175VII | 6.016423 | 5.0340868 | 5.0203463 | 5.8251264 | 5.9551175 | 5.9465065 |
| EFM19 | 6.1107326 | 5.4020944 | 6.5207779 | 5.9043433 | 5.870225 | 6.0619101 |
| CAMA1 | 5.5756308 | 6.4604215 | 5.1321719 | 5.7863214 | 5.491043 | 5.3753956 |
| HCC70 | 5.9117719 | 6.3772485 | 6.0844492 | 5.7848391 | 5.9047281 | 6.1268615 |
| UACC893 | 5.8603159 | 6.4630629 | 4.614846 | 5.4925613 | 5.1853933 | 5.6370159 |
| MDAMB415 | 5.8998735 | 6.2935404 | 5.6579608 | 5.7741664 | 6.1047212 | 6.0871432 |
| HCC1428 | 5.7695975 | 7.0231659 | 6.8799994 | 5.9411738 | 5.7454443 | 5.8273357 |
| BT549 | 6.113637 | 6.1190781 | 6.2702654 | 5.8506255 | 5.9891767 | 5.9045327 |
| SKBR3 | 6.1624523 | 5.3401038 | 4.8127582 | 5.7456066 | 6.0347534 | 6.1431601 |
| CAL851 | 5.9918726 | 6.1999785 | 5.755602 | 5.7381245 | 5.9125278 | 5.6946806 |
| CAL148 | 6.1983544 | 6.1112157 | 4.820717 | 5.5516078 | 5.5308706 | 5.5870298 |
| DU4475 | 5.8486921 | 4.9895362 | 4.7213871 | 6.5531559 | 5.8317143 | 5.7668892 |
| ZR7530 | 5.9477351 | 5.2382969 | 5.1750155 | 5.5072854 | 5.7718041 | 6.0525889 |
| CAL120 | 5.8476419 | 6.7523928 | 6.9969689 | 6.1552263 | 5.9643724 | 5.667012 |
| EFM192A | 5.9154378 | 6.0572907 | 6.0354476 | 5.847956 | 6.1033962 | 6.177286 |
| CAL51 | 6.0442339 | 5.5925095 | 6.2763708 | 5.6305639 | 5.9019104 | 6.1470488 |
| AU565 | 6.0592389 | 5.0255717 | 6.5047752 | 5.7502719 | 5.8467116 | 5.9717442 |
| BT474 | 6.0770853 | 5.8131198 | 5.5299124 | 5.9585089 | 6.0725019 | 5.7477111 |
| ZR751 | 5.3459376 | 6.7082271 | 4.2189217 | 6.203934 | 5.9367732 | 5.8784057 |
| MCF7 | 5.7121749 | 6.4666729 | 4.8061969 | 5.8830199 | 5.9061484 | 5.9823243 |
| JIMT1 | 5.5254592 | 6.0603485 | 5.9699336 | 6.1005682 | 5.9958088 | 5.7960035 |
| MDAMB231 | 5.8790032 | 6.1291573 | 4.7999831 | 5.5578294 | 5.8447825 | 5.9023627 |
| MDAMB157 | 6.0301739 | 6.1228528 | 4.2381778 | 6.0285735 | 5.9744852 | 5.9033001 |
| MDAMB453 | 5.8726107 | 5.7872565 | 6.5337998 | 6.0293229 | 5.747528 | 5.8541081 |
| UACC812 | 6.0205135 | 6.3847721 | 7.3890452 | 5.5157176 | 6.0999343 | 6.1341813 |
| HMC18 | 5.6674335 | 5.6303195 | 4.94876 | 5.245812 | 4.834519 | 5.7943648 |
| Hs_578T | 5.9933079 | 5.6704974 | 3.9494807 | 5.7124412 | 5.8932912 | 6.0008438 |
| HCC1395 | 5.9476558 | 6.1526339 | 4.2147446 | 5.4298608 | 5.7408512 | 5.8380435 |

| **Cell line** | **trimethylamine-N-oxide** | **hexoses (HILIC pos)** | **adenosine** | **thymidine** | **xanthosine** | **2-deoxyadenosine** |
| --- | --- | --- | --- | --- | --- | --- |
| HDQP1 | 5.7551573 | 6.1144716 | 6.5408282 | 5.3913805 | 6.2158243 | 5.3463369 |
| HCC1937 | 6.2334255 | 5.9398491 | 6.2965356 | 6.9885106 | 6.5687182 | 6.586683 |
| MDAMB361 | 5.9208526 | 6.110844 | 5.6639295 | 6.6199858 | 5.8588007 | 5.404477 |
| HCC1143 | 5.8276809 | 5.9712404 | 4.8097347 | 5.2473466 | 6.2078924 | 5.647141 |
| HCC1599 | 6.1016792 | 5.1186767 | 5.806042 | 6.4465046 | 5.5914594 | 6.2993109 |
| HCC202 | 5.8023587 | 6.2803053 | 6.1049689 | 6.5084091 | 5.9777145 | 6.8791474 |
| BT20 | 6.1251709 | 5.1333555 | 5.7847124 | 5.9524425 | 5.7306754 | 5.8515097 |
| MDAMB468 | 5.8179572 | 6.0866876 | 6.3227943 | 5.8188004 | 5.7468049 | 5.7614417 |
| HCC1569 | 6.1292972 | 5.1588292 | 5.7267577 | 5.9715474 | 5.4964147 | 5.1244795 |
| HCC1954 | 5.9709607 | 5.8690514 | 5.6098565 | 5.4544275 | 5.6344951 | 6.0094757 |
| HCC1806 | 5.6262712 | 5.8031672 | 5.3288446 | 5.0625344 | 6.0282853 | 5.9028069 |
| MDAMB134VI | 6.3724007 | 5.07045 | 5.2560745 | 5.5257815 | 5.1891991 | 5.7931879 |
| HCC1419 | 5.5799336 | 5.8669927 | 5.9405997 | 5.7647141 | 5.7427353 | 6.6968464 |
| HCC1187 | 6.242255 | 5.3450756 | 5.8805716 | 6.1335619 | 6.1423311 | 5.6428134 |
| T47D | 5.7770855 | 5.4720603 | 4.2717454 | 6.0431901 | 5.7637761 | 6.5082153 |
| HCC38 | 5.9948475 | 5.6616701 | 4.6161292 | 5.9095873 | 5.8223424 | 5.5165153 |
| BT483 | 5.9185459 | 5.9814715 | 5.5055725 | 5.5529958 | 5.974267 | 5.8014303 |
| HCC2218 | 6.1333209 | 5.1238191 | 6.1413176 | 6.0640501 | 5.6592944 | 5.8615646 |
| MDAMB175VII | 5.7090042 | 6.2093844 | 5.9100485 | 5.8492676 | 6.5409387 | 5.6419077 |
| EFM19 | 6.0093742 | 6.0575686 | 5.7141117 | 6.3490739 | 5.902992 | 6.2048425 |
| CAMA1 | 5.9017321 | 5.1244756 | 6.0392149 | 5.8539781 | 5.2574374 | 6.1317186 |
| HCC70 | 5.8291261 | 5.8699418 | 5.9470751 | 5.0754734 | 5.8777539 | 5.6375872 |
| UACC893 | 6.3006315 | 5.1015193 | 5.1229715 | 5.2607908 | 5.3065478 | 5.2210002 |
| MDAMB415 | 5.8733779 | 5.9781692 | 5.72325 | 5.2270431 | 6.1425938 | 6.6004358 |
| HCC1428 | 5.9058985 | 5.8120141 | 5.1841306 | 5.1586519 | 5.6828111 | 5.8450615 |
| BT549 | 6.0246476 | 6.1805016 | 5.7729925 | 5.9043755 | 6.1393229 | 5.9393927 |
| SKBR3 | 5.7898082 | 6.060414 | 6.6355691 | 5.4721402 | 6.2113143 | 6.1085623 |
| CAL851 | 5.9642924 | 6.3680897 | 5.8100938 | 5.5657509 | 5.9154608 | 6.0326672 |
| CAL148 | 6.2571864 | 5.2679368 | 5.2339846 | 5.8237028 | 5.349963 | 5.5400052 |
| DU4475 | 6.0661641 | 5.3083487 | 5.621905 | 5.6046577 | 5.073039 | 6.9752521 |
| ZR7530 | 5.8298449 | 6.1140168 | 5.7149373 | 5.521106 | 5.8152379 | 6.1942763 |
| CAL120 | 5.866052 | 5.8237472 | 4.5433704 | 6.2797178 | 5.8311542 | 5.7439569 |
| EFM192A | 5.8472918 | 5.8803487 | 5.3026875 | 5.8594983 | 6.1036252 | 6.2708086 |
| CAL51 | 5.8309173 | 6.3598292 | 6.6673492 | 5.7188523 | 5.9577 | 6.0767714 |
| AU565 | 5.8362446 | 6.1392038 | 6.0215975 | 5.190882 | 5.8786862 | 5.9555608 |
| BT474 | 5.9809788 | 6.4082045 | 5.5440379 | 5.3949848 | 6.0126372 | 5.7683484 |
| ZR751 | 5.6726692 | 5.9668995 | 5.6525293 | 5.6167229 | 5.7062685 | 5.9402921 |
| MCF7 | 5.8695672 | 5.847657 | 6.2725013 | 6.4023391 | 5.8507392 | 6.5912441 |
| JIMT1 | 5.7050558 | 6.0449482 | 5.9879766 | 5.3034314 | 5.8347282 | 5.6537947 |
| MDAMB231 | 5.9998178 | 6.1079835 | 6.2685712 | 5.5124362 | 5.8159671 | 6.668417 |
| MDAMB157 | 5.9398455 | 5.6120199 | 4.7644185 | 5.5203072 | 5.8548356 | 4.9863451 |
| MDAMB453 | 5.9810475 | 6.0606045 | 5.4874815 | 5.1017346 | 5.6939654 | 6.5984117 |
| UACC812 | 5.8091646 | 6.0478637 | 5.8440264 | 6.3603282 | 6.1837925 | 5.9926523 |
| HMC18 | 6.4524446 | 5.3025996 | 6.1909406 | 6.7387759 | 5.3855778 | 6.4745821 |
| Hs_578T | 6.0615288 | 6.4150934 | 6.0817729 | 6.5116896 | 5.7859333 | 5.9017919 |
| HCC1395 | 6.1783707 | 6.3191267 | 5.4431096 | 5.4976129 | 5.6785245 | 5.4912237 |

| **Cell line** | **2-deoxycytidine** | **cAMP** | **cotinine** | **pipecolic acid** | **pyroglutamic acid** | **1-methyl nicotinamide** |
| --- | --- | --- | --- | --- | --- | --- |
| HDQP1 | 4.9008934 | 5.7045279 | 6.1246371 | 6.1519055 | 5.9842107 | 7.343506 |
| HCC1937 | 6.0969577 | 6.2299554 | 6.5469791 | 6.0888723 | 5.8132555 | 7.637424 |
| MDAMB361 | 6.0962593 | 5.6374169 | 6.1702404 | 5.9804889 | 5.6009477 | 4.3295785 |
| HCC1143 | 5.2168175 | 6.0268647 | 5.0536806 | 6.0772873 | 6.2318545 | 5.5086862 |
| HCC1599 | 7.0252654 | 6.1771026 | 6.2295979 | 5.3513533 | 4.9291824 | 4.7836298 |
| HCC202 | 6.6611736 | 5.9203068 | 5.7327469 | 5.4436119 | 6.2002261 | 4.1324578 |
| BT20 | 6.0915587 | 6.1003609 | 5.8999031 | 6.7803924 | 5.8356065 | 7.422795 |
| MDAMB468 | 6.094875 | 5.7400659 | 5.7624839 | 5.7914399 | 6.0175558 | 6.3613154 |
| HCC1569 | 5.0450439 | 5.9871232 | 5.6582833 | 5.7100792 | 4.7793794 | 6.5068744 |
| HCC1954 | 5.6980158 | 5.8411923 | 6.0189131 | 6.042152 | 5.9502355 | 7.3093765 |
| HCC1806 | 4.8644684 | 6.1624573 | 6.2382211 | 5.3527662 | 5.9398178 | 5.0740705 |
| MDAMB134VI | 5.9774826 | 6.2110939 | 5.8881311 | 5.893484 | 5.004218 | 4.1676763 |
| HCC1419 | 6.1751612 | 6.1041778 | 5.7228378 | 5.2189619 | 5.6704996 | 3.7819164 |
| HCC1187 | 6.1281496 | 5.7903205 | 6.0587203 | 4.8885944 | 5.2803235 | 5.9526731 |
| T47D | 6.5597669 | 6.0079228 | 5.0727288 | 5.787166 | 5.7949092 | 3.458982 |
| HCC38 | 5.5220042 | 5.7618116 | 5.549987 | 5.8024011 | 5.8527757 | 5.4451975 |
| BT483 | 6.7950348 | 6.3033754 | 5.8212413 | 6.278866 | 5.6205903 | 3.8869696 |
| HCC2218 | 5.8987128 | 6.0386492 | 6.3267023 | 5.7610988 | 4.8814806 | 4.1537177 |
| MDAMB175VII | 6.0435253 | 5.8939748 | 5.4867265 | 5.7832246 | 6.1654449 | 3.9331123 |
| EFM19 | 6.1792135 | 5.8364465 | 6.3916963 | 6.16081 | 5.9174917 | 4.7377722 |
| CAMA1 | 5.8975119 | 7.0158347 | 5.8564373 | 6.3171312 | 5.5743416 | 4.110105 |
| HCC70 | 5.5465615 | 5.8162952 | 6.1280196 | 5.4632821 | 6.0150932 | 7.3270669 |
| UACC893 | 5.8035928 | 5.7840566 | 5.6791881 | 5.8839763 | 5.073123 | 6.7380219 |
| MDAMB415 | 5.4374029 | 5.5016416 | 5.8837508 | 6.0560268 | 5.9373349 | 6.321611 |
| HCC1428 | 6.6903188 | 6.9596711 | 5.2504318 | 6.1077921 | 5.8619397 | 4.8419898 |
| BT549 | 6.544986 | 5.8792555 | 5.830029 | 5.8033999 | 6.0816246 | 7.2424122 |
| SKBR3 | 5.6461637 | 5.55215 | 5.260207 | 6.2163678 | 6.0489299 | 4.3597316 |
| CAL851 | 4.7816989 | 6.1389685 | 6.3510561 | 6.1840351 | 5.9549986 | 7.5537861 |
| CAL148 | 6.0195894 | 5.8045639 | 5.9565834 | 6.170677 | 5.3966517 | 4.199764 |
| DU4475 | 5.8087311 | 5.9787959 | 6.285296 | 6.3536259 | 5.0494359 | 3.8676708 |
| ZR7530 | 6.0718583 | 5.3404005 | 5.8471371 | 5.9171015 | 6.0032881 | 4.5800123 |
| CAL120 | 5.0479482 | 6.2917219 | 5.2605271 | 6.1174943 | 6.1047832 | 5.122785 |
| EFM192A | 5.413418 | 5.5337269 | 5.817848 | 5.0900645 | 5.9250866 | 3.7697776 |
| CAL51 | 6.1986814 | 6.0301774 | 5.9172017 | 6.2523216 | 6.1129806 | 5.2255166 |
| AU565 | 6.4002461 | 5.9139732 | 5.7088426 | 5.8100007 | 6.0625057 | 5.1973595 |
| BT474 | 5.5134294 | 5.6580396 | 5.7064958 | 5.4923048 | 6.2585966 | 3.9305804 |
| ZR751 | 6.3711945 | 5.8839763 | 6.3328237 | 5.7345771 | 5.8195985 | 3.8829325 |
| MCF7 | 6.956061 | 6.847157 | 6.2757765 | 6.4700529 | 5.8670263 | 3.8393019 |
| JIMT1 | 4.8855746 | 6.3774955 | 5.862867 | 5.1585943 | 6.3088008 | 5.6833699 |
| MDAMB231 | 5.298375 | 5.7100956 | 6.2049252 | 5.6761366 | 5.8634214 | 7.2532379 |
| MDAMB157 | 6.222906 | 6.0684441 | 5.8287901 | 5.8406755 | 5.8512006 | 7.0621473 |
| MDAMB453 | 5.6151563 | 6.1470406 | 6.1111596 | 5.1790375 | 5.9872843 | 4.2171824 |
| UACC812 | 6.8945467 | 6.459997 | 5.5820887 | 5.8890795 | 5.8848991 | 4.2909392 |
| HMC18 | 5.1193605 | 6.4159248 | 6.691618 | 6.7720803 | 4.7516656 | 5.9134703 |
| Hs_578T | 6.0925999 | 5.6966029 | 6.1740214 | 5.8929218 | 6.1732074 | 7.360613 |
| HCC1395 | 5.8524511 | 5.6945604 | 6.2504467 | 6.1093795 | 5.6933233 | 7.1018134 |

| **Cell line** | **butyrobetaine** | **putrescine** | **methionine sulfoxide** | **carnitine** | **acetylcarnitine** | **propionylcarnitine** |
| --- | --- | --- | --- | --- | --- | --- |
| HDQP1 | 6.0099561 | 5.7142976 | 5.8956513 | 6.055646 | 5.9904641 | 5.6845923 |
| HCC1937 | 5.7687341 | 6.1626764 | 6.1510869 | 5.8839763 | 5.6782083 | 6.312589 |
| MDAMB361 | 5.421321 | 4.9218004 | 5.7121188 | 5.3564646 | 5.7558067 | 5.8601884 |
| HCC1143 | 5.7950811 | 5.9154381 | 6.1670631 | 5.8769834 | 5.7034089 | 5.8500919 |
| HCC1599 | 5.8337115 | 5.3239282 | 5.0789749 | 5.4601436 | 5.4700486 | 5.9086936 |
| HCC202 | 5.6469844 | 5.6824935 | 6.3526976 | 5.7697063 | 5.7639587 | 5.7457157 |
| BT20 | 5.9957118 | 5.2493376 | 5.6030041 | 6.1488705 | 6.0970299 | 6.268112 |
| MDAMB468 | 5.480455 | 5.7784318 | 5.9306453 | 5.4262054 | 5.7070149 | 5.6791669 |
| HCC1569 | 5.5650558 | 5.1690754 | 5.3612898 | 5.4794374 | 5.0993165 | 5.6637923 |
| HCC1954 | 5.894054 | 5.8152767 | 5.7992598 | 5.9839784 | 6.0931529 | 5.7379006 |
| HCC1806 | 5.6628678 | 6.2158093 | 6.1548801 | 5.7247604 | 5.4230518 | 5.5899389 |
| MDAMB134VI | 6.0421143 | 6.184358 | 6.0337529 | 6.2769051 | 6.6263632 | 6.1388808 |
| HCC1419 | 5.9804627 | 5.6256624 | 5.8871701 | 5.9018769 | 5.9397083 | 6.4772624 |
| HCC1187 | 5.5753854 | 5.69853 | 5.7311546 | 5.7438672 | 5.4373189 | 5.5663366 |
| T47D | 6.1900189 | 6.2070087 | 5.6800818 | 6.3561702 | 6.1644864 | 6.8428596 |
| HCC38 | 5.7542627 | 5.319857 | 5.9696936 | 5.7462679 | 5.6391432 | 5.7781942 |
| BT483 | 6.1625205 | 5.8180983 | 5.7369732 | 6.2039979 | 6.320094 | 6.401023 |
| HCC2218 | 6.2817079 | 5.2585704 | 5.3756054 | 6.1859502 | 6.4140428 | 6.0069539 |
| MDAMB175VII | 5.7994501 | 5.3606508 | 5.9202095 | 5.7286683 | 5.2935439 | 5.8736255 |
| EFM19 | 5.9869937 | 5.3799287 | 6.1669594 | 5.8701443 | 5.9836236 | 5.679024 |
| CAMA1 | 5.9392298 | 5.1150756 | 5.4926289 | 6.0798118 | 6.0837207 | 6.2938882 |
| HCC70 | 5.525727 | 5.9563914 | 6.0237139 | 5.6420763 | 5.7109138 | 6.17058 |
| UACC893 | 5.4139889 | 5.3092691 | 5.8517481 | 5.8349831 | 6.1118352 | 5.6574634 |
| MDAMB415 | 6.293837 | 5.6286414 | 5.8746892 | 6.361796 | 6.446796 | 6.6051413 |
| HCC1428 | 6.3442641 | 5.9256946 | 5.8770699 | 6.2841264 | 6.4401248 | 6.4197179 |
| BT549 | 5.730108 | 5.8318803 | 6.0165514 | 5.796571 | 5.6485645 | 5.8164869 |
| SKBR3 | 5.6466241 | 5.1946141 | 6.6152429 | 5.8848263 | 6.0484949 | 5.6582846 |
| CAL851 | 5.8582536 | 6.1413973 | 6.0698309 | 5.9101723 | 5.7634776 | 6.5047919 |
| CAL148 | 5.921184 | 5.3873703 | 5.7320991 | 6.0866263 | 6.4387383 | 5.9308827 |
| DU4475 | 5.9020202 | 6.2687521 | 5.6802493 | 6.0667382 | 5.9590368 | 5.7564127 |
| ZR7530 | 5.7755908 | 5.1831609 | 5.9202879 | 5.8460299 | 5.9993944 | 5.7435087 |
| CAL120 | 5.8959281 | 6.1183671 | 6.0221447 | 5.8561249 | 5.7621093 | 6.5454891 |
| EFM192A | 5.8994928 | 5.7926911 | 5.9745435 | 5.8695024 | 6.0643813 | 5.8839763 |
| CAL51 | 5.842495 | 5.6598936 | 5.9077221 | 5.9080496 | 6.2218206 | 5.9117019 |
| AU565 | 5.5762537 | 5.7078591 | 5.872396 | 5.7722159 | 5.583226 | 5.7684017 |
| BT474 | 6.0352077 | 5.91654 | 5.9636989 | 5.9008569 | 5.8437339 | 6.8867027 |
| ZR751 | 6.0237179 | 5.8699968 | 5.8823222 | 6.0813127 | 6.1701627 | 6.089679 |
| MCF7 | 6.2316299 | 5.990671 | 5.5964169 | 6.1106542 | 6.3475804 | 6.6428714 |
| JIMT1 | 6.0768348 | 6.1651568 | 6.1827447 | 6.0976393 | 6.1121228 | 6.6574353 |
| MDAMB231 | 5.7990614 | 5.6812858 | 5.7933441 | 6.0542834 | 6.0027546 | 6.4246493 |
| MDAMB157 | 5.8376618 | 5.9585937 | 5.8526268 | 5.855076 | 5.6531752 | 5.6282172 |
| MDAMB453 | 5.8040754 | 5.7531862 | 6.0269893 | 5.8499522 | 5.8078255 | 5.6174674 |
| UACC812 | 6.3231298 | 5.1225571 | 5.6721753 | 6.1364318 | 6.3568075 | 6.1274216 |
| HMC18 | 4.6697161 | 5.1465486 | 5.1781207 | 4.678596 | 4.2579985 | 4.8770561 |
| Hs_578T | 5.3817221 | 5.5576846 | 6.1189203 | 5.5117701 | 5.4167961 | 5.7175924 |
| HCC1395 | 5.4670454 | 5.586108 | 5.8822794 | 5.7054426 | 5.3837258 | 5.5512115 |

| **Cell line** | **malonylcarnitine** | **butyrylcarnitine/ isobutyrylcarnitine** | **valerylcarnitine/isovalerylcarnitine/ 2-methylbutyroylcarnitine** | **hexanoylcarnitine** |
| --- | --- | --- | --- | --- |
| HDQP1 | 5.7345967 | 5.6874499 | 5.9920548 | 5.8387281 |
| HCC1937 | 5.8952933 | 5.929487 | 5.9805811 | 6.23371 |
| MDAMB361 | 5.8335716 | 5.9367991 | 6.3518535 | 6.0779008 |
| HCC1143 | 5.8346911 | 5.2678419 | 5.9840578 | 6.0204404 |
| HCC1599 | 5.6776386 | 6.3547525 | 6.427938 | 6.1517985 |
| HCC202 | 5.92376 | 5.2203041 | 5.5101242 | 5.9453246 |
| BT20 | 6.0632773 | 6.2236529 | 5.8839763 | 5.7767017 |
| MDAMB468 | 5.7151027 | 5.2871215 | 5.474199 | 5.6732192 |
| HCC1569 | 5.4698074 | 5.2323563 | 6.3191613 | 5.8751671 |
| HCC1954 | 6.1761126 | 6.0651123 | 6.5429847 | 5.6672452 |
| HCC1806 | 5.6333772 | 5.407435 | 5.1813859 | 5.6094977 |
| MDAMB134VI | 6.4432927 | 5.5554347 | 6.0725953 | 6.4540284 |
| HCC1419 | 6.2063392 | 6.1726934 | 6.9085433 | 6.4161496 |
| HCC1187 | 5.7842201 | 6.210794 | 6.3145381 | 6.1373337 |
| T47D | 6.3231864 | 6.3953758 | 6.8693889 | 6.4760115 |
| HCC38 | 5.7367478 | 5.9462579 | 5.5026124 | 6.060952 |
| BT483 | 6.1395107 | 5.7252508 | 6.1084296 | 6.2160803 |
| HCC2218 | 7.0673738 | 6.8317841 | 5.5093139 | 7.5649113 |
| MDAMB175VII | 6.2984772 | 5.1363128 | 5.97637 | 6.3199926 |
| EFM19 | 5.7518355 | 6.2656807 | 6.7932719 | 6.0985865 |
| CAMA1 | 5.8943089 | 6.2870379 | 6.2613758 | 5.9999925 |
| HCC70 | 5.7288513 | 5.5183027 | 5.671029 | 5.6832292 |
| UACC893 | 6.1502554 | 4.8436829 | 5.6479735 | 5.4991701 |
| MDAMB415 | 6.5981536 | 6.3926082 | 6.5780413 | 6.5820389 |
| HCC1428 | 6.7016261 | 7.0242305 | 6.0449226 | 6.7253178 |
| BT549 | 5.7131779 | 5.5259062 | 5.873651 | 6.0688158 |
| SKBR3 | 6.039244 | 6.5247423 | 6.3659084 | 6.1843843 |
| CAL851 | 5.8999096 | 5.8426398 | 6.6108367 | 5.7835144 |
| CAL148 | 6.2460311 | 6.3408377 | 5.8988721 | 6.1483102 |
| DU4475 | 6.5224054 | 6.4583264 | 5.3717054 | 5.8855109 |
| ZR7530 | 6.3806747 | 6.0094987 | 5.881907 | 6.3512387 |
| CAL120 | 5.7840373 | 5.5976008 | 6.2306887 | 5.7076511 |
| EFM192A | 6.1241648 | 6.3491572 | 6.7219977 | 6.9261656 |
| CAL51 | 5.930773 | 5.769968 | 5.6976504 | 5.9075921 |
| AU565 | 6.4887334 | 6.0201328 | 6.7882851 | 6.3565011 |
| BT474 | 6.5778734 | 6.621253 | 6.7618301 | 6.4253427 |
| ZR751 | 6.655988 | 6.6641958 | 6.12012 | 6.6885987 |
| MCF7 | 6.3214116 | 7.3164958 | 5.9360482 | 6.4340559 |
| JIMT1 | 5.9796032 | 5.7227664 | 6.0277144 | 5.6678786 |
| MDAMB231 | 5.7373265 | 5.3850273 | 5.8545556 | 5.7012854 |
| MDAMB157 | 5.8445948 | 5.2668589 | 6.5770463 | 5.6627896 |
| MDAMB453 | 5.74107 | 6.4174 | 6.7111372 | 6.3604407 |
| UACC812 | 6.29797 | 5.566998 | 5.738806 | 6.7248564 |
| HMC18 | 4.9901995 | 4.9525128 | 4.6786222 | 6.2042151 |
| Hs_578T | 5.5596649 | 5.31875 | 5.582783 | 5.7120528 |
| HCC1395 | 5.4252189 | 5.3520441 | 5.8666162 | 5.6312982 |

| **Cell line** | **heptanoylcarnitine** | **lauroylcarnitine** | **myristoylcarnitine** | **palmitoylcarnitine** |
| --- | --- | --- | --- | --- |
| HDQP1 | 5.7962433 | 5.2522929 | 5.0848482 | 5.0074342 |
| HCC1937 | 6.172265 | 6.4322078 | 6.4696259 | 6.4975051 |
| MDAMB361 | 5.9268231 | 6.7776175 | 6.9749643 | 7.1530984 |
| HCC1143 | 6.1461594 | 6.6743255 | 7.1575601 | 7.0479247 |
| HCC1599 | 6.1687512 | 5.9939516 | 7.1820395 | 7.2166135 |
| HCC202 | 6.1044838 | 6.0522865 | 6.0679524 | 6.0668205 |
| BT20 | 5.8982101 | 6.1168499 | 6.3069544 | 6.2855181 |
| MDAMB468 | 5.8694582 | 5.4989284 | 5.6454349 | 5.7167681 |
| HCC1569 | 5.7883712 | 6.1354048 | 6.29274 | 6.3610149 |
| HCC1954 | 5.3354668 | 5.8412278 | 5.8690196 | 5.6909605 |
| HCC1806 | 5.7875283 | 5.229127 | 5.3178625 | 5.5283999 |
| MDAMB134VI | 5.8258762 | 7.1917848 | 7.1877315 | 7.6105977 |
| HCC1419 | 6.2985389 | 6.5114553 | 6.7524553 | 6.3672257 |
| HCC1187 | 5.8545396 | 6.5877209 | 6.5577542 | 6.7095302 |
| T47D | 5.9268938 | 6.6376862 | 6.6914187 | 6.5005975 |
| HCC38 | 6.044479 | 6.8972169 | 6.7524924 | 6.7745796 |
| BT483 | 6.2200999 | 6.1643899 | 6.1793085 | 6.0764535 |
| HCC2218 | 7.1480145 | 8.0243472 | 7.8746314 | 8.1373235 |
| MDAMB175VII | 6.2010099 | 6.7729263 | 6.9619103 | 7.0139041 |
| EFM19 | 6.0126269 | 7.0224572 | 7.5465528 | 7.4695527 |
| CAMA1 | 5.3884285 | 6.1286608 | 6.3059297 | 6.4572732 |
| HCC70 | 5.8381518 | 5.9195219 | 5.9186864 | 5.8918339 |
| UACC893 | 6.1182759 | 6.0234972 | 7.2249956 | 7.6968391 |
| MDAMB415 | 5.9600323 | 7.1603081 | 6.7374704 | 6.9608962 |
| HCC1428 | 6.2249037 | 6.8682737 | 6.8148619 | 6.4225564 |
| BT549 | 6.1367994 | 5.8235115 | 5.9970958 | 6.2196354 |
| SKBR3 | 6.0522729 | 7.3174194 | 7.880633 | 7.8642977 |
| CAL851 | 5.8752093 | 6.1033868 | 5.9468844 | 5.8890424 |
| CAL148 | 5.5443399 | 6.5898702 | 6.4097385 | 6.9416997 |
| DU4475 | 5.4418873 | 6.9526369 | 7.1748737 | 6.9866504 |
| ZR7530 | 5.8629594 | 7.253768 | 7.4890081 | 7.2355897 |
| CAL120 | 5.5688827 | 6.0051727 | 5.9836322 | 6.1540542 |
| EFM192A | 6.0678685 | 7.9305012 | 7.8105881 | 7.583401 |
| CAL51 | 6.1313265 | 6.0244311 | 5.9718407 | 5.7293963 |
| AU565 | 5.9939606 | 7.4551734 | 7.7976874 | 7.7979854 |
| BT474 | 6.4573534 | 6.7033159 | 6.7836812 | 6.9514857 |
| ZR751 | 5.9352153 | 6.7780053 | 6.5398441 | 5.9379227 |
| MCF7 | 6.0369415 | 6.2420906 | 6.2707081 | 5.8151156 |
| JIMT1 | 5.7693235 | 6.1372359 | 5.9047654 | 5.7506174 |
| MDAMB231 | 5.8840289 | 5.6476906 | 5.6199252 | 5.7693328 |
| MDAMB157 | 5.6201286 | 5.9216022 | 6.117667 | 6.2555751 |
| MDAMB453 | 6.1624704 | 7.1723645 | 7.044028 | 7.0123363 |
| UACC812 | 6.2655473 | 6.4574595 | 6.3274599 | 6.4443744 |
| HMC18 | 6.3352813 | 6.3614236 | 6.3880619 | 6.4694524 |
| Hs_578T | 5.9947739 | 5.694446 | 5.7512851 | 5.9796687 |
| HCC1395 | 5.8289504 | 6.3388228 | 6.0629263 | 5.599071 |

| **Cell line** | **stearoylcarnitine** | **oleylcarnitine** | **arachidonyl_carnitine** | **sarcosine** | **beta-alanine** | **anserine** |
| --- | --- | --- | --- | --- | --- | --- |
| HDQP1 | 5.2258792 | 5.1407906 | 5.8729879 | 5.6902806 | 6.0479771 | 6.1039651 |
| HCC1937 | 6.6562116 | 6.9284213 | 6.1176344 | 6.2569278 | 5.4726837 | 5.8298799 |
| MDAMB361 | 7.2256157 | 7.0251399 | 5.7189561 | 5.725176 | 5.1072221 | 5.904506 |
| HCC1143 | 6.9931014 | 7.0744103 | 5.2188518 | 6.0129671 | 5.7848435 | 6.1578678 |
| HCC1599 | 6.5401332 | 7.4036973 | 6.0402741 | 5.6067512 | 5.397262 | 5.6310305 |
| HCC202 | 5.9373972 | 5.8636531 | 5.4913811 | 5.8578621 | 5.2085977 | 6.0213304 |
| BT20 | 6.4425828 | 6.2715608 | 6.0714876 | 5.7940236 | 6.2656914 | 6.00864 |
| MDAMB468 | 5.7179388 | 5.6690713 | 5.697907 | 6.0339455 | 5.308772 | 5.7711968 |
| HCC1569 | 5.6317851 | 6.3443012 | 5.9131126 | 5.8577042 | 5.1701504 | 5.4749944 |
| HCC1954 | 5.7806269 | 6.0501456 | 5.8000547 | 5.8550054 | 6.0561076 | 5.5817011 |
| HCC1806 | 5.6447255 | 5.2858289 | 5.6749989 | 6.2456318 | 5.9612175 | 5.9819483 |
| MDAMB134VI | 7.8334155 | 7.6679482 | 6.063707 | 6.1007878 | 5.2304633 | 5.7767368 |
| HCC1419 | 6.117062 | 6.248094 | 5.7996978 | 6.2641577 | 5.4692689 | 5.9049129 |
| HCC1187 | 6.6903099 | 6.437597 | 6.3225195 | 5.7468237 | 5.3620068 | 5.3980701 |
| T47D | 6.1166694 | 6.206625 | 6.1042178 | 6.1215095 | 5.6976699 | 5.8529334 |
| HCC38 | 7.1107073 | 6.9375872 | 5.3407457 | 6.1660669 | 6.2263965 | 6.1464344 |
| BT483 | 6.3354554 | 6.1276617 | 5.7973185 | 6.0015254 | 5.1842655 | 6.0677643 |
| HCC2218 | 7.9108938 | 7.852177 | 6.1483101 | 6.0804487 | 5.7552141 | 5.2265782 |
| MDAMB175VII | 6.7253571 | 6.6976412 | 5.9481098 | 5.6501804 | 5.4070429 | 6.0574338 |
| EFM19 | 7.2253493 | 7.2587488 | 5.706425 | 6.2245414 | 5.353586 | 6.0711699 |
| CAMA1 | 6.199761 | 6.1231055 | 5.9858111 | 6.2505058 | 4.9313893 | 5.5331702 |
| HCC70 | 5.1582935 | 5.7204176 | 5.6825324 | 6.2908198 | 5.7348423 | 6.2876738 |
| UACC893 | 7.9205547 | 7.9776235 | 6.1279749 | 5.6578265 | 5.3451402 | 5.6499147 |
| MDAMB415 | 6.3671764 | 6.8204275 | 5.7713704 | 5.9021742 | 5.8793685 | 6.1184795 |
| HCC1428 | 6.4567036 | 6.7060881 | 6.1727227 | 5.8126993 | 5.3942367 | 6.3211007 |
| BT549 | 6.3963504 | 6.4931816 | 5.8255904 | 5.8308465 | 5.5366268 | 6.2846225 |
| SKBR3 | 7.6618497 | 7.4501235 | 6.64804 | 5.6914295 | 5.2313537 | 6.0337392 |
| CAL851 | 5.9473784 | 5.9289517 | 6.0124041 | 5.893954 | 5.7283225 | 5.9455147 |
| CAL148 | 6.5911725 | 6.6215237 | 6.4687641 | 5.5597789 | 5.4949759 | 5.8550448 |
| DU4475 | 6.674157 | 6.9152666 | 6.1752477 | 5.6207106 | 5.9318236 | 6.0916479 |
| ZR7530 | 6.7474623 | 6.9982447 | 5.7092647 | 5.3429975 | 5.3144563 | 5.8348604 |
| CAL120 | 5.7535798 | 5.8978013 | 5.3565899 | 5.6618737 | 5.7106904 | 5.761011 |
| EFM192A | 7.193194 | 7.2635143 | 6.1685957 | 6.1231627 | 5.5871257 | 6.2863433 |
| CAL51 | 5.8883758 | 6.1885462 | 5.9374237 | 5.7652279 | 5.524897 | 5.9443558 |
| AU565 | 7.4395527 | 7.6040759 | 5.9992883 | 5.7575962 | 5.5166654 | 5.8800497 |
| BT474 | 6.8028658 | 6.5240969 | 6.1185303 | 5.983781 | 5.6078765 | 6.1223176 |
| ZR751 | 5.6934157 | 6.221932 | 6.05433 | 6.5160345 | 5.2805013 | 5.8338557 |
| MCF7 | 5.6430316 | 6.4590308 | 6.3756661 | 5.8924825 | 5.4807452 | 5.7797754 |
| JIMT1 | 5.7077307 | 5.5972784 | 5.6067272 | 6.1933067 | 6.2597307 | 5.9906171 |
| MDAMB231 | 6.1986595 | 5.7359893 | 5.9389835 | 5.491237 | 5.7259591 | 5.8535874 |
| MDAMB157 | 6.2655941 | 6.0998297 | 5.8429525 | 5.6230673 | 5.5628103 | 5.8936558 |
| MDAMB453 | 6.8112014 | 6.6936815 | 6.0765561 | 6.6448341 | 5.3563504 | 5.6890432 |
| UACC812 | 6.3789726 | 6.1780984 | 5.4445804 | 6.1001528 | 5.1858391 | 6.2121556 |
| HMC18 | 6.4851148 | 6.5343899 | 6.4133515 | 6.117722 | 5.2227648 | 5.714683 |
| Hs_578T | 6.3437404 | 6.1403068 | 6.0681584 | 5.6974904 | 5.4400649 | 5.9561111 |
| HCC1395 | 5.7182961 | 6.0938506 | 6.0680246 | 5.6920015 | 5.8350011 | 5.8529373 |

**Supplementary Table 6. Transcriptomics data in the 46 BC cell lines and stratification in tertiles based on TBC1D gene expression.**

| **Cell line** | **EVI5** | **EVI5 STATUS** | **EVI5L** | **EVI5L STATUS** | **GRTP1** | **GRTP1 STATUS** |
| --- | --- | --- | --- | --- | --- | --- |
| HDQP1 | 9.009 | LOWER TERTILE | 31.670 | UPPER TERTILE | 25.939 |  |
| HCC1937 | 16.101 |  | 40.282 | UPPER TERTILE | 40.126 | UPPER TERTILE |
| MDAMB361 | 8.717 | LOWER TERTILE | 12.034 | LOWER TERTILE | 23.462 |  |
| HCC1143 | 8.563 | LOWER TERTILE | 9.670 | LOWER TERTILE | 15.386 | LOWER TERTILE |
| HCC1599 | 9.250 | LOWER TERTILE | 11.048 | LOWER TERTILE | 12.630 | LOWER TERTILE |
| HCC202 | 14.002 |  | 40.096 | UPPER TERTILE | 41.778 | UPPER TERTILE |
| BT20 | 10.289 | LOWER TERTILE | 22.599 |  | 25.721 |  |
| MDAMB468 | 21.950 | UPPER TERTILE | 24.956 |  | 49.492 | UPPER TERTILE |
| HCC1569 | 8.841 | LOWER TERTILE | 29.176 |  | 10.190 | LOWER TERTILE |
| HCC1954 | 18.780 | UPPER TERTILE | 15.798 | LOWER TERTILE | 23.304 |  |
| HCC1806 | 13.424 |  | 21.252 |  | 54.071 | UPPER TERTILE |
| MDAMB134VI | 12.313 |  | 20.320 |  | 22.954 |  |
| HCC1419 | 20.759 | UPPER TERTILE | 35.252 | UPPER TERTILE | 37.255 | UPPER TERTILE |
| HCC1187 | 8.629 | LOWER TERTILE | 30.887 | UPPER TERTILE | 30.433 | UPPER TERTILE |
| T47D | 9.155 | LOWER TERTILE | 15.769 | LOWER TERTILE | 43.081 | UPPER TERTILE |
| HCC38 | 12.091 |  | 23.046 |  | 6.281 | LOWER TERTILE |
| BT483 | 13.475 |  | 22.327 |  | 34.453 | UPPER TERTILE |
| HCC2218 | 17.139 |  | 37.919 | UPPER TERTILE | 34.300 | UPPER TERTILE |
| MDAMB175VII | 11.346 |  | 31.105 | UPPER TERTILE | 54.169 | UPPER TERTILE |
| EFM19 | 17.824 |  | 31.355 | UPPER TERTILE | 50.198 | UPPER TERTILE |
| CAMA1 | 24.290 | UPPER TERTILE | 34.776 | UPPER TERTILE | 45.145 | UPPER TERTILE |
| HCC70 | 7.041 | LOWER TERTILE | 22.771 |  | 29.181 |  |
| UACC893 | 11.909 |  | 14.426 | LOWER TERTILE | 13.128 | LOWER TERTILE |
| MDAMB415 | 12.716 |  | 15.972 | LOWER TERTILE | 19.185 |  |
| HCC1428 | 14.266 |  | 17.868 |  | 27.261 |  |
| BT549 | 14.864 |  | 11.419 | LOWER TERTILE | 4.584 | LOWER TERTILE |
| SKBR3 | 21.699 | UPPER TERTILE | 18.660 |  | 18.161 |  |
| CAL851 | 11.367 |  | 9.650 | LOWER TERTILE | 22.588 |  |
| CAL148 | 7.489 | LOWER TERTILE | 60.030 | UPPER TERTILE | 21.134 |  |
| DU4475 | 4.742 | LOWER TERTILE | 13.388 | LOWER TERTILE | 16.078 | LOWER TERTILE |
| ZR7530 | 25.304 | UPPER TERTILE | 28.915 |  | 42.644 | UPPER TERTILE |
| CAL120 | 40.929 | UPPER TERTILE | 40.761 | UPPER TERTILE | 5.576 | LOWER TERTILE |
| EFM192A | 19.507 | UPPER TERTILE | 15.242 | LOWER TERTILE | 39.459 | UPPER TERTILE |
| CAL51 | 34.606 | UPPER TERTILE | 18.070 |  | 29.084 |  |
| AU565 | 25.606 | UPPER TERTILE | 17.492 | LOWER TERTILE | 21.130 |  |
| BT474 | 11.346 | LOWER TERTILE | 19.521 |  | 16.387 | LOWER TERTILE |
| ZR751 | 10.099 | LOWER TERTILE | 8.519 | LOWER TERTILE | 37.905 | UPPER TERTILE |
| MCF7 | 7.268 | LOWER TERTILE | 13.133 | LOWER TERTILE | 22.361 |  |
| JIMT1 | 18.174 | UPPER TERTILE | 43.536 | UPPER TERTILE | 30.313 |  |
| MDAMB231 | 73.237 | UPPER TERTILE | 57.704 | UPPER TERTILE | 1.916 | LOWER TERTILE |
| MDAMB157 | 23.544 | UPPER TERTILE | 28.766 |  | 9.077 | LOWER TERTILE |
| MDAMB453 | 10.331 | LOWER TERTILE | 46.586 | UPPER TERTILE | 16.660 |  |
| UACC812 | 11.356 |  | 19.420 |  | 13.472 | LOWER TERTILE |
| HMC18 | 28.225 | UPPER TERTILE | 17.136 | LOWER TERTILE | 8.711 | LOWER TERTILE |
| Hs_578T | 46.548 | UPPER TERTILE | 64.422 | UPPER TERTILE | 0.464 | LOWER TERTILE |
| HCC1395 | 17.695 |  | 25.508 |  | 3.731 | LOWER TERTILE |

| **Cell line** | **RABGAP1** | **RABGAP1 STATUS** | **RABGAP1L** | **RABGAP1L STATUS** | **SGSM1** | **SGSM1 STATUS** |
| --- | --- | --- | --- | --- | --- | --- |
| HDQP1 | 59.705 | LOWER TERTILE | 99.005 | UPPER TERTILE | 1.118 | LOWER TERTILE |
| HCC1937 | 114.987 | UPPER TERTILE | 58.365 | UPPER TERTILE | 6.371 | UPPER TERTILE |
| MDAMB361 | 84.540 | LOWER TERTILE | 14.365 | LOWER TERTILE | 2.809 |  |
| HCC1143 | 61.410 | LOWER TERTILE | 21.455 | LOWER TERTILE | 6.757 | UPPER TERTILE |
| HCC1599 | 99.124 |  | 24.023 |  | 1.831 |  |
| HCC202 | 107.036 |  | 28.009 |  | 1.809 |  |
| BT20 | 118.625 | UPPER TERTILE | 23.113 |  | 2.019 |  |
| MDAMB468 | 67.872 | LOWER TERTILE | 36.457 |  | 13.709 | UPPER TERTILE |
| HCC1569 | 61.464 | LOWER TERTILE | 27.819 |  | 1.323 | LOWER TERTILE |
| HCC1954 | 109.879 | UPPER TERTILE | 13.084 | LOWER TERTILE | 3.221 | UPPER TERTILE |
| HCC1806 | 96.700 |  | 49.991 | UPPER TERTILE | 7.138 | UPPER TERTILE |
| MDAMB134VI | 84.278 | LOWER TERTILE | 16.717 | LOWER TERTILE | 1.683 |  |
| HCC1419 | 199.322 | UPPER TERTILE | 28.228 |  | 19.600 | UPPER TERTILE |
| HCC1187 | 112.114 | UPPER TERTILE | 30.436 |  | 3.186 | UPPER TERTILE |
| T47D | 183.757 | UPPER TERTILE | 31.738 |  | 0.889 | LOWER TERTILE |
| HCC38 | 139.618 | UPPER TERTILE | 26.000 |  | 3.126 | UPPER TERTILE |
| BT483 | 116.148 | UPPER TERTILE | 37.226 |  | 1.473 |  |
| HCC2218 | 48.891 | LOWER TERTILE | 20.456 | LOWER TERTILE | 3.677 | UPPER TERTILE |
| MDAMB175VII | 98.555 |  | 55.791 | UPPER TERTILE | 21.466 | UPPER TERTILE |
| EFM19 | 144.091 | UPPER TERTILE | 59.717 | UPPER TERTILE | 1.790 |  |
| CAMA1 | 103.701 |  | 37.086 |  | 1.952 |  |
| HCC70 | 108.767 | UPPER TERTILE | 50.529 | UPPER TERTILE | 2.172 |  |
| UACC893 | 128.888 | UPPER TERTILE | 51.530 | UPPER TERTILE | 7.914 | UPPER TERTILE |
| MDAMB415 | 58.136 | LOWER TERTILE | 32.340 |  | 5.348 | UPPER TERTILE |
| HCC1428 | 93.332 |  | 42.048 | UPPER TERTILE | 2.065 |  |
| BT549 | 89.313 | LOWER TERTILE | 20.961 | LOWER TERTILE | 0.500 | LOWER TERTILE |
| SKBR3 | 93.893 |  | 64.426 | UPPER TERTILE | 1.472 |  |
| CAL851 | 95.149 |  | 39.910 | UPPER TERTILE | 0.319 | LOWER TERTILE |
| CAL148 | 85.816 | LOWER TERTILE | 26.949 |  | 0.960 | LOWER TERTILE |
| DU4475 | 45.771 | LOWER TERTILE | 54.996 | UPPER TERTILE | 2.885 |  |
| ZR7530 | 83.864 | LOWER TERTILE | 43.080 | UPPER TERTILE | 12.296 | UPPER TERTILE |
| CAL120 | 75.677 | LOWER TERTILE | 15.986 | LOWER TERTILE | 0.327 | LOWER TERTILE |
| EFM192A | 112.957 | UPPER TERTILE | 21.752 | LOWER TERTILE | 1.888 |  |
| CAL51 | 95.002 |  | 37.739 | UPPER TERTILE | 0.567 | LOWER TERTILE |
| AU565 | 89.878 |  | 59.482 | UPPER TERTILE | 2.231 |  |
| BT474 | 239.835 | UPPER TERTILE | 26.009 |  | 5.587 | UPPER TERTILE |
| ZR751 | 66.675 | LOWER TERTILE | 28.000 |  | 2.231 |  |
| MCF7 | 126.715 | UPPER TERTILE | 21.916 | LOWER TERTILE | 1.471 | LOWER TERTILE |
| JIMT1 | 117.621 | UPPER TERTILE | 39.945 | UPPER TERTILE | 2.904 |  |
| MDAMB231 | 84.729 | LOWER TERTILE | 22.039 | LOWER TERTILE | 0.795 | LOWER TERTILE |
| MDAMB157 | 73.299 | LOWER TERTILE | 9.625 | LOWER TERTILE | 1.149 | LOWER TERTILE |
| MDAMB453 | 99.843 |  | 9.025 | LOWER TERTILE | 0.625 | LOWER TERTILE |
| UACC812 | 48.542 | LOWER TERTILE | 15.906 | LOWER TERTILE | 4.418 | UPPER TERTILE |
| HMC18 | 96.010 |  | 22.526 |  | 0.898 | LOWER TERTILE |
| Hs_578T | 79.330 | LOWER TERTILE | 21.726 | LOWER TERTILE | 0.691 | LOWER TERTILE |
| HCC1395 | 51.458 | LOWER TERTILE | 14.993 | LOWER TERTILE | 0.662 | LOWER TERTILE |

| **Cell line** | **SGSM2** | **SGSM2 STATUS** | **SGSM3** | **SGSM3 STATUS** | **TBC1D1** | **TBC1D1 STATUS** |
| --- | --- | --- | --- | --- | --- | --- |
| HDQP1 | 106.444 | UPPER TERTILE | 57.462 |  | 69.688 | UPPER TERTILE |
| HCC1937 | 64.581 | LOWER TERTILE | 39.266 | LOWER TERTILE | 89.729 | UPPER TERTILE |
| MDAMB361 | 95.813 | UPPER TERTILE | 101.915 | UPPER TERTILE | 32.619 |  |
| HCC1143 | 145.717 | UPPER TERTILE | 57.200 |  | 47.988 |  |
| HCC1599 | 82.259 |  | 35.403 | LOWER TERTILE | 86.319 | UPPER TERTILE |
| HCC202 | 91.259 |  | 219.812 | UPPER TERTILE | 13.660 | LOWER TERTILE |
| BT20 | 57.721 | LOWER TERTILE | 41.400 | LOWER TERTILE | 121.354 | UPPER TERTILE |
| MDAMB468 | 55.411 | LOWER TERTILE | 52.374 |  | 88.863 | UPPER TERTILE |
| HCC1569 | 96.583 | UPPER TERTILE | 69.613 |  | 193.606 | UPPER TERTILE |
| HCC1954 | 50.521 | LOWER TERTILE | 48.044 |  | 72.827 | UPPER TERTILE |
| HCC1806 | 85.955 |  | 60.326 |  | 49.065 |  |
| MDAMB134VI | 87.011 |  | 92.073 |  | 15.885 | LOWER TERTILE |
| HCC1419 | 117.605 | UPPER TERTILE | 160.921 | UPPER TERTILE | 5.551 | LOWER TERTILE |
| HCC1187 | 63.224 | LOWER TERTILE | 39.811 | LOWER TERTILE | 41.914 |  |
| T47D | 143.830 | UPPER TERTILE | 111.961 | UPPER TERTILE | 25.868 |  |
| HCC38 | 35.285 | LOWER TERTILE | 28.118 | LOWER TERTILE | 110.526 | UPPER TERTILE |
| BT483 | 94.315 |  | 45.283 | LOWER TERTILE | 26.716 |  |
| HCC2218 | 106.142 | UPPER TERTILE | 126.884 | UPPER TERTILE | 10.602 | LOWER TERTILE |
| MDAMB175VII | 103.390 | UPPER TERTILE | 254.150 | UPPER TERTILE | 10.833 | LOWER TERTILE |
| EFM19 | 114.397 | UPPER TERTILE | 37.496 | LOWER TERTILE | 15.344 | LOWER TERTILE |
| CAMA1 | 74.098 |  | 103.032 | UPPER TERTILE | 1.579 | LOWER TERTILE |
| HCC70 | 67.752 | LOWER TERTILE | 88.649 |  | 113.788 | UPPER TERTILE |
| UACC893 | 433.438 | UPPER TERTILE | 103.885 | UPPER TERTILE | 30.764 |  |
| MDAMB415 | 221.006 | UPPER TERTILE | 197.106 | UPPER TERTILE | 35.351 |  |
| HCC1428 | 50.964 | LOWER TERTILE | 27.062 | LOWER TERTILE | 9.021 | LOWER TERTILE |
| BT549 | 86.396 |  | 35.063 | LOWER TERTILE | 54.666 |  |
| SKBR3 | 50.792 | LOWER TERTILE | 104.934 | UPPER TERTILE | 21.312 | LOWER TERTILE |
| CAL851 | 80.698 |  | 75.871 |  | 61.505 |  |
| CAL148 | 134.269 | UPPER TERTILE | 69.575 |  | 44.141 |  |
| DU4475 | 92.621 |  | 37.908 | LOWER TERTILE | 106.843 | UPPER TERTILE |
| ZR7530 | 95.588 |  | 135.332 | UPPER TERTILE | 3.578 | LOWER TERTILE |
| CAL120 | 53.688 | LOWER TERTILE | 35.347 | LOWER TERTILE | 73.561 | UPPER TERTILE |
| EFM192A | 74.603 |  | 44.341 | LOWER TERTILE | 42.856 |  |
| CAL51 | 100.208 | UPPER TERTILE | 64.926 |  | 69.576 | UPPER TERTILE |
| AU565 | 89.653 |  | 107.685 | UPPER TERTILE | 60.322 |  |
| BT474 | 70.770 | LOWER TERTILE | 79.716 |  | 5.228 | LOWER TERTILE |
| ZR751 | 65.704 | LOWER TERTILE | 54.491 |  | 13.568 | LOWER TERTILE |
| MCF7 | 184.170 | UPPER TERTILE | 38.651 | LOWER TERTILE | 17.391 | LOWER TERTILE |
| JIMT1 | 36.124 | LOWER TERTILE | 54.641 |  | 53.032 |  |
| MDAMB231 | 63.551 | LOWER TERTILE | 37.664 | LOWER TERTILE | 53.474 |  |
| MDAMB157 | 71.283 |  | 46.085 | LOWER TERTILE | 63.971 |  |
| MDAMB453 | 73.553 |  | 197.499 | UPPER TERTILE | 1.584 | LOWER TERTILE |
| UACC812 | 153.292 | UPPER TERTILE | 113.559 | UPPER TERTILE | 4.523 | LOWER TERTILE |
| HMC18 | 59.494 | LOWER TERTILE | 56.961 |  | 67.979 | UPPER TERTILE |
| Hs_578T | 89.868 |  | 127.372 | UPPER TERTILE | 75.915 | UPPER TERTILE |
| HCC1395 | 93.276 |  | 90.739 |  | 74.130 | UPPER TERTILE |

| **Cell line** | **TBC1D10A** | **TBC1D10A STATUS** | **TBC1D10B** | **TBC1D10B STATUS** | **TBC1D10C** | **TBC1D10C STATUS** |
| --- | --- | --- | --- | --- | --- | --- |
| HDQP1 | 15.618 | LOWER TERTILE | 38.627 | LOWER TERTILE | 0.557 | UPPER TERTILE |
| HCC1937 | 16.014 | LOWER TERTILE | 99.080 | UPPER TERTILE | 0.388 |  |
| MDAMB361 | 30.625 | UPPER TERTILE | 70.884 |  | 0.034 | LOWER TERTILE |
| HCC1143 | 31.939 | UPPER TERTILE | 73.122 |  | 0.111 | LOWER TERTILE |
| HCC1599 | 16.338 | LOWER TERTILE | 47.455 | LOWER TERTILE | 0.072 | LOWER TERTILE |
| HCC202 | 24.762 |  | 122.976 | UPPER TERTILE | 0.886 | UPPER TERTILE |
| BT20 | 23.888 |  | 57.623 |  | 0.441 | UPPER TERTILE |
| MDAMB468 | 28.922 |  | 51.668 | LOWER TERTILE | 0.251 |  |
| HCC1569 | 23.709 |  | 56.141 |  | 0.041 | LOWER TERTILE |
| HCC1954 | 27.358 |  | 50.539 | LOWER TERTILE | 0.101 | LOWER TERTILE |
| HCC1806 | 23.237 |  | 43.023 | LOWER TERTILE | 0.179 |  |
| MDAMB134VI | 39.203 | UPPER TERTILE | 43.274 | LOWER TERTILE | 1.906 | UPPER TERTILE |
| HCC1419 | 55.620 | UPPER TERTILE | 77.921 |  | 0.195 |  |
| HCC1187 | 14.781 | LOWER TERTILE | 66.477 |  | 0.716 | UPPER TERTILE |
| T47D | 23.188 |  | 74.878 |  | 0.325 |  |
| HCC38 | 12.434 | LOWER TERTILE | 66.596 |  | 0.594 | UPPER TERTILE |
| BT483 | 30.962 | UPPER TERTILE | 74.770 |  | 0.918 | UPPER TERTILE |
| HCC2218 | 47.283 | UPPER TERTILE | 67.214 |  | 0.283 |  |
| MDAMB175VII | 55.294 | UPPER TERTILE | 61.751 |  | 1.076 | UPPER TERTILE |
| EFM19 | 28.559 |  | 90.219 | UPPER TERTILE | 0.383 |  |
| CAMA1 | 35.601 | UPPER TERTILE | 69.196 |  | 0.199 |  |
| HCC70 | 53.451 | UPPER TERTILE | 34.072 | LOWER TERTILE | 0.101 | LOWER TERTILE |
| UACC893 | 12.030 | LOWER TERTILE | 52.645 | LOWER TERTILE | 0.315 |  |
| MDAMB415 | 42.032 | UPPER TERTILE | 80.325 | UPPER TERTILE | 0.155 |  |
| HCC1428 | 26.629 |  | 75.637 |  | 0.476 | UPPER TERTILE |
| BT549 | 10.795 | LOWER TERTILE | 66.189 |  | 0.013 | LOWER TERTILE |
| SKBR3 | 33.221 | UPPER TERTILE | 84.394 | UPPER TERTILE | 0.450 | UPPER TERTILE |
| CAL851 | 24.711 |  | 37.012 | LOWER TERTILE | 0.109 | LOWER TERTILE |
| CAL148 | 15.125 | LOWER TERTILE | 188.196 | UPPER TERTILE | 0.147 | LOWER TERTILE |
| DU4475 | 6.980 | LOWER TERTILE | 68.085 |  | 12.849 | UPPER TERTILE |
| ZR7530 | 46.342 | UPPER TERTILE | 54.814 | LOWER TERTILE | 0.367 |  |
| CAL120 | 10.584 | LOWER TERTILE | 110.762 | UPPER TERTILE | 0.075 | LOWER TERTILE |
| EFM192A | 22.688 |  | 84.860 | UPPER TERTILE | 0.150 |  |
| CAL51 | 5.396 | LOWER TERTILE | 58.286 |  | 0.575 | UPPER TERTILE |
| AU565 | 20.709 | LOWER TERTILE | 82.960 | UPPER TERTILE | 0.549 | UPPER TERTILE |
| BT474 | 26.121 |  | 55.801 | LOWER TERTILE | 0.408 | UPPER TERTILE |
| ZR751 | 29.369 |  | 41.651 | LOWER TERTILE | 4.078 | UPPER TERTILE |
| MCF7 | 22.242 | LOWER TERTILE | 62.687 |  | 0.218 |  |
| JIMT1 | 29.332 |  | 61.802 |  | 0.020 | LOWER TERTILE |
| MDAMB231 | 18.522 | LOWER TERTILE | 50.504 | LOWER TERTILE | 0.043 | LOWER TERTILE |
| MDAMB157 | 46.801 | UPPER TERTILE | 42.697 | LOWER TERTILE | 0.243 |  |
| MDAMB453 | 63.252 | UPPER TERTILE | 181.502 | UPPER TERTILE | 0.245 |  |
| UACC812 | 22.635 |  | 62.265 |  | 0.058 | LOWER TERTILE |
| HMC18 | 17.048 | LOWER TERTILE | 60.381 |  | 0.071 | LOWER TERTILE |
| Hs_578T | 68.236 | UPPER TERTILE | 53.402 | LOWER TERTILE | 0.100 | LOWER TERTILE |
| HCC1395 | 40.792 | UPPER TERTILE | 67.269 |  | 0.255 |  |

| **Cell line** | **TBC1D12** | **TBC1D12 STATUS** | **TBC1D13** | **TBC1D13 STATUS** | **TBC1D14** | **TBC1D14 STATUS** |
| --- | --- | --- | --- | --- | --- | --- |
| HDQP1 | 4.591 | LOWER TERTILE | 25.339 | LOWER TERTILE | 71.734 |  |
| HCC1937 | 5.177 | LOWER TERTILE | 38.423 |  | 72.006 |  |
| MDAMB361 | 11.527 |  | 42.963 |  | 109.780 | UPPER TERTILE |
| HCC1143 | 12.203 |  | 30.531 | LOWER TERTILE | 82.383 |  |
| HCC1599 | 20.966 | UPPER TERTILE | 49.051 | UPPER TERTILE | 91.685 |  |
| HCC202 | 16.793 |  | 60.332 | UPPER TERTILE | 73.144 |  |
| BT20 | 4.459 | LOWER TERTILE | 87.329 | UPPER TERTILE | 70.263 |  |
| MDAMB468 | 10.803 | LOWER TERTILE | 45.218 | UPPER TERTILE | 62.460 | LOWER TERTILE |
| HCC1569 | 16.175 |  | 44.777 | UPPER TERTILE | 105.454 | UPPER TERTILE |
| HCC1954 | 11.722 |  | 44.718 |  | 93.038 |  |
| HCC1806 | 11.583 |  | 23.201 | LOWER TERTILE | 39.300 | LOWER TERTILE |
| MDAMB134VI | 17.999 | UPPER TERTILE | 25.171 | LOWER TERTILE | 68.564 | LOWER TERTILE |
| HCC1419 | 23.420 | UPPER TERTILE | 32.915 |  | 131.843 | UPPER TERTILE |
| HCC1187 | 12.274 |  | 55.340 | UPPER TERTILE | 95.076 |  |
| T47D | 21.856 | UPPER TERTILE | 48.545 | UPPER TERTILE | 93.656 |  |
| HCC38 | 10.764 | LOWER TERTILE | 31.989 | LOWER TERTILE | 85.638 |  |
| BT483 | 11.370 |  | 50.122 | UPPER TERTILE | 154.715 | UPPER TERTILE |
| HCC2218 | 18.270 | UPPER TERTILE | 37.176 |  | 69.882 |  |
| MDAMB175VII | 15.861 |  | 53.565 | UPPER TERTILE | 63.904 | LOWER TERTILE |
| EFM19 | 14.945 |  | 54.621 | UPPER TERTILE | 64.163 | LOWER TERTILE |
| CAMA1 | 28.629 | UPPER TERTILE | 39.571 |  | 126.516 | UPPER TERTILE |
| HCC70 | 5.961 | LOWER TERTILE | 40.840 |  | 69.649 | LOWER TERTILE |
| UACC893 | 12.998 |  | 37.009 |  | 70.989 |  |
| MDAMB415 | 8.451 | LOWER TERTILE | 20.175 | LOWER TERTILE | 42.612 | LOWER TERTILE |
| HCC1428 | 18.661 | UPPER TERTILE | 32.768 | LOWER TERTILE | 55.195 | LOWER TERTILE |
| BT549 | 16.875 |  | 45.247 | UPPER TERTILE | 82.098 |  |
| SKBR3 | 10.881 | LOWER TERTILE | 44.053 |  | 58.052 | LOWER TERTILE |
| CAL851 | 9.063 | LOWER TERTILE | 29.492 | LOWER TERTILE | 53.972 | LOWER TERTILE |
| CAL148 | 14.567 |  | 75.126 | UPPER TERTILE | 223.152 | UPPER TERTILE |
| DU4475 | 7.389 | LOWER TERTILE | 30.078 | LOWER TERTILE | 72.687 |  |
| ZR7530 | 23.616 | UPPER TERTILE | 27.349 | LOWER TERTILE | 162.836 | UPPER TERTILE |
| CAL120 | 9.366 | LOWER TERTILE | 37.745 |  | 124.295 | UPPER TERTILE |
| EFM192A | 14.658 |  | 35.143 |  | 112.274 | UPPER TERTILE |
| CAL51 | 17.448 | UPPER TERTILE | 41.024 |  | 99.968 | UPPER TERTILE |
| AU565 | 10.966 |  | 37.166 |  | 102.058 | UPPER TERTILE |
| BT474 | 13.292 |  | 70.095 | UPPER TERTILE | 111.855 | UPPER TERTILE |
| ZR751 | 16.723 |  | 22.185 | LOWER TERTILE | 83.560 |  |
| MCF7 | 17.173 | UPPER TERTILE | 35.609 |  | 69.501 | LOWER TERTILE |
| JIMT1 | 8.907 | LOWER TERTILE | 60.115 | UPPER TERTILE | 103.887 | UPPER TERTILE |
| MDAMB231 | 23.158 | UPPER TERTILE | 64.839 | UPPER TERTILE | 108.567 | UPPER TERTILE |
| MDAMB157 | 9.247 | LOWER TERTILE | 30.723 | LOWER TERTILE | 50.395 | LOWER TERTILE |
| MDAMB453 | 18.251 | UPPER TERTILE | 38.018 |  | 72.778 |  |
| UACC812 | 20.826 | UPPER TERTILE | 19.748 | LOWER TERTILE | 58.481 | LOWER TERTILE |
| HMC18 | 9.749 | LOWER TERTILE | 40.231 |  | 140.449 | UPPER TERTILE |
| Hs_578T | 17.751 | UPPER TERTILE | 28.845 | LOWER TERTILE | 55.224 | LOWER TERTILE |
| HCC1395 | 7.387 | LOWER TERTILE | 25.185 | LOWER TERTILE | 35.297 | LOWER TERTILE |

| **Cell line** | **TBC1D15** | **TBC1D15 STATUS** | **TBC1D16** | **TBC1D16 STATUS** | **TBC1D17** | **TBC1D17 STATUS** |
| --- | --- | --- | --- | --- | --- | --- |
| HDQP1 | 33.537 |  | 75.601 | LOWER TERTILE | 24.087 | LOWER TERTILE |
| HCC1937 | 26.641 | LOWER TERTILE | 76.712 | LOWER TERTILE | 29.259 |  |
| MDAMB361 | 34.177 |  | 127.638 |  | 33.502 |  |
| HCC1143 | 75.833 | UPPER TERTILE | 42.876 | LOWER TERTILE | 43.114 | UPPER TERTILE |
| HCC1599 | 41.180 |  | 148.176 | UPPER TERTILE | 11.378 | LOWER TERTILE |
| HCC202 | 36.081 |  | 292.000 | UPPER TERTILE | 47.964 | UPPER TERTILE |
| BT20 | 35.874 |  | 51.445 | LOWER TERTILE | 47.718 | UPPER TERTILE |
| MDAMB468 | 32.650 | LOWER TERTILE | 121.660 |  | 35.786 |  |
| HCC1569 | 58.445 | UPPER TERTILE | 97.806 |  | 23.199 | LOWER TERTILE |
| HCC1954 | 40.446 |  | 137.805 |  | 26.486 | LOWER TERTILE |
| HCC1806 | 32.086 | LOWER TERTILE | 72.838 | LOWER TERTILE | 48.974 | UPPER TERTILE |
| MDAMB134VI | 77.913 | UPPER TERTILE | 53.217 | LOWER TERTILE | 25.457 | LOWER TERTILE |
| HCC1419 | 64.662 | UPPER TERTILE | 147.645 | UPPER TERTILE | 56.675 | UPPER TERTILE |
| HCC1187 | 24.427 | LOWER TERTILE | 231.050 | UPPER TERTILE | 22.834 | LOWER TERTILE |
| T47D | 49.650 | UPPER TERTILE | 97.385 |  | 38.993 |  |
| HCC38 | 30.872 | LOWER TERTILE | 124.250 |  | 27.562 | LOWER TERTILE |
| BT483 | 28.981 | LOWER TERTILE | 230.911 | UPPER TERTILE | 25.821 | LOWER TERTILE |
| HCC2218 | 25.332 | LOWER TERTILE | 313.137 | UPPER TERTILE | 40.555 | UPPER TERTILE |
| MDAMB175VII | 37.981 |  | 129.607 |  | 33.289 |  |
| EFM19 | 55.602 | UPPER TERTILE | 252.317 | UPPER TERTILE | 42.273 | UPPER TERTILE |
| CAMA1 | 37.687 |  | 238.066 | UPPER TERTILE | 30.749 |  |
| HCC70 | 42.789 |  | 249.674 | UPPER TERTILE | 35.214 |  |
| UACC893 | 56.239 | UPPER TERTILE | 53.250 | LOWER TERTILE | 28.319 | LOWER TERTILE |
| MDAMB415 | 59.067 | UPPER TERTILE | 90.611 | LOWER TERTILE | 68.628 | UPPER TERTILE |
| HCC1428 | 37.266 |  | 139.083 |  | 41.284 | UPPER TERTILE |
| BT549 | 51.816 | UPPER TERTILE | 61.447 | LOWER TERTILE | 30.672 |  |
| SKBR3 | 48.092 |  | 148.473 | UPPER TERTILE | 38.534 |  |
| CAL851 | 59.491 | UPPER TERTILE | 37.729 | LOWER TERTILE | 31.118 |  |
| CAL148 | 33.386 | LOWER TERTILE | 357.801 | UPPER TERTILE | 37.710 |  |
| DU4475 | 36.704 |  | 47.079 | LOWER TERTILE | 27.334 | LOWER TERTILE |
| ZR7530 | 57.724 | UPPER TERTILE | 144.925 |  | 53.326 | UPPER TERTILE |
| CAL120 | 31.014 | LOWER TERTILE | 178.422 | UPPER TERTILE | 43.964 | UPPER TERTILE |
| EFM192A | 28.557 | LOWER TERTILE | 109.818 |  | 67.814 | UPPER TERTILE |
| CAL51 | 56.880 | UPPER TERTILE | 80.291 | LOWER TERTILE | 23.283 | LOWER TERTILE |
| AU565 | 62.846 | UPPER TERTILE | 121.239 |  | 34.014 |  |
| BT474 | 43.549 |  | 118.462 |  | 40.377 |  |
| ZR751 | 73.879 | UPPER TERTILE | 109.539 |  | 25.655 | LOWER TERTILE |
| MCF7 | 27.585 | LOWER TERTILE | 65.759 | LOWER TERTILE | 34.843 |  |
| JIMT1 | 19.147 | LOWER TERTILE | 275.031 | UPPER TERTILE | 51.757 | UPPER TERTILE |
| MDAMB231 | 37.109 |  | 62.190 | LOWER TERTILE | 23.158 | LOWER TERTILE |
| MDAMB157 | 31.401 | LOWER TERTILE | 165.010 | UPPER TERTILE | 7.089 | LOWER TERTILE |
| MDAMB453 | 32.119 | LOWER TERTILE | 230.230 | UPPER TERTILE | 56.051 | UPPER TERTILE |
| UACC812 | 37.334 |  | 60.552 | LOWER TERTILE | 38.470 |  |
| HMC18 | 76.165 | UPPER TERTILE | 95.199 |  | 34.466 |  |
| Hs_578T | 39.283 |  | 90.873 |  | 51.010 | UPPER TERTILE |
| HCC1395 | 20.208 | LOWER TERTILE | 111.288 |  | 18.154 | LOWER TERTILE |

| **Cell line** | **TBC1D19** | **TBC1D19 STATUS** | **TBC1D2** | **TBC1D2 STATUS** | **TBC1D20** | **TBC1D20 STATUS** |
| --- | --- | --- | --- | --- | --- | --- |
| HDQP1 | 5.412 |  | 45.148 | UPPER TERTILE | 20.183 | LOWER TERTILE |
| HCC1937 | 3.088 |  | 94.839 | UPPER TERTILE | 60.818 |  |
| MDAMB361 | 5.716 | UPPER TERTILE | 9.499 | LOWER TERTILE | 40.538 | LOWER TERTILE |
| HCC1143 | 3.340 |  | 26.163 |  | 63.575 |  |
| HCC1599 | 3.402 |  | 1.686 | LOWER TERTILE | 9.865 | LOWER TERTILE |
| HCC202 | 2.902 |  | 15.153 |  | 56.010 |  |
| BT20 | 2.173 | LOWER TERTILE | 44.175 | UPPER TERTILE | 30.197 | LOWER TERTILE |
| MDAMB468 | 2.394 | LOWER TERTILE | 59.759 | UPPER TERTILE | 67.974 |  |
| HCC1569 | 5.117 |  | 3.849 | LOWER TERTILE | 119.229 | UPPER TERTILE |
| HCC1954 | 4.340 |  | 55.730 | UPPER TERTILE | 42.812 | LOWER TERTILE |
| HCC1806 | 4.003 |  | 56.697 | UPPER TERTILE | 40.573 | LOWER TERTILE |
| MDAMB134VI | 17.412 | UPPER TERTILE | 4.279 | LOWER TERTILE | 35.600 | LOWER TERTILE |
| HCC1419 | 4.483 |  | 23.063 |  | 74.822 | UPPER TERTILE |
| HCC1187 | 2.261 | LOWER TERTILE | 1.526 | LOWER TERTILE | 94.406 | UPPER TERTILE |
| T47D | 5.337 |  | 18.309 |  | 57.936 |  |
| HCC38 | 2.601 | LOWER TERTILE | 33.305 |  | 128.665 | UPPER TERTILE |
| BT483 | 12.523 | UPPER TERTILE | 17.577 |  | 104.734 | UPPER TERTILE |
| HCC2218 | 0.600 | LOWER TERTILE | 16.415 |  | 53.450 |  |
| MDAMB175VII | 3.423 |  | 33.381 |  | 145.912 | UPPER TERTILE |
| EFM19 | 2.430 | LOWER TERTILE | 34.800 | UPPER TERTILE | 70.614 | UPPER TERTILE |
| CAMA1 | 6.460 | UPPER TERTILE | 11.255 |  | 64.701 |  |
| HCC70 | 1.159 | LOWER TERTILE | 27.775 |  | 48.968 |  |
| UACC893 | 1.905 | LOWER TERTILE | 17.427 |  | 48.044 | LOWER TERTILE |
| MDAMB415 | 5.272 |  | 40.749 | UPPER TERTILE | 65.928 |  |
| HCC1428 | 2.807 | LOWER TERTILE | 10.101 | LOWER TERTILE | 30.549 | LOWER TERTILE |
| BT549 | 4.975 |  | 113.710 | UPPER TERTILE | 79.115 | UPPER TERTILE |
| SKBR3 | 2.609 | LOWER TERTILE | 50.096 | UPPER TERTILE | 125.699 | UPPER TERTILE |
| CAL851 | 2.884 | LOWER TERTILE | 41.728 | UPPER TERTILE | 65.845 |  |
| CAL148 | 7.283 | UPPER TERTILE | 11.072 | LOWER TERTILE | 105.515 | UPPER TERTILE |
| DU4475 | 7.439 | UPPER TERTILE | 2.926 | LOWER TERTILE | 56.989 |  |
| ZR7530 | 6.460 | UPPER TERTILE | 23.480 |  | 98.471 | UPPER TERTILE |
| CAL120 | 5.526 | UPPER TERTILE | 75.771 | UPPER TERTILE | 56.308 |  |
| EFM192A | 5.505 | UPPER TERTILE | 35.577 | UPPER TERTILE | 37.190 | LOWER TERTILE |
| CAL51 | 14.027 | UPPER TERTILE | 5.909 | LOWER TERTILE | 45.028 | LOWER TERTILE |
| AU565 | 1.977 | LOWER TERTILE | 25.792 |  | 96.782 | UPPER TERTILE |
| BT474 | 4.282 |  | 7.619 | LOWER TERTILE | 88.708 | UPPER TERTILE |
| ZR751 | 9.507 | UPPER TERTILE | 13.714 |  | 32.864 | LOWER TERTILE |
| MCF7 | 2.220 | LOWER TERTILE | 8.875 | LOWER TERTILE | 47.216 | LOWER TERTILE |
| JIMT1 | 2.303 | LOWER TERTILE | 45.695 | UPPER TERTILE | 82.014 | UPPER TERTILE |
| MDAMB231 | 3.454 |  | 120.855 | UPPER TERTILE | 59.853 |  |
| MDAMB157 | 18.212 | UPPER TERTILE | 8.032 | LOWER TERTILE | 67.516 |  |
| MDAMB453 | 2.788 | LOWER TERTILE | 9.795 | LOWER TERTILE | 55.734 |  |
| UACC812 | 9.662 | UPPER TERTILE | 11.106 | LOWER TERTILE | 29.797 | LOWER TERTILE |
| HMC18 | 3.596 |  | 6.347 | LOWER TERTILE | 44.973 | LOWER TERTILE |
| Hs_578T | 12.922 | UPPER TERTILE | 27.424 |  | 106.760 | UPPER TERTILE |
| HCC1395 | 11.072 | UPPER TERTILE | 14.384 |  | 69.258 |  |

| **Cell line** | **TBC1D21** | **TBC1D21 STATUS** | **TBC1D22A** | **TBC1D22A STATUS** | **TBC1D22B** | **TBC1D22B STATUS** |
| --- | --- | --- | --- | --- | --- | --- |
| HDQP1 | 0.157 | UPPER TERTILE | 32.898 | UPPER TERTILE | 26.459 |  |
| HCC1937 | 0.080 |  | 26.904 |  | 23.787 |  |
| MDAMB361 | 0.034 |  | 16.259 | LOWER TERTILE | 17.677 | LOWER TERTILE |
| HCC1143 | 0.412 | UPPER TERTILE | 34.667 | UPPER TERTILE | 16.564 | LOWER TERTILE |
| HCC1599 | 0.054 |  | 17.649 | LOWER TERTILE | 27.928 |  |
| HCC202 | 0.033 |  | 13.633 | LOWER TERTILE | 18.333 | LOWER TERTILE |
| BT20 | 0.000 | LOWER TERTILE | 29.728 | UPPER TERTILE | 30.958 | UPPER TERTILE |
| MDAMB468 | 0.023 |  | 32.528 | UPPER TERTILE | 28.515 |  |
| HCC1569 | 0.000 | LOWER TERTILE | 29.135 | UPPER TERTILE | 44.035 | UPPER TERTILE |
| HCC1954 | 0.000 | LOWER TERTILE | 26.506 |  | 34.186 | UPPER TERTILE |
| HCC1806 | 0.000 | LOWER TERTILE | 22.450 |  | 27.616 |  |
| MDAMB134VI | 0.052 |  | 14.702 | LOWER TERTILE | 18.248 | LOWER TERTILE |
| HCC1419 | 0.000 | LOWER TERTILE | 24.650 |  | 31.681 | UPPER TERTILE |
| HCC1187 | 0.483 | UPPER TERTILE | 19.357 |  | 41.444 | UPPER TERTILE |
| T47D | 0.046 |  | 18.595 |  | 16.029 | LOWER TERTILE |
| HCC38 | 0.405 | UPPER TERTILE | 27.181 |  | 35.719 | UPPER TERTILE |
| BT483 | 0.162 | UPPER TERTILE | 14.483 | LOWER TERTILE | 16.713 | LOWER TERTILE |
| HCC2218 | 0.035 |  | 33.554 | UPPER TERTILE | 22.441 |  |
| MDAMB175VII | 0.000 | LOWER TERTILE | 35.351 | UPPER TERTILE | 14.765 | LOWER TERTILE |
| EFM19 | 0.116 |  | 16.848 | LOWER TERTILE | 28.824 |  |
| CAMA1 | 0.199 | UPPER TERTILE | 21.633 |  | 29.090 |  |
| HCC70 | 0.000 | LOWER TERTILE | 39.060 | UPPER TERTILE | 40.497 | UPPER TERTILE |
| UACC893 | 0.048 |  | 27.379 |  | 26.930 |  |
| MDAMB415 | 0.155 | UPPER TERTILE | 36.344 | UPPER TERTILE | 52.966 | UPPER TERTILE |
| HCC1428 | 0.000 | LOWER TERTILE | 13.632 | LOWER TERTILE | 16.682 | LOWER TERTILE |
| BT549 | 0.247 | UPPER TERTILE | 27.658 |  | 31.949 | UPPER TERTILE |
| SKBR3 | 0.000 | LOWER TERTILE | 8.615 | LOWER TERTILE | 22.124 |  |
| CAL851 | 0.000 | LOWER TERTILE | 40.730 | UPPER TERTILE | 29.736 | UPPER TERTILE |
| CAL148 | 0.000 | LOWER TERTILE | 14.447 | LOWER TERTILE | 22.347 |  |
| DU4475 | 0.376 | UPPER TERTILE | 20.123 |  | 13.161 | LOWER TERTILE |
| ZR7530 | 0.193 | UPPER TERTILE | 40.066 | UPPER TERTILE | 19.610 | LOWER TERTILE |
| CAL120 | 0.000 | LOWER TERTILE | 25.251 |  | 37.690 | UPPER TERTILE |
| EFM192A | 0.033 |  | 14.809 | LOWER TERTILE | 30.262 | UPPER TERTILE |
| CAL51 | 0.031 |  | 17.634 | LOWER TERTILE | 14.742 | LOWER TERTILE |
| AU565 | 0.000 | LOWER TERTILE | 7.439 | LOWER TERTILE | 25.016 |  |
| BT474 | 0.019 |  | 19.354 |  | 21.260 |  |
| ZR751 | 0.000 | LOWER TERTILE | 14.004 | LOWER TERTILE | 11.174 | LOWER TERTILE |
| MCF7 | 0.156 | UPPER TERTILE | 18.405 |  | 19.752 |  |
| JIMT1 | 0.157 | UPPER TERTILE | 24.015 |  | 34.331 | UPPER TERTILE |
| MDAMB231 | 0.107 |  | 24.718 |  | 41.715 | UPPER TERTILE |
| MDAMB157 | 0.283 | UPPER TERTILE | 11.932 | LOWER TERTILE | 18.300 | LOWER TERTILE |
| MDAMB453 | 0.000 | LOWER TERTILE | 41.860 | UPPER TERTILE | 23.922 |  |
| UACC812 | 0.000 | LOWER TERTILE | 15.568 | LOWER TERTILE | 15.917 | LOWER TERTILE |
| HMC18 | 0.000 | LOWER TERTILE | 28.485 | UPPER TERTILE | 18.387 | LOWER TERTILE |
| Hs_578T | 0.698 | UPPER TERTILE | 38.544 | UPPER TERTILE | 32.028 | UPPER TERTILE |
| HCC1395 | 0.238 | UPPER TERTILE | 29.059 | UPPER TERTILE | 27.602 |  |

| **Cell line** | **TBC1D23** | **TBC1D23 STATUS** | **TBC1D24** | **TBC1D24 STATUS** | **TBC1D25** | **TBC1D25 STATUS** |
| --- | --- | --- | --- | --- | --- | --- |
| HDQP1 | 32.853 |  | 15.968 | LOWER TERTILE | 9.009 | LOWER TERTILE |
| HCC1937 | 26.783 | LOWER TERTILE | 21.914 | LOWER TERTILE | 9.992 | LOWER TERTILE |
| MDAMB361 | 26.163 | LOWER TERTILE | 56.496 | UPPER TERTILE | 14.501 |  |
| HCC1143 | 47.070 | UPPER TERTILE | 45.283 | UPPER TERTILE | 7.106 | LOWER TERTILE |
| HCC1599 | 39.348 |  | 27.104 |  | 8.092 | LOWER TERTILE |
| HCC202 | 34.339 |  | 30.125 |  | 10.184 | LOWER TERTILE |
| BT20 | 57.391 | UPPER TERTILE | 22.683 |  | 12.710 |  |
| MDAMB468 | 41.252 |  | 19.942 | LOWER TERTILE | 20.763 | UPPER TERTILE |
| HCC1569 | 67.013 | UPPER TERTILE | 19.272 | LOWER TERTILE | 40.724 | UPPER TERTILE |
| HCC1954 | 48.774 | UPPER TERTILE | 33.059 |  | 20.564 | UPPER TERTILE |
| HCC1806 | 39.484 |  | 20.262 | LOWER TERTILE | 19.288 | UPPER TERTILE |
| MDAMB134VI | 31.459 | LOWER TERTILE | 22.580 |  | 12.263 | LOWER TERTILE |
| HCC1419 | 55.913 | UPPER TERTILE | 32.480 |  | 13.017 |  |
| HCC1187 | 31.510 | LOWER TERTILE | 63.047 | UPPER TERTILE | 10.701 | LOWER TERTILE |
| T47D | 32.699 |  | 17.190 | LOWER TERTILE | 19.208 | UPPER TERTILE |
| HCC38 | 41.982 | UPPER TERTILE | 30.281 |  | 8.842 | LOWER TERTILE |
| BT483 | 37.871 |  | 35.086 | UPPER TERTILE | 18.602 |  |
| HCC2218 | 44.774 | UPPER TERTILE | 37.837 | UPPER TERTILE | 13.711 |  |
| MDAMB175VII | 28.353 | LOWER TERTILE | 20.879 | LOWER TERTILE | 16.408 |  |
| EFM19 | 52.773 | UPPER TERTILE | 40.932 | UPPER TERTILE | 17.674 |  |
| CAMA1 | 38.148 |  | 31.452 |  | 17.035 |  |
| HCC70 | 109.875 | UPPER TERTILE | 65.554 | UPPER TERTILE | 8.631 | LOWER TERTILE |
| UACC893 | 40.300 |  | 28.978 |  | 5.228 | LOWER TERTILE |
| MDAMB415 | 33.979 |  | 14.234 | LOWER TERTILE | 25.883 | UPPER TERTILE |
| HCC1428 | 27.321 | LOWER TERTILE | 39.785 | UPPER TERTILE | 13.329 |  |
| BT549 | 59.784 | UPPER TERTILE | 22.174 | LOWER TERTILE | 33.945 | UPPER TERTILE |
| SKBR3 | 28.078 | LOWER TERTILE | 50.106 | UPPER TERTILE | 17.423 |  |
| CAL851 | 57.463 | UPPER TERTILE | 15.775 | LOWER TERTILE | 18.604 |  |
| CAL148 | 21.615 | LOWER TERTILE | 38.615 | UPPER TERTILE | 20.235 | UPPER TERTILE |
| DU4475 | 21.153 | LOWER TERTILE | 61.067 | UPPER TERTILE | 10.397 | LOWER TERTILE |
| ZR7530 | 42.880 | UPPER TERTILE | 31.162 |  | 11.779 | LOWER TERTILE |
| CAL120 | 41.416 |  | 18.425 | LOWER TERTILE | 18.001 |  |
| EFM192A | 40.134 |  | 26.063 |  | 21.603 | UPPER TERTILE |
| CAL51 | 28.319 | LOWER TERTILE | 26.446 |  | 9.813 | LOWER TERTILE |
| AU565 | 28.317 | LOWER TERTILE | 40.965 | UPPER TERTILE | 21.758 | UPPER TERTILE |
| BT474 | 36.391 |  | 26.295 |  | 13.774 |  |
| ZR751 | 37.597 |  | 38.202 | UPPER TERTILE | 8.422 | LOWER TERTILE |
| MCF7 | 38.074 |  | 33.016 |  | 15.535 |  |
| JIMT1 | 26.251 | LOWER TERTILE | 27.935 |  | 34.707 | UPPER TERTILE |
| MDAMB231 | 66.442 | UPPER TERTILE | 17.897 | LOWER TERTILE | 41.403 | UPPER TERTILE |
| MDAMB157 | 32.600 |  | 22.871 |  | 44.951 | UPPER TERTILE |
| MDAMB453 | 22.133 | LOWER TERTILE | 43.442 | UPPER TERTILE | 15.220 |  |
| UACC812 | 24.521 | LOWER TERTILE | 33.285 | UPPER TERTILE | 11.470 | LOWER TERTILE |
| HMC18 | 52.465 | UPPER TERTILE | 22.085 | LOWER TERTILE | 20.485 | UPPER TERTILE |
| Hs_578T | 152.677 | UPPER TERTILE | 22.284 | LOWER TERTILE | 30.565 | UPPER TERTILE |
| HCC1395 | 31.656 | LOWER TERTILE | 20.412 | LOWER TERTILE | 15.030 |  |

| **Cell line** | **TBC1D26** | **TBC1D26 STATUS** | **TBC1D28** | **TBC1D28 STATUS** | **TBC1D2B** | **TBC1D2B STATUS** |
| --- | --- | --- | --- | --- | --- | --- |
| HDQP1 | 0.274 | UPPER TERTILE | 0.665 | UPPER TERTILE | 30.748 |  |
| HCC1937 | 0.054 |  | 0.067 |  | 32.095 |  |
| MDAMB361 | 0.068 |  | 0.034 |  | 40.698 |  |
| HCC1143 | 0.251 | UPPER TERTILE | 0.129 | UPPER TERTILE | 73.138 | UPPER TERTILE |
| HCC1599 | 0.233 | UPPER TERTILE | 0.233 | UPPER TERTILE | 23.261 | LOWER TERTILE |
| HCC202 | 0.049 |  | 0.049 |  | 13.365 | LOWER TERTILE |
| BT20 | 0.083 |  | 0.114 | UPPER TERTILE | 73.184 | UPPER TERTILE |
| MDAMB468 | 0.023 |  | 0.023 |  | 40.888 |  |
| HCC1569 | 0.020 |  | 0.020 |  | 53.131 | UPPER TERTILE |
| HCC1954 | 0.000 | LOWER TERTILE | 0.020 |  | 33.566 |  |
| HCC1806 | 0.552 | UPPER TERTILE | 0.110 | UPPER TERTILE | 33.729 |  |
| MDAMB134VI | 0.605 | UPPER TERTILE | 0.503 | UPPER TERTILE | 24.010 | LOWER TERTILE |
| HCC1419 | 0.081 |  | 0.065 |  | 25.936 | LOWER TERTILE |
| HCC1187 | 0.062 |  | 0.062 |  | 19.283 | LOWER TERTILE |
| T47D | 0.000 | LOWER TERTILE | 0.000 | LOWER TERTILE | 20.586 | LOWER TERTILE |
| HCC38 | 0.121 | UPPER TERTILE | 0.132 | UPPER TERTILE | 27.979 | LOWER TERTILE |
| BT483 | 0.018 |  | 0.018 |  | 40.011 |  |
| HCC2218 | 0.000 | LOWER TERTILE | 0.018 | LOWER TERTILE | 57.938 | UPPER TERTILE |
| MDAMB175VII | 0.000 | LOWER TERTILE | 0.018 | LOWER TERTILE | 77.595 | UPPER TERTILE |
| EFM19 | 0.017 | LOWER TERTILE | 0.050 |  | 74.941 | UPPER TERTILE |
| CAMA1 | 0.158 | UPPER TERTILE | 0.184 | UPPER TERTILE | 24.175 | LOWER TERTILE |
| HCC70 | 0.000 | LOWER TERTILE | 0.017 | LOWER TERTILE | 42.654 |  |
| UACC893 | 0.000 | LOWER TERTILE | 0.048 |  | 49.740 | UPPER TERTILE |
| MDAMB415 | 0.022 |  | 0.000 | LOWER TERTILE | 25.971 | LOWER TERTILE |
| HCC1428 | 0.000 | LOWER TERTILE | 0.000 | LOWER TERTILE | 21.658 | LOWER TERTILE |
| BT549 | 0.039 |  | 0.143 | UPPER TERTILE | 24.449 | LOWER TERTILE |
| SKBR3 | 0.016 | LOWER TERTILE | 0.016 | LOWER TERTILE | 67.631 | UPPER TERTILE |
| CAL851 | 0.087 |  | 0.043 |  | 40.511 |  |
| CAL148 | 0.241 | UPPER TERTILE | 1.727 | UPPER TERTILE | 47.782 |  |
| DU4475 | 0.000 | LOWER TERTILE | 0.000 | LOWER TERTILE | 127.843 | UPPER TERTILE |
| ZR7530 | 0.000 | LOWER TERTILE | 0.000 | LOWER TERTILE | 70.480 | UPPER TERTILE |
| CAL120 | 0.075 |  | 0.000 | LOWER TERTILE | 40.030 |  |
| EFM192A | 0.504 | UPPER TERTILE | 0.465 | UPPER TERTILE | 43.974 |  |
| CAL51 | 0.187 | UPPER TERTILE | 0.264 | UPPER TERTILE | 39.453 |  |
| AU565 | 0.016 | LOWER TERTILE | 0.000 | LOWER TERTILE | 65.891 | UPPER TERTILE |
| BT474 | 0.111 | UPPER TERTILE | 0.037 |  | 42.509 |  |
| ZR751 | 0.000 | LOWER TERTILE | 0.000 | LOWER TERTILE | 32.039 |  |
| MCF7 | 0.107 | UPPER TERTILE | 0.033 |  | 59.536 | UPPER TERTILE |
| JIMT1 | 0.059 |  | 0.059 |  | 116.110 | UPPER TERTILE |
| MDAMB231 | 0.000 | LOWER TERTILE | 0.000 | LOWER TERTILE | 23.821 | LOWER TERTILE |
| MDAMB157 | 0.249 | UPPER TERTILE | 0.183 | UPPER TERTILE | 28.955 | LOWER TERTILE |
| MDAMB453 | 0.067 |  | 0.089 | UPPER TERTILE | 8.880 | LOWER TERTILE |
| UACC812 | 0.058 |  | 0.000 | LOWER TERTILE | 49.426 |  |
| HMC18 | 0.018 | LOWER TERTILE | 0.018 | LOWER TERTILE | 21.050 | LOWER TERTILE |
| Hs_578T | 0.125 | UPPER TERTILE | 0.025 |  | 76.139 | UPPER TERTILE |
| HCC1395 | 0.127 | UPPER TERTILE | 0.076 | UPPER TERTILE | 59.456 | UPPER TERTILE |

| **Cell line** | **TBC1D3** | **TBC1D3 STATUS** | **TBC1D30** | **TBC1D30 STATUS** | **TBC1D31** | **TBC1D31 STATUS** |
| --- | --- | --- | --- | --- | --- | --- |
| HDQP1 | 7.154 | UPPER TERTILE | 1.339 | LOWER TERTILE | 17.997 |  |
| HCC1937 | 0.264 |  | 6.181 | LOWER TERTILE | 27.146 |  |
| MDAMB361 | 0.233 | LOWER TERTILE | 38.095 |  | 15.896 | LOWER TERTILE |
| HCC1143 | 0.485 |  | 14.783 |  | 36.734 | UPPER TERTILE |
| HCC1599 | 0.732 |  | 34.685 |  | 49.690 | UPPER TERTILE |
| HCC202 | 2.183 | UPPER TERTILE | 49.804 | UPPER TERTILE | 22.746 |  |
| BT20 | 0.985 | UPPER TERTILE | 6.968 | LOWER TERTILE | 11.468 | LOWER TERTILE |
| MDAMB468 | 0.283 |  | 9.777 |  | 12.148 | LOWER TERTILE |
| HCC1569 | 0.125 | LOWER TERTILE | 7.944 |  | 19.881 |  |
| HCC1954 | 0.793 |  | 19.469 |  | 40.500 | UPPER TERTILE |
| HCC1806 | 0.211 | LOWER TERTILE | 7.793 | LOWER TERTILE | 42.344 | UPPER TERTILE |
| MDAMB134VI | 0.421 |  | 23.629 |  | 9.710 | LOWER TERTILE |
| HCC1419 | 0.078 | LOWER TERTILE | 93.339 | UPPER TERTILE | 79.326 | UPPER TERTILE |
| HCC1187 | 0.622 |  | 1.246 | LOWER TERTILE | 10.810 | LOWER TERTILE |
| T47D | 1.259 | UPPER TERTILE | 43.325 |  | 15.087 | LOWER TERTILE |
| HCC38 | 0.295 |  | 5.490 | LOWER TERTILE | 19.998 |  |
| BT483 | 0.216 | LOWER TERTILE | 43.646 | UPPER TERTILE | 10.183 | LOWER TERTILE |
| HCC2218 | 4.550 | UPPER TERTILE | 63.345 | UPPER TERTILE | 19.083 |  |
| MDAMB175VII | 1.002 | UPPER TERTILE | 49.295 | UPPER TERTILE | 13.749 | LOWER TERTILE |
| EFM19 | 0.528 |  | 31.255 |  | 12.033 | LOWER TERTILE |
| CAMA1 | 6.300 | UPPER TERTILE | 47.653 | UPPER TERTILE | 16.036 | LOWER TERTILE |
| HCC70 | 0.638 |  | 10.663 |  | 20.587 |  |
| UACC893 | 0.000 | LOWER TERTILE | 52.911 | UPPER TERTILE | 28.706 | UPPER TERTILE |
| MDAMB415 | 0.462 |  | 25.883 |  | 9.181 | LOWER TERTILE |
| HCC1428 | 1.730 | UPPER TERTILE | 30.318 |  | 30.016 | UPPER TERTILE |
| BT549 | 0.000 | LOWER TERTILE | 0.715 | LOWER TERTILE | 34.620 | UPPER TERTILE |
| SKBR3 | 0.035 | LOWER TERTILE | 52.427 | UPPER TERTILE | 102.924 | UPPER TERTILE |
| CAL851 | 0.875 | UPPER TERTILE | 4.347 | LOWER TERTILE | 17.148 |  |
| CAL148 | 2.040 | UPPER TERTILE | 59.208 | UPPER TERTILE | 4.464 | LOWER TERTILE |
| DU4475 | 0.233 |  | 4.577 | LOWER TERTILE | 16.936 |  |
| ZR7530 | 2.494 | UPPER TERTILE | 89.067 | UPPER TERTILE | 30.657 | UPPER TERTILE |
| CAL120 | 0.332 |  | 10.340 |  | 24.140 |  |
| EFM192A | 0.271 |  | 57.998 | UPPER TERTILE | 166.870 | UPPER TERTILE |
| CAL51 | 0.207 | LOWER TERTILE | 1.151 | LOWER TERTILE | 17.464 |  |
| AU565 | 0.078 | LOWER TERTILE | 69.578 | UPPER TERTILE | 90.146 | UPPER TERTILE |
| BT474 | 2.544 | UPPER TERTILE | 78.140 | UPPER TERTILE | 11.798 | LOWER TERTILE |
| ZR751 | 3.057 | UPPER TERTILE | 40.777 |  | 16.614 |  |
| MCF7 | 0.248 |  | 65.245 | UPPER TERTILE | 18.331 |  |
| JIMT1 | 0.184 | LOWER TERTILE | 26.428 |  | 26.693 |  |
| MDAMB231 | 0.193 | LOWER TERTILE | 1.495 | LOWER TERTILE | 18.117 |  |
| MDAMB157 | 0.579 |  | 1.417 | LOWER TERTILE | 37.511 | UPPER TERTILE |
| MDAMB453 | 3.619 | UPPER TERTILE | 66.911 | UPPER TERTILE | 14.725 | LOWER TERTILE |
| UACC812 | 3.679 | UPPER TERTILE | 43.037 |  | 16.430 | LOWER TERTILE |
| HMC18 | 0.152 | LOWER TERTILE | 0.194 | LOWER TERTILE | 24.769 |  |
| Hs_578T | 0.000 | LOWER TERTILE | 0.324 | LOWER TERTILE | 32.719 | UPPER TERTILE |
| HCC1395 | 0.078 | LOWER TERTILE | 0.306 | LOWER TERTILE | 33.676 | UPPER TERTILE |

| **Cell line** | **TBC1D32** | **TBC1D32 STATUS** | **TBC1D3E** | **TBC1D3E STATUS** | **TBC1D3G** | **TBC1D3G STATUS** |
| --- | --- | --- | --- | --- | --- | --- |
| HDQP1 | 14.837 | UPPER TERTILE | 6.858 |  | 57.923 | UPPER TERTILE |
| HCC1937 | 4.257 | LOWER TERTILE | 7.388 |  | 7.644 | UPPER TERTILE |
| MDAMB361 | 2.806 | LOWER TERTILE | 1.870 | LOWER TERTILE | 3.260 | LOWER TERTILE |
| HCC1143 | 9.718 | UPPER TERTILE | 5.306 |  | 4.041 |  |
| HCC1599 | 8.353 | UPPER TERTILE | 10.191 | UPPER TERTILE | 15.707 | UPPER TERTILE |
| HCC202 | 3.690 | LOWER TERTILE | 16.664 | UPPER TERTILE | 19.661 | UPPER TERTILE |
| BT20 | 3.600 | LOWER TERTILE | 9.524 | UPPER TERTILE | 4.972 |  |
| MDAMB468 | 7.909 |  | 3.825 | LOWER TERTILE | 3.079 | LOWER TERTILE |
| HCC1569 | 6.115 |  | 4.293 | LOWER TERTILE | 3.811 | LOWER TERTILE |
| HCC1954 | 1.988 | LOWER TERTILE | 5.859 |  | 6.759 |  |
| HCC1806 | 7.644 |  | 5.489 |  | 4.360 |  |
| MDAMB134VI | 19.336 | UPPER TERTILE | 4.798 |  | 14.940 | UPPER TERTILE |
| HCC1419 | 14.493 | UPPER TERTILE | 2.399 | LOWER TERTILE | 3.507 | LOWER TERTILE |
| HCC1187 | 2.540 | LOWER TERTILE | 5.662 |  | 5.854 |  |
| T47D | 6.073 |  | 13.641 | UPPER TERTILE | 14.193 | UPPER TERTILE |
| HCC38 | 6.376 |  | 4.299 |  | 5.632 |  |
| BT483 | 5.504 |  | 5.836 |  | 9.109 | UPPER TERTILE |
| HCC2218 | 5.672 |  | 2.127 | LOWER TERTILE | 2.601 | LOWER TERTILE |
| MDAMB175VII | 4.959 |  | 11.671 | UPPER TERTILE | 15.867 | UPPER TERTILE |
| EFM19 | 13.747 | UPPER TERTILE | 14.060 | UPPER TERTILE | 9.824 | UPPER TERTILE |
| CAMA1 | 4.255 | LOWER TERTILE | 4.947 |  | 6.546 |  |
| HCC70 | 3.429 | LOWER TERTILE | 12.129 | UPPER TERTILE | 10.023 | UPPER TERTILE |
| UACC893 | 12.417 | UPPER TERTILE | 3.384 | LOWER TERTILE | 5.215 |  |
| MDAMB415 | 12.090 | UPPER TERTILE | 9.842 | UPPER TERTILE | 5.299 |  |
| HCC1428 | 6.157 |  | 9.342 |  | 3.499 | LOWER TERTILE |
| BT549 | 8.285 | UPPER TERTILE | 3.424 | LOWER TERTILE | 3.143 | LOWER TERTILE |
| SKBR3 | 6.075 |  | 1.693 | LOWER TERTILE | 2.182 | LOWER TERTILE |
| CAL851 | 5.066 |  | 25.022 | UPPER TERTILE | 6.044 |  |
| CAL148 | 4.758 | LOWER TERTILE | 3.617 | LOWER TERTILE | 1.399 | LOWER TERTILE |
| DU4475 | 11.327 | UPPER TERTILE | 4.346 |  | 4.016 | LOWER TERTILE |
| ZR7530 | 8.104 | UPPER TERTILE | 40.304 | UPPER TERTILE | 92.086 | UPPER TERTILE |
| CAL120 | 7.961 |  | 11.855 | UPPER TERTILE | 15.231 | UPPER TERTILE |
| EFM192A | 4.608 | LOWER TERTILE | 2.485 | LOWER TERTILE | 3.769 | LOWER TERTILE |
| CAL51 | 1.011 | LOWER TERTILE | 6.102 |  | 8.128 | UPPER TERTILE |
| AU565 | 6.417 |  | 1.829 | LOWER TERTILE | 0.926 | LOWER TERTILE |
| BT474 | 7.865 |  | 50.911 | UPPER TERTILE | 46.335 | UPPER TERTILE |
| ZR751 | 10.219 | UPPER TERTILE | 16.410 | UPPER TERTILE | 21.011 | UPPER TERTILE |
| MCF7 | 3.385 | LOWER TERTILE | 3.178 | LOWER TERTILE | 5.035 |  |
| JIMT1 | 4.375 | LOWER TERTILE | 2.733 | LOWER TERTILE | 1.140 | LOWER TERTILE |
| MDAMB231 | 2.199 | LOWER TERTILE | 4.203 | LOWER TERTILE | 4.955 |  |
| MDAMB157 | 9.705 | UPPER TERTILE | 14.346 | UPPER TERTILE | 2.507 | LOWER TERTILE |
| MDAMB453 | 3.860 | LOWER TERTILE | 6.931 |  | 3.564 | LOWER TERTILE |
| UACC812 | 8.642 | UPPER TERTILE | 32.724 | UPPER TERTILE | 6.748 |  |
| HMC18 | 6.470 |  | 4.705 |  | 4.668 |  |
| Hs_578T | 6.258 |  | 5.229 |  | 6.455 |  |
| HCC1395 | 8.214 | UPPER TERTILE | 2.557 | LOWER TERTILE | 4.249 |  |

| **Cell line** | **TBC1D4** | **TBC1D4 STATUS** | **TBC1D5** | **TBC1D5 STATUS** | **TBC1D7** | **TBC1D7 STATUS** |
| --- | --- | --- | --- | --- | --- | --- |
| HDQP1 | 79.671 | UPPER TERTILE | 52.581 |  | 17.852 |  |
| HCC1937 | 10.903 | LOWER TERTILE | 34.944 | LOWER TERTILE | 27.232 | UPPER TERTILE |
| MDAMB361 | 42.253 |  | 38.895 |  | 13.546 | LOWER TERTILE |
| HCC1143 | 25.434 |  | 60.808 | UPPER TERTILE | 11.256 | LOWER TERTILE |
| HCC1599 | 54.270 | UPPER TERTILE | 53.925 |  | 10.632 | LOWER TERTILE |
| HCC202 | 38.275 |  | 45.212 |  | 20.252 |  |
| BT20 | 14.632 | LOWER TERTILE | 65.810 | UPPER TERTILE | 19.329 |  |
| MDAMB468 | 34.483 |  | 38.516 | LOWER TERTILE | 24.622 | UPPER TERTILE |
| HCC1569 | 68.814 | UPPER TERTILE | 33.455 | LOWER TERTILE | 12.722 | LOWER TERTILE |
| HCC1954 | 40.703 |  | 42.589 |  | 23.524 | UPPER TERTILE |
| HCC1806 | 8.866 | LOWER TERTILE | 46.842 |  | 22.605 | UPPER TERTILE |
| MDAMB134VI | 10.342 | LOWER TERTILE | 18.843 | LOWER TERTILE | 13.286 | LOWER TERTILE |
| HCC1419 | 51.514 |  | 44.065 |  | 21.232 |  |
| HCC1187 | 14.392 | LOWER TERTILE | 30.123 | LOWER TERTILE | 17.423 |  |
| T47D | 14.870 | LOWER TERTILE | 76.304 | UPPER TERTILE | 23.527 | UPPER TERTILE |
| HCC38 | 7.918 | LOWER TERTILE | 94.386 | UPPER TERTILE | 18.971 |  |
| BT483 | 31.016 |  | 37.403 | LOWER TERTILE | 20.578 |  |
| HCC2218 | 86.103 | UPPER TERTILE | 21.150 | LOWER TERTILE | 20.755 |  |
| MDAMB175VII | 7.957 | LOWER TERTILE | 38.679 |  | 8.197 | LOWER TERTILE |
| EFM19 | 22.850 |  | 71.922 | UPPER TERTILE | 16.875 |  |
| CAMA1 | 47.824 |  | 55.727 |  | 29.453 | UPPER TERTILE |
| HCC70 | 66.533 | UPPER TERTILE | 38.791 |  | 19.211 |  |
| UACC893 | 63.416 | UPPER TERTILE | 42.382 |  | 21.965 |  |
| MDAMB415 | 26.259 |  | 9.335 | LOWER TERTILE | 30.045 | UPPER TERTILE |
| HCC1428 | 14.496 | LOWER TERTILE | 41.976 |  | 28.613 | UPPER TERTILE |
| BT549 | 71.411 | UPPER TERTILE | 69.657 | UPPER TERTILE | 13.926 | LOWER TERTILE |
| SKBR3 | 36.098 |  | 29.672 | LOWER TERTILE | 24.499 | UPPER TERTILE |
| CAL851 | 48.313 |  | 33.687 | LOWER TERTILE | 25.468 | UPPER TERTILE |
| CAL148 | 11.924 | LOWER TERTILE | 64.846 | UPPER TERTILE | 27.012 | UPPER TERTILE |
| DU4475 | 52.114 | UPPER TERTILE | 65.796 | UPPER TERTILE | 30.623 | UPPER TERTILE |
| ZR7530 | 56.187 | UPPER TERTILE | 36.612 | LOWER TERTILE | 12.207 | LOWER TERTILE |
| CAL120 | 70.526 | UPPER TERTILE | 98.118 | UPPER TERTILE | 17.251 |  |
| EFM192A | 24.658 |  | 54.103 |  | 21.271 |  |
| CAL51 | 12.145 | LOWER TERTILE | 63.818 | UPPER TERTILE | 13.689 | LOWER TERTILE |
| AU565 | 12.394 | LOWER TERTILE | 43.504 |  | 36.295 | UPPER TERTILE |
| BT474 | 62.828 | UPPER TERTILE | 75.230 | UPPER TERTILE | 17.095 |  |
| ZR751 | 22.767 |  | 31.141 | LOWER TERTILE | 8.012 | LOWER TERTILE |
| MCF7 | 14.256 | LOWER TERTILE | 60.737 | UPPER TERTILE | 26.508 | UPPER TERTILE |
| JIMT1 | 27.899 |  | 122.114 | UPPER TERTILE | 21.305 |  |
| MDAMB231 | 55.888 | UPPER TERTILE | 38.113 | LOWER TERTILE | 10.109 | LOWER TERTILE |
| MDAMB157 | 21.855 | LOWER TERTILE | 42.697 |  | 11.720 | LOWER TERTILE |
| MDAMB453 | 15.395 | LOWER TERTILE | 35.006 | LOWER TERTILE | 17.256 |  |
| UACC812 | 80.935 | UPPER TERTILE | 16.110 | LOWER TERTILE | 6.417 | LOWER TERTILE |
| HMC18 | 43.986 |  | 56.536 |  | 16.711 | LOWER TERTILE |
| Hs_578T | 56.020 | UPPER TERTILE | 103.314 | UPPER TERTILE | 25.125 | UPPER TERTILE |
| HCC1395 | 87.987 | UPPER TERTILE | 91.146 | UPPER TERTILE | 15.362 | LOWER TERTILE |

| **Cell line** | **TBC1D8** | **TBC1D8 STATUS** | **TBC1D8B** | **TBC1D8B STATUS** | **TBC1D9** | **TBC1D9 STATUS** |
| --- | --- | --- | --- | --- | --- | --- |
| HDQP1 | 23.619 | LOWER TERTILE | 7.009 |  | 29.113 |  |
| HCC1937 | 35.240 |  | 0.936 | LOWER TERTILE | 13.579 | LOWER TERTILE |
| MDAMB361 | 71.429 | UPPER TERTILE | 1.555 | LOWER TERTILE | 55.943 | UPPER TERTILE |
| HCC1143 | 116.513 | UPPER TERTILE | 2.263 | LOWER TERTILE | 15.242 | LOWER TERTILE |
| HCC1599 | 135.371 | UPPER TERTILE | 12.572 | UPPER TERTILE | 18.993 | LOWER TERTILE |
| HCC202 | 38.016 |  | 6.478 |  | 28.813 |  |
| BT20 | 12.774 | LOWER TERTILE | 12.658 | UPPER TERTILE | 35.524 |  |
| MDAMB468 | 56.088 | UPPER TERTILE | 2.803 | LOWER TERTILE | 22.996 | LOWER TERTILE |
| HCC1569 | 48.403 |  | 5.078 | LOWER TERTILE | 14.926 | LOWER TERTILE |
| HCC1954 | 39.264 |  | 15.535 | UPPER TERTILE | 25.188 |  |
| HCC1806 | 123.496 | UPPER TERTILE | 8.812 |  | 0.947 | LOWER TERTILE |
| MDAMB134VI | 31.725 |  | 1.958 | LOWER TERTILE | 114.628 | UPPER TERTILE |
| HCC1419 | 68.936 | UPPER TERTILE | 20.255 | UPPER TERTILE | 173.792 | UPPER TERTILE |
| HCC1187 | 5.919 | LOWER TERTILE | 6.308 |  | 33.067 |  |
| T47D | 8.071 | LOWER TERTILE | 4.802 | LOWER TERTILE | 21.485 | LOWER TERTILE |
| HCC38 | 17.268 | LOWER TERTILE | 6.755 |  | 27.979 |  |
| BT483 | 12.252 | LOWER TERTILE | 7.556 |  | 197.287 | UPPER TERTILE |
| HCC2218 | 137.601 | UPPER TERTILE | 11.026 |  | 49.333 |  |
| MDAMB175VII | 72.590 | UPPER TERTILE | 17.290 | UPPER TERTILE | 120.997 | UPPER TERTILE |
| EFM19 | 21.787 | LOWER TERTILE | 8.288 |  | 41.956 |  |
| CAMA1 | 7.784 | LOWER TERTILE | 4.582 | LOWER TERTILE | 50.072 |  |
| HCC70 | 71.706 | UPPER TERTILE | 29.706 | UPPER TERTILE | 57.549 | UPPER TERTILE |
| UACC893 | 85.277 | UPPER TERTILE | 4.962 | LOWER TERTILE | 168.100 | UPPER TERTILE |
| MDAMB415 | 34.604 |  | 17.410 | UPPER TERTILE | 34.510 |  |
| HCC1428 | 17.206 | LOWER TERTILE | 2.089 | LOWER TERTILE | 118.909 | UPPER TERTILE |
| BT549 | 40.758 |  | 7.950 |  | 1.806 | LOWER TERTILE |
| SKBR3 | 28.738 |  | 12.584 | UPPER TERTILE | 71.344 | UPPER TERTILE |
| CAL851 | 29.232 |  | 15.192 | UPPER TERTILE | 13.909 | LOWER TERTILE |
| CAL148 | 43.643 |  | 4.258 | LOWER TERTILE | 41.439 |  |
| DU4475 | 16.396 | LOWER TERTILE | 18.096 | UPPER TERTILE | 2.942 | LOWER TERTILE |
| ZR7530 | 101.860 | UPPER TERTILE | 23.074 | UPPER TERTILE | 48.547 |  |
| CAL120 | 2.567 | LOWER TERTILE | 15.510 | UPPER TERTILE | 42.316 |  |
| EFM192A | 28.766 |  | 10.201 |  | 50.669 | UPPER TERTILE |
| CAL51 | 40.453 |  | 5.381 |  | 23.622 | LOWER TERTILE |
| AU565 | 68.310 | UPPER TERTILE | 3.593 | LOWER TERTILE | 102.445 | UPPER TERTILE |
| BT474 | 44.347 |  | 6.044 |  | 34.890 |  |
| ZR751 | 10.704 | LOWER TERTILE | 15.413 | UPPER TERTILE | 68.350 | UPPER TERTILE |
| MCF7 | 23.897 | LOWER TERTILE | 0.577 | LOWER TERTILE | 277.684 | UPPER TERTILE |
| JIMT1 | 56.015 | UPPER TERTILE | 7.142 |  | 27.173 |  |
| MDAMB231 | 24.869 |  | 6.666 |  | 50.996 | UPPER TERTILE |
| MDAMB157 | 15.884 | LOWER TERTILE | 0.175 | LOWER TERTILE | 19.978 | LOWER TERTILE |
| MDAMB453 | 55.670 | UPPER TERTILE | 2.187 | LOWER TERTILE | 14.056 | LOWER TERTILE |
| UACC812 | 32.876 |  | 9.508 |  | 25.233 |  |
| HMC18 | 24.171 | LOWER TERTILE | 8.039 |  | 18.529 | LOWER TERTILE |
| Hs_578T | 33.358 |  | 32.684 | UPPER TERTILE | 64.147 | UPPER TERTILE |
| HCC1395 | 73.030 | UPPER TERTILE | 13.569 | UPPER TERTILE | 11.837 | LOWER TERTILE |

| **Cell line** | **TBC1D9B** | **TBC1D9B STATUS** | **TBCK** | **TBCK STATUS** | **USP6** | **USP6 STATUS** |
| --- | --- | --- | --- | --- | --- | --- |
| HDQP1 | 178.020 |  | 12.781 | LOWER TERTILE | 1.079 | UPPER TERTILE |
| HCC1937 | 198.963 |  | 16.385 |  | 0.284 | UPPER TERTILE |
| MDAMB361 | 135.447 | LOWER TERTILE | 15.285 |  | 0.036 | LOWER TERTILE |
| HCC1143 | 219.715 | UPPER TERTILE | 9.667 | LOWER TERTILE | 0.331 | UPPER TERTILE |
| HCC1599 | 202.105 |  | 7.972 | LOWER TERTILE | 0.156 |  |
| HCC202 | 187.244 |  | 12.134 | LOWER TERTILE | 0.246 |  |
| BT20 | 221.480 | UPPER TERTILE | 12.318 | LOWER TERTILE | 0.046 | LOWER TERTILE |
| MDAMB468 | 226.545 | UPPER TERTILE | 11.804 | LOWER TERTILE | 0.099 | LOWER TERTILE |
| HCC1569 | 214.694 |  | 12.546 | LOWER TERTILE | 0.172 |  |
| HCC1954 | 155.530 | LOWER TERTILE | 21.891 | UPPER TERTILE | 0.303 | UPPER TERTILE |
| HCC1806 | 86.654 | LOWER TERTILE | 10.998 | LOWER TERTILE | 0.077 | LOWER TERTILE |
| MDAMB134VI | 145.637 | LOWER TERTILE | 36.891 | UPPER TERTILE | 0.581 | UPPER TERTILE |
| HCC1419 | 178.612 |  | 15.521 |  | 0.173 |  |
| HCC1187 | 181.441 |  | 10.948 | LOWER TERTILE | 0.229 |  |
| T47D | 172.883 |  | 19.244 |  | 0.225 |  |
| HCC38 | 145.057 | LOWER TERTILE | 10.703 | LOWER TERTILE | 0.788 | UPPER TERTILE |
| BT483 | 268.386 | UPPER TERTILE | 35.386 | UPPER TERTILE | 0.159 |  |
| HCC2218 | 353.776 | UPPER TERTILE | 26.014 | UPPER TERTILE | 0.366 | UPPER TERTILE |
| MDAMB175VII | 248.169 | UPPER TERTILE | 19.464 |  | 0.113 |  |
| EFM19 | 150.682 | LOWER TERTILE | 19.406 |  | 0.467 | UPPER TERTILE |
| CAMA1 | 398.157 | UPPER TERTILE | 20.429 |  | 0.106 | LOWER TERTILE |
| HCC70 | 158.305 | LOWER TERTILE | 12.717 | LOWER TERTILE | 0.000 | LOWER TERTILE |
| UACC893 | 115.044 | LOWER TERTILE | 33.791 | UPPER TERTILE | 0.125 |  |
| MDAMB415 | 219.162 |  | 12.919 |  | 0.131 |  |
| HCC1428 | 136.749 | LOWER TERTILE | 16.295 |  | 0.109 | LOWER TERTILE |
| BT549 | 213.285 |  | 15.001 |  | 0.315 | UPPER TERTILE |
| SKBR3 | 211.701 |  | 19.904 |  | 0.035 | LOWER TERTILE |
| CAL851 | 137.833 | LOWER TERTILE | 6.943 | LOWER TERTILE | 0.084 | LOWER TERTILE |
| CAL148 | 280.355 | UPPER TERTILE | 35.172 | UPPER TERTILE | 0.130 |  |
| DU4475 | 203.731 |  | 23.551 | UPPER TERTILE | 0.773 | UPPER TERTILE |
| ZR7530 | 220.917 | UPPER TERTILE | 37.262 | UPPER TERTILE | 0.020 | LOWER TERTILE |
| CAL120 | 183.686 |  | 20.887 | UPPER TERTILE | 0.142 |  |
| EFM192A | 333.128 | UPPER TERTILE | 20.503 | UPPER TERTILE | 0.156 |  |
| CAL51 | 142.153 | LOWER TERTILE | 43.626 | UPPER TERTILE | 0.484 | UPPER TERTILE |
| AU565 | 223.292 | UPPER TERTILE | 9.731 | LOWER TERTILE | 0.000 | LOWER TERTILE |
| BT474 | 255.036 | UPPER TERTILE | 19.605 |  | 0.200 |  |
| ZR751 | 125.146 | LOWER TERTILE | 26.746 | UPPER TERTILE | 0.204 |  |
| MCF7 | 275.500 | UPPER TERTILE | 24.398 | UPPER TERTILE | 0.353 | UPPER TERTILE |
| JIMT1 | 352.431 | UPPER TERTILE | 7.169 | LOWER TERTILE | 0.083 | LOWER TERTILE |
| MDAMB231 | 158.819 | LOWER TERTILE | 6.523 | LOWER TERTILE | 0.135 |  |
| MDAMB157 | 171.031 |  | 14.616 |  | 0.433 | UPPER TERTILE |
| MDAMB453 | 199.552 |  | 14.898 |  | 0.071 | LOWER TERTILE |
| UACC812 | 178.480 |  | 22.850 | UPPER TERTILE | 0.077 | LOWER TERTILE |
| HMC18 | 139.908 | LOWER TERTILE | 19.312 |  | 1.766 | UPPER TERTILE |
| Hs_578T | 332.479 | UPPER TERTILE | 14.342 |  | 0.271 | UPPER TERTILE |
| HCC1395 | 87.002 | LOWER TERTILE | 24.184 | UPPER TERTILE | 0.108 | LOWER TERTILE |

| **Cell line** | **USP6NL** | **USP6NL STATUS** |
| --- | --- | --- |
| HDQP1 | 92.011 | UPPER TERTILE |
| HCC1937 | 24.034 | LOWER TERTILE |
| MDAMB361 | 31.651 | LOWER TERTILE |
| HCC1143 | 65.380 | UPPER TERTILE |
| HCC1599 | 44.116 |  |
| HCC202 | 64.847 | UPPER TERTILE |
| BT20 | 29.031 | LOWER TERTILE |
| MDAMB468 | 31.783 | LOWER TERTILE |
| HCC1569 | 58.589 | UPPER TERTILE |
| HCC1954 | 49.657 |  |
| HCC1806 | 31.112 | LOWER TERTILE |
| MDAMB134VI | 15.039 | LOWER TERTILE |
| HCC1419 | 51.852 |  |
| HCC1187 | 95.888 | UPPER TERTILE |
| T47D | 47.348 |  |
| HCC38 | 56.935 | UPPER TERTILE |
| BT483 | 56.744 | UPPER TERTILE |
| HCC2218 | 38.190 |  |
| MDAMB175VII | 97.817 | UPPER TERTILE |
| EFM19 | 63.599 | UPPER TERTILE |
| CAMA1 | 29.803 | LOWER TERTILE |
| HCC70 | 109.931 | UPPER TERTILE |
| UACC893 | 97.742 | UPPER TERTILE |
| MDAMB415 | 68.477 | UPPER TERTILE |
| HCC1428 | 67.972 | UPPER TERTILE |
| BT549 | 64.752 | UPPER TERTILE |
| SKBR3 | 62.581 | UPPER TERTILE |
| CAL851 | 56.715 |  |
| CAL148 | 51.379 |  |
| DU4475 | 50.636 |  |
| ZR7530 | 50.192 |  |
| CAL120 | 48.689 |  |
| EFM192A | 47.419 |  |
| CAL51 | 46.821 |  |
| AU565 | 45.016 |  |
| BT474 | 44.099 |  |
| ZR751 | 42.809 |  |
| MCF7 | 35.167 |  |
| JIMT1 | 34.786 | LOWER TERTILE |
| MDAMB231 | 32.351 | LOWER TERTILE |
| MDAMB157 | 31.953 | LOWER TERTILE |
| MDAMB453 | 27.661 | LOWER TERTILE |
| UACC812 | 26.095 | LOWER TERTILE |
| HMC18 | 23.143 | LOWER TERTILE |
| Hs_578T | 17.531 | LOWER TERTILE |
| HCC1395 | 12.753 | LOWER TERTILE |

**Supplementary Table 7. Metabolite concentrations in various BC lines, expressed as the FC between the upper and lower tertiles for each TBC1 protein**

| **Metabolite** | **EVI5 FC** | **EVI5 P VALUE** | **EVI5L FC** | **EVI5L P VALUE** | **GRTP1 FC** | **GRTP1 P VALUE** | **RABGAP1 FC** | **RABGAP1 P VALUE** |
| --- | --- | --- | --- | --- | --- | --- | --- | --- |
| 1-methylnicotinamide | 2.720 | 0.443 | 0.985 | 0.756 | 0.057 | 0.011 | 0.604 | 0.567 |
| alpha-glycerophosphate | 1.712 | 0.040 | 1.127 | 0.494 | 2.243 | 0.024 | 0.887 | 0.768 |
| malonylcarnitine | 0.775 | 0.694 | 0.770 | 0.221 | 1.987 | 0.036 | 1.043 | 0.640 |
| erythrose-4-phosphate | 1.377 | 0.082 | 1.086 | 0.663 | 1.149 | 0.254 | 0.961 | 0.931 |
| N-carbamoyl-beta-alanine | 1.627 | 0.290 | 1.253 | 0.820 | 0.529 | 0.065 | 0.711 | 0.323 |
| hexanoylcarnitine | 0.754 | 0.165 | 0.862 | 0.419 | 2.265 | 0.029 | 0.977 | 0.849 |
| beta-alanine | 0.877 | 0.663 | 0.918 | 0.576 | 0.693 | 0.165 | 1.353 | 0.358 |
| 3-phosphoglycerate | 1.464 | 0.237 | 1.186 | 0.756 | 1.603 | 0.029 | 1.495 | 0.206 |
| inositol | 1.267 | 0.237 | 0.664 | 0.130 | 0.557 | 0.003 | 0.949 | 0.435 |
| phenylalanine | 1.516 | 0.191 | 1.705 | 0.130 | 1.196 | 0.724 | 1.229 | 0.358 |
| GABA | 0.488 | 0.310 | 0.731 | 0.520 | 0.431 | 0.101 | 0.858 | 0.544 |
| acetylcarnitine | 0.851 | 0.885 | 1.094 | 0.788 | 1.886 | 0.078 | 1.113 | 0.742 |
| butyrylcarnitine/isobutyrylcarnitine | 0.403 | 0.049 | 0.751 | 0.419 | 2.591 | 0.085 | 2.063 | 0.080 |
| myristoylcarnitine | 0.844 | 0.330 | 0.435 | 0.165 | 1.684 | 0.351 | 1.442 | 0.456 |
| 2-aminoadipate | 1.109 | 0.548 | 0.834 | 0.330 | 0.695 | 0.065 | 0.780 | 0.141 |
| DHAP/glyceraldehyde 3P | 1.772 | 0.093 | 1.512 | 0.330 | 1.476 | 0.221 | 1.060 | 0.849 |
| alanine | 0.978 | 0.663 | 1.352 | 0.548 | 1.898 | 0.027 | 1.756 | 0.021 |
| serine | 1.222 | 0.351 | 1.312 | 0.520 | 1.462 | 0.548 | 1.120 | 0.849 |
| glutamate | 1.021 | 0.885 | 1.233 | 0.520 | 0.682 | 0.024 | 0.766 | 0.232 |
| valine | 1.425 | 0.272 | 1.549 | 0.141 | 1.044 | 0.694 | 1.150 | 0.615 |
| lauroylcarnitine | 0.884 | 0.419 | 0.658 | 0.443 | 2.289 | 0.110 | 1.115 | 0.876 |
| cytidine | 0.614 | 0.330 | 0.687 | 0.443 | 1.055 | 0.756 | 1.707 | 0.232 |
| GMP | 1.039 | 0.950 | 1.049 | 0.917 | 1.722 | 0.059 | 1.361 | 0.115 |
| kynurenine | 0.924 | 0.663 | 0.998 | 0.852 | 0.538 | 0.101 | 1.095 | 0.742 |
| lactate | 1.304 | 0.468 | 1.242 | 0.494 | 1.555 | 0.120 | 1.301 | 0.716 |
| alpha-hydroxybutyrate | 0.778 | 0.152 | 0.834 | 0.419 | 0.631 | 0.004 | 0.783 | 0.054 |
| asparagine | 0.693 | 0.663 | 0.507 | 0.237 | 1.562 | 0.237 | 1.258 | 0.795 |
| lysine | 1.644 | 0.272 | 1.577 | 0.221 | 0.977 | 0.885 | 1.059 | 0.986 |
| tryptophan | 1.233 | 0.468 | 1.864 | 0.059 | 1.274 | 0.604 | 1.267 | 0.323 |
| 2-deoxycytidine | 0.668 | 0.604 | 0.703 | 0.604 | 1.918 | 0.110 | 1.322 | 0.499 |
| palmitoylcarnitine | 0.858 | 0.468 | 0.573 | 0.330 | 1.234 | 0.756 | 1.088 | 0.903 |
| guanosine | 1.080 | 0.724 | 1.012 | 0.694 | 0.623 | 0.141 | 0.948 | 0.986 |
| inosine | 0.896 | 0.663 | 1.050 | 0.852 | 0.918 | 0.468 | 0.655 | 0.086 |
| ribose-5-P/ribulose5-P | 1.339 | 0.330 | 1.486 | 0.443 | 2.129 | 0.029 | 1.096 | 0.795 |
| sorbitol | 1.307 | 0.663 | 1.090 | 0.885 | 1.212 | 0.950 | 1.273 | 0.795 |
| 2-hydroxyglutarate | 1.032 | 0.604 | 1.048 | 0.443 | 0.662 | 0.330 | 1.276 | 0.435 |
| threonine | 1.295 | 0.351 | 1.682 | 0.101 | 1.145 | 0.663 | 1.190 | 0.499 |
| isoleucine | 1.352 | 0.395 | 1.334 | 0.310 | 1.145 | 0.852 | 1.174 | 0.456 |
| 5-HIAA | 0.886 | 0.520 | 0.975 | 0.917 | 0.733 | 0.130 | 1.104 | 0.615 |
| trimethylamine-N-oxide | 0.844 | 0.254 | 0.960 | 0.917 | 0.668 | 0.019 | 0.963 | 0.822 |
| butyrobetaine | 0.721 | 0.237 | 1.190 | 0.724 | 1.530 | 0.141 | 1.148 | 0.521 |
| stearoylcarnitine | 1.213 | 0.852 | 0.700 | 0.633 | 0.955 | 0.788 | 1.139 | 0.742 |
| F1P/F6P/G1P/G6P | 1.677 | 0.024 | 1.258 | 0.290 | 1.467 | 0.059 | 0.798 | 0.456 |
| hexoses (HILIC neg) | 1.845 | 0.071 | 1.363 | 0.221 | 1.134 | 0.756 | 1.043 | 0.591 |
| hypoxanthine | 1.537 | 0.330 | 0.663 | 0.373 | 0.923 | 0.950 | 0.471 | 0.100 |
| NAD | 1.017 | 0.917 | 0.988 | 0.885 | 1.070 | 0.756 | 1.031 | 0.795 |
| NADP | 1.632 | 0.141 | 0.968 | 0.694 | 1.630 | 0.373 | 1.659 | 0.100 |
| methionine | 1.448 | 0.221 | 1.442 | 0.165 | 1.279 | 0.788 | 1.209 | 0.340 |
| glutamine | 1.444 | 0.443 | 1.446 | 0.272 | 1.694 | 0.290 | 1.042 | 0.849 |
| leucine | 1.317 | 0.330 | 1.364 | 0.351 | 1.150 | 0.820 | 1.086 | 0.822 |
| tyrosine | 1.356 | 0.310 | 1.397 | 0.206 | 1.075 | 0.950 | 1.194 | 0.396 |
| thiamine | 1.229 | 0.520 | 1.745 | 0.130 | 1.261 | 0.788 | 1.051 | 0.591 |
| niacinamide | 1.656 | 0.120 | 1.864 | 0.101 | 1.348 | 0.443 | 1.240 | 0.567 |
| alpha-glycerophosphocholine | 0.546 | 0.221 | 0.869 | 0.820 | 0.795 | 0.984 | 0.918 | 0.822 |
| thymidine | 0.954 | 0.852 | 1.413 | 0.468 | 1.135 | 0.663 | 1.095 | 0.876 |
| carnitine | 0.796 | 0.419 | 1.195 | 0.917 | 1.516 | 0.165 | 1.141 | 0.567 |
| oleylcarnitine | 0.799 | 0.254 | 0.355 | 0.059 | 0.836 | 0.820 | 1.150 | 0.768 |
| aconitate | 1.268 | 0.395 | 0.979 | 0.984 | 1.525 | 0.078 | 1.238 | 0.115 |
| CMP | 0.764 | 0.272 | 0.894 | 0.724 | 0.879 | 0.663 | 1.087 | 0.567 |
| cystathionine | 0.806 | 0.756 | 2.169 | 0.130 | 0.889 | 0.468 | 1.290 | 0.640 |
| fumarate/maleate/alpha-ketoisovalerate | 0.767 | 0.310 | 0.955 | 0.633 | 1.078 | 0.984 | 1.183 | 0.290 |
| glutathione oxidized | 1.160 | 0.373 | 0.735 | 0.468 | 1.097 | 0.576 | 1.146 | 0.665 |
| hippurate | 1.697 | 0.120 | 1.047 | 0.663 | 1.318 | 0.604 | 1.008 | 0.931 |
| pantothenate | 1.605 | 0.165 | 1.550 | 0.130 | 1.906 | 0.071 | 1.263 | 0.544 |
| sucrose | 1.210 | 0.237 | 0.748 | 0.395 | 0.793 | 0.191 | 0.510 | 0.007 |
| UMP | 0.758 | 0.178 | 1.171 | 0.520 | 1.464 | 0.040 | 1.105 | 0.477 |
| UDP-galactose/UDP-glucose | 1.187 | 0.604 | 0.905 | 0.820 | 0.737 | 0.548 | 0.882 | 0.876 |
| 6-phosphogluconate | 2.089 | 0.165 | 1.254 | 0.852 | 3.007 | 0.014 | 1.647 | 0.115 |
| histidine | 1.286 | 0.917 | 1.175 | 0.633 | 0.981 | 0.419 | 1.126 | 0.903 |
| proline | 1.011 | 0.788 | 1.054 | 0.950 | 1.410 | 0.221 | 1.291 | 0.206 |
| cis/trans-hydroxyproline | 0.770 | 0.576 | 0.721 | 0.520 | 1.817 | 0.059 | 0.946 | 0.690 |
| homocysteine | 0.952 | 0.852 | 1.387 | 0.110 | 1.295 | 0.494 | 1.680 | 0.054 |
| SDMA/ADMA | 1.134 | 0.984 | 1.074 | 0.756 | 1.387 | 0.520 | 1.178 | 0.358 |
| NMMA | 1.108 | 0.852 | 1.212 | 0.520 | 1.373 | 0.330 | 1.234 | 0.377 |
| carnosine | 0.699 | 0.351 | 0.832 | 0.520 | 0.904 | 0.520 | 1.145 | 0.876 |
| adenosine | 1.805 | 0.101 | 2.001 | 0.049 | 1.839 | 0.191 | 0.888 | 0.903 |
| 2-deoxyadenosine | 1.310 | 0.272 | 0.888 | 0.663 | 1.927 | 0.120 | 1.445 | 0.377 |
| cotinine | 0.841 | 0.237 | 1.066 | 0.984 | 1.171 | 0.395 | 0.970 | 0.690 |
| pipecolic acid | 1.084 | 0.820 | 0.595 | 0.290 | 0.616 | 0.206 | 0.676 | 0.340 |
| pyroglutamic acid | 1.735 | 0.085 | 1.422 | 0.373 | 1.369 | 0.917 | 1.286 | 0.876 |
| sarcosine | 0.832 | 0.694 | 1.103 | 0.820 | 1.737 | 0.010 | 1.661 | 0.009 |
| isocitrate | 1.264 | 0.520 | 0.896 | 0.788 | 1.227 | 0.373 | 0.942 | 0.396 |
| dCMP | 1.049 | 0.852 | 1.058 | 0.520 | 1.015 | 0.724 | 0.892 | 0.742 |
| glutathione reduced | 0.838 | 0.395 | 0.780 | 0.395 | 0.952 | 0.820 | 1.150 | 0.591 |
| lactose | 1.814 | 0.015 | 0.662 | 0.604 | 1.029 | 0.950 | 0.773 | 0.567 |
| oxalate | 0.946 | 0.576 | 1.073 | 0.694 | 0.888 | 0.373 | 0.995 | 0.876 |
| thymine | 0.893 | 0.520 | 0.817 | 0.633 | 1.051 | 0.756 | 0.974 | 0.690 |
| taurodeoxycholate/taurochenodeoxycholate | 2.404 | 0.178 | 1.848 | 0.724 | 2.007 | 0.290 | 0.600 | 0.477 |
| malondialdehyde | 0.953 | 0.419 | 1.107 | 0.534 | 1.032 | 0.494 | 1.077 | 0.849 |
| ornithine | 1.081 | 0.885 | 0.755 | 0.395 | 1.162 | 0.885 | 1.017 | 0.931 |
| citrulline | 1.603 | 0.165 | 1.092 | 0.756 | 1.249 | 0.663 | 1.072 | 0.768 |
| taurine | 0.565 | 0.237 | 0.772 | 0.395 | 1.071 | 0.984 | 1.322 | 0.456 |
| acetylglycine | 0.711 | 0.120 | 0.719 | 0.085 | 1.374 | 0.237 | 1.567 | 0.043 |
| allantoin | 1.315 | 0.604 | 1.215 | 0.724 | 1.424 | 0.351 | 1.257 | 0.340 |
| betaine | 0.815 | 0.351 | 1.328 | 0.254 | 1.219 | 0.604 | 0.957 | 0.716 |
| choline | 1.199 | 0.576 | 1.298 | 0.373 | 0.910 | 0.576 | 0.915 | 0.591 |
| acetylcholine | 1.617 | 0.576 | 3.269 | 0.101 | 2.469 | 0.130 | 1.985 | 0.323 |
| xanthosine | 1.045 | 0.820 | 1.310 | 0.576 | 1.524 | 0.310 | 1.283 | 0.396 |
| methionine sulfoxide | 1.448 | 0.120 | 1.449 | 0.093 | 1.308 | 0.395 | 1.121 | 0.640 |
| valerylcarnitine/isovalerylcarnitine/2-methylbutyroylcarnitine | 0.855 | 0.852 | 0.864 | 0.520 | 1.633 | 0.310 | 1.917 | 0.093 |
| heptanoylcarnitine | 0.891 | 0.756 | 1.079 | 0.917 | 1.175 | 0.852 | 1.202 | 0.115 |
| anserine | 0.962 | 0.756 | 0.799 | 0.494 | 0.841 | 0.443 | 1.094 | 0.640 |
| adenine | 0.792 | 0.663 | 0.667 | 0.191 | 0.539 | 0.078 | 0.856 | 0.849 |
| adipate | 1.005 | 0.852 | 0.970 | 0.468 | 0.854 | 0.093 | 0.982 | 0.959 |
| alpha-ketoglutarate | 1.115 | 0.663 | 0.905 | 0.604 | 0.927 | 0.694 | 1.124 | 0.742 |
| AMP | 0.602 | 0.065 | 0.878 | 0.633 | 1.519 | 0.078 | 1.197 | 0.171 |
| citrate | 1.278 | 0.443 | 0.920 | 0.633 | 1.365 | 0.110 | 0.871 | 0.716 |
| glucuronate | 1.127 | 0.373 | 1.126 | 0.852 | 1.071 | 0.820 | 0.979 | 0.665 |
| malate | 0.918 | 0.820 | 0.880 | 0.548 | 1.020 | 0.820 | 1.091 | 0.435 |
| PEP | 1.204 | 0.419 | 1.120 | 0.520 | 1.382 | 0.059 | 1.356 | 0.131 |
| succinate/methylmalonate | 1.391 | 0.120 | 0.880 | 0.604 | 1.151 | 0.724 | 1.012 | 0.849 |
| uracil | 1.227 | 0.820 | 0.882 | 0.468 | 1.436 | 0.330 | 1.071 | 0.768 |
| urate | 1.631 | 0.237 | 0.607 | 0.221 | 0.883 | 0.852 | 1.131 | 0.499 |
| xanthine | 1.129 | 0.694 | 0.843 | 0.520 | 1.320 | 0.548 | 1.010 | 0.716 |
| phosphocreatine | 0.663 | 0.419 | 0.455 | 0.044 | 1.203 | 0.633 | 1.180 | 0.396 |
| glycine | 1.004 | 0.852 | 0.925 | 0.373 | 0.962 | 0.852 | 1.037 | 0.903 |
| aspartate | 0.901 | 0.520 | 0.754 | 0.373 | 0.913 | 0.663 | 0.941 | 0.690 |
| serotonin | 1.387 | 0.694 | 0.448 | 0.237 | 0.997 | 0.788 | 1.051 | 0.521 |
| dimethylglycine | 1.271 | 0.494 | 1.093 | 0.885 | 1.277 | 0.604 | 1.138 | 0.477 |
| kynurenic acid | 1.568 | 0.254 | 0.941 | 0.984 | 1.073 | 0.852 | 1.334 | 0.435 |
| 5-adenosylhomocysteine | 0.723 | 0.494 | 1.075 | 0.694 | 1.148 | 0.663 | 1.411 | 0.107 |
| creatine | 0.760 | 0.351 | 0.944 | 0.885 | 1.056 | 0.663 | 1.434 | 0.206 |
| creatinine | 1.035 | 0.756 | 0.927 | 0.310 | 1.127 | 0.694 | 1.087 | 0.435 |
| thyroxine | 1.235 | 0.351 | 0.829 | 0.494 | 1.242 | 0.206 | 1.064 | 0.742 |
| cAMP | 1.010 | 0.984 | 1.165 | 0.633 | 1.033 | 0.820 | 1.327 | 0.290 |
| putrescine | 0.879 | 0.443 | 0.830 | 0.310 | 1.152 | 0.663 | 1.323 | 0.323 |
| arachidonyl_carnitine | 0.827 | 0.206 | 0.871 | 0.443 | 1.137 | 0.917 | 1.207 | 0.435 |
| 4-pyridoxate | 1.203 | 0.272 | 1.059 | 0.852 | 1.606 | 0.130 | 1.205 | 0.665 |
| uridine | 0.674 | 0.272 | 1.119 | 0.788 | 1.171 | 0.724 | 1.393 | 0.435 |
| taurocholate | 1.475 | 0.130 | 1.539 | 0.141 | 1.087 | 0.885 | 1.037 | 0.849 |
| glycodeoxycholate/glycochenodeoxycholate | 1.215 | 0.443 | 0.812 | 0.373 | 1.053 | 0.756 | 0.982 | 0.959 |
| 3-methyladipate/pimelate | 0.921 | 0.709 | 0.927 | 0.576 | 0.847 | 0.206 | 0.906 | 0.376 |
| arginine | 1.179 | 0.724 | 0.921 | 0.724 | 1.327 | 0.330 | 1.258 | 0.306 |
| anthranilic acid | 0.888 | 0.788 | 0.623 | 0.141 | 0.889 | 0.852 | 1.094 | 0.986 |
| hexoses (HILIC pos) | 1.752 | 0.165 | 1.196 | 0.520 | 1.116 | 0.852 | 0.861 | 0.260 |
| propionylcarnitine | 0.766 | 0.604 | 1.119 | 0.885 | 1.359 | 0.443 | 1.818 | 0.115 |

| **Metabolite** | **RABGAP1L FC** | **RABGAP1L P VALUE** | **SGSM1 FC** | **SGSM1 P VALUE** | **SGSM2 FC** | **SGSM2 P VALUE** | **SGSM3 FC** | **SGSM3 P VALUE** |
| --- | --- | --- | --- | --- | --- | --- | --- | --- |
| 1-methylnicotinamide | 1.234 | 0.443 | 0.276 | 0.290 | 0.115 | 0.054 | 0.161 | 0.152 |
| alpha-glycerophosphate | 0.967 | 0.820 | 1.105 | 0.494 | 1.369 | 0.724 | 2.558 | 0.015 |
| malonylcarnitine | 1.208 | 0.351 | 2.374 | 0.029 | 1.468 | 0.221 | 1.586 | 0.093 |
| erythrose-4-phosphate | 1.360 | 0.082 | 1.192 | 0.089 | 0.861 | 0.290 | 1.494 | 0.054 |
| N-carbamoyl-beta-alanine | 1.616 | 0.520 | 0.644 | 0.272 | 0.502 | 0.290 | 0.613 | 0.443 |
| hexanoylcarnitine | 0.674 | 0.520 | 1.785 | 0.272 | 1.697 | 0.178 | 1.533 | 0.191 |
| beta-alanine | 1.143 | 0.885 | 1.097 | 0.950 | 0.718 | 0.419 | 0.598 | 0.040 |
| 3-phosphoglycerate | 1.807 | 0.085 | 0.925 | 0.788 | 0.531 | 0.049 | 0.851 | 0.984 |
| inositol | 1.323 | 0.191 | 0.769 | 0.351 | 0.886 | 0.633 | 0.913 | 0.576 |
| phenylalanine | 1.249 | 0.310 | 1.012 | 0.724 | 0.930 | 0.885 | 1.147 | 0.756 |
| GABA | 1.335 | 0.852 | 0.964 | 0.950 | 0.612 | 0.756 | 0.254 | 0.033 |
| acetylcarnitine | 0.896 | 0.852 | 1.386 | 0.604 | 1.651 | 0.165 | 1.111 | 0.576 |
| butyrylcarnitine/isobutyrylcarnitine | 1.065 | 0.576 | 1.219 | 0.576 | 0.924 | 0.820 | 0.681 | 0.694 |
| myristoylcarnitine | 1.250 | 0.820 | 3.569 | 0.012 | 2.103 | 0.130 | 2.187 | 0.152 |
| 2-aminoadipate | 0.953 | 0.724 | 0.753 | 0.191 | 0.963 | 0.950 | 0.891 | 0.633 |
| DHAP/glyceraldehyde 3P | 1.173 | 0.694 | 0.982 | 0.917 | 0.615 | 0.110 | 1.147 | 0.663 |
| alanine | 1.871 | 0.021 | 2.066 | 0.010 | 0.969 | 0.548 | 1.387 | 0.152 |
| serine | 1.008 | 0.604 | 1.524 | 0.165 | 1.259 | 0.272 | 1.602 | 0.014 |
| glutamate | 0.860 | 0.604 | 0.586 | 0.007 | 0.866 | 0.917 | 0.792 | 0.468 |
| valine | 1.165 | 0.310 | 0.852 | 0.633 | 0.920 | 0.984 | 1.138 | 0.520 |
| lauroylcarnitine | 0.861 | 0.820 | 2.558 | 0.054 | 1.633 | 0.330 | 2.100 | 0.120 |
| cytidine | 0.497 | 0.178 | 0.866 | 0.984 | 1.616 | 0.443 | 0.918 | 0.950 |
| GMP | 1.439 | 0.071 | 1.509 | 0.059 | 1.199 | 0.756 | 1.589 | 0.040 |
| kynurenine | 0.862 | 0.694 | 0.918 | 0.788 | 0.819 | 0.520 | 0.718 | 0.373 |
| lactate | 1.341 | 0.272 | 1.273 | 0.548 | 0.607 | 0.071 | 0.892 | 0.984 |
| alpha-hydroxybutyrate | 0.797 | 0.120 | 0.823 | 0.395 | 1.055 | 0.443 | 0.831 | 0.663 |
| asparagine | 0.824 | 0.820 | 2.403 | 0.078 | 1.614 | 0.330 | 1.466 | 0.024 |
| lysine | 1.017 | 0.852 | 0.799 | 0.520 | 1.003 | 0.984 | 1.290 | 0.494 |
| tryptophan | 1.248 | 0.272 | 1.111 | 0.950 | 0.950 | 0.724 | 1.009 | 0.852 |
| 2-deoxycytidine | 0.640 | 0.520 | 1.203 | 0.820 | 1.758 | 0.272 | 0.922 | 0.604 |
| palmitoylcarnitine | 1.049 | 0.950 | 4.024 | 0.021 | 2.421 | 0.085 | 2.692 | 0.071 |
| guanosine | 0.747 | 0.468 | 0.619 | 0.254 | 1.151 | 0.576 | 0.862 | 0.984 |
| inosine | 0.805 | 0.351 | 0.880 | 0.395 | 1.736 | 0.024 | 0.966 | 0.724 |
| ribose-5-P/ribulose5-P | 1.648 | 0.206 | 0.952 | 0.820 | 0.641 | 0.141 | 1.364 | 0.206 |
| sorbitol | 1.030 | 0.852 | 0.983 | 0.885 | 0.805 | 0.663 | 0.885 | 0.254 |
| 2-hydroxyglutarate | 0.969 | 0.788 | 0.973 | 0.917 | 0.562 | 0.027 | 0.781 | 0.694 |
| threonine | 1.180 | 0.351 | 0.914 | 0.576 | 1.114 | 0.633 | 1.249 | 0.237 |
| isoleucine | 1.178 | 0.373 | 1.105 | 0.950 | 0.922 | 0.633 | 1.086 | 0.950 |
| 5-HIAA | 0.876 | 0.395 | 0.902 | 0.724 | 0.846 | 0.520 | 0.667 | 0.044 |
| trimethylamine-N-oxide | 0.805 | 0.078 | 0.837 | 0.290 | 0.884 | 0.604 | 0.754 | 0.017 |
| butyrobetaine | 0.910 | 0.694 | 1.284 | 0.788 | 1.484 | 0.272 | 0.826 | 0.395 |
| stearoylcarnitine | 0.811 | 0.852 | 3.714 | 0.015 | 1.750 | 0.520 | 1.941 | 0.351 |
| F1P/F6P/G1P/G6P | 1.215 | 0.272 | 1.000 | 0.548 | 0.905 | 0.373 | 1.914 | 0.005 |
| hexoses (HILIC neg) | 1.090 | 0.663 | 0.857 | 0.663 | 0.835 | 0.576 | 1.155 | 0.419 |
| hypoxanthine | 0.302 | 0.024 | 1.731 | 0.178 | 2.348 | 0.078 | 1.818 | 0.191 |
| NAD | 1.250 | 0.351 | 0.800 | 0.395 | 0.796 | 0.178 | 1.128 | 0.852 |
| NADP | 1.965 | 0.078 | 1.472 | 0.130 | 0.568 | 0.036 | 1.233 | 0.633 |
| methionine | 1.238 | 0.237 | 1.222 | 0.576 | 1.028 | 0.724 | 1.257 | 0.468 |
| glutamine | 0.939 | 0.633 | 1.064 | 0.694 | 1.100 | 0.576 | 1.318 | 0.663 |
| leucine | 1.098 | 0.494 | 1.043 | 0.724 | 0.935 | 0.633 | 1.123 | 0.917 |
| tyrosine | 1.224 | 0.310 | 1.006 | 0.788 | 0.949 | 0.633 | 1.166 | 0.520 |
| thiamine | 1.236 | 0.395 | 0.922 | 0.756 | 0.809 | 0.178 | 0.860 | 0.373 |
| niacinamide | 1.437 | 0.191 | 0.978 | 0.917 | 0.774 | 0.604 | 1.079 | 0.633 |
| alpha-glycerophosphocholine | 0.765 | 0.950 | 0.472 | 0.165 | 0.731 | 0.663 | 0.506 | 0.330 |
| thymidine | 0.513 | 0.085 | 0.744 | 0.395 | 1.218 | 0.520 | 0.711 | 0.443 |
| carnitine | 1.013 | 0.950 | 1.178 | 0.984 | 1.326 | 0.373 | 0.944 | 0.724 |
| oleylcarnitine | 0.951 | 0.984 | 2.974 | 0.033 | 2.295 | 0.141 | 1.571 | 0.494 |
| aconitate | 1.251 | 0.395 | 1.036 | 0.950 | 0.914 | 0.330 | 1.062 | 0.984 |
| CMP | 0.936 | 0.694 | 0.926 | 0.604 | 1.210 | 0.443 | 1.000 | 0.950 |
| cystathionine | 0.465 | 0.152 | 0.478 | 0.085 | 1.219 | 0.724 | 0.659 | 0.330 |
| fumarate/maleate/alpha-ketoisovalerate | 1.117 | 0.633 | 0.970 | 0.395 | 0.867 | 0.165 | 0.822 | 0.419 |
| glutathione oxidized | 1.302 | 0.373 | 0.877 | 0.756 | 0.670 | 0.254 | 0.895 | 0.756 |
| hippurate | 0.849 | 0.724 | 1.069 | 0.694 | 1.147 | 0.576 | 1.318 | 0.520 |
| pantothenate | 1.362 | 0.165 | 1.178 | 0.633 | 0.639 | 0.093 | 1.206 | 0.604 |
| sucrose | 0.756 | 0.633 | 0.799 | 0.237 | 1.213 | 0.724 | 1.727 | 0.101 |
| UMP | 1.769 | 0.008 | 1.114 | 0.950 | 1.335 | 0.178 | 1.248 | 0.330 |
| UDP-galactose/UDP-glucose | 1.351 | 0.178 | 0.550 | 0.110 | 0.796 | 0.110 | 1.076 | 0.724 |
| 6-phosphogluconate | 1.384 | 0.520 | 1.203 | 0.395 | 0.409 | 0.024 | 1.208 | 0.885 |
| histidine | 1.018 | 0.576 | 1.087 | 0.950 | 1.174 | 0.520 | 1.281 | 0.373 |
| proline | 1.167 | 0.272 | 1.354 | 0.373 | 0.863 | 0.141 | 0.859 | 0.237 |
| cis/trans-hydroxyproline | 0.836 | 0.984 | 1.650 | 0.093 | 1.066 | 0.604 | 1.018 | 0.054 |
| homocysteine | 1.516 | 0.290 | 0.715 | 0.351 | 0.658 | 0.206 | 0.630 | 0.130 |
| SDMA/ADMA | 0.956 | 0.820 | 1.277 | 0.221 | 1.096 | 0.576 | 1.104 | 0.820 |
| NMMA | 1.130 | 0.663 | 1.331 | 0.272 | 1.186 | 0.395 | 1.275 | 0.330 |
| carnosine | 1.664 | 0.049 | 1.263 | 0.373 | 0.917 | 0.885 | 0.667 | 0.373 |
| adenosine | 2.236 | 0.110 | 0.959 | 0.852 | 0.873 | 0.756 | 1.677 | 0.237 |
| 2-deoxyadenosine | 1.077 | 0.852 | 1.003 | 0.984 | 0.891 | 0.724 | 1.126 | 0.663 |
| cotinine | 1.045 | 0.820 | 0.709 | 0.178 | 0.828 | 0.633 | 0.667 | 0.165 |
| pipecolic acid | 1.177 | 0.310 | 0.535 | 0.044 | 1.206 | 0.494 | 0.773 | 0.351 |
| pyroglutamic acid | 1.204 | 0.351 | 1.090 | 0.820 | 0.683 | 0.373 | 1.203 | 0.310 |
| sarcosine | 0.935 | 0.852 | 1.310 | 0.120 | 0.844 | 0.443 | 1.147 | 0.604 |
| isocitrate | 0.990 | 0.917 | 0.814 | 0.548 | 0.890 | 0.221 | 1.117 | 0.788 |
| dCMP | 0.823 | 0.694 | 0.722 | 0.141 | 0.958 | 0.917 | 0.682 | 0.101 |
| glutathione reduced | 1.185 | 0.548 | 0.681 | 0.093 | 0.689 | 0.237 | 0.776 | 0.395 |
| lactose | 0.596 | 0.373 | 1.023 | 0.950 | 0.943 | 0.373 | 1.852 | 0.272 |
| oxalate | 0.975 | 0.724 | 0.935 | 0.820 | 0.731 | 0.085 | 0.773 | 0.120 |
| thymine | 1.030 | 0.984 | 1.175 | 0.852 | 0.903 | 0.756 | 0.977 | 0.694 |
| taurodeoxycholate/taurochenodeoxycholate | 1.038 | 0.576 | 1.593 | 0.141 | 0.785 | 0.788 | 1.410 | 0.191 |
| malondialdehyde | 1.360 | 0.237 | 1.087 | 0.633 | 0.733 | 0.147 | 0.774 | 0.254 |
| ornithine | 0.696 | 0.165 | 1.652 | 0.237 | 1.265 | 0.633 | 1.126 | 0.576 |
| citrulline | 0.891 | 0.756 | 1.284 | 0.152 | 1.320 | 0.141 | 1.305 | 0.373 |
| taurine | 0.912 | 0.633 | 1.131 | 0.917 | 1.085 | 0.984 | 0.625 | 0.191 |
| acetylglycine | 1.227 | 0.237 | 1.502 | 0.120 | 0.864 | 0.950 | 1.047 | 0.694 |
| allantoin | 1.051 | 0.395 | 1.255 | 0.152 | 0.922 | 0.694 | 1.082 | 0.604 |
| betaine | 1.024 | 0.788 | 1.355 | 0.206 | 1.220 | 0.290 | 0.967 | 0.443 |
| choline | 1.132 | 0.272 | 1.072 | 0.788 | 1.201 | 0.351 | 1.037 | 0.917 |
| acetylcholine | 2.644 | 0.191 | 1.463 | 0.663 | 0.895 | 0.917 | 0.578 | 0.548 |
| xanthosine | 1.225 | 0.178 | 1.542 | 0.206 | 1.027 | 0.663 | 0.960 | 0.852 |
| methionine sulfoxide | 1.412 | 0.110 | 1.209 | 0.443 | 0.744 | 0.130 | 1.188 | 0.820 |
| valerylcarnitine/isovalerylcarnitine/2-methylbutyroylcarnitine | 0.792 | 0.724 | 0.896 | 0.633 | 1.525 | 0.494 | 1.177 | 0.694 |
| heptanoylcarnitine | 0.902 | 0.852 | 1.559 | 0.110 | 1.372 | 0.330 | 1.370 | 0.419 |
| anserine | 1.320 | 0.130 | 1.005 | 0.694 | 0.949 | 0.852 | 0.788 | 0.330 |
| adenine | 0.707 | 0.191 | 0.975 | 0.395 | 0.908 | 0.290 | 0.709 | 0.141 |
| adipate | 1.001 | 0.852 | 1.016 | 0.694 | 0.837 | 0.059 | 0.890 | 0.330 |
| alpha-ketoglutarate | 1.085 | 0.788 | 0.814 | 0.165 | 0.813 | 0.373 | 0.962 | 0.950 |
| AMP | 1.427 | 0.054 | 1.414 | 0.093 | 0.931 | 0.756 | 1.000 | 0.917 |
| citrate | 1.024 | 0.604 | 0.847 | 0.633 | 1.085 | 0.788 | 1.175 | 0.756 |
| glucuronate | 0.896 | 0.272 | 1.137 | 0.419 | 1.210 | 0.130 | 0.863 | 0.694 |
| malate | 1.243 | 0.330 | 0.896 | 0.520 | 0.949 | 0.310 | 0.948 | 0.788 |
| PEP | 1.394 | 0.165 | 1.042 | 0.604 | 0.715 | 0.272 | 1.057 | 0.633 |
| succinate/methylmalonate | 1.077 | 0.984 | 1.239 | 0.221 | 1.183 | 0.191 | 1.108 | 0.548 |
| uracil | 0.998 | 0.885 | 1.275 | 0.272 | 1.256 | 0.254 | 1.279 | 0.351 |
| urate | 0.771 | 0.576 | 1.314 | 0.310 | 0.772 | 0.917 | 0.985 | 0.724 |
| xanthine | 0.903 | 0.788 | 1.222 | 0.221 | 1.305 | 0.221 | 1.116 | 0.548 |
| phosphocreatine | 1.127 | 0.520 | 0.894 | 0.917 | 1.203 | 0.820 | 1.224 | 0.852 |
| glycine | 1.257 | 0.443 | 0.883 | 0.419 | 0.738 | 0.351 | 1.130 | 0.272 |
| aspartate | 0.936 | 0.852 | 0.817 | 0.494 | 1.484 | 0.130 | 1.175 | 0.520 |
| serotonin | 0.832 | 0.576 | 0.932 | 0.468 | 1.552 | 0.520 | 0.717 | 0.468 |
| dimethylglycine | 0.973 | 0.724 | 1.158 | 0.254 | 0.969 | 0.724 | 1.040 | 0.820 |
| kynurenic acid | 0.902 | 0.724 | 1.281 | 0.468 | 1.043 | 0.820 | 0.978 | 0.724 |
| 5-adenosylhomocysteine | 1.098 | 0.820 | 1.044 | 0.548 | 1.004 | 0.576 | 0.676 | 0.085 |
| creatine | 1.107 | 0.984 | 1.129 | 0.788 | 0.852 | 0.395 | 0.733 | 0.130 |
| creatinine | 1.011 | 0.633 | 1.086 | 0.290 | 1.027 | 0.984 | 0.941 | 0.984 |
| thyroxine | 1.155 | 0.443 | 1.187 | 0.373 | 1.123 | 0.443 | 1.115 | 0.694 |
| cAMP | 0.946 | 0.917 | 0.725 | 0.395 | 0.931 | 0.984 | 0.686 | 0.237 |
| putrescine | 1.141 | 0.633 | 0.783 | 0.443 | 0.598 | 0.101 | 0.472 | 0.008 |
| arachidonyl_carnitine | 1.216 | 0.756 | 0.628 | 0.110 | 0.886 | 0.756 | 0.968 | 0.576 |
| 4-pyridoxate | 1.201 | 0.788 | 1.253 | 0.724 | 0.994 | 0.756 | 1.037 | 0.520 |
| uridine | 0.966 | 0.984 | 0.876 | 0.724 | 1.852 | 0.059 | 0.978 | 0.984 |
| taurocholate | 0.931 | 0.663 | 1.048 | 0.756 | 0.831 | 0.468 | 1.167 | 0.984 |
| glycodeoxycholate/glycochenodeoxycholate | 1.209 | 0.548 | 1.281 | 0.141 | 0.911 | 0.885 | 1.215 | 0.576 |
| 3-methyladipate/pimelate | 1.205 | 0.254 | 0.932 | 0.590 | 1.000 | 0.724 | 0.898 | 0.494 |
| arginine | 0.908 | 0.950 | 1.418 | 0.191 | 1.025 | 0.604 | 1.221 | 0.468 |
| anthranilic acid | 0.639 | 0.221 | 0.899 | 0.788 | 1.163 | 0.663 | 0.777 | 0.694 |
| hexoses (HILIC pos) | 1.142 | 0.604 | 0.873 | 0.520 | 0.892 | 0.917 | 1.422 | 0.110 |
| propionylcarnitine | 0.976 | 0.885 | 1.085 | 0.788 | 0.961 | 0.820 | 0.853 | 0.520 |

| **Metabolite** | **TBC1D1 FC** | **TBC1D1 P VALUE** | **TBC1D10A FC** | **TBC1D10A P VALUE** | **TBC1D10B FC** | **TBC1D10B P VALUE** | **TBC1D10C FC** | **TBC1D10C P VALUE** |
| --- | --- | --- | --- | --- | --- | --- | --- | --- |
| 1-methylnicotinamide | 131.212 | <.0001 | 0.263 | 0.220 | 0.083 | 0.134 | 0.076 | 0.021 |
| alpha-glycerophosphate | 0.239 | 0.001 | 2.419 | 0.048 | 1.326 | 0.149 | 1.660 | 0.221 |
| malonylcarnitine | 0.298 | 0.001 | 1.414 | 0.385 | 1.161 | 0.292 | 2.555 | 0.004 |
| erythrose-4-phosphate | 0.651 | 0.008 | 1.044 | 0.664 | 1.211 | 0.183 | 1.251 | 0.158 |
| N-carbamoyl-beta-alanine | 3.733 | 0.001 | 0.708 | 0.553 | 0.845 | 0.292 | 0.341 | 0.011 |
| hexanoylcarnitine | 0.264 | <.0001 | 1.513 | 0.268 | 2.126 | 0.040 | 1.772 | 0.019 |
| beta-alanine | 2.201 | 0.011 | 0.711 | 0.192 | 0.791 | 0.437 | 0.979 | 0.820 |
| 3-phosphoglycerate | 1.100 | 0.756 | 0.957 | 0.502 | 0.758 | 0.267 | 1.177 | 0.604 |
| inositol | 1.604 | 0.024 | 0.750 | 0.155 | 1.071 | 0.912 | 0.780 | 0.178 |
| phenylalanine | 0.762 | 0.663 | 1.252 | 0.874 | 1.188 | 0.506 | 1.325 | 0.633 |
| GABA | 4.698 | 0.008 | 0.173 | 0.002 | 0.716 | 0.657 | 1.306 | 0.917 |
| acetylcarnitine | 0.361 | 0.007 | 1.389 | 0.553 | 1.237 | 0.579 | 1.804 | 0.178 |
| butyrylcarnitine/isobutyrylcarnitine | 0.296 | 0.024 | 0.929 | 0.843 | 2.421 | 0.096 | 2.574 | 0.036 |
| myristoylcarnitine | 0.185 | 0.002 | 2.069 | 0.304 | 5.365 | 0.027 | 2.646 | 0.040 |
| 2-aminoadipate | 1.438 | 0.065 | 0.902 | 0.580 | 0.876 | 0.405 | 0.856 | 0.419 |
| DHAP/glyceraldehyde 3P | 1.024 | 0.663 | 1.216 | 0.553 | 0.660 | 0.052 | 0.768 | 0.373 |
| alanine | 0.745 | 0.520 | 1.455 | 0.268 | 1.060 | 0.956 | 1.655 | 0.178 |
| serine | 0.566 | 0.059 | 1.710 | 0.048 | 1.428 | 0.108 | 1.777 | 0.101 |
| glutamate | 1.528 | 0.054 | 1.102 | 0.693 | 0.928 | 0.405 | 0.784 | 0.351 |
| valine | 0.897 | 0.724 | 1.140 | 0.635 | 1.049 | 0.405 | 1.082 | 0.917 |
| lauroylcarnitine | 0.184 | 0.001 | 2.530 | 0.105 | 6.976 | 0.006 | 2.916 | 0.008 |
| cytidine | 0.463 | 0.065 | 1.171 | 0.635 | 1.613 | 0.437 | 2.171 | 0.093 |
| GMP | 0.659 | 0.044 | 1.468 | 0.105 | 1.253 | 0.202 | 1.459 | 0.059 |
| kynurenine | 2.534 | 0.014 | 0.443 | 0.030 | 0.801 | 0.471 | 0.666 | 0.290 |
| lactate | 1.307 | 0.310 | 1.025 | 0.843 | 1.126 | 0.912 | 0.894 | 0.724 |
| alpha-hydroxybutyrate | 1.570 | 0.024 | 0.732 | 0.167 | 1.009 | 0.956 | 0.727 | 0.059 |
| asparagine | 0.301 | 0.004 | 2.524 | 0.030 | 1.559 | 0.346 | 2.028 | 0.141 |
| lysine | 0.789 | 0.633 | 1.338 | 0.527 | 1.319 | 0.437 | 0.968 | 0.852 |
| tryptophan | 0.867 | 0.984 | 1.141 | 0.843 | 1.227 | 0.739 | 1.240 | 0.788 |
| 2-deoxycytidine | 0.372 | 0.065 | 0.970 | 0.874 | 1.261 | 0.618 | 2.304 | 0.130 |
| palmitoylcarnitine | 0.201 | 0.011 | 1.872 | 0.304 | 5.533 | 0.023 | 1.922 | 0.290 |
| guanosine | 1.075 | 0.694 | 0.564 | 0.114 | 1.012 | 0.956 | 0.962 | 0.852 |
| inosine | 0.933 | 0.917 | 0.765 | 0.236 | 1.662 | 0.076 | 0.779 | 0.351 |
| ribose-5-P/ribulose5-P | 0.810 | 0.443 | 1.313 | 0.268 | 0.820 | 0.506 | 1.449 | 0.548 |
| sorbitol | 0.996 | 0.330 | 1.428 | 0.268 | 1.219 | 0.956 | 1.093 | 0.788 |
| 2-hydroxyglutarate | 2.112 | 0.036 | 0.988 | 0.607 | 0.648 | 0.222 | 0.631 | 0.191 |
| threonine | 0.726 | 0.395 | 1.183 | 0.693 | 0.926 | 0.912 | 1.353 | 0.604 |
| isoleucine | 0.808 | 0.885 | 1.271 | 0.937 | 1.156 | 0.506 | 1.278 | 0.820 |
| 5-HIAA | 1.572 | 0.033 | 0.667 | 0.063 | 1.198 | 0.346 | 0.961 | 0.984 |
| trimethylamine-N-oxide | 1.373 | 0.054 | 0.746 | 0.082 | 1.003 | 0.912 | 0.869 | 0.310 |
| butyrobetaine | 0.473 | 0.005 | 1.241 | 0.580 | 1.181 | 0.657 | 1.506 | 0.130 |
| stearoylcarnitine | 0.251 | 0.029 | 1.479 | 0.580 | 3.228 | 0.067 | 2.431 | 0.110 |
| F1P/F6P/G1P/G6P | 0.584 | 0.054 | 1.321 | 0.363 | 0.815 | 0.657 | 1.520 | 0.165 |
| hexoses (HILIC neg) | 0.834 | 0.950 | 1.389 | 0.527 | 1.700 | 0.166 | 0.952 | 0.820 |
| hypoxanthine | 0.488 | 0.141 | 1.871 | 0.058 | 2.238 | 0.202 | 1.234 | 0.756 |
| NAD | 1.103 | 0.494 | 1.106 | 0.874 | 0.605 | 0.096 | 1.012 | 0.984 |
| NADP | 1.037 | 0.852 | 1.489 | 0.363 | 1.052 | 0.739 | 1.537 | 0.165 |
| methionine | 0.668 | 0.633 | 1.509 | 0.635 | 1.241 | 0.318 | 1.390 | 0.633 |
| glutamine | 0.523 | 0.049 | 1.630 | 0.429 | 1.673 | 0.120 | 1.398 | 0.272 |
| leucine | 0.766 | 0.885 | 1.399 | 0.874 | 1.216 | 0.579 | 1.236 | 0.984 |
| tyrosine | 0.781 | 0.852 | 1.210 | 0.969 | 0.962 | 0.698 | 1.360 | 0.468 |
| thiamine | 1.153 | 0.494 | 1.021 | 0.580 | 1.309 | 0.698 | 0.925 | 0.724 |
| niacinamide | 1.087 | 0.984 | 1.122 | 0.906 | 1.692 | 0.318 | 0.822 | 0.852 |
| alpha-glycerophosphocholine | 0.840 | 0.694 | 0.656 | 0.477 | 0.798 | 0.618 | 0.762 | 0.548 |
| thymidine | 1.449 | 0.468 | 0.497 | 0.105 | 2.002 | 0.318 | 0.555 | 0.237 |
| carnitine | 0.513 | 0.015 | 1.225 | 0.722 | 1.210 | 0.542 | 1.750 | 0.093 |
| oleylcarnitine | 0.357 | 0.059 | 1.145 | 0.874 | 3.435 | 0.108 | 1.850 | 0.178 |
| aconitate | 0.649 | 0.254 | 1.272 | 0.580 | 1.252 | 0.318 | 1.648 | 0.033 |
| CMP | 1.095 | 0.788 | 0.945 | 0.813 | 1.042 | 0.318 | 1.007 | 0.820 |
| cystathionine | 1.222 | 0.663 | 0.948 | 0.937 | 0.700 | 0.405 | 0.976 | 0.984 |
| fumarate/maleate/alpha-ketoisovalerate | 0.952 | 0.917 | 0.881 | 0.236 | 0.787 | 0.202 | 1.236 | 0.576 |
| glutathione oxidized | 1.615 | 0.237 | 1.353 | 0.385 | 0.790 | 0.956 | 1.329 | 0.272 |
| hippurate | 0.610 | 0.085 | 1.589 | 0.323 | 1.736 | 0.023 | 1.067 | 0.852 |
| pantothenate | 0.856 | 0.852 | 1.381 | 0.363 | 0.985 | 0.739 | 1.437 | 0.419 |
| sucrose | 1.288 | 0.373 | 1.458 | 0.502 | 0.875 | 0.471 | 0.623 | 0.120 |
| UMP | 0.762 | 0.191 | 1.036 | 0.782 | 1.168 | 0.657 | 1.380 | 0.178 |
| UDP-galactose/UDP-glucose | 1.491 | 0.017 | 1.252 | 0.813 | 0.466 | 0.166 | 1.083 | 0.984 |
| 6-phosphogluconate | 0.556 | 0.206 | 0.912 | 0.782 | 2.510 | 0.149 | 2.305 | 0.078 |
| histidine | 0.631 | 0.178 | 1.464 | 0.385 | 1.126 | 0.912 | 1.473 | 0.330 |
| proline | 0.729 | 0.310 | 1.206 | 0.937 | 1.436 | 0.166 | 1.227 | 0.494 |
| cis/trans-hydroxyproline | 0.573 | 0.071 | 2.235 | 0.010 | 2.367 | 0.149 | 1.402 | 0.221 |
| homocysteine | 1.426 | 0.290 | 0.597 | 0.063 | 0.812 | 0.912 | 0.668 | 0.110 |
| SDMA/ADMA | 0.662 | 0.165 | 1.562 | 0.236 | 1.540 | 0.031 | 1.237 | 0.330 |
| NMMA | 0.685 | 0.191 | 1.604 | 0.075 | 1.650 | 0.108 | 1.193 | 0.604 |
| carnosine | 0.951 | 0.820 | 0.975 | 0.843 | 1.449 | 0.134 | 1.582 | 0.237 |
| adenosine | 0.963 | 0.694 | 0.796 | 0.429 | 0.973 | 1.000 | 1.284 | 0.852 |
| 2-deoxyadenosine | 0.589 | 0.101 | 0.737 | 0.635 | 2.618 | 0.040 | 1.179 | 0.820 |
| cotinine | 1.520 | 0.206 | 0.695 | 0.268 | 0.680 | 0.267 | 0.719 | 0.206 |
| pipecolic acid | 1.608 | 0.237 | 0.797 | 0.453 | 1.136 | 0.375 | 1.114 | 0.520 |
| pyroglutamic acid | 0.726 | 0.548 | 1.458 | 0.453 | 1.413 | 0.506 | 1.190 | 0.950 |
| sarcosine | 0.702 | 0.152 | 1.404 | 0.167 | 1.257 | 0.437 | 1.041 | 0.984 |
| isocitrate | 0.734 | 0.494 | 1.291 | 0.937 | 0.927 | 0.824 | 1.483 | 0.141 |
| dCMP | 1.220 | 0.373 | 0.745 | 0.075 | 1.181 | 0.267 | 1.113 | 0.788 |
| glutathione reduced | 1.714 | 0.165 | 0.936 | 0.813 | 0.458 | 0.020 | 0.998 | 0.852 |
| lactose | 1.035 | 0.917 | 2.072 | 0.179 | 0.859 | 0.579 | 0.707 | 0.548 |
| oxalate | 1.263 | 0.152 | 0.728 | 0.075 | 0.935 | 0.346 | 1.005 | 0.984 |
| thymine | 1.039 | 0.756 | 0.959 | 0.635 | 1.981 | 0.096 | 1.481 | 0.254 |
| taurodeoxycholate/taurochenodeoxycholate | 1.564 | 0.984 | 1.935 | 0.363 | 1.670 | 0.698 | 0.364 | 0.443 |
| malondialdehyde | 1.266 | 0.245 | 0.672 | 0.005 | 0.953 | 0.598 | 0.986 | 0.534 |
| ornithine | 0.453 | 0.024 | 1.842 | 0.048 | 1.842 | 0.134 | 1.531 | 0.290 |
| citrulline | 0.637 | 0.093 | 1.494 | 0.252 | 1.698 | 0.040 | 1.296 | 0.373 |
| taurine | 0.745 | 0.663 | 1.251 | 0.580 | 0.912 | 0.506 | 2.039 | 0.120 |
| acetylglycine | 0.718 | 0.237 | 0.986 | 0.782 | 0.887 | 0.657 | 1.336 | 0.548 |
| allantoin | 0.775 | 0.290 | 1.539 | 0.133 | 1.743 | 0.020 | 1.119 | 0.468 |
| betaine | 0.667 | 0.059 | 1.163 | 0.527 | 1.469 | 0.027 | 1.314 | 0.178 |
| choline | 0.988 | 0.950 | 1.096 | 0.937 | 1.644 | 0.031 | 1.129 | 0.468 |
| acetylcholine | 0.384 | 0.272 | 0.568 | 0.453 | 5.705 | 0.059 | 1.173 | 0.724 |
| xanthosine | 0.854 | 0.468 | 1.117 | 0.906 | 1.584 | 0.086 | 1.204 | 0.272 |
| methionine sulfoxide | 0.716 | 0.419 | 1.412 | 0.144 | 1.540 | 0.149 | 1.383 | 0.663 |
| valerylcarnitine/isovalerylcarnitine/2-methylbutyroylcarnitine | 0.439 | 0.054 | 1.865 | 0.123 | 2.144 | 0.076 | 1.116 | 0.917 |
| heptanoylcarnitine | 0.561 | 0.040 | 1.205 | 0.722 | 1.182 | 0.183 | 1.279 | 0.310 |
| anserine | 0.961 | 0.633 | 1.076 | 0.453 | 1.183 | 0.405 | 1.202 | 0.272 |
| adenine | 1.397 | 0.254 | 0.694 | 0.252 | 1.113 | 0.739 | 0.843 | 0.633 |
| adipate | 1.077 | 0.272 | 0.923 | 0.477 | 0.945 | 0.437 | 0.995 | 0.950 |
| alpha-ketoglutarate | 1.045 | 0.724 | 0.864 | 0.385 | 0.906 | 0.657 | 1.027 | 0.917 |
| AMP | 0.734 | 0.272 | 1.093 | 0.693 | 0.936 | 1.000 | 1.534 | 0.110 |
| citrate | 0.697 | 0.310 | 1.560 | 0.722 | 0.900 | 0.542 | 1.543 | 0.078 |
| glucuronate | 1.247 | 0.443 | 0.955 | 0.906 | 1.137 | 1.000 | 0.764 | 0.141 |
| malate | 0.956 | 0.984 | 0.994 | 0.607 | 0.753 | 0.096 | 1.142 | 0.724 |
| PEP | 0.859 | 0.178 | 1.097 | 0.453 | 0.967 | 0.739 | 1.412 | 0.141 |
| succinate/methylmalonate | 0.725 | 0.071 | 1.286 | 0.323 | 1.354 | 0.149 | 1.082 | 0.520 |
| uracil | 0.654 | 0.191 | 1.679 | 0.192 | 2.044 | 0.015 | 1.594 | 0.130 |
| urate | 1.027 | 0.950 | 1.564 | 0.429 | 0.845 | 0.657 | 1.280 | 0.756 |
| xanthine | 0.658 | 0.165 | 1.795 | 0.192 | 1.871 | 0.020 | 1.556 | 0.221 |
| phosphocreatine | 0.481 | 0.237 | 1.972 | 0.323 | 1.101 | 0.375 | 1.405 | 0.468 |
| glycine | 1.118 | 0.724 | 1.205 | 0.286 | 0.784 | 0.267 | 1.048 | 0.756 |
| aspartate | 0.560 | 0.033 | 1.379 | 0.268 | 1.603 | 0.134 | 1.147 | 0.395 |
| serotonin | 0.820 | 0.520 | 0.568 | 0.220 | 0.907 | 0.782 | 1.882 | 0.141 |
| dimethylglycine | 0.694 | 0.165 | 1.536 | 0.206 | 1.526 | 0.023 | 1.324 | 0.290 |
| kynurenic acid | 0.650 | 0.395 | 1.638 | 0.429 | 1.830 | 0.096 | 1.301 | 0.494 |
| 5-adenosylhomocysteine | 1.027 | 0.724 | 1.005 | 0.969 | 0.913 | 0.542 | 1.487 | 0.419 |
| creatine | 0.824 | 0.221 | 0.930 | 0.813 | 1.473 | 0.222 | 1.165 | 0.351 |
| creatinine | 0.876 | 0.494 | 1.383 | 0.304 | 1.454 | 0.120 | 1.095 | 0.917 |
| thyroxine | 0.953 | 0.852 | 1.338 | 0.206 | 1.356 | 0.134 | 1.127 | 0.494 |
| cAMP | 0.692 | 0.443 | 0.830 | 0.453 | 1.025 | 0.782 | 0.912 | 0.604 |
| putrescine | 1.214 | 0.604 | 0.751 | 0.304 | 0.807 | 0.375 | 1.224 | 0.548 |
| arachidonyl_carnitine | 0.833 | 0.468 | 0.773 | 0.155 | 1.166 | 0.542 | 1.469 | 0.110 |
| 4-pyridoxate | 0.957 | 0.520 | 1.118 | 0.477 | 1.892 | 0.052 | 1.166 | 0.468 |
| uridine | 0.755 | 0.373 | 1.218 | 0.527 | 1.610 | 0.149 | 0.981 | 0.852 |
| taurocholate | 0.916 | 0.885 | 1.179 | 0.937 | 1.089 | 0.912 | 0.746 | 0.290 |
| glycodeoxycholate/glycochenodeoxycholate | 0.872 | 0.724 | 1.245 | 0.429 | 1.453 | 0.267 | 1.259 | 0.310 |
| 3-methyladipate/pimelate | 1.080 | 0.604 | 0.838 | 0.236 | 0.983 | 0.912 | 0.962 | 0.494 |
| arginine | 0.609 | 0.085 | 1.637 | 0.220 | 1.374 | 0.149 | 1.189 | 0.494 |
| anthranilic acid | 0.903 | 0.633 | 0.912 | 0.969 | 1.517 | 0.096 | 0.730 | 0.310 |
| hexoses (HILIC pos) | 0.786 | 0.724 | 1.446 | 0.363 | 1.203 | 0.868 | 1.030 | 0.788 |
| propionylcarnitine | 0.555 | 0.178 | 1.173 | 0.906 | 1.009 | 0.782 | 1.024 | 0.663 |

| **Metabolite** | **TBC1D12 FC** | **TBC1D12 P VALUE** | **TBC1D13 FC** | **TBC1D13 P VALUE** | **TBC1D14 FC** | **TBC1D14 P VALUE** | **TBC1D15 FC** | **TBC1D15 P VALUE** |
| --- | --- | --- | --- | --- | --- | --- | --- | --- |
| 1-methylnicotinamide | 0.026 | 0.003 | 0.470 | 0.520 | 0.189 | 0.152 | 1.223 | 0.852 |
| alpha-glycerophosphate | 2.652 | 0.055 | 0.879 | 0.788 | 0.754 | 0.663 | 0.947 | 0.950 |
| malonylcarnitine | 1.992 | 0.106 | 0.751 | 0.468 | 0.930 | 0.917 | 1.240 | 0.237 |
| erythrose-4-phosphate | 1.052 | 0.827 | 0.879 | 0.468 | 0.975 | 0.852 | 1.004 | 0.950 |
| N-carbamoyl-beta-alanine | 0.313 | 0.016 | 0.746 | 0.373 | 0.541 | 0.110 | 0.837 | 0.984 |
| hexanoylcarnitine | 2.823 | 0.004 | 0.851 | 0.917 | 1.114 | 0.694 | 1.122 | 0.395 |
| beta-alanine | 0.458 | 0.010 | 0.756 | 0.254 | 0.870 | 0.468 | 0.681 | 0.130 |
| 3-phosphoglycerate | 0.822 | 0.541 | 1.122 | 0.984 | 0.961 | 0.663 | 0.976 | 0.576 |
| inositol | 0.596 | 0.021 | 0.819 | 0.351 | 1.141 | 0.604 | 1.519 | 0.078 |
| phenylalanine | 0.788 | 0.663 | 0.826 | 0.820 | 0.722 | 0.548 | 0.728 | 0.520 |
| GABA | 0.338 | 0.097 | 0.713 | 0.494 | 1.057 | 0.885 | 0.433 | 0.141 |
| acetylcarnitine | 2.213 | 0.029 | 0.783 | 0.633 | 0.842 | 0.950 | 0.931 | 0.724 |
| butyrylcarnitine/isobutyrylcarnitine | 2.464 | 0.106 | 0.983 | 0.820 | 1.055 | 0.520 | 0.652 | 0.520 |
| myristoylcarnitine | 2.352 | 0.150 | 0.953 | 0.788 | 1.397 | 0.520 | 2.263 | 0.152 |
| 2-aminoadipate | 0.810 | 0.407 | 0.686 | 0.021 | 0.807 | 0.254 | 1.043 | 0.917 |
| DHAP/glyceraldehyde 3P | 0.957 | 0.861 | 1.096 | 0.852 | 0.964 | 0.820 | 0.951 | 0.694 |
| alanine | 0.764 | 0.222 | 0.957 | 0.633 | 0.730 | 0.419 | 0.859 | 0.852 |
| serine | 1.003 | 0.930 | 0.829 | 0.852 | 0.704 | 0.950 | 1.093 | 0.049 |
| glutamate | 0.752 | 0.150 | 0.831 | 0.165 | 0.973 | 0.724 | 0.710 | 0.221 |
| valine | 0.803 | 0.513 | 0.999 | 0.852 | 0.851 | 0.694 | 0.780 | 0.520 |
| lauroylcarnitine | 1.846 | 0.295 | 0.642 | 0.237 | 1.378 | 0.604 | 1.356 | 0.443 |
| cytidine | 2.450 | 0.050 | 2.115 | 0.141 | 0.990 | 0.820 | 1.227 | 0.604 |
| GMP | 1.282 | 0.337 | 1.071 | 0.694 | 1.185 | 0.576 | 1.281 | 0.351 |
| kynurenine | 0.616 | 0.190 | 0.983 | 0.917 | 0.974 | 0.756 | 0.634 | 0.165 |
| lactate | 0.733 | 0.239 | 0.984 | 0.494 | 0.924 | 0.419 | 0.828 | 0.885 |
| alpha-hydroxybutyrate | 0.780 | 0.206 | 0.876 | 0.237 | 1.115 | 0.820 | 0.919 | 0.373 |
| asparagine | 1.613 | 0.407 | 0.556 | 0.290 | 0.531 | 0.221 | 1.708 | 0.093 |
| lysine | 0.785 | 0.485 | 1.025 | 0.917 | 0.800 | 0.548 | 0.775 | 0.520 |
| tryptophan | 0.839 | 0.485 | 0.895 | 0.984 | 0.862 | 0.950 | 0.651 | 0.310 |
| 2-deoxycytidine | 5.039 | 0.004 | 1.900 | 0.206 | 0.602 | 0.395 | 1.075 | 0.663 |
| palmitoylcarnitine | 2.227 | 0.190 | 1.234 | 0.820 | 1.431 | 0.468 | 2.071 | 0.165 |
| guanosine | 1.390 | 0.359 | 1.291 | 0.254 | 1.283 | 0.330 | 0.987 | 0.852 |
| inosine | 1.148 | 0.695 | 1.169 | 0.520 | 1.164 | 0.520 | 0.794 | 0.788 |
| ribose-5-P/ribulose5-P | 0.813 | 0.793 | 0.682 | 0.443 | 0.629 | 0.085 | 0.767 | 0.633 |
| sorbitol | 0.642 | 0.055 | 0.755 | 0.221 | 0.603 | 0.012 | 0.856 | 0.663 |
| 2-hydroxyglutarate | 0.983 | 0.861 | 1.458 | 0.576 | 1.111 | 0.885 | 0.792 | 0.373 |
| threonine | 0.983 | 0.896 | 1.007 | 0.756 | 0.801 | 0.788 | 0.890 | 0.694 |
| isoleucine | 0.791 | 0.190 | 0.736 | 0.724 | 0.655 | 0.206 | 0.827 | 0.494 |
| 5-HIAA | 0.777 | 0.337 | 1.276 | 0.351 | 1.307 | 0.310 | 0.710 | 0.065 |
| trimethylamine-N-oxide | 0.926 | 0.861 | 1.117 | 0.419 | 1.047 | 0.724 | 0.980 | 0.724 |
| butyrobetaine | 1.730 | 0.089 | 0.902 | 0.694 | 0.816 | 0.788 | 0.848 | 0.885 |
| stearoylcarnitine | 2.092 | 0.275 | 0.949 | 0.984 | 1.208 | 0.663 | 1.598 | 0.468 |
| F1P/F6P/G1P/G6P | 1.413 | 0.275 | 0.757 | 0.237 | 0.965 | 0.788 | 1.125 | 0.724 |
| hexoses (HILIC neg) | 0.682 | 0.163 | 0.790 | 0.694 | 0.738 | 0.694 | 0.783 | 0.694 |
| hypoxanthine | 1.765 | 0.275 | 1.050 | 0.984 | 1.224 | 0.885 | 1.116 | 0.820 |
| NAD | 0.966 | 0.571 | 0.929 | 0.756 | 1.059 | 0.852 | 0.877 | 0.724 |
| NADP | 0.621 | 0.097 | 0.923 | 0.395 | 1.078 | 0.984 | 0.814 | 0.395 |
| methionine | 0.878 | 0.239 | 0.761 | 0.950 | 0.637 | 0.330 | 0.830 | 0.520 |
| glutamine | 1.090 | 0.359 | 0.803 | 0.885 | 0.755 | 0.468 | 0.766 | 0.984 |
| leucine | 0.800 | 0.163 | 0.783 | 0.917 | 0.698 | 0.330 | 0.779 | 0.494 |
| tyrosine | 0.834 | 0.513 | 0.749 | 0.520 | 0.676 | 0.310 | 0.866 | 0.756 |
| thiamine | 0.718 | 0.257 | 0.904 | 0.820 | 0.908 | 0.694 | 0.609 | 0.272 |
| niacinamide | 0.644 | 0.316 | 1.105 | 0.950 | 0.933 | 0.520 | 0.676 | 0.494 |
| alpha-glycerophosphocholine | 0.944 | 0.965 | 0.587 | 0.101 | 0.629 | 0.120 | 0.681 | 0.468 |
| thymidine | 1.258 | 0.359 | 2.104 | 0.029 | 1.300 | 0.373 | 0.774 | 0.548 |
| carnitine | 1.500 | 0.176 | 0.817 | 0.468 | 0.814 | 0.788 | 0.941 | 0.633 |
| oleylcarnitine | 2.225 | 0.138 | 0.863 | 0.724 | 1.106 | 0.724 | 2.363 | 0.120 |
| aconitate | 1.033 | 0.760 | 0.890 | 0.724 | 0.993 | 0.885 | 0.987 | 0.756 |
| CMP | 1.094 | 0.861 | 1.109 | 0.663 | 1.115 | 0.548 | 0.987 | 0.917 |
| cystathionine | 1.552 | 0.432 | 1.555 | 0.290 | 1.762 | 0.178 | 0.507 | 0.221 |
| fumarate/maleate/alpha-ketoisovalerate | 1.086 | 0.965 | 0.961 | 0.917 | 0.833 | 0.520 | 0.731 | 0.141 |
| glutathione oxidized | 0.696 | 0.206 | 0.761 | 0.351 | 1.124 | 0.852 | 0.775 | 0.290 |
| hippurate | 1.138 | 0.631 | 0.826 | 0.756 | 0.847 | 0.950 | 1.054 | 0.724 |
| pantothenate | 1.280 | 0.896 | 0.877 | 0.694 | 1.112 | 0.395 | 0.725 | 0.330 |
| sucrose | 0.669 | 0.138 | 0.876 | 0.633 | 1.281 | 0.419 | 1.255 | 0.237 |
| UMP | 1.201 | 0.190 | 1.079 | 0.852 | 0.972 | 0.852 | 1.069 | 0.694 |
| UDP-galactose/UDP-glucose | 0.764 | 0.097 | 0.713 | 0.310 | 0.937 | 0.950 | 1.043 | 0.694 |
| 6-phosphogluconate | 0.969 | 1.000 | 1.495 | 0.351 | 1.141 | 0.950 | 0.624 | 0.272 |
| histidine | 0.959 | 0.965 | 0.735 | 0.885 | 0.612 | 0.101 | 0.995 | 0.756 |
| proline | 1.033 | 0.601 | 0.903 | 0.604 | 0.874 | 0.633 | 0.765 | 0.395 |
| cis/trans-hydroxyproline | 1.214 | 0.861 | 0.803 | 0.237 | 0.725 | 0.310 | 0.992 | 0.694 |
| homocysteine | 1.011 | 0.827 | 1.245 | 0.395 | 1.655 | 0.110 | 0.781 | 0.443 |
| SDMA/ADMA | 0.928 | 0.383 | 0.832 | 0.663 | 0.715 | 0.237 | 0.799 | 0.633 |
| NMMA | 0.780 | 0.458 | 0.880 | 0.604 | 0.708 | 0.130 | 0.774 | 0.468 |
| carnosine | 0.662 | 0.074 | 0.765 | 0.272 | 0.629 | 0.237 | 0.826 | 0.724 |
| adenosine | 1.085 | 1.000 | 1.696 | 0.101 | 1.018 | 0.917 | 1.215 | 0.724 |
| 2-deoxyadenosine | 2.032 | 0.040 | 1.187 | 0.852 | 1.168 | 0.663 | 1.459 | 0.165 |
| cotinine | 0.814 | 0.571 | 0.862 | 0.419 | 0.919 | 0.494 | 0.778 | 0.548 |
| pipecolic acid | 0.599 | 0.163 | 0.623 | 0.065 | 0.826 | 0.788 | 1.625 | 0.373 |
| pyroglutamic acid | 0.744 | 0.407 | 0.885 | 0.950 | 0.703 | 0.468 | 0.814 | 0.984 |
| sarcosine | 1.077 | 0.861 | 0.931 | 0.756 | 0.890 | 0.756 | 0.918 | 0.852 |
| isocitrate | 1.083 | 0.827 | 0.713 | 0.373 | 0.872 | 0.852 | 0.971 | 0.663 |
| dCMP | 1.202 | 0.513 | 0.900 | 0.984 | 0.836 | 0.724 | 0.868 | 0.419 |
| glutathione reduced | 1.037 | 0.965 | 0.776 | 0.310 | 1.122 | 0.984 | 0.784 | 0.373 |
| lactose | 0.665 | 0.337 | 0.646 | 0.141 | 1.449 | 0.604 | 0.872 | 0.663 |
| oxalate | 0.971 | 1.000 | 1.152 | 0.310 | 1.164 | 0.419 | 0.921 | 0.788 |
| thymine | 0.528 | 0.029 | 0.662 | 0.373 | 0.713 | 0.373 | 1.124 | 0.885 |
| taurodeoxycholate/taurochenodeoxycholate | 0.704 | 1.000 | 0.823 | 0.633 | 1.331 | 0.984 | 0.903 | 0.443 |
| malondialdehyde | 0.804 | 0.471 | 0.977 | 0.756 | 0.997 | 0.917 | 0.862 | 0.852 |
| ornithine | 1.043 | 0.695 | 0.667 | 0.351 | 0.603 | 0.206 | 1.222 | 0.633 |
| citrulline | 0.934 | 1.000 | 0.707 | 0.604 | 0.737 | 0.419 | 0.907 | 0.984 |
| taurine | 1.021 | 0.896 | 0.552 | 0.120 | 0.669 | 0.351 | 0.777 | 0.468 |
| acetylglycine | 1.090 | 0.793 | 1.061 | 0.852 | 1.054 | 0.756 | 0.986 | 0.984 |
| allantoin | 0.775 | 0.275 | 0.843 | 0.694 | 0.699 | 0.254 | 0.796 | 0.633 |
| betaine | 1.054 | 0.896 | 0.877 | 0.330 | 0.805 | 0.290 | 0.735 | 0.165 |
| choline | 0.709 | 0.081 | 0.892 | 0.950 | 0.880 | 0.548 | 0.929 | 0.950 |
| acetylcholine | 1.704 | 0.571 | 2.151 | 0.178 | 1.315 | 0.576 | 0.692 | 0.694 |
| xanthosine | 0.715 | 0.097 | 1.117 | 0.984 | 0.705 | 0.221 | 0.784 | 0.633 |
| methionine sulfoxide | 0.658 | 0.116 | 0.758 | 0.330 | 0.626 | 0.065 | 0.933 | 0.885 |
| valerylcarnitine/isovalerylcarnitine/2-methylbutyroylcarnitine | 1.535 | 0.407 | 1.582 | 0.290 | 1.364 | 0.330 | 1.533 | 0.206 |
| heptanoylcarnitine | 1.671 | 0.055 | 1.207 | 0.419 | 0.976 | 0.984 | 1.105 | 0.310 |
| anserine | 0.712 | 0.097 | 0.762 | 0.237 | 0.743 | 0.101 | 1.151 | 0.576 |
| adenine | 0.915 | 0.727 | 0.828 | 0.604 | 1.099 | 0.694 | 0.871 | 0.351 |
| adipate | 1.003 | 0.861 | 0.991 | 0.984 | 1.011 | 0.663 | 0.998 | 0.984 |
| alpha-ketoglutarate | 0.990 | 0.827 | 0.937 | 0.604 | 0.790 | 0.351 | 1.049 | 0.756 |
| AMP | 1.347 | 0.295 | 1.104 | 0.468 | 1.279 | 0.290 | 1.232 | 0.310 |
| citrate | 1.206 | 0.896 | 0.749 | 0.756 | 0.835 | 0.885 | 0.974 | 0.756 |
| glucuronate | 1.109 | 0.337 | 0.939 | 0.604 | 1.160 | 0.520 | 0.867 | 0.788 |
| malate | 1.051 | 0.965 | 0.879 | 0.788 | 0.861 | 0.663 | 0.868 | 0.520 |
| PEP | 1.106 | 0.407 | 1.027 | 0.984 | 1.150 | 0.548 | 0.996 | 0.950 |
| succinate/methylmalonate | 1.158 | 0.190 | 0.876 | 0.917 | 0.965 | 0.633 | 1.146 | 0.395 |
| uracil | 0.814 | 0.663 | 0.603 | 0.395 | 0.689 | 0.395 | 1.123 | 0.419 |
| urate | 0.825 | 0.663 | 0.621 | 0.633 | 1.122 | 0.576 | 0.663 | 0.395 |
| xanthine | 0.954 | 0.861 | 0.626 | 0.395 | 0.553 | 0.272 | 1.160 | 0.633 |
| phosphocreatine | 1.074 | 0.485 | 0.849 | 0.694 | 1.051 | 0.724 | 1.128 | 0.950 |
| glycine | 0.709 | 0.316 | 0.914 | 0.520 | 0.822 | 0.520 | 1.005 | 0.663 |
| aspartate | 0.839 | 0.571 | 0.636 | 0.065 | 0.961 | 0.330 | 1.082 | 0.852 |
| serotonin | 1.067 | 0.663 | 1.051 | 0.724 | 1.441 | 0.254 | 1.531 | 0.468 |
| dimethylglycine | 0.862 | 0.432 | 0.748 | 0.494 | 0.707 | 0.237 | 0.888 | 0.724 |
| kynurenic acid | 0.638 | 0.275 | 0.663 | 0.756 | 0.623 | 0.633 | 0.897 | 0.604 |
| 5-adenosylhomocysteine | 1.165 | 0.631 | 0.935 | 0.820 | 1.071 | 0.494 | 0.622 | 0.059 |
| creatine | 0.933 | 1.000 | 0.976 | 0.917 | 1.119 | 0.788 | 0.691 | 0.141 |
| creatinine | 0.738 | 0.097 | 0.789 | 0.494 | 0.797 | 0.443 | 0.870 | 0.663 |
| thyroxine | 0.790 | 0.485 | 0.677 | 0.152 | 0.760 | 0.272 | 1.009 | 0.950 |
| cAMP | 1.730 | 0.206 | 0.940 | 0.950 | 0.932 | 0.917 | 0.767 | 0.395 |
| putrescine | 0.715 | 0.316 | 0.742 | 0.310 | 0.674 | 0.221 | 0.731 | 0.290 |
| arachidonyl_carnitine | 1.198 | 0.407 | 1.271 | 0.520 | 0.963 | 0.984 | 0.940 | 0.663 |
| 4-pyridoxate | 1.029 | 0.727 | 1.072 | 0.756 | 1.161 | 0.468 | 1.207 | 0.330 |
| uridine | 0.936 | 0.861 | 1.278 | 0.520 | 0.611 | 0.254 | 1.173 | 0.633 |
| taurocholate | 0.863 | 0.257 | 0.882 | 0.330 | 0.816 | 0.130 | 1.071 | 0.694 |
| glycodeoxycholate/glycochenodeoxycholate | 0.787 | 0.432 | 0.847 | 0.310 | 0.767 | 0.351 | 1.047 | 0.917 |
| 3-methyladipate/pimelate | 0.870 | 0.541 | 0.918 | 0.576 | 0.980 | 0.917 | 1.026 | 0.678 |
| arginine | 1.002 | 0.965 | 0.724 | 0.310 | 0.644 | 0.141 | 0.989 | 0.885 |
| anthranilic acid | 0.879 | 0.513 | 0.920 | 0.984 | 0.548 | 0.078 | 0.810 | 0.576 |
| hexoses (HILIC pos) | 0.815 | 0.631 | 0.769 | 0.788 | 0.751 | 0.724 | 1.049 | 0.520 |
| propionylcarnitine | 1.527 | 0.359 | 1.393 | 0.395 | 1.228 | 0.290 | 0.975 | 0.724 |

| **Metabolite** | **TBC1D16 FC** | **TBC1D16 P VALUE** | **TBC1D17 FC** | **TBC1D17 P VALUE** | **TBC1D19 FC** | **TBC1D19 P VALUE** | **TBC1D2 FC** | **TBC1D2 P VALUE** |
| --- | --- | --- | --- | --- | --- | --- | --- | --- |
| 1-methylnicotinamide | 0.086 | 0.049 | 0.222 | 0.254 | 0.188 | 0.065 | 21.757 | 0.007 |
| alpha-glycerophosphate | 2.363 | 0.085 | 2.792 | 0.008 | 0.781 | 0.724 | 1.058 | 0.520 |
| malonylcarnitine | 0.862 | 0.494 | 1.343 | 0.419 | 0.987 | 0.694 | 0.790 | 0.351 |
| erythrose-4-phosphate | 1.261 | 0.300 | 1.494 | 0.031 | 0.625 | 0.054 | 1.092 | 0.310 |
| N-carbamoyl-beta-alanine | 0.364 | 0.019 | 1.084 | 0.885 | 0.728 | 0.395 | 3.593 | 0.006 |
| hexanoylcarnitine | 1.244 | 0.694 | 1.873 | 0.165 | 1.014 | 0.852 | 0.571 | 0.036 |
| beta-alanine | 0.622 | 0.071 | 1.141 | 0.548 | 0.631 | 0.152 | 2.186 | 0.005 |
| 3-phosphoglycerate | 1.042 | 0.917 | 1.086 | 0.633 | 0.685 | 0.065 | 1.791 | 0.014 |
| inositol | 0.654 | 0.044 | 1.068 | 0.663 | 1.054 | 0.885 | 1.278 | 0.101 |
| phenylalanine | 0.631 | 0.141 | 2.211 | 0.007 | 0.882 | 0.395 | 2.492 | 0.029 |
| GABA | 0.538 | 0.221 | 0.341 | 0.093 | 0.613 | 0.178 | 0.887 | 0.984 |
| acetylcarnitine | 0.803 | 0.494 | 1.202 | 0.724 | 1.167 | 0.633 | 1.185 | 0.950 |
| butyrylcarnitine/isobutyrylcarnitine | 1.655 | 0.254 | 1.766 | 0.290 | 0.558 | 0.272 | 0.649 | 0.419 |
| myristoylcarnitine | 2.131 | 0.351 | 2.071 | 0.330 | 0.612 | 0.520 | 0.438 | 0.054 |
| 2-aminoadipate | 0.640 | 0.024 | 0.915 | 0.494 | 1.283 | 0.049 | 1.078 | 0.917 |
| DHAP/glyceraldehyde 3P | 1.016 | 0.917 | 1.865 | 0.120 | 0.597 | 0.078 | 2.700 | 0.003 |
| alanine | 0.799 | 0.468 | 1.483 | 0.152 | 0.684 | 0.206 | 2.233 | 0.017 |
| serine | 0.823 | 0.694 | 1.774 | 0.024 | 0.966 | 0.950 | 1.735 | 0.085 |
| glutamate | 0.942 | 0.310 | 1.313 | 0.419 | 1.068 | 0.788 | 1.354 | 0.254 |
| valine | 0.725 | 0.237 | 2.006 | 0.003 | 0.878 | 0.548 | 2.028 | 0.027 |
| lauroylcarnitine | 1.910 | 0.330 | 2.552 | 0.130 | 0.835 | 0.756 | 0.504 | 0.110 |
| cytidine | 2.111 | 0.191 | 0.518 | 0.206 | 1.131 | 0.756 | 0.306 | 0.015 |
| GMP | 1.542 | 0.141 | 1.310 | 0.310 | 0.851 | 0.330 | 1.029 | 0.604 |
| kynurenine | 0.743 | 0.290 | 0.407 | 0.012 | 1.009 | 0.984 | 0.744 | 0.419 |
| lactate | 0.820 | 0.221 | 1.765 | 0.029 | 0.689 | 0.141 | 2.479 | 0.000 |
| alpha-hydroxybutyrate | 0.829 | 0.290 | 0.727 | 0.120 | 1.388 | 0.059 | 0.685 | 0.036 |
| asparagine | 0.789 | 0.756 | 1.118 | 0.330 | 0.874 | 0.633 | 1.179 | 0.395 |
| lysine | 0.833 | 0.520 | 2.515 | 0.005 | 0.886 | 0.468 | 2.363 | 0.044 |
| tryptophan | 0.682 | 0.178 | 2.063 | 0.007 | 0.955 | 0.756 | 2.083 | 0.033 |
| 2-deoxycytidine | 1.554 | 0.373 | 0.702 | 0.468 | 1.472 | 0.419 | 0.278 | 0.021 |
| palmitoylcarnitine | 2.116 | 0.272 | 2.020 | 0.272 | 0.622 | 0.604 | 0.417 | 0.085 |
| guanosine | 1.003 | 0.984 | 0.581 | 0.044 | 1.681 | 0.044 | 0.528 | 0.085 |
| inosine | 0.953 | 0.724 | 0.984 | 0.885 | 1.688 | 0.021 | 0.832 | 0.443 |
| ribose-5-P/ribulose5-P | 0.910 | 0.950 | 2.079 | 0.029 | 0.619 | 0.085 | 3.100 | 0.002 |
| sorbitol | 0.568 | 0.011 | 1.778 | 0.085 | 0.956 | 0.950 | 2.589 | 0.004 |
| 2-hydroxyglutarate | 1.768 | 0.191 | 0.770 | 0.756 | 0.525 | 0.040 | 0.966 | 0.468 |
| threonine | 0.703 | 0.373 | 1.849 | 0.012 | 0.844 | 0.520 | 1.937 | 0.085 |
| isoleucine | 0.611 | 0.085 | 1.849 | 0.010 | 0.932 | 0.165 | 2.288 | 0.015 |
| 5-HIAA | 1.004 | 0.950 | 0.694 | 0.054 | 1.208 | 0.272 | 0.752 | 0.206 |
| trimethylamine-N-oxide | 0.909 | 0.633 | 0.703 | 0.036 | 0.995 | 0.950 | 0.708 | 0.049 |
| butyrobetaine | 0.918 | 0.694 | 1.298 | 0.330 | 1.044 | 0.820 | 1.105 | 0.885 |
| stearoylcarnitine | 1.113 | 0.852 | 1.390 | 0.395 | 0.631 | 0.468 | 0.541 | 0.165 |
| F1P/F6P/G1P/G6P | 1.173 | 0.724 | 1.388 | 0.093 | 0.715 | 0.078 | 1.368 | 0.085 |
| hexoses (HILIC neg) | 0.820 | 0.520 | 1.961 | 0.049 | 1.186 | 0.950 | 2.488 | 0.015 |
| hypoxanthine | 1.117 | 0.788 | 1.132 | 0.576 | 4.646 | 0.001 | 0.587 | 0.419 |
| NAD | 0.955 | 0.756 | 1.100 | 0.694 | 0.854 | 0.548 | 1.829 | 0.027 |
| NADP | 1.284 | 0.663 | 1.466 | 0.237 | 0.912 | 0.694 | 1.696 | 0.120 |
| methionine | 0.648 | 0.272 | 2.071 | 0.002 | 0.934 | 0.237 | 2.537 | 0.011 |
| glutamine | 0.916 | 0.468 | 2.534 | 0.008 | 1.024 | 0.852 | 2.306 | 0.130 |
| leucine | 0.724 | 0.290 | 2.024 | 0.007 | 0.953 | 0.290 | 2.254 | 0.015 |
| tyrosine | 0.564 | 0.059 | 1.789 | 0.040 | 0.896 | 0.254 | 2.135 | 0.071 |
| thiamine | 0.820 | 0.395 | 2.638 | 0.004 | 0.866 | 0.310 | 2.748 | 0.014 |
| niacinamide | 0.923 | 0.520 | 2.830 | 0.005 | 0.809 | 0.468 | 2.981 | 0.006 |
| alpha-glycerophosphocholine | 0.699 | 0.206 | 0.917 | 0.820 | 1.538 | 0.395 | 1.001 | 0.633 |
| thymidine | 1.264 | 0.468 | 1.130 | 0.984 | 1.611 | 0.206 | 0.694 | 0.272 |
| carnitine | 0.775 | 0.178 | 1.113 | 0.576 | 1.052 | 0.788 | 1.304 | 0.724 |
| oleylcarnitine | 1.052 | 0.788 | 1.101 | 0.756 | 0.631 | 0.468 | 0.398 | 0.093 |
| aconitate | 1.008 | 0.576 | 1.658 | 0.093 | 0.846 | 0.395 | 1.719 | 0.021 |
| CMP | 1.436 | 0.033 | 0.777 | 0.272 | 0.961 | 0.950 | 0.633 | 0.049 |
| cystathionine | 1.208 | 0.756 | 1.438 | 0.663 | 1.258 | 0.443 | 0.559 | 0.191 |
| fumarate/maleate/alpha-ketoisovalerate | 0.920 | 0.984 | 0.957 | 0.724 | 0.758 | 0.120 | 1.064 | 0.820 |
| glutathione oxidized | 0.888 | 0.756 | 1.547 | 0.206 | 0.783 | 0.419 | 1.974 | 0.078 |
| hippurate | 0.610 | 0.049 | 1.960 | 0.021 | 1.266 | 0.520 | 2.140 | 0.078 |
| pantothenate | 0.883 | 0.604 | 1.585 | 0.101 | 0.884 | 0.756 | 1.642 | 0.165 |
| sucrose | 0.836 | 0.468 | 0.917 | 0.694 | 1.537 | 0.130 | 1.232 | 0.237 |
| UMP | 1.429 | 0.191 | 1.057 | 0.633 | 0.859 | 0.468 | 0.730 | 0.254 |
| UDP-galactose/UDP-glucose | 0.921 | 0.756 | 1.158 | 0.788 | 0.739 | 0.290 | 1.856 | 0.065 |
| 6-phosphogluconate | 1.849 | 0.141 | 1.691 | 0.419 | 0.637 | 0.221 | 1.557 | 0.443 |
| histidine | 0.542 | 0.059 | 1.662 | 0.078 | 0.963 | 0.290 | 1.927 | 0.191 |
| proline | 1.043 | 0.852 | 1.477 | 0.054 | 0.907 | 0.395 | 1.708 | 0.014 |
| cis/trans-hydroxyproline | 1.546 | 0.419 | 1.360 | 0.237 | 1.005 | 0.852 | 1.161 | 0.310 |
| homocysteine | 1.206 | 0.310 | 1.160 | 0.494 | 0.722 | 0.254 | 1.229 | 0.885 |
| SDMA/ADMA | 0.785 | 0.178 | 1.773 | 0.044 | 1.008 | 0.820 | 2.015 | 0.040 |
| NMMA | 0.850 | 0.604 | 2.556 | 0.002 | 0.863 | 0.756 | 2.068 | 0.036 |
| carnosine | 0.685 | 0.221 | 1.399 | 0.101 | 1.043 | 0.694 | 1.486 | 0.443 |
| adenosine | 0.808 | 0.820 | 0.907 | 0.917 | 0.576 | 0.152 | 1.467 | 0.272 |
| 2-deoxyadenosine | 0.802 | 0.633 | 2.141 | 0.071 | 0.964 | 0.984 | 1.272 | 0.494 |
| cotinine | 0.830 | 0.395 | 0.705 | 0.351 | 1.059 | 0.885 | 0.976 | 0.950 |
| pipecolic acid | 0.440 | 0.085 | 0.776 | 0.548 | 1.397 | 0.237 | 1.050 | 0.984 |
| pyroglutamic acid | 0.878 | 0.788 | 2.557 | 0.007 | 0.838 | 0.373 | 2.959 | 0.008 |
| sarcosine | 1.248 | 0.520 | 1.460 | 0.049 | 0.724 | 0.130 | 1.119 | 0.330 |
| isocitrate | 0.857 | 0.494 | 1.450 | 0.310 | 0.905 | 0.254 | 1.704 | 0.019 |
| dCMP | 0.918 | 0.917 | 0.748 | 0.310 | 1.002 | 0.419 | 0.641 | 0.040 |
| glutathione reduced | 0.844 | 0.576 | 1.074 | 0.820 | 0.838 | 0.494 | 1.282 | 0.548 |
| lactose | 0.869 | 0.852 | 1.309 | 0.351 | 1.706 | 0.373 | 1.763 | 0.027 |
| oxalate | 1.070 | 0.724 | 0.732 | 0.029 | 0.989 | 0.694 | 0.716 | 0.065 |
| thymine | 0.746 | 0.520 | 0.826 | 0.237 | 1.258 | 0.351 | 1.139 | 0.788 |
| taurodeoxycholate/taurochenodeoxycholate | 1.497 | 0.373 | 3.550 | 0.093 | 1.664 | 0.254 | 3.796 | 0.036 |
| malondialdehyde | 0.701 | 0.036 | 1.069 | 0.663 | 0.845 | 0.290 | 1.394 | 0.093 |
| ornithine | 0.626 | 0.633 | 1.878 | 0.101 | 1.389 | 0.395 | 1.453 | 0.494 |
| citrulline | 0.717 | 0.272 | 2.003 | 0.036 | 1.282 | 0.494 | 2.009 | 0.065 |
| taurine | 0.590 | 0.120 | 1.039 | 0.984 | 1.027 | 0.885 | 1.496 | 0.290 |
| acetylglycine | 1.004 | 0.885 | 0.915 | 0.756 | 1.005 | 0.576 | 0.763 | 0.237 |
| allantoin | 0.771 | 0.468 | 1.943 | 0.044 | 1.049 | 0.984 | 2.194 | 0.065 |
| betaine | 0.917 | 0.633 | 1.231 | 0.254 | 1.228 | 0.110 | 1.079 | 0.694 |
| choline | 0.782 | 0.351 | 1.273 | 0.373 | 1.026 | 0.694 | 1.297 | 0.141 |
| acetylcholine | 1.645 | 0.330 | 9.187 | 0.015 | 0.249 | 0.085 | 1.793 | 0.330 |
| xanthosine | 0.804 | 0.254 | 1.509 | 0.120 | 0.778 | 0.520 | 1.995 | 0.027 |
| methionine sulfoxide | 0.971 | 0.950 | 1.646 | 0.007 | 0.823 | 0.395 | 2.104 | 0.003 |
| valerylcarnitine/isovalerylcarnitine/2-methylbutyroylcarnitine | 2.126 | 0.036 | 1.190 | 0.663 | 0.885 | 0.852 | 1.204 | 0.788 |
| heptanoylcarnitine | 1.043 | 0.852 | 1.588 | 0.141 | 0.605 | 0.110 | 0.782 | 0.272 |
| anserine | 0.695 | 0.130 | 1.365 | 0.093 | 1.097 | 0.984 | 1.321 | 0.191 |
| adenine | 0.850 | 0.885 | 0.924 | 0.917 | 1.589 | 0.130 | 0.580 | 0.330 |
| adipate | 0.865 | 0.191 | 0.911 | 0.330 | 1.168 | 0.206 | 0.904 | 0.395 |
| alpha-ketoglutarate | 0.698 | 0.049 | 1.353 | 0.310 | 0.649 | 0.071 | 1.578 | 0.152 |
| AMP | 1.417 | 0.165 | 0.971 | 0.984 | 0.857 | 0.395 | 0.653 | 0.071 |
| citrate | 0.806 | 0.373 | 1.546 | 0.206 | 1.051 | 0.756 | 1.823 | 0.029 |
| glucuronate | 0.668 | 0.011 | 1.248 | 0.395 | 1.261 | 0.178 | 1.341 | 0.206 |
| malate | 0.866 | 0.950 | 1.107 | 0.950 | 0.791 | 0.130 | 1.267 | 0.604 |
| PEP | 1.112 | 0.548 | 0.958 | 0.788 | 0.741 | 0.221 | 1.255 | 0.290 |
| succinate/methylmalonate | 0.904 | 0.788 | 1.739 | 0.027 | 1.211 | 0.633 | 1.543 | 0.373 |
| uracil | 0.560 | 0.120 | 1.745 | 0.152 | 1.378 | 0.290 | 2.024 | 0.085 |
| urate | 0.359 | 0.059 | 1.147 | 0.395 | 1.783 | 0.373 | 2.145 | 0.065 |
| xanthine | 0.522 | 0.093 | 1.702 | 0.065 | 1.411 | 0.330 | 2.017 | 0.165 |
| phosphocreatine | 1.633 | 0.443 | 1.767 | 0.152 | 0.953 | 0.663 | 1.154 | 0.548 |
| glycine | 1.183 | 0.520 | 0.971 | 0.820 | 0.799 | 0.443 | 1.389 | 0.141 |
| aspartate | 0.866 | 0.633 | 1.509 | 0.191 | 1.195 | 0.633 | 1.051 | 0.885 |
| serotonin | 0.383 | 0.085 | 0.516 | 0.093 | 1.729 | 0.663 | 1.701 | 0.494 |
| dimethylglycine | 0.740 | 0.330 | 1.616 | 0.054 | 1.271 | 0.663 | 1.774 | 0.065 |
| kynurenic acid | 0.661 | 0.788 | 2.563 | 0.040 | 1.123 | 0.820 | 2.657 | 0.085 |
| 5-adenosylhomocysteine | 0.909 | 0.852 | 0.881 | 0.724 | 1.056 | 0.917 | 0.945 | 0.917 |
| creatine | 1.579 | 0.036 | 1.279 | 0.419 | 0.852 | 0.443 | 0.942 | 0.820 |
| creatinine | 0.729 | 0.206 | 1.522 | 0.101 | 1.264 | 0.254 | 1.883 | 0.021 |
| thyroxine | 0.644 | 0.093 | 1.577 | 0.085 | 1.138 | 0.419 | 1.615 | 0.040 |
| cAMP | 1.076 | 0.694 | 1.186 | 0.520 | 0.877 | 0.724 | 0.578 | 0.130 |
| putrescine | 0.667 | 0.191 | 1.012 | 0.917 | 0.957 | 0.885 | 1.455 | 0.330 |
| arachidonyl_carnitine | 1.054 | 0.885 | 0.707 | 0.237 | 0.841 | 0.520 | 0.649 | 0.036 |
| 4-pyridoxate | 0.741 | 0.419 | 1.209 | 0.443 | 1.300 | 0.120 | 1.230 | 0.694 |
| uridine | 1.103 | 0.917 | 1.059 | 0.984 | 0.692 | 0.443 | 1.215 | 0.520 |
| taurocholate | 1.023 | 0.950 | 1.513 | 0.237 | 0.784 | 0.520 | 1.644 | 0.071 |
| glycodeoxycholate/glycochenodeoxycholate | 0.704 | 0.054 | 1.202 | 0.419 | 0.980 | 0.756 | 1.473 | 0.078 |
| 3-methyladipate/pimelate | 0.829 | 0.281 | 0.965 | 0.772 | 1.110 | 0.468 | 0.814 | 0.330 |
| arginine | 0.778 | 0.373 | 1.604 | 0.141 | 0.888 | 0.576 | 1.725 | 0.054 |
| anthranilic acid | 0.755 | 0.494 | 1.023 | 0.950 | 0.793 | 0.694 | 1.287 | 0.468 |
| hexoses (HILIC pos) | 0.771 | 0.468 | 1.652 | 0.254 | 1.112 | 0.756 | 2.032 | 0.078 |
| propionylcarnitine | 0.857 | 0.576 | 1.563 | 0.178 | 0.927 | 0.950 | 1.383 | 0.494 |

| **Metabolite** | **TBC1D20 FC** | **TBC1D20 P VALUE** | **TBC1D21 FC** | **TBC1D21 P VALUE** | **TBC1D22A FC** | **TBC1D22A P VALUE** | **TBC1D22B FC** | **TBC1D22B P VALUE** |
| --- | --- | --- | --- | --- | --- | --- | --- | --- |
| 1-methylnicotinamide | 0.721 | 0.885 | 2.177 | 0.637 | 32.796 | 0.004 | 46.212 | 0.003 |
| alpha-glycerophosphate | 1.123 | 0.694 | 0.860 | 0.610 | 0.597 | 0.237 | 0.665 | 0.206 |
| malonylcarnitine | 0.946 | 0.788 | 0.871 | 0.558 | 0.576 | 0.065 | 0.579 | 0.021 |
| erythrose-4-phosphate | 0.961 | 0.820 | 0.648 | 0.014 | 1.002 | 0.852 | 1.111 | 0.694 |
| N-carbamoyl-beta-alanine | 0.618 | 0.165 | 1.214 | 0.836 | 2.683 | 0.004 | 2.152 | 0.110 |
| hexanoylcarnitine | 0.905 | 0.788 | 0.770 | 0.396 | 0.642 | 0.093 | 0.550 | 0.036 |
| beta-alanine | 0.924 | 0.950 | 1.293 | 0.317 | 1.845 | 0.024 | 2.193 | 0.007 |
| 3-phosphoglycerate | 1.073 | 0.788 | 0.649 | 0.012 | 1.152 | 0.330 | 1.807 | 0.027 |
| inositol | 0.835 | 0.373 | 0.871 | 0.610 | 1.110 | 0.520 | 0.959 | 0.820 |
| phenylalanine | 1.256 | 0.633 | 1.379 | 0.558 | 1.061 | 0.604 | 1.315 | 0.468 |
| GABA | 1.317 | 0.633 | 3.029 | 0.086 | 1.858 | 0.237 | 2.274 | 0.071 |
| acetylcarnitine | 0.669 | 0.071 | 1.243 | 0.955 | 0.410 | 0.044 | 0.800 | 0.221 |
| butyrylcarnitine/isobutyrylcarnitine | 0.865 | 0.820 | 0.945 | 0.806 | 0.456 | 0.110 | 1.089 | 0.694 |
| myristoylcarnitine | 1.788 | 0.494 | 0.735 | 0.584 | 0.389 | 0.093 | 0.408 | 0.019 |
| 2-aminoadipate | 0.592 | 0.012 | 1.237 | 0.168 | 1.044 | 0.984 | 0.815 | 0.254 |
| DHAP/glyceraldehyde 3P | 1.322 | 0.443 | 0.934 | 0.637 | 1.676 | 0.036 | 2.425 | 0.002 |
| alanine | 1.027 | 0.468 | 0.881 | 0.282 | 1.001 | 0.633 | 1.473 | 0.373 |
| serine | 1.460 | 0.065 | 1.074 | 0.836 | 0.901 | 0.852 | 0.923 | 0.788 |
| glutamate | 1.361 | 0.351 | 1.224 | 0.317 | 1.672 | 0.027 | 1.581 | 0.059 |
| valine | 1.227 | 0.310 | 1.314 | 0.439 | 1.233 | 0.330 | 1.374 | 0.351 |
| lauroylcarnitine | 1.842 | 0.206 | 0.793 | 0.720 | 0.536 | 0.254 | 0.511 | 0.049 |
| cytidine | 1.593 | 0.351 | 1.281 | 0.584 | 0.300 | 0.005 | 0.398 | 0.044 |
| GMP | 1.156 | 0.520 | 0.719 | 0.193 | 1.038 | 0.756 | 1.157 | 0.576 |
| kynurenine | 0.765 | 0.633 | 1.260 | 0.317 | 1.282 | 0.351 | 1.077 | 0.820 |
| lactate | 1.003 | 0.576 | 0.971 | 0.637 | 1.529 | 0.036 | 1.599 | 0.017 |
| alpha-hydroxybutyrate | 0.750 | 0.191 | 1.203 | 0.206 | 1.077 | 0.633 | 0.774 | 0.152 |
| asparagine | 0.869 | 0.520 | 0.920 | 0.558 | 0.638 | 0.663 | 0.491 | 0.178 |
| lysine | 1.578 | 0.191 | 1.309 | 0.375 | 1.151 | 0.351 | 1.360 | 0.351 |
| tryptophan | 1.317 | 0.351 | 1.488 | 0.220 | 1.132 | 0.310 | 1.228 | 0.604 |
| 2-deoxycytidine | 0.853 | 0.756 | 1.322 | 0.584 | 0.191 | 0.000 | 0.250 | 0.008 |
| palmitoylcarnitine | 2.129 | 0.254 | 0.708 | 0.720 | 0.400 | 0.152 | 0.496 | 0.078 |
| guanosine | 0.691 | 0.351 | 0.911 | 0.748 | 0.685 | 0.191 | 0.825 | 0.548 |
| inosine | 1.142 | 0.633 | 1.196 | 0.355 | 0.760 | 0.290 | 0.753 | 0.237 |
| ribose-5-P/ribulose5-P | 1.124 | 0.756 | 0.930 | 0.509 | 1.699 | 0.152 | 1.854 | 0.040 |
| sorbitol | 1.096 | 0.756 | 1.113 | 0.462 | 1.095 | 0.152 | 1.330 | 0.330 |
| 2-hydroxyglutarate | 1.191 | 0.548 | 0.814 | 0.462 | 1.280 | 0.130 | 2.220 | 0.005 |
| threonine | 1.187 | 0.419 | 1.360 | 0.462 | 1.156 | 0.373 | 1.408 | 0.351 |
| isoleucine | 1.237 | 0.310 | 1.297 | 0.485 | 1.024 | 0.206 | 1.110 | 0.395 |
| 5-HIAA | 0.967 | 0.917 | 1.257 | 0.136 | 0.983 | 0.885 | 1.063 | 0.548 |
| trimethylamine-N-oxide | 0.927 | 0.917 | 1.081 | 0.417 | 1.106 | 0.576 | 1.060 | 0.221 |
| butyrobetaine | 0.906 | 0.310 | 1.172 | 0.806 | 0.650 | 0.101 | 0.919 | 0.395 |
| stearoylcarnitine | 1.628 | 0.373 | 1.040 | 0.925 | 0.372 | 0.165 | 0.470 | 0.130 |
| F1P/F6P/G1P/G6P | 0.890 | 0.395 | 0.723 | 0.146 | 1.007 | 0.917 | 1.284 | 0.237 |
| hexoses (HILIC neg) | 1.800 | 0.027 | 1.182 | 0.439 | 0.837 | 0.494 | 1.079 | 0.694 |
| hypoxanthine | 1.535 | 0.419 | 2.032 | 0.193 | 0.512 | 0.237 | 0.350 | 0.054 |
| NAD | 0.806 | 0.351 | 0.777 | 0.282 | 1.370 | 0.120 | 2.060 | 0.005 |
| NADP | 1.038 | 0.756 | 0.702 | 0.180 | 1.454 | 0.178 | 1.597 | 0.130 |
| methionine | 1.353 | 0.141 | 1.274 | 0.664 | 0.945 | 0.330 | 1.131 | 0.494 |
| glutamine | 1.362 | 0.330 | 1.230 | 0.558 | 0.986 | 0.468 | 0.932 | 0.663 |
| leucine | 1.400 | 0.206 | 1.322 | 0.396 | 1.016 | 0.290 | 1.152 | 0.419 |
| tyrosine | 1.014 | 0.756 | 1.326 | 0.462 | 1.047 | 0.330 | 1.159 | 0.494 |
| thiamine | 1.650 | 0.373 | 1.144 | 0.925 | 1.459 | 0.141 | 1.694 | 0.120 |
| niacinamide | 1.996 | 0.078 | 1.096 | 0.720 | 1.406 | 0.290 | 1.552 | 0.395 |
| alpha-glycerophosphocholine | 0.316 | 0.014 | 1.428 | 0.584 | 0.512 | 0.254 | 0.801 | 0.604 |
| thymidine | 0.869 | 0.917 | 1.078 | 0.692 | 0.715 | 0.468 | 0.837 | 0.820 |
| carnitine | 0.949 | 0.310 | 1.273 | 0.664 | 0.691 | 0.221 | 0.952 | 0.468 |
| oleylcarnitine | 1.185 | 0.788 | 0.964 | 0.895 | 0.417 | 0.141 | 0.492 | 0.130 |
| aconitate | 0.905 | 0.576 | 0.720 | 0.168 | 0.634 | 0.065 | 0.989 | 0.885 |
| CMP | 0.917 | 0.852 | 0.886 | 0.584 | 0.958 | 0.885 | 1.028 | 0.694 |
| cystathionine | 0.672 | 0.373 | 0.862 | 0.865 | 1.607 | 0.395 | 1.173 | 0.852 |
| fumarate/maleate/alpha-ketoisovalerate | 0.873 | 0.310 | 0.904 | 0.180 | 0.914 | 0.852 | 1.256 | 0.272 |
| glutathione oxidized | 0.990 | 0.724 | 1.047 | 0.955 | 2.257 | 0.049 | 1.866 | 0.101 |
| hippurate | 1.051 | 0.694 | 1.075 | 0.462 | 0.710 | 0.237 | 0.889 | 0.604 |
| pantothenate | 0.868 | 0.724 | 1.059 | 0.925 | 1.097 | 0.520 | 1.220 | 0.494 |
| sucrose | 1.229 | 0.221 | 0.989 | 0.985 | 1.069 | 0.633 | 0.912 | 0.950 |
| UMP | 1.095 | 0.917 | 0.667 | 0.109 | 0.998 | 0.820 | 0.829 | 0.351 |
| UDP-galactose/UDP-glucose | 0.968 | 0.852 | 0.813 | 0.336 | 1.790 | 0.036 | 2.077 | 0.033 |
| 6-phosphogluconate | 1.110 | 0.950 | 0.416 | 0.073 | 0.581 | 0.310 | 1.013 | 0.852 |
| histidine | 1.010 | 0.885 | 1.364 | 0.282 | 0.864 | 0.724 | 0.984 | 0.950 |
| proline | 1.114 | 0.520 | 1.082 | 0.925 | 0.760 | 0.468 | 1.026 | 0.852 |
| cis/trans-hydroxyproline | 1.482 | 0.290 | 0.843 | 0.664 | 0.761 | 0.885 | 0.652 | 0.206 |
| homocysteine | 0.843 | 0.395 | 0.702 | 0.220 | 1.398 | 0.272 | 2.338 | 0.015 |
| SDMA/ADMA | 1.213 | 0.694 | 1.173 | 0.509 | 0.826 | 0.468 | 1.023 | 0.548 |
| NMMA | 1.239 | 0.724 | 1.017 | 0.806 | 1.060 | 0.917 | 1.029 | 0.885 |
| carnosine | 1.312 | 0.419 | 1.244 | 0.692 | 0.594 | 0.443 | 0.991 | 0.694 |
| adenosine | 1.009 | 0.633 | 0.901 | 0.985 | 1.419 | 0.206 | 1.325 | 0.330 |
| 2-deoxyadenosine | 0.815 | 0.373 | 0.887 | 0.533 | 0.867 | 0.494 | 0.875 | 0.756 |
| cotinine | 0.623 | 0.071 | 1.125 | 0.692 | 1.331 | 0.351 | 1.251 | 0.468 |
| pipecolic acid | 0.589 | 0.206 | 1.065 | 0.558 | 1.241 | 0.950 | 0.523 | 0.093 |
| pyroglutamic acid | 1.604 | 0.101 | 1.137 | 0.925 | 1.027 | 0.443 | 1.314 | 0.373 |
| sarcosine | 0.853 | 0.633 | 0.726 | 0.299 | 1.037 | 0.820 | 1.063 | 0.548 |
| isocitrate | 0.785 | 0.221 | 0.810 | 0.396 | 0.829 | 0.290 | 1.190 | 0.178 |
| dCMP | 0.691 | 0.395 | 1.066 | 0.720 | 0.889 | 0.917 | 0.884 | 0.576 |
| glutathione reduced | 0.589 | 0.085 | 0.889 | 0.509 | 1.687 | 0.093 | 1.710 | 0.093 |
| lactose | 0.947 | 0.694 | 1.460 | 0.249 | 0.769 | 0.885 | 0.910 | 0.633 |
| oxalate | 0.989 | 0.756 | 1.006 | 0.985 | 0.984 | 0.917 | 1.071 | 0.254 |
| thymine | 1.147 | 0.576 | 1.110 | 0.692 | 0.854 | 0.604 | 0.541 | 0.027 |
| taurodeoxycholate/taurochenodeoxycholate | 3.877 | 0.178 | 0.907 | 0.692 | 1.552 | 0.576 | 1.085 | 0.548 |
| malondialdehyde | 0.829 | 0.165 | 0.896 | 0.220 | 1.091 | 0.481 | 1.140 | 0.330 |
| ornithine | 1.365 | 0.419 | 1.539 | 0.485 | 0.651 | 0.290 | 0.606 | 0.191 |
| citrulline | 1.226 | 0.290 | 1.215 | 0.439 | 0.783 | 0.468 | 0.835 | 0.788 |
| taurine | 0.628 | 0.110 | 2.097 | 0.057 | 0.960 | 0.852 | 1.006 | 0.917 |
| acetylglycine | 0.883 | 0.576 | 1.014 | 0.836 | 1.020 | 0.468 | 0.617 | 0.078 |
| allantoin | 1.268 | 0.272 | 1.070 | 0.584 | 0.860 | 0.351 | 0.934 | 0.443 |
| betaine | 1.296 | 0.272 | 1.336 | 0.079 | 0.766 | 0.254 | 0.876 | 0.101 |
| choline | 1.578 | 0.011 | 1.025 | 0.610 | 1.038 | 0.254 | 0.982 | 0.494 |
| acetylcholine | 1.114 | 0.756 | 0.216 | 0.057 | 0.735 | 0.756 | 2.698 | 0.310 |
| xanthosine | 1.407 | 0.221 | 1.086 | 0.462 | 1.075 | 0.820 | 1.138 | 0.885 |
| methionine sulfoxide | 1.579 | 0.059 | 1.023 | 0.985 | 0.842 | 0.852 | 1.140 | 0.351 |
| valerylcarnitine/isovalerylcarnitine/2-methylbutyroylcarnitine | 1.866 | 0.272 | 0.780 | 0.282 | 0.501 | 0.059 | 1.748 | 0.290 |
| heptanoylcarnitine | 1.060 | 0.984 | 0.843 | 0.375 | 1.098 | 0.520 | 0.752 | 0.110 |
| anserine | 1.055 | 0.494 | 1.079 | 0.485 | 0.790 | 0.418 | 0.900 | 0.950 |
| adenine | 0.650 | 0.178 | 0.865 | 0.664 | 0.571 | 0.033 | 0.754 | 0.604 |
| adipate | 0.987 | 0.917 | 1.031 | 0.509 | 0.921 | 0.885 | 0.958 | 0.788 |
| alpha-ketoglutarate | 0.861 | 0.272 | 0.675 | 0.062 | 1.250 | 0.468 | 1.677 | 0.078 |
| AMP | 1.018 | 0.756 | 0.835 | 0.533 | 0.983 | 0.756 | 0.855 | 0.788 |
| citrate | 0.741 | 0.093 | 0.786 | 0.355 | 0.806 | 0.272 | 1.023 | 0.950 |
| glucuronate | 1.025 | 0.788 | 1.009 | 0.925 | 1.090 | 0.788 | 1.234 | 0.290 |
| malate | 0.786 | 0.310 | 0.746 | 0.100 | 0.966 | 0.950 | 1.140 | 0.548 |
| PEP | 1.109 | 0.694 | 0.666 | 0.062 | 1.205 | 0.633 | 1.516 | 0.165 |
| succinate/methylmalonate | 1.193 | 0.272 | 1.244 | 0.282 | 0.713 | 0.110 | 0.907 | 0.520 |
| uracil | 1.155 | 0.373 | 1.090 | 0.462 | 0.749 | 0.290 | 0.728 | 0.130 |
| urate | 0.964 | 0.917 | 1.612 | 0.317 | 0.792 | 0.373 | 1.129 | 0.917 |
| xanthine | 1.187 | 0.468 | 0.991 | 0.509 | 0.697 | 0.221 | 0.776 | 0.221 |
| phosphocreatine | 1.412 | 0.548 | 0.815 | 0.417 | 0.625 | 0.330 | 1.318 | 0.373 |
| glycine | 1.056 | 0.917 | 1.028 | 0.439 | 1.221 | 0.468 | 1.238 | 0.443 |
| aspartate | 1.206 | 0.178 | 1.077 | 0.485 | 0.937 | 0.984 | 0.751 | 0.351 |
| serotonin | 0.459 | 0.152 | 1.153 | 0.777 | 0.422 | 0.085 | 0.531 | 0.272 |
| dimethylglycine | 1.142 | 0.576 | 1.176 | 0.439 | 0.744 | 0.237 | 0.869 | 0.494 |
| kynurenic acid | 1.402 | 0.165 | 1.068 | 0.720 | 0.571 | 0.141 | 0.813 | 0.633 |
| 5-adenosylhomocysteine | 0.878 | 0.373 | 1.193 | 0.836 | 0.899 | 0.576 | 1.375 | 0.071 |
| creatine | 1.648 | 0.078 | 1.099 | 0.806 | 0.814 | 0.468 | 1.221 | 0.494 |
| creatinine | 1.311 | 0.633 | 1.144 | 0.558 | 0.809 | 0.237 | 1.073 | 0.604 |
| thyroxine | 0.879 | 0.633 | 1.046 | 0.439 | 0.895 | 0.254 | 0.716 | 0.165 |
| cAMP | 0.554 | 0.101 | 0.817 | 0.193 | 0.730 | 0.351 | 0.670 | 0.120 |
| putrescine | 0.867 | 0.663 | 1.213 | 0.584 | 0.922 | 0.604 | 1.084 | 0.852 |
| arachidonyl_carnitine | 0.903 | 0.443 | 0.781 | 0.375 | 0.873 | 0.788 | 0.938 | 0.694 |
| 4-pyridoxate | 1.013 | 0.984 | 1.057 | 0.836 | 1.043 | 0.663 | 0.935 | 0.724 |
| uridine | 1.196 | 0.820 | 1.001 | 0.558 | 0.833 | 0.351 | 0.947 | 0.756 |
| taurocholate | 1.232 | 0.395 | 0.987 | 0.558 | 1.397 | 0.191 | 1.229 | 0.694 |
| glycodeoxycholate/glycochenodeoxycholate | 1.134 | 0.633 | 1.015 | 0.462 | 0.789 | 0.330 | 0.957 | 0.694 |
| 3-methyladipate/pimelate | 0.937 | 0.694 | 1.181 | 0.299 | 1.024 | 0.740 | 0.784 | 0.191 |
| arginine | 1.214 | 0.395 | 1.165 | 0.533 | 0.958 | 0.917 | 0.835 | 0.351 |
| anthranilic acid | 0.910 | 0.788 | 1.141 | 0.985 | 0.826 | 0.419 | 0.816 | 0.290 |
| hexoses (HILIC pos) | 1.678 | 0.065 | 1.144 | 0.558 | 1.073 | 0.443 | 1.013 | 0.885 |
| propionylcarnitine | 1.134 | 0.788 | 1.046 | 0.925 | 0.814 | 0.373 | 1.479 | 0.395 |

| **Metabolite** | **TBC1D23 FC** | **TBC1D23 P VALUE** | **TBC1D24 FC** | **TBC1D24 P VALUE** | **TBC1D25 FC** | **TBC1D25 P VALUE** | **TBC1D26 FC** | **TBC1D26 P VALUE** |
| --- | --- | --- | --- | --- | --- | --- | --- | --- |
| 1-methylnicotinamide | 13.785 | 0.036 | 0.027 | 0.004 | 3.327 | 0.395 | 1.234 | 0.756 |
| alpha-glycerophosphate | 0.831 | 0.520 | 2.188 | 0.101 | 1.857 | 0.191 | 0.406 | 0.015 |
| malonylcarnitine | 0.601 | 0.059 | 2.533 | 0.008 | 0.725 | 0.310 | 0.539 | 0.054 |
| erythrose-4-phosphate | 0.764 | 0.141 | 1.269 | 0.237 | 1.251 | 0.468 | 0.637 | 0.024 |
| N-carbamoyl-beta-alanine | 1.115 | 0.419 | 0.262 | 0.002 | 1.301 | 0.494 | 1.047 | 0.724 |
| hexanoylcarnitine | 0.755 | 0.120 | 2.310 | 0.021 | 0.825 | 0.395 | 0.689 | 0.351 |
| beta-alanine | 1.462 | 0.221 | 0.684 | 0.130 | 1.234 | 0.330 | 1.192 | 0.310 |
| 3-phosphoglycerate | 1.131 | 0.604 | 0.598 | 0.130 | 1.781 | 0.044 | 0.776 | 0.120 |
| inositol | 1.205 | 0.419 | 0.785 | 0.330 | 1.188 | 0.494 | 0.745 | 0.141 |
| phenylalanine | 0.767 | 0.520 | 0.725 | 0.152 | 0.917 | 0.852 | 2.006 | 0.017 |
| GABA | 1.132 | 0.395 | 0.572 | 0.120 | 0.848 | 0.820 | 1.948 | 0.059 |
| acetylcarnitine | 0.596 | 0.290 | 2.547 | 0.021 | 0.658 | 0.419 | 1.011 | 0.604 |
| butyrylcarnitine/isobutyrylcarnitine | 0.593 | 0.221 | 4.188 | 0.002 | 0.814 | 0.604 | 0.825 | 0.576 |
| myristoylcarnitine | 0.551 | 0.165 | 7.641 | 0.001 | 0.482 | 0.101 | 0.310 | 0.085 |
| 2-aminoadipate | 0.954 | 0.520 | 0.826 | 0.724 | 0.858 | 0.290 | 1.267 | 0.101 |
| DHAP/glyceraldehyde 3P | 1.751 | 0.021 | 0.566 | 0.036 | 2.390 | 0.005 | 0.846 | 0.351 |
| alanine | 0.688 | 0.395 | 1.006 | 0.604 | 0.661 | 0.221 | 1.212 | 0.694 |
| serine | 0.889 | 0.820 | 1.106 | 0.885 | 0.726 | 0.254 | 1.114 | 0.852 |
| glutamate | 1.343 | 0.330 | 0.713 | 0.059 | 1.515 | 0.120 | 1.500 | 0.029 |
| valine | 0.917 | 0.694 | 0.695 | 0.130 | 1.054 | 0.917 | 1.986 | 0.024 |
| lauroylcarnitine | 0.461 | 0.059 | 5.996 | 0.001 | 0.689 | 0.272 | 0.394 | 0.110 |
| cytidine | 0.772 | 0.443 | 2.834 | 0.024 | 0.501 | 0.130 | 1.019 | 0.820 |
| GMP | 1.179 | 0.548 | 1.315 | 0.191 | 0.926 | 0.576 | 0.649 | 0.027 |
| kynurenine | 0.695 | 0.290 | 0.753 | 0.310 | 0.961 | 0.756 | 1.231 | 0.468 |
| lactate | 1.397 | 0.120 | 0.613 | 0.065 | 1.209 | 0.443 | 0.865 | 0.633 |
| alpha-hydroxybutyrate | 0.855 | 0.351 | 0.869 | 0.494 | 0.769 | 0.165 | 1.372 | 0.036 |
| asparagine | 1.178 | 0.756 | 2.134 | 0.254 | 0.570 | 0.351 | 0.408 | 0.054 |
| lysine | 0.982 | 0.950 | 0.690 | 0.330 | 1.284 | 0.604 | 2.142 | 0.036 |
| tryptophan | 0.816 | 0.950 | 0.781 | 0.178 | 0.817 | 0.548 | 1.905 | 0.015 |
| 2-deoxycytidine | 0.459 | 0.101 | 3.175 | 0.029 | 0.499 | 0.206 | 0.799 | 0.576 |
| palmitoylcarnitine | 0.546 | 0.290 | 6.533 | 0.004 | 0.600 | 0.254 | 0.375 | 0.141 |
| guanosine | 0.686 | 0.165 | 0.666 | 0.178 | 0.828 | 0.468 | 1.299 | 0.548 |
| inosine | 0.763 | 0.694 | 0.975 | 0.694 | 0.983 | 0.885 | 1.269 | 0.310 |
| ribose-5-P/ribulose5-P | 1.058 | 0.756 | 0.622 | 0.165 | 1.729 | 0.101 | 0.723 | 0.165 |
| sorbitol | 1.018 | 0.604 | 0.720 | 0.120 | 1.185 | 0.852 | 1.407 | 0.101 |
| 2-hydroxyglutarate | 1.263 | 0.351 | 0.897 | 0.373 | 1.336 | 0.237 | 1.051 | 0.820 |
| threonine | 0.879 | 0.984 | 0.769 | 0.290 | 0.872 | 0.788 | 1.952 | 0.036 |
| isoleucine | 0.847 | 0.917 | 0.789 | 0.059 | 0.888 | 0.694 | 1.508 | 0.085 |
| 5-HIAA | 0.839 | 0.272 | 0.910 | 0.663 | 1.022 | 0.984 | 1.456 | 0.027 |
| trimethylamine-N-oxide | 1.015 | 0.820 | 0.959 | 0.984 | 0.948 | 0.984 | 1.041 | 0.694 |
| butyrobetaine | 0.792 | 0.494 | 1.518 | 0.178 | 0.825 | 0.724 | 1.135 | 0.756 |
| stearoylcarnitine | 0.544 | 0.254 | 4.873 | 0.005 | 0.578 | 0.221 | 0.525 | 0.351 |
| F1P/F6P/G1P/G6P | 0.882 | 0.576 | 1.097 | 0.724 | 1.313 | 0.548 | 0.745 | 0.191 |
| hexoses (HILIC neg) | 0.747 | 0.419 | 0.718 | 0.178 | 1.342 | 0.788 | 1.507 | 0.237 |
| hypoxanthine | 0.584 | 0.351 | 1.483 | 0.419 | 0.767 | 0.576 | 1.045 | 0.917 |
| NAD | 1.236 | 0.373 | 0.755 | 0.130 | 1.483 | 0.130 | 1.001 | 0.917 |
| NADP | 0.772 | 0.351 | 0.685 | 0.395 | 1.331 | 0.694 | 1.203 | 0.633 |
| methionine | 0.801 | 0.663 | 0.811 | 0.130 | 0.890 | 0.788 | 1.542 | 0.120 |
| glutamine | 0.989 | 0.756 | 1.093 | 0.468 | 1.042 | 0.950 | 1.132 | 0.494 |
| leucine | 0.896 | 0.852 | 0.874 | 0.152 | 0.996 | 0.820 | 1.544 | 0.078 |
| tyrosine | 0.782 | 0.663 | 0.718 | 0.101 | 0.797 | 0.373 | 1.867 | 0.021 |
| thiamine | 1.186 | 0.604 | 0.685 | 0.093 | 1.288 | 0.756 | 1.557 | 0.152 |
| niacinamide | 1.049 | 0.917 | 0.527 | 0.040 | 1.372 | 0.724 | 1.693 | 0.178 |
| alpha-glycerophosphocholine | 0.551 | 0.272 | 0.736 | 0.419 | 0.616 | 0.221 | 2.463 | 0.141 |
| thymidine | 1.201 | 0.494 | 0.632 | 0.395 | 0.811 | 0.633 | 1.212 | 0.548 |
| carnitine | 0.719 | 0.351 | 1.471 | 0.165 | 0.822 | 0.724 | 0.985 | 0.663 |
| oleylcarnitine | 0.610 | 0.330 | 4.620 | 0.007 | 0.451 | 0.152 | 0.471 | 0.254 |
| aconitate | 0.953 | 0.663 | 1.381 | 0.290 | 1.515 | 0.191 | 0.805 | 0.468 |
| CMP | 0.943 | 0.724 | 1.125 | 0.520 | 0.960 | 0.820 | 0.962 | 0.950 |
| cystathionine | 1.388 | 0.395 | 0.606 | 0.237 | 1.150 | 0.694 | 4.419 | 0.002 |
| fumarate/maleate/alpha-ketoisovalerate | 0.822 | 0.633 | 1.262 | 0.290 | 1.086 | 0.272 | 0.979 | 0.520 |
| glutathione oxidized | 1.271 | 0.984 | 0.713 | 0.373 | 1.890 | 0.093 | 0.953 | 0.984 |
| hippurate | 0.849 | 0.395 | 0.869 | 0.984 | 1.165 | 0.852 | 1.425 | 0.419 |
| pantothenate | 0.655 | 0.165 | 1.174 | 0.788 | 0.817 | 0.520 | 0.977 | 0.885 |
| sucrose | 1.035 | 0.663 | 0.912 | 0.373 | 1.670 | 0.044 | 1.002 | 0.756 |
| UMP | 0.818 | 0.468 | 1.262 | 0.494 | 0.828 | 0.310 | 0.680 | 0.085 |
| UDP-galactose/UDP-glucose | 1.659 | 0.290 | 0.783 | 0.178 | 1.641 | 0.419 | 1.048 | 0.917 |
| 6-phosphogluconate | 0.484 | 0.101 | 1.256 | 0.494 | 1.491 | 0.663 | 0.439 | 0.093 |
| histidine | 0.787 | 0.984 | 0.955 | 0.724 | 0.831 | 0.373 | 1.526 | 0.110 |
| proline | 0.907 | 0.917 | 1.263 | 0.694 | 0.844 | 0.548 | 0.828 | 0.141 |
| cis/trans-hydroxyproline | 1.222 | 0.548 | 2.012 | 0.206 | 0.999 | 0.917 | 0.442 | 0.049 |
| homocysteine | 1.203 | 0.633 | 0.793 | 0.885 | 1.607 | 0.395 | 0.991 | 0.917 |
| SDMA/ADMA | 0.925 | 0.694 | 1.088 | 0.604 | 0.983 | 0.576 | 1.260 | 0.548 |
| NMMA | 1.029 | 0.917 | 1.025 | 0.724 | 0.968 | 0.663 | 1.171 | 0.419 |
| carnosine | 0.612 | 0.395 | 1.652 | 0.065 | 0.735 | 0.443 | 1.283 | 0.663 |
| adenosine | 0.857 | 0.917 | 0.873 | 0.330 | 0.822 | 0.820 | 0.702 | 0.443 |
| 2-deoxyadenosine | 1.072 | 0.576 | 0.916 | 0.885 | 0.933 | 0.950 | 0.543 | 0.120 |
| cotinine | 1.261 | 0.468 | 0.845 | 0.604 | 0.963 | 0.756 | 1.028 | 0.788 |
| pipecolic acid | 1.236 | 0.756 | 0.831 | 0.885 | 0.964 | 0.494 | 0.831 | 0.950 |
| pyroglutamic acid | 0.954 | 0.950 | 0.807 | 0.373 | 1.274 | 0.604 | 1.266 | 0.663 |
| sarcosine | 1.097 | 0.272 | 1.287 | 0.468 | 0.952 | 0.950 | 1.028 | 0.820 |
| isocitrate | 1.081 | 0.917 | 1.003 | 0.756 | 1.639 | 0.141 | 1.016 | 0.917 |
| dCMP | 0.704 | 0.254 | 0.828 | 0.604 | 0.994 | 0.820 | 1.163 | 0.494 |
| glutathione reduced | 1.315 | 0.443 | 0.790 | 0.520 | 1.471 | 0.290 | 1.079 | 0.852 |
| lactose | 0.884 | 0.576 | 0.988 | 0.395 | 1.596 | 0.130 | 1.595 | 0.221 |
| oxalate | 0.936 | 0.917 | 1.007 | 0.984 | 0.941 | 0.984 | 1.069 | 0.756 |
| thymine | 0.716 | 0.395 | 1.384 | 0.330 | 0.546 | 0.093 | 0.828 | 0.520 |
| taurodeoxycholate/taurochenodeoxycholate | 1.826 | 0.756 | 0.396 | 0.237 | 2.352 | 0.820 | 0.754 | 0.663 |
| malondialdehyde | 0.990 | 0.724 | 0.822 | 0.561 | 1.001 | 0.917 | 0.932 | 0.351 |
| ornithine | 0.828 | 0.917 | 1.324 | 0.443 | 0.874 | 0.917 | 1.010 | 0.724 |
| citrulline | 1.001 | 0.788 | 0.913 | 0.724 | 1.053 | 0.852 | 1.388 | 0.206 |
| taurine | 0.809 | 0.820 | 1.276 | 0.950 | 0.872 | 0.950 | 0.970 | 0.820 |
| acetylglycine | 0.797 | 0.310 | 1.214 | 0.468 | 0.687 | 0.071 | 0.771 | 0.237 |
| allantoin | 0.899 | 0.520 | 0.883 | 0.885 | 1.003 | 0.548 | 1.330 | 0.272 |
| betaine | 0.687 | 0.130 | 1.429 | 0.152 | 0.822 | 0.165 | 1.279 | 0.178 |
| choline | 0.982 | 0.852 | 1.011 | 0.756 | 1.021 | 0.756 | 1.134 | 0.254 |
| acetylcholine | 0.509 | 0.395 | 2.798 | 0.237 | 1.033 | 0.820 | 0.475 | 0.395 |
| xanthosine | 0.869 | 0.494 | 0.776 | 0.576 | 0.902 | 0.520 | 1.105 | 0.494 |
| methionine sulfoxide | 0.789 | 0.852 | 1.035 | 0.633 | 0.885 | 0.576 | 1.029 | 0.395 |
| valerylcarnitine/isovalerylcarnitine/2-methylbutyroylcarnitine | 0.781 | 0.419 | 1.514 | 0.330 | 1.598 | 0.237 | 1.055 | 0.984 |
| heptanoylcarnitine | 1.203 | 0.724 | 1.287 | 0.254 | 0.771 | 0.152 | 0.798 | 0.494 |
| anserine | 0.942 | 0.917 | 1.075 | 0.350 | 0.937 | 0.788 | 1.119 | 0.548 |
| adenine | 0.795 | 0.663 | 1.391 | 0.395 | 0.861 | 0.917 | 0.893 | 0.950 |
| adipate | 0.874 | 0.633 | 1.047 | 0.694 | 0.920 | 0.272 | 1.145 | 0.254 |
| alpha-ketoglutarate | 1.064 | 0.852 | 0.839 | 0.443 | 1.564 | 0.059 | 0.783 | 0.191 |
| AMP | 1.093 | 0.852 | 1.607 | 0.101 | 0.779 | 0.419 | 0.610 | 0.078 |
| citrate | 1.025 | 0.395 | 1.044 | 0.917 | 1.665 | 0.120 | 0.971 | 0.604 |
| glucuronate | 1.119 | 0.520 | 0.757 | 0.178 | 1.285 | 0.165 | 1.207 | 0.395 |
| malate | 0.993 | 0.852 | 1.071 | 0.604 | 1.230 | 0.221 | 0.922 | 0.351 |
| PEP | 0.912 | 0.468 | 0.700 | 0.351 | 1.399 | 0.165 | 0.751 | 0.110 |
| succinate/methylmalonate | 1.004 | 0.756 | 1.070 | 0.395 | 1.031 | 0.852 | 1.311 | 0.290 |
| uracil | 0.814 | 0.468 | 1.017 | 0.852 | 0.973 | 0.724 | 1.248 | 0.576 |
| urate | 0.537 | 0.290 | 0.813 | 0.917 | 1.524 | 0.221 | 1.953 | 0.191 |
| xanthine | 0.927 | 0.663 | 0.926 | 0.885 | 0.919 | 0.663 | 1.464 | 0.373 |
| phosphocreatine | 1.115 | 0.984 | 2.031 | 0.468 | 1.832 | 0.178 | 0.817 | 0.395 |
| glycine | 1.018 | 0.885 | 0.984 | 0.984 | 0.829 | 0.272 | 1.011 | 0.494 |
| aspartate | 1.075 | 0.852 | 1.435 | 0.290 | 0.990 | 0.950 | 1.048 | 0.443 |
| serotonin | 1.300 | 0.633 | 0.711 | 0.663 | 0.862 | 0.694 | 1.468 | 0.310 |
| dimethylglycine | 0.791 | 0.395 | 1.035 | 0.663 | 0.948 | 0.520 | 1.358 | 0.191 |
| kynurenic acid | 0.900 | 0.917 | 1.152 | 0.548 | 1.083 | 0.984 | 1.628 | 0.221 |
| 5-adenosylhomocysteine | 0.767 | 0.576 | 1.426 | 0.576 | 0.878 | 0.663 | 1.249 | 0.221 |
| creatine | 1.036 | 0.885 | 1.534 | 0.178 | 1.113 | 0.468 | 0.768 | 0.494 |
| creatinine | 0.908 | 0.788 | 0.956 | 0.756 | 1.157 | 0.576 | 1.212 | 0.443 |
| thyroxine | 0.995 | 0.788 | 1.051 | 0.694 | 0.997 | 0.633 | 0.968 | 0.724 |
| cAMP | 0.737 | 0.443 | 1.183 | 0.633 | 0.930 | 0.576 | 1.243 | 0.724 |
| putrescine | 0.751 | 0.330 | 0.722 | 0.494 | 1.109 | 0.756 | 1.235 | 0.443 |
| arachidonyl_carnitine | 0.620 | 0.059 | 1.140 | 0.468 | 1.297 | 0.604 | 0.809 | 0.494 |
| 4-pyridoxate | 0.861 | 0.663 | 0.932 | 0.756 | 0.738 | 0.494 | 0.885 | 0.852 |
| uridine | 1.218 | 0.520 | 0.891 | 0.820 | 0.833 | 0.468 | 1.064 | 0.917 |
| taurocholate | 1.365 | 0.395 | 0.648 | 0.093 | 1.154 | 0.984 | 1.023 | 0.756 |
| glycodeoxycholate/glycochenodeoxycholate | 0.787 | 0.351 | 0.997 | 0.756 | 1.037 | 0.788 | 1.077 | 0.351 |
| 3-methyladipate/pimelate | 0.888 | 0.694 | 0.972 | 0.950 | 0.793 | 0.229 | 1.123 | 0.604 |
| arginine | 1.080 | 0.917 | 1.049 | 0.917 | 0.958 | 0.756 | 0.845 | 0.520 |
| anthranilic acid | 1.303 | 0.443 | 1.011 | 0.820 | 1.275 | 0.419 | 1.101 | 0.917 |
| hexoses (HILIC pos) | 0.872 | 0.633 | 0.718 | 0.178 | 1.175 | 0.724 | 1.073 | 0.885 |
| propionylcarnitine | 0.935 | 0.950 | 0.886 | 0.820 | 1.060 | 0.694 | 1.112 | 0.917 |

| **Metabolite** | **TBC1D28 FC** | **TBC1D28 P VALUE** | **TBC1D2B FC** | **TBC1D2B P VALUE** | **TBC1D3 FC** | **TBC1D3 P VALUE** | **TBC1D30 FC** | **TBC1D30 P VALUE** |
| --- | --- | --- | --- | --- | --- | --- | --- | --- |
| 1-methylnicotinamide | 3.505 | 0.272 | 1.425 | 0.724 | 0.124 | 0.071 | 0.007 | <.0001 |
| alpha-glycerophosphate | 0.405 | 0.027 | 0.758 | 0.468 | 2.803 | 0.017 | 2.722 | 0.017 |
| malonylcarnitine | 0.425 | 0.011 | 1.367 | 0.443 | 2.716 | 0.007 | 2.987 | 0.000 |
| erythrose-4-phosphate | 0.661 | 0.026 | 1.197 | 0.431 | 1.169 | 0.330 | 1.567 | 0.005 |
| N-carbamoyl-beta-alanine | 0.952 | 0.756 | 1.389 | 0.310 | 0.398 | 0.033 | 0.235 | 0.002 |
| hexanoylcarnitine | 0.498 | 0.054 | 0.786 | 0.178 | 2.512 | 0.011 | 2.899 | 0.001 |
| beta-alanine | 1.309 | 0.290 | 1.337 | 0.373 | 1.023 | 0.852 | 0.467 | 0.005 |
| 3-phosphoglycerate | 0.821 | 0.152 | 0.846 | 0.395 | 0.801 | 0.604 | 0.851 | 0.917 |
| inositol | 0.777 | 0.395 | 1.201 | 0.237 | 0.595 | 0.006 | 0.623 | 0.015 |
| phenylalanine | 1.543 | 0.065 | 1.781 | 0.049 | 1.200 | 0.852 | 1.149 | 0.917 |
| GABA | 1.930 | 0.093 | 0.695 | 0.419 | 0.722 | 0.419 | 0.207 | 0.004 |
| acetylcarnitine | 0.946 | 0.468 | 1.027 | 0.820 | 2.257 | 0.049 | 2.322 | 0.015 |
| butyrylcarnitine/isobutyrylcarnitine | 0.658 | 0.254 | 0.914 | 0.820 | 3.619 | 0.012 | 2.729 | 0.040 |
| myristoylcarnitine | 0.430 | 0.254 | 2.319 | 0.178 | 1.475 | 0.254 | 7.742 | 0.001 |
| 2-aminoadipate | 1.422 | 0.027 | 1.100 | 0.576 | 0.920 | 0.520 | 0.621 | 0.014 |
| DHAP/glyceraldehyde 3P | 0.873 | 0.351 | 1.067 | 0.885 | 0.771 | 0.494 | 0.716 | 0.165 |
| alanine | 1.076 | 0.756 | 1.680 | 0.237 | 1.314 | 0.576 | 1.566 | 0.178 |
| serine | 1.092 | 0.820 | 1.153 | 0.548 | 1.211 | 0.984 | 1.500 | 0.040 |
| glutamate | 1.278 | 0.101 | 1.196 | 0.351 | 1.040 | 0.694 | 0.571 | 0.010 |
| valine | 1.500 | 0.120 | 1.701 | 0.059 | 1.147 | 0.694 | 1.064 | 0.885 |
| lauroylcarnitine | 0.379 | 0.101 | 1.636 | 0.290 | 2.246 | 0.110 | 5.377 | 0.005 |
| cytidine | 1.162 | 0.520 | 0.605 | 0.272 | 0.824 | 0.604 | 2.043 | 0.237 |
| GMP | 0.688 | 0.085 | 1.431 | 0.071 | 1.158 | 0.419 | 2.307 | 0.001 |
| kynurenine | 1.482 | 0.152 | 0.892 | 0.950 | 0.630 | 0.237 | 0.459 | 0.033 |
| lactate | 0.730 | 0.221 | 1.218 | 0.152 | 0.986 | 0.756 | 0.669 | 0.191 |
| alpha-hydroxybutyrate | 1.279 | 0.165 | 0.968 | 0.756 | 0.988 | 0.984 | 0.741 | 0.152 |
| asparagine | 0.692 | 0.395 | 0.876 | 0.950 | 1.037 | 0.984 | 2.088 | 0.071 |
| lysine | 1.521 | 0.221 | 1.641 | 0.078 | 1.108 | 0.788 | 1.240 | 0.756 |
| tryptophan | 1.491 | 0.085 | 1.626 | 0.101 | 1.271 | 0.520 | 1.037 | 0.820 |
| 2-deoxycytidine | 0.798 | 0.633 | 0.642 | 0.310 | 1.633 | 0.494 | 2.286 | 0.272 |
| palmitoylcarnitine | 0.515 | 0.351 | 1.914 | 0.330 | 1.347 | 0.330 | 7.723 | 0.002 |
| guanosine | 0.746 | 0.272 | 0.773 | 0.373 | 0.622 | 0.078 | 1.281 | 0.310 |
| inosine | 0.865 | 0.604 | 1.175 | 0.604 | 1.189 | 0.494 | 1.216 | 0.548 |
| ribose-5-P/ribulose5-P | 0.616 | 0.059 | 1.106 | 0.984 | 1.345 | 0.443 | 0.800 | 0.950 |
| sorbitol | 1.310 | 0.272 | 1.185 | 0.395 | 1.084 | 0.984 | 0.728 | 0.040 |
| 2-hydroxyglutarate | 1.377 | 0.576 | 0.784 | 0.520 | 0.677 | 0.101 | 0.738 | 0.152 |
| threonine | 1.607 | 0.071 | 1.761 | 0.093 | 1.320 | 0.604 | 1.342 | 0.254 |
| isoleucine | 1.352 | 0.178 | 1.545 | 0.054 | 1.098 | 0.756 | 1.008 | 0.443 |
| 5-HIAA | 1.462 | 0.019 | 1.123 | 0.548 | 0.623 | 0.040 | 0.872 | 0.494 |
| trimethylamine-N-oxide | 1.205 | 0.178 | 0.944 | 0.885 | 0.891 | 0.468 | 0.764 | 0.085 |
| butyrobetaine | 0.924 | 0.604 | 0.920 | 0.419 | 2.345 | 0.002 | 1.551 | 0.110 |
| stearoylcarnitine | 0.869 | 0.950 | 1.649 | 0.373 | 0.894 | 0.694 | 3.602 | 0.029 |
| F1P/F6P/G1P/G6P | 0.757 | 0.272 | 0.945 | 0.694 | 1.235 | 0.419 | 1.593 | 0.152 |
| hexoses (HILIC neg) | 1.139 | 0.604 | 1.093 | 0.330 | 0.913 | 0.468 | 0.971 | 0.694 |
| hypoxanthine | 0.575 | 0.191 | 1.191 | 0.724 | 0.834 | 0.917 | 2.412 | 0.078 |
| NAD | 0.932 | 0.694 | 1.271 | 0.443 | 1.336 | 0.272 | 1.087 | 0.852 |
| NADP | 1.212 | 0.694 | 1.150 | 0.633 | 0.849 | 0.984 | 1.456 | 0.237 |
| methionine | 1.317 | 0.419 | 1.637 | 0.054 | 1.119 | 0.694 | 1.265 | 0.820 |
| glutamine | 0.933 | 0.950 | 1.317 | 0.633 | 1.948 | 0.049 | 1.621 | 0.120 |
| leucine | 1.309 | 0.272 | 1.553 | 0.065 | 1.171 | 0.756 | 1.061 | 0.548 |
| tyrosine | 1.568 | 0.054 | 1.632 | 0.078 | 1.136 | 0.984 | 1.118 | 0.885 |
| thiamine | 1.167 | 0.373 | 1.539 | 0.373 | 1.531 | 0.351 | 0.879 | 0.373 |
| niacinamide | 1.117 | 0.633 | 1.552 | 0.178 | 1.119 | 0.917 | 0.939 | 0.724 |
| alpha-glycerophosphocholine | 1.498 | 0.885 | 0.290 | 0.017 | 1.591 | 0.178 | 0.678 | 0.576 |
| thymidine | 0.871 | 0.885 | 0.838 | 0.724 | 1.019 | 0.694 | 0.693 | 0.395 |
| carnitine | 0.927 | 0.373 | 0.986 | 0.694 | 1.998 | 0.008 | 1.409 | 0.191 |
| oleylcarnitine | 0.716 | 0.604 | 2.077 | 0.191 | 0.882 | 0.852 | 4.215 | 0.017 |
| aconitate | 0.852 | 0.633 | 0.938 | 0.468 | 1.227 | 0.520 | 1.404 | 0.310 |
| CMP | 1.048 | 0.724 | 0.992 | 0.852 | 0.912 | 0.604 | 1.222 | 0.310 |
| cystathionine | 3.986 | 0.007 | 0.639 | 0.351 | 1.422 | 0.724 | 0.982 | 0.917 |
| fumarate/maleate/alpha-ketoisovalerate | 1.037 | 0.852 | 1.073 | 0.694 | 1.495 | 0.049 | 0.996 | 0.694 |
| glutathione oxidized | 0.931 | 0.694 | 1.442 | 0.178 | 0.822 | 0.520 | 0.937 | 0.852 |
| hippurate | 1.195 | 0.820 | 1.060 | 0.548 | 0.982 | 0.494 | 1.205 | 0.548 |
| pantothenate | 1.012 | 0.984 | 1.422 | 0.152 | 1.143 | 0.852 | 1.035 | 0.788 |
| sucrose | 0.877 | 0.576 | 1.749 | 0.044 | 0.687 | 0.373 | 0.930 | 0.663 |
| UMP | 0.714 | 0.191 | 1.304 | 0.351 | 1.385 | 0.165 | 1.655 | 0.029 |
| UDP-galactose/UDP-glucose | 1.054 | 0.984 | 1.694 | 0.351 | 1.095 | 0.917 | 0.818 | 0.085 |
| 6-phosphogluconate | 0.450 | 0.093 | 0.977 | 0.950 | 1.015 | 0.917 | 1.929 | 0.165 |
| histidine | 1.615 | 0.049 | 1.536 | 0.085 | 1.015 | 0.548 | 1.328 | 0.520 |
| proline | 0.831 | 0.044 | 1.251 | 0.221 | 1.057 | 0.917 | 1.141 | 0.885 |
| cis/trans-hydroxyproline | 0.547 | 0.191 | 0.809 | 0.724 | 1.054 | 0.694 | 1.186 | 0.272 |
| homocysteine | 1.298 | 0.520 | 0.940 | 0.694 | 0.751 | 0.917 | 0.795 | 0.604 |
| SDMA/ADMA | 1.060 | 0.885 | 1.306 | 0.152 | 1.169 | 0.917 | 1.306 | 0.237 |
| NMMA | 0.853 | 0.663 | 1.601 | 0.120 | 1.127 | 0.885 | 1.413 | 0.178 |
| carnosine | 1.184 | 0.950 | 1.218 | 0.468 | 1.369 | 0.191 | 0.928 | 0.788 |
| adenosine | 0.649 | 0.254 | 1.760 | 0.443 | 0.622 | 0.373 | 0.986 | 0.756 |
| 2-deoxyadenosine | 0.482 | 0.065 | 0.528 | 0.141 | 1.300 | 0.548 | 1.332 | 0.419 |
| cotinine | 1.208 | 0.724 | 1.039 | 0.756 | 0.788 | 0.633 | 0.490 | 0.007 |
| pipecolic acid | 0.823 | 0.984 | 1.833 | 0.093 | 1.094 | 0.885 | 0.639 | 0.330 |
| pyroglutamic acid | 1.024 | 0.756 | 1.249 | 0.254 | 1.323 | 0.724 | 1.079 | 0.604 |
| sarcosine | 1.157 | 0.548 | 0.695 | 0.152 | 1.223 | 0.520 | 1.189 | 0.548 |
| isocitrate | 1.015 | 0.885 | 0.902 | 0.351 | 1.186 | 0.443 | 1.122 | 0.950 |
| dCMP | 1.262 | 0.351 | 0.736 | 0.548 | 0.770 | 0.395 | 0.695 | 0.206 |
| glutathione reduced | 1.174 | 0.724 | 1.155 | 0.852 | 0.994 | 0.885 | 0.742 | 0.419 |
| lactose | 1.246 | 0.494 | 1.174 | 0.604 | 0.539 | 0.191 | 0.926 | 0.984 |
| oxalate | 1.169 | 0.290 | 0.874 | 0.633 | 0.906 | 0.663 | 0.799 | 0.130 |
| thymine | 0.910 | 0.724 | 1.216 | 0.330 | 1.175 | 0.788 | 0.841 | 0.290 |
| taurodeoxycholate/taurochenodeoxycholate | 0.449 | 0.373 | 0.512 | 0.885 | 0.791 | 0.633 | 0.416 | 0.724 |
| malondialdehyde | 0.894 | 0.290 | 1.259 | 0.078 | 1.087 | 0.724 | 0.742 | 0.071 |
| ornithine | 0.976 | 0.694 | 0.819 | 0.468 | 1.291 | 0.604 | 1.194 | 0.468 |
| citrulline | 1.082 | 0.820 | 1.057 | 0.756 | 1.033 | 0.756 | 1.246 | 0.237 |
| taurine | 1.019 | 0.917 | 1.067 | 0.917 | 1.911 | 0.130 | 0.876 | 0.548 |
| acetylglycine | 0.648 | 0.049 | 0.964 | 0.950 | 1.312 | 0.290 | 1.383 | 0.130 |
| allantoin | 1.091 | 0.663 | 1.159 | 0.576 | 1.194 | 0.788 | 1.029 | 0.395 |
| betaine | 1.077 | 0.820 | 1.059 | 0.917 | 1.677 | 0.015 | 1.229 | 0.419 |
| choline | 1.134 | 0.221 | 1.378 | 0.141 | 0.906 | 0.788 | 0.989 | 0.984 |
| acetylcholine | 0.456 | 0.373 | 0.824 | 0.852 | 1.857 | 0.351 | 1.699 | 0.419 |
| xanthosine | 0.892 | 0.756 | 1.126 | 0.694 | 1.065 | 0.885 | 0.893 | 0.604 |
| methionine sulfoxide | 1.063 | 0.330 | 1.253 | 0.548 | 0.883 | 0.694 | 1.049 | 0.820 |
| valerylcarnitine/isovalerylcarnitine/2-methylbutyroylcarnitine | 1.161 | 0.788 | 0.708 | 0.290 | 1.472 | 0.443 | 2.519 | 0.033 |
| heptanoylcarnitine | 0.725 | 0.254 | 1.057 | 0.576 | 1.084 | 0.852 | 1.550 | 0.078 |
| anserine | 1.010 | 0.984 | 0.989 | 0.820 | 1.019 | 0.756 | 0.849 | 0.494 |
| adenine | 0.661 | 0.310 | 0.739 | 0.178 | 0.626 | 0.101 | 0.666 | 0.178 |
| adipate | 1.265 | 0.049 | 1.004 | 0.663 | 0.969 | 0.604 | 0.929 | 0.548 |
| alpha-ketoglutarate | 0.750 | 0.085 | 1.463 | 0.141 | 1.210 | 0.443 | 0.906 | 0.443 |
| AMP | 0.652 | 0.152 | 0.972 | 0.852 | 1.602 | 0.065 | 1.613 | 0.027 |
| citrate | 0.954 | 0.663 | 1.155 | 0.852 | 1.254 | 0.494 | 1.238 | 0.576 |
| glucuronate | 0.995 | 0.917 | 1.152 | 0.419 | 0.854 | 0.178 | 0.779 | 0.272 |
| malate | 0.949 | 0.419 | 1.179 | 0.885 | 1.487 | 0.101 | 1.156 | 0.724 |
| PEP | 0.735 | 0.101 | 0.850 | 0.395 | 0.855 | 0.604 | 1.287 | 0.059 |
| succinate/methylmalonate | 1.155 | 0.694 | 0.985 | 0.950 | 1.072 | 0.604 | 1.365 | 0.078 |
| uracil | 1.079 | 0.950 | 1.153 | 0.633 | 1.280 | 0.604 | 1.235 | 0.330 |
| urate | 1.365 | 0.548 | 1.233 | 0.330 | 0.633 | 0.178 | 1.001 | 0.917 |
| xanthine | 1.155 | 0.917 | 1.059 | 0.694 | 1.235 | 0.885 | 1.134 | 0.468 |
| phosphocreatine | 0.718 | 0.221 | 1.383 | 0.694 | 1.344 | 0.885 | 2.105 | 0.221 |
| glycine | 0.907 | 0.820 | 1.275 | 0.093 | 1.061 | 0.917 | 1.142 | 0.254 |
| aspartate | 1.023 | 0.272 | 1.175 | 0.788 | 1.329 | 0.191 | 1.297 | 0.520 |
| serotonin | 1.520 | 0.206 | 1.045 | 0.885 | 0.778 | 0.351 | 1.159 | 0.756 |
| dimethylglycine | 1.133 | 0.724 | 1.055 | 0.468 | 1.099 | 0.820 | 1.111 | 0.330 |
| kynurenic acid | 1.394 | 0.548 | 1.282 | 0.694 | 0.944 | 0.950 | 1.304 | 0.443 |
| 5-adenosylhomocysteine | 1.228 | 0.178 | 1.351 | 0.663 | 1.126 | 0.917 | 1.111 | 0.694 |
| creatine | 0.717 | 0.395 | 1.051 | 0.694 | 1.128 | 0.494 | 1.057 | 0.520 |
| creatinine | 0.953 | 0.576 | 1.282 | 0.633 | 1.066 | 0.984 | 0.965 | 0.950 |
| thyroxine | 0.901 | 0.984 | 1.333 | 0.221 | 0.800 | 0.221 | 0.933 | 0.820 |
| cAMP | 1.089 | 0.694 | 0.671 | 0.165 | 1.323 | 0.468 | 1.079 | 0.852 |
| putrescine | 1.060 | 0.820 | 0.760 | 0.330 | 1.047 | 0.724 | 0.622 | 0.178 |
| arachidonyl_carnitine | 0.870 | 0.548 | 1.096 | 0.663 | 1.022 | 0.373 | 1.199 | 0.443 |
| 4-pyridoxate | 0.805 | 0.604 | 1.010 | 0.820 | 0.922 | 0.604 | 0.994 | 0.694 |
| uridine | 0.950 | 0.917 | 0.619 | 0.191 | 0.850 | 0.820 | 1.162 | 0.756 |
| taurocholate | 0.988 | 0.633 | 0.798 | 0.254 | 0.984 | 0.788 | 0.811 | 0.059 |
| glycodeoxycholate/glycochenodeoxycholate | 0.955 | 0.852 | 1.121 | 0.633 | 0.741 | 0.237 | 0.977 | 0.724 |
| 3-methyladipate/pimelate | 1.165 | 0.395 | 1.124 | 0.281 | 1.017 | 0.984 | 0.892 | 0.604 |
| arginine | 0.963 | 0.984 | 1.145 | 0.468 | 0.961 | 0.604 | 1.269 | 0.419 |
| anthranilic acid | 1.310 | 0.633 | 1.022 | 0.984 | 0.784 | 0.548 | 0.806 | 0.694 |
| hexoses (HILIC pos) | 0.775 | 0.548 | 1.362 | 0.351 | 0.801 | 0.724 | 0.925 | 0.633 |
| propionylcarnitine | 0.625 | 0.110 | 0.777 | 0.443 | 1.849 | 0.078 | 1.634 | 0.152 |

| **Metabolite** | **TBC1D31 FC** | **TBC1D31 P VALUE** | **TBC1D32 FC** | **TBC1D32 P VALUE** | **TBC1D3E FC** | **TBC1D3E P VALUE** | **TBC1D3G FC** | **TBC1D3G P VALUE** |
| --- | --- | --- | --- | --- | --- | --- | --- | --- |
| 1-methylnicotinamide | 7.811 | 0.044 | 0.858 | 0.820 | 0.738 | 0.950 | 0.804 | 0.820 |
| alpha-glycerophosphate | 0.984 | 0.917 | 1.034 | 0.984 | 1.555 | 0.237 | 0.474 | 0.093 |
| malonylcarnitine | 0.764 | 0.290 | 1.330 | 0.520 | 1.219 | 0.373 | 0.934 | 0.984 |
| erythrose-4-phosphate | 1.117 | 0.950 | 1.022 | 0.633 | 0.766 | 0.263 | 0.607 | 0.049 |
| N-carbamoyl-beta-alanine | 1.463 | 0.152 | 0.536 | 0.468 | 1.046 | 0.984 | 1.727 | 0.351 |
| hexanoylcarnitine | 0.683 | 0.165 | 1.241 | 0.548 | 1.024 | 0.520 | 0.845 | 0.820 |
| beta-alanine | 1.372 | 0.093 | 0.939 | 0.756 | 1.082 | 0.694 | 0.925 | 0.820 |
| 3-phosphoglycerate | 1.159 | 0.724 | 0.718 | 0.206 | 1.064 | 0.950 | 1.004 | 0.663 |
| inositol | 1.515 | 0.021 | 1.131 | 0.663 | 1.156 | 0.756 | 1.034 | 0.820 |
| phenylalanine | 0.969 | 0.984 | 0.730 | 0.254 | 1.057 | 0.885 | 1.296 | 0.330 |
| GABA | 2.983 | 0.008 | 1.123 | 0.885 | 0.543 | 0.120 | 0.448 | 0.078 |
| acetylcarnitine | 0.595 | 0.078 | 0.974 | 0.663 | 0.973 | 0.984 | 1.052 | 0.756 |
| butyrylcarnitine/isobutyrylcarnitine | 0.645 | 0.254 | 0.602 | 0.443 | 1.037 | 0.820 | 0.665 | 0.351 |
| myristoylcarnitine | 1.272 | 0.663 | 2.181 | 0.059 | 0.745 | 0.820 | 0.506 | 0.351 |
| 2-aminoadipate | 1.312 | 0.141 | 1.136 | 0.290 | 0.963 | 0.694 | 1.055 | 0.852 |
| DHAP/glyceraldehyde 3P | 1.474 | 0.206 | 0.679 | 0.290 | 0.791 | 0.520 | 0.720 | 0.237 |
| alanine | 0.959 | 0.788 | 0.792 | 0.419 | 1.135 | 0.604 | 1.352 | 0.120 |
| serine | 1.015 | 0.443 | 1.190 | 0.221 | 1.252 | 0.443 | 1.373 | 0.272 |
| glutamate | 1.142 | 0.494 | 0.850 | 0.820 | 1.160 | 0.917 | 0.717 | 0.141 |
| valine | 0.902 | 0.694 | 0.682 | 0.152 | 1.079 | 0.724 | 1.261 | 0.395 |
| lauroylcarnitine | 0.724 | 0.443 | 1.309 | 0.443 | 0.788 | 0.788 | 0.470 | 0.254 |
| cytidine | 0.751 | 0.576 | 1.117 | 0.788 | 1.195 | 0.852 | 1.302 | 0.788 |
| GMP | 1.081 | 0.984 | 1.177 | 0.520 | 0.945 | 0.950 | 0.883 | 0.756 |
| kynurenine | 0.819 | 0.548 | 0.876 | 0.788 | 0.559 | 0.071 | 0.943 | 0.694 |
| lactate | 1.375 | 0.110 | 0.618 | 0.110 | 1.217 | 0.548 | 1.248 | 0.419 |
| alpha-hydroxybutyrate | 0.795 | 0.221 | 1.190 | 0.221 | 0.998 | 0.788 | 1.125 | 0.917 |
| asparagine | 2.023 | 0.044 | 2.190 | 0.065 | 1.263 | 0.576 | 1.139 | 0.724 |
| lysine | 0.989 | 0.885 | 0.692 | 0.310 | 1.171 | 0.576 | 1.259 | 0.576 |
| tryptophan | 0.858 | 0.576 | 0.707 | 0.206 | 1.141 | 0.419 | 1.387 | 0.101 |
| 2-deoxycytidine | 0.779 | 0.663 | 1.334 | 0.468 | 1.427 | 0.373 | 1.453 | 0.373 |
| palmitoylcarnitine | 0.885 | 0.852 | 2.058 | 0.178 | 0.681 | 0.917 | 0.449 | 0.310 |
| guanosine | 0.942 | 0.820 | 0.936 | 0.885 | 0.912 | 0.633 | 1.912 | 0.033 |
| inosine | 0.898 | 0.724 | 1.024 | 0.885 | 0.864 | 0.852 | 0.984 | 0.984 |
| ribose-5-P/ribulose5-P | 1.249 | 0.468 | 0.747 | 0.395 | 1.145 | 0.604 | 0.905 | 0.694 |
| sorbitol | 1.058 | 0.633 | 0.771 | 0.694 | 1.338 | 0.373 | 1.269 | 0.419 |
| 2-hydroxyglutarate | 1.681 | 0.059 | 0.836 | 0.548 | 0.974 | 0.548 | 0.719 | 0.237 |
| threonine | 0.790 | 0.443 | 0.795 | 0.494 | 1.040 | 0.788 | 1.378 | 0.191 |
| isoleucine | 1.171 | 0.310 | 0.898 | 0.548 | 1.192 | 0.494 | 1.366 | 0.165 |
| 5-HIAA | 0.903 | 0.756 | 0.911 | 0.788 | 0.679 | 0.065 | 0.964 | 0.820 |
| trimethylamine-N-oxide | 0.874 | 0.576 | 0.973 | 0.852 | 0.845 | 0.254 | 0.963 | 0.468 |
| butyrobetaine | 0.657 | 0.033 | 1.179 | 0.419 | 1.475 | 0.078 | 1.179 | 0.373 |
| stearoylcarnitine | 0.963 | 0.852 | 1.840 | 0.395 | 0.466 | 0.494 | 0.453 | 0.254 |
| F1P/F6P/G1P/G6P | 1.027 | 0.724 | 0.953 | 0.724 | 0.810 | 0.443 | 0.706 | 0.272 |
| hexoses (HILIC neg) | 1.216 | 0.468 | 0.807 | 0.694 | 1.242 | 0.917 | 1.303 | 0.576 |
| hypoxanthine | 0.826 | 0.576 | 2.276 | 0.071 | 0.930 | 0.984 | 0.969 | 0.885 |
| NAD | 1.244 | 0.272 | 0.755 | 0.237 | 1.036 | 0.756 | 0.903 | 0.950 |
| NADP | 1.378 | 0.395 | 0.760 | 0.494 | 1.002 | 0.820 | 0.900 | 0.756 |
| methionine | 1.184 | 0.443 | 0.918 | 0.633 | 1.196 | 0.443 | 1.372 | 0.191 |
| glutamine | 0.977 | 0.756 | 0.833 | 0.820 | 1.401 | 0.165 | 1.245 | 0.165 |
| leucine | 1.118 | 0.494 | 0.850 | 0.395 | 1.242 | 0.419 | 1.225 | 0.468 |
| tyrosine | 1.005 | 0.917 | 0.868 | 0.494 | 1.086 | 0.724 | 1.427 | 0.165 |
| thiamine | 1.189 | 0.443 | 0.655 | 0.191 | 1.361 | 0.351 | 1.175 | 0.373 |
| niacinamide | 1.148 | 0.548 | 0.627 | 0.548 | 1.357 | 0.395 | 1.452 | 0.191 |
| alpha-glycerophosphocholine | 0.790 | 0.576 | 1.011 | 0.633 | 1.752 | 0.141 | 2.159 | 0.036 |
| thymidine | 0.564 | 0.093 | 0.624 | 0.290 | 1.210 | 0.548 | 1.605 | 0.373 |
| carnitine | 0.711 | 0.101 | 1.087 | 0.950 | 1.232 | 0.468 | 1.153 | 0.694 |
| oleylcarnitine | 1.483 | 0.604 | 2.367 | 0.120 | 0.513 | 0.310 | 0.563 | 0.330 |
| aconitate | 0.982 | 0.885 | 0.846 | 0.494 | 1.206 | 0.237 | 0.827 | 0.788 |
| CMP | 1.053 | 0.950 | 1.218 | 0.443 | 0.860 | 0.373 | 0.839 | 0.330 |
| cystathionine | 0.598 | 0.373 | 0.636 | 0.468 | 1.195 | 0.468 | 1.077 | 0.520 |
| fumarate/maleate/alpha-ketoisovalerate | 1.055 | 0.885 | 0.794 | 0.141 | 0.964 | 0.820 | 0.693 | 0.040 |
| glutathione oxidized | 1.552 | 0.310 | 0.891 | 0.694 | 0.852 | 0.576 | 0.664 | 0.191 |
| hippurate | 1.041 | 0.788 | 0.780 | 0.468 | 1.150 | 0.950 | 1.282 | 0.663 |
| pantothenate | 0.817 | 0.468 | 0.515 | 0.021 | 0.852 | 0.494 | 0.942 | 0.633 |
| sucrose | 0.932 | 0.788 | 0.819 | 0.885 | 0.739 | 0.756 | 0.693 | 0.494 |
| UMP | 1.054 | 0.885 | 1.214 | 0.663 | 0.980 | 0.885 | 0.835 | 0.443 |
| UDP-galactose/UDP-glucose | 1.440 | 0.221 | 0.845 | 0.237 | 1.076 | 0.917 | 0.700 | 0.520 |
| 6-phosphogluconate | 1.129 | 0.694 | 0.527 | 0.165 | 0.757 | 0.520 | 0.729 | 0.520 |
| histidine | 1.004 | 0.984 | 0.982 | 0.984 | 0.970 | 0.663 | 1.210 | 0.520 |
| proline | 1.472 | 0.015 | 0.912 | 0.788 | 1.196 | 0.756 | 1.136 | 0.395 |
| cis/trans-hydroxyproline | 1.832 | 0.065 | 1.299 | 0.152 | 1.373 | 0.468 | 0.805 | 0.950 |
| homocysteine | 1.093 | 0.984 | 0.580 | 0.040 | 0.618 | 0.290 | 0.756 | 0.443 |
| SDMA/ADMA | 1.119 | 0.604 | 0.838 | 0.576 | 1.233 | 0.494 | 1.236 | 0.237 |
| NMMA | 1.122 | 0.917 | 0.826 | 0.604 | 1.358 | 0.272 | 1.258 | 0.254 |
| carnosine | 1.131 | 0.756 | 1.087 | 0.604 | 1.550 | 0.351 | 1.007 | 0.468 |
| adenosine | 0.906 | 0.633 | 0.477 | 0.044 | 0.484 | 0.130 | 0.950 | 0.917 |
| 2-deoxyadenosine | 0.888 | 0.788 | 0.712 | 0.548 | 1.358 | 0.395 | 1.298 | 0.633 |
| cotinine | 0.893 | 0.694 | 0.779 | 0.395 | 0.891 | 0.950 | 1.154 | 0.468 |
| pipecolic acid | 0.766 | 0.520 | 1.247 | 0.820 | 1.193 | 0.724 | 1.303 | 0.520 |
| pyroglutamic acid | 1.206 | 0.254 | 0.671 | 0.373 | 1.686 | 0.191 | 1.406 | 0.373 |
| sarcosine | 0.668 | 0.130 | 0.824 | 0.395 | 1.117 | 0.663 | 1.039 | 0.788 |
| isocitrate | 1.089 | 0.984 | 0.949 | 0.272 | 1.059 | 0.756 | 0.790 | 0.633 |
| dCMP | 0.898 | 0.694 | 0.955 | 0.576 | 0.666 | 0.040 | 0.845 | 0.191 |
| glutathione reduced | 1.442 | 0.290 | 0.779 | 0.443 | 0.911 | 0.852 | 0.795 | 0.520 |
| lactose | 1.223 | 0.141 | 0.601 | 0.330 | 0.778 | 0.820 | 0.688 | 0.694 |
| oxalate | 0.934 | 0.494 | 0.884 | 0.604 | 0.784 | 0.059 | 0.886 | 0.178 |
| thymine | 0.915 | 0.663 | 1.305 | 0.272 | 1.245 | 0.468 | 1.306 | 0.373 |
| taurodeoxycholate/taurochenodeoxycholate | 2.296 | 0.330 | 1.083 | 0.917 | 3.031 | 0.191 | 2.075 | 0.059 |
| malondialdehyde | 1.157 | 0.330 | 0.778 | 0.152 | 0.983 | 0.663 | 1.097 | 0.885 |
| ornithine | 1.234 | 0.395 | 1.304 | 0.330 | 1.654 | 0.178 | 1.219 | 0.604 |
| citrulline | 1.107 | 0.917 | 1.078 | 0.604 | 1.381 | 0.351 | 1.411 | 0.221 |
| taurine | 0.705 | 0.330 | 1.127 | 0.852 | 1.564 | 0.310 | 1.241 | 0.373 |
| acetylglycine | 0.803 | 0.351 | 1.103 | 0.576 | 1.250 | 0.351 | 1.560 | 0.152 |
| allantoin | 1.023 | 0.820 | 0.765 | 0.604 | 1.596 | 0.093 | 1.523 | 0.078 |
| betaine | 0.677 | 0.071 | 0.952 | 0.917 | 1.241 | 0.152 | 1.119 | 0.290 |
| choline | 1.043 | 0.604 | 0.993 | 0.633 | 0.898 | 0.663 | 0.909 | 0.820 |
| acetylcholine | 0.427 | 0.272 | 0.736 | 0.633 | 1.575 | 0.633 | 0.592 | 0.494 |
| xanthosine | 1.027 | 0.885 | 0.870 | 0.820 | 1.396 | 0.152 | 1.490 | 0.120 |
| methionine sulfoxide | 1.317 | 0.110 | 0.967 | 0.788 | 1.047 | 0.468 | 1.103 | 0.290 |
| valerylcarnitine/isovalerylcarnitine/2-methylbutyroylcarnitine | 0.807 | 0.548 | 1.075 | 0.820 | 1.378 | 0.351 | 0.878 | 0.820 |
| heptanoylcarnitine | 1.019 | 0.852 | 1.221 | 0.419 | 0.889 | 0.950 | 1.028 | 0.576 |
| anserine | 1.133 | 0.663 | 1.250 | 0.330 | 1.339 | 0.191 | 1.095 | 0.917 |
| adenine | 0.982 | 0.724 | 1.376 | 0.548 | 0.888 | 0.663 | 0.717 | 0.290 |
| adipate | 1.042 | 0.290 | 0.937 | 0.724 | 0.964 | 0.885 | 1.037 | 0.663 |
| alpha-ketoglutarate | 1.053 | 0.663 | 0.860 | 0.663 | 0.981 | 0.984 | 0.734 | 0.206 |
| AMP | 0.811 | 0.237 | 1.228 | 0.548 | 1.292 | 0.178 | 1.029 | 0.756 |
| citrate | 0.967 | 0.419 | 0.985 | 0.395 | 1.178 | 0.633 | 0.865 | 0.633 |
| glucuronate | 1.281 | 0.141 | 0.769 | 0.330 | 0.900 | 0.290 | 0.914 | 0.330 |
| malate | 1.202 | 0.694 | 0.929 | 0.373 | 1.015 | 0.984 | 0.727 | 0.065 |
| PEP | 1.179 | 0.494 | 0.875 | 0.548 | 0.894 | 0.633 | 0.979 | 0.820 |
| succinate/methylmalonate | 1.233 | 0.419 | 1.217 | 0.330 | 1.328 | 0.576 | 1.192 | 0.950 |
| uracil | 0.990 | 0.984 | 1.072 | 0.633 | 1.417 | 0.191 | 1.339 | 0.310 |
| urate | 1.335 | 0.520 | 0.739 | 0.468 | 0.671 | 0.373 | 0.796 | 0.604 |
| xanthine | 1.124 | 0.788 | 1.080 | 0.788 | 1.745 | 0.152 | 1.598 | 0.254 |
| phosphocreatine | 1.196 | 0.548 | 1.062 | 0.633 | 1.445 | 0.548 | 0.518 | 0.085 |
| glycine | 1.036 | 0.604 | 1.023 | 0.548 | 1.218 | 0.419 | 1.347 | 0.093 |
| aspartate | 1.095 | 0.468 | 1.140 | 0.373 | 1.235 | 0.419 | 0.799 | 0.351 |
| serotonin | 1.205 | 0.820 | 1.795 | 0.633 | 0.746 | 0.165 | 1.856 | 0.254 |
| dimethylglycine | 0.925 | 0.984 | 0.927 | 0.917 | 1.419 | 0.237 | 1.437 | 0.071 |
| kynurenic acid | 1.288 | 0.694 | 1.148 | 0.724 | 1.595 | 0.130 | 1.549 | 0.351 |
| 5-adenosylhomocysteine | 1.000 | 0.663 | 0.753 | 0.093 | 0.769 | 0.237 | 0.738 | 0.468 |
| creatine | 1.180 | 0.885 | 0.867 | 0.494 | 1.288 | 0.351 | 0.743 | 0.351 |
| creatinine | 1.088 | 0.820 | 0.910 | 0.724 | 1.395 | 0.093 | 1.124 | 0.330 |
| thyroxine | 1.240 | 0.419 | 0.981 | 0.694 | 1.121 | 0.576 | 1.192 | 0.272 |
| cAMP | 0.810 | 0.548 | 0.719 | 0.520 | 1.078 | 0.351 | 0.938 | 0.820 |
| putrescine | 1.244 | 0.443 | 0.980 | 0.852 | 1.298 | 0.395 | 1.318 | 0.443 |
| arachidonyl_carnitine | 0.980 | 0.885 | 0.731 | 0.237 | 0.616 | 0.085 | 0.707 | 0.165 |
| 4-pyridoxate | 0.832 | 0.419 | 0.783 | 0.756 | 1.107 | 0.633 | 1.333 | 0.373 |
| uridine | 0.738 | 0.468 | 1.107 | 0.756 | 1.536 | 0.206 | 1.882 | 0.110 |
| taurocholate | 1.274 | 0.221 | 0.927 | 0.633 | 1.082 | 0.373 | 1.233 | 0.419 |
| glycodeoxycholate/glycochenodeoxycholate | 1.252 | 0.395 | 0.884 | 0.468 | 0.936 | 0.917 | 0.941 | 0.852 |
| 3-methyladipate/pimelate | 1.061 | 0.548 | 1.116 | 0.395 | 0.887 | 0.330 | 0.959 | 0.852 |
| arginine | 1.377 | 0.071 | 1.060 | 0.788 | 1.171 | 0.633 | 1.225 | 0.330 |
| anthranilic acid | 1.310 | 0.494 | 0.903 | 0.756 | 0.929 | 0.984 | 1.052 | 0.852 |
| hexoses (HILIC pos) | 1.341 | 0.694 | 0.938 | 0.984 | 1.196 | 0.724 | 1.375 | 0.330 |
| propionylcarnitine | 0.512 | 0.059 | 0.701 | 0.165 | 1.570 | 0.178 | 1.612 | 0.101 |

| **Metabolite** | **TBC1D4 FC** | **TBC1D4 P VALUE** | **TBC1D5 FC** | **TBC1D5 P VALUE** | **TBC1D7 FC** | **TBC1D7 P VALUE** | **TBC1D8 FC** | **TBC1D8 P VALUE** |
| --- | --- | --- | --- | --- | --- | --- | --- | --- |
| 1-methylnicotinamide | 4.590 | 0.351 | 0.860 | 0.724 | 0.957 | 0.950 | 1.335 | 0.633 |
| alpha-glycerophosphate | 0.787 | 0.330 | 0.966 | 0.548 | 1.880 | 0.206 | 0.906 | 0.852 |
| malonylcarnitine | 0.779 | 0.191 | 0.725 | 0.395 | 1.706 | 0.130 | 0.987 | 0.520 |
| erythrose-4-phosphate | 0.944 | 0.787 | 0.925 | 0.443 | 1.388 | 0.085 | 1.282 | 0.221 |
| N-carbamoyl-beta-alanine | 1.207 | 0.576 | 1.123 | 0.576 | 1.059 | 0.820 | 1.677 | 0.272 |
| hexanoylcarnitine | 0.754 | 0.165 | 0.500 | 0.054 | 0.945 | 0.950 | 0.922 | 0.468 |
| beta-alanine | 0.968 | 0.852 | 2.143 | 0.004 | 1.410 | 0.141 | 0.985 | 0.852 |
| 3-phosphoglycerate | 0.708 | 0.101 | 0.700 | 0.029 | 1.556 | 0.120 | 0.909 | 0.917 |
| inositol | 1.182 | 0.330 | 1.516 | 0.040 | 0.746 | 0.130 | 0.910 | 0.950 |
| phenylalanine | 0.822 | 0.576 | 1.928 | 0.044 | 1.855 | 0.130 | 1.199 | 0.548 |
| GABA | 1.128 | 0.852 | 1.331 | 0.604 | 2.067 | 0.330 | 0.652 | 0.852 |
| acetylcarnitine | 0.772 | 0.520 | 0.837 | 0.395 | 1.834 | 0.178 | 0.748 | 0.120 |
| butyrylcarnitine/isobutyrylcarnitine | 0.489 | 0.206 | 1.088 | 0.984 | 3.763 | 0.008 | 0.414 | 0.093 |
| myristoylcarnitine | 0.747 | 0.520 | 0.628 | 0.468 | 0.833 | 0.604 | 2.024 | 0.237 |
| 2-aminoadipate | 1.133 | 0.494 | 1.198 | 0.351 | 0.830 | 0.165 | 0.929 | 0.950 |
| DHAP/glyceraldehyde 3P | 1.063 | 0.548 | 0.940 | 0.520 | 1.609 | 0.120 | 1.235 | 0.788 |
| alanine | 0.764 | 0.351 | 0.978 | 0.852 | 1.807 | 0.054 | 1.442 | 0.310 |
| serine | 0.849 | 0.663 | 1.088 | 0.950 | 1.102 | 0.633 | 1.530 | 0.024 |
| glutamate | 1.077 | 0.756 | 1.823 | 0.005 | 1.321 | 0.272 | 0.824 | 0.443 |
| valine | 0.994 | 0.852 | 1.940 | 0.036 | 1.539 | 0.178 | 1.172 | 0.443 |
| lauroylcarnitine | 0.548 | 0.130 | 0.536 | 0.237 | 1.000 | 0.950 | 1.328 | 0.852 |
| cytidine | 0.505 | 0.054 | 1.444 | 0.310 | 0.512 | 0.206 | 0.582 | 0.330 |
| GMP | 1.147 | 0.494 | 0.734 | 0.254 | 1.328 | 0.221 | 1.079 | 0.724 |
| kynurenine | 1.136 | 0.788 | 1.366 | 0.254 | 1.155 | 0.604 | 0.891 | 0.885 |
| lactate | 0.958 | 0.885 | 0.871 | 0.633 | 1.388 | 0.071 | 1.011 | 0.984 |
| alpha-hydroxybutyrate | 1.180 | 0.237 | 1.074 | 0.520 | 0.793 | 0.254 | 0.918 | 0.885 |
| asparagine | 0.771 | 0.373 | 0.564 | 0.351 | 0.601 | 0.272 | 1.492 | 0.036 |
| lysine | 0.918 | 0.950 | 2.290 | 0.014 | 1.399 | 0.520 | 1.299 | 0.373 |
| tryptophan | 0.838 | 0.663 | 1.746 | 0.036 | 1.691 | 0.206 | 1.116 | 0.788 |
| 2-deoxycytidine | 0.538 | 0.101 | 1.053 | 0.885 | 0.866 | 0.724 | 0.661 | 0.272 |
| palmitoylcarnitine | 0.826 | 0.694 | 0.540 | 0.373 | 0.856 | 0.663 | 2.452 | 0.221 |
| guanosine | 1.277 | 0.206 | 1.201 | 0.756 | 0.778 | 0.443 | 0.768 | 0.604 |
| inosine | 1.387 | 0.221 | 1.093 | 0.576 | 1.060 | 0.885 | 0.933 | 0.443 |
| ribose-5-P/ribulose5-P | 0.920 | 0.984 | 0.998 | 0.756 | 2.571 | 0.010 | 0.916 | 0.917 |
| sorbitol | 0.715 | 0.984 | 1.380 | 0.351 | 1.309 | 0.663 | 1.043 | 0.694 |
| 2-hydroxyglutarate | 0.937 | 0.494 | 0.773 | 0.310 | 0.783 | 0.788 | 1.804 | 0.078 |
| threonine | 0.975 | 0.917 | 1.918 | 0.024 | 1.613 | 0.178 | 1.152 | 0.548 |
| isoleucine | 0.808 | 0.724 | 1.715 | 0.029 | 1.460 | 0.604 | 1.334 | 0.165 |
| 5-HIAA | 0.964 | 0.788 | 1.159 | 0.395 | 0.961 | 0.950 | 0.808 | 0.419 |
| trimethylamine-N-oxide | 1.058 | 0.724 | 0.971 | 0.950 | 0.878 | 0.576 | 0.842 | 0.272 |
| butyrobetaine | 0.806 | 0.310 | 0.971 | 0.984 | 1.394 | 0.290 | 0.668 | 0.019 |
| stearoylcarnitine | 0.639 | 0.373 | 0.614 | 0.663 | 0.797 | 0.548 | 1.865 | 0.272 |
| F1P/F6P/G1P/G6P | 1.061 | 0.852 | 0.692 | 0.054 | 1.602 | 0.049 | 1.238 | 0.310 |
| hexoses (HILIC neg) | 0.762 | 0.820 | 1.562 | 0.310 | 1.328 | 0.663 | 1.382 | 0.078 |
| hypoxanthine | 2.376 | 0.078 | 1.095 | 0.820 | 0.630 | 0.221 | 1.210 | 0.576 |
| NAD | 1.421 | 0.221 | 1.201 | 0.576 | 1.604 | 0.071 | 1.080 | 0.885 |
| NADP | 0.667 | 0.395 | 1.211 | 0.494 | 1.801 | 0.059 | 1.538 | 0.120 |
| methionine | 0.822 | 0.724 | 1.641 | 0.054 | 1.544 | 0.395 | 1.470 | 0.130 |
| glutamine | 0.883 | 0.788 | 1.516 | 0.395 | 1.696 | 0.494 | 1.099 | 0.950 |
| leucine | 0.853 | 0.788 | 1.810 | 0.015 | 1.454 | 0.494 | 1.277 | 0.290 |
| tyrosine | 0.841 | 0.724 | 1.811 | 0.059 | 1.579 | 0.272 | 1.245 | 0.351 |
| thiamine | 0.984 | 0.885 | 2.102 | 0.036 | 1.901 | 0.110 | 1.277 | 0.395 |
| niacinamide | 0.938 | 0.950 | 1.657 | 0.165 | 1.557 | 0.272 | 1.476 | 0.152 |
| alpha-glycerophosphocholine | 0.906 | 0.885 | 1.084 | 0.788 | 1.046 | 0.494 | 0.421 | 0.085 |
| thymidine | 1.066 | 0.820 | 1.261 | 0.443 | 0.750 | 0.351 | 0.487 | 0.049 |
| carnitine | 0.742 | 0.178 | 0.957 | 0.694 | 1.635 | 0.085 | 0.658 | 0.014 |
| oleylcarnitine | 0.808 | 0.443 | 0.695 | 0.663 | 0.753 | 0.520 | 2.027 | 0.310 |
| aconitate | 0.614 | 0.049 | 1.225 | 0.443 | 1.672 | 0.065 | 0.755 | 0.178 |
| CMP | 0.976 | 0.604 | 1.057 | 0.520 | 0.951 | 0.694 | 1.059 | 0.984 |
| cystathionine | 0.609 | 0.373 | 1.700 | 0.351 | 0.742 | 0.330 | 0.664 | 0.351 |
| fumarate/maleate/alpha-ketoisovalerate | 0.768 | 0.059 | 1.272 | 0.237 | 1.764 | 0.012 | 0.879 | 0.254 |
| glutathione oxidized | 1.020 | 0.852 | 1.550 | 0.395 | 2.092 | 0.008 | 1.177 | 0.663 |
| hippurate | 0.734 | 0.494 | 1.044 | 0.788 | 0.944 | 0.633 | 1.278 | 0.272 |
| pantothenate | 0.990 | 0.885 | 0.893 | 0.852 | 1.938 | 0.071 | 1.386 | 0.191 |
| sucrose | 1.257 | 0.254 | 0.979 | 0.950 | 0.941 | 0.885 | 1.237 | 0.724 |
| UMP | 0.905 | 0.663 | 0.843 | 0.468 | 1.324 | 0.290 | 1.007 | 0.820 |
| UDP-galactose/UDP-glucose | 1.236 | 0.917 | 2.250 | 0.059 | 1.562 | 0.071 | 0.988 | 0.520 |
| 6-phosphogluconate | 0.523 | 0.152 | 0.468 | 0.017 | 1.623 | 0.272 | 1.185 | 0.604 |
| histidine | 0.777 | 0.788 | 1.844 | 0.040 | 1.320 | 0.724 | 1.311 | 0.290 |
| proline | 0.871 | 0.604 | 1.197 | 0.395 | 1.174 | 0.443 | 1.131 | 0.852 |
| cis/trans-hydroxyproline | 0.798 | 0.548 | 0.582 | 0.254 | 0.725 | 0.663 | 1.191 | 0.120 |
| homocysteine | 0.870 | 0.520 | 1.254 | 0.468 | 1.932 | 0.044 | 0.869 | 0.520 |
| SDMA/ADMA | 0.800 | 0.468 | 1.255 | 0.373 | 1.191 | 0.852 | 1.208 | 0.443 |
| NMMA | 0.908 | 0.950 | 1.420 | 0.191 | 1.124 | 0.885 | 1.334 | 0.373 |
| carnosine | 0.830 | 0.950 | 1.990 | 0.049 | 1.481 | 0.494 | 0.995 | 0.950 |
| adenosine | 1.620 | 0.468 | 0.404 | 0.093 | 1.159 | 0.633 | 1.601 | 0.443 |
| 2-deoxyadenosine | 0.875 | 0.724 | 0.819 | 0.290 | 2.082 | 0.093 | 0.783 | 0.548 |
| cotinine | 1.258 | 0.443 | 0.719 | 0.520 | 0.951 | 0.788 | 0.981 | 0.694 |
| pipecolic acid | 0.907 | 0.663 | 1.625 | 0.130 | 1.328 | 0.101 | 0.356 | 0.004 |
| pyroglutamic acid | 0.730 | 0.917 | 1.787 | 0.093 | 1.506 | 0.520 | 1.242 | 0.165 |
| sarcosine | 0.678 | 0.110 | 0.801 | 0.395 | 1.195 | 0.310 | 0.994 | 0.984 |
| isocitrate | 0.817 | 0.221 | 1.253 | 0.520 | 1.469 | 0.059 | 0.835 | 0.272 |
| dCMP | 0.611 | 0.206 | 0.868 | 0.885 | 1.062 | 0.419 | 0.796 | 0.351 |
| glutathione reduced | 1.108 | 0.724 | 1.516 | 0.221 | 1.838 | 0.059 | 1.017 | 0.984 |
| lactose | 0.870 | 0.984 | 1.125 | 0.694 | 1.087 | 0.443 | 1.357 | 0.820 |
| oxalate | 0.981 | 0.885 | 0.904 | 0.633 | 1.024 | 0.756 | 0.900 | 0.724 |
| thymine | 0.657 | 0.272 | 0.969 | 0.852 | 1.021 | 0.548 | 0.588 | 0.059 |
| taurodeoxycholate/taurochenodeoxycholate | 1.566 | 0.756 | 0.625 | 0.604 | 0.414 | 0.419 | 4.028 | 0.015 |
| malondialdehyde | 0.917 | 0.934 | 1.044 | 0.576 | 1.339 | 0.085 | 0.848 | 0.199 |
| ornithine | 0.633 | 0.310 | 1.492 | 0.419 | 0.672 | 0.178 | 1.088 | 0.820 |
| citrulline | 0.824 | 0.724 | 1.501 | 0.310 | 1.133 | 0.820 | 1.271 | 0.351 |
| taurine | 0.699 | 0.395 | 2.204 | 0.040 | 1.636 | 0.330 | 0.440 | 0.008 |
| acetylglycine | 1.027 | 0.984 | 0.968 | 0.663 | 1.088 | 0.663 | 1.017 | 0.852 |
| allantoin | 0.715 | 0.290 | 1.165 | 0.724 | 1.115 | 0.984 | 1.212 | 0.221 |
| betaine | 0.861 | 0.604 | 1.204 | 0.272 | 1.448 | 0.330 | 0.887 | 0.141 |
| choline | 0.920 | 0.788 | 1.135 | 0.373 | 1.132 | 0.950 | 1.127 | 0.443 |
| acetylcholine | 0.973 | 0.950 | 0.594 | 0.468 | 1.437 | 0.419 | 1.224 | 0.788 |
| xanthosine | 0.786 | 0.395 | 0.817 | 0.694 | 0.922 | 0.885 | 1.215 | 0.820 |
| methionine sulfoxide | 0.800 | 0.520 | 1.082 | 0.633 | 1.416 | 0.494 | 1.283 | 0.093 |
| valerylcarnitine/isovalerylcarnitine/2-methylbutyroylcarnitine | 0.660 | 0.141 | 0.812 | 0.443 | 1.373 | 0.395 | 0.958 | 0.576 |
| heptanoylcarnitine | 1.144 | 0.950 | 0.743 | 0.494 | 0.686 | 0.221 | 1.541 | 0.351 |
| anserine | 0.989 | 0.984 | 1.539 | 0.033 | 1.016 | 0.820 | 0.890 | 0.576 |
| adenine | 1.383 | 0.310 | 1.303 | 0.221 | 0.697 | 0.468 | 0.762 | 0.310 |
| adipate | 0.893 | 0.788 | 1.118 | 0.330 | 0.927 | 0.254 | 1.068 | 0.395 |
| alpha-ketoglutarate | 0.913 | 0.756 | 1.331 | 0.373 | 1.550 | 0.059 | 0.862 | 0.494 |
| AMP | 1.012 | 0.917 | 0.694 | 0.191 | 1.244 | 0.237 | 0.809 | 0.373 |
| citrate | 0.871 | 0.237 | 1.221 | 0.885 | 1.478 | 0.059 | 0.882 | 0.178 |
| glucuronate | 0.971 | 0.950 | 0.983 | 0.950 | 1.099 | 0.917 | 1.072 | 0.604 |
| malate | 0.876 | 0.178 | 1.349 | 0.290 | 1.801 | 0.008 | 0.978 | 0.520 |
| PEP | 0.793 | 0.152 | 0.599 | 0.033 | 1.249 | 0.310 | 0.982 | 0.917 |
| succinate/methylmalonate | 0.944 | 0.885 | 1.379 | 0.351 | 1.041 | 0.520 | 1.298 | 0.206 |
| uracil | 0.606 | 0.254 | 1.139 | 0.788 | 1.396 | 0.852 | 0.898 | 0.724 |
| urate | 0.672 | 0.419 | 1.394 | 0.419 | 1.252 | 0.950 | 1.287 | 0.548 |
| xanthine | 0.639 | 0.206 | 1.123 | 0.694 | 1.159 | 0.950 | 0.976 | 0.885 |
| phosphocreatine | 0.905 | 0.633 | 1.920 | 0.272 | 1.765 | 0.085 | 0.810 | 0.290 |
| glycine | 1.465 | 0.040 | 0.961 | 0.788 | 1.167 | 0.724 | 1.165 | 0.290 |
| aspartate | 0.793 | 0.191 | 1.752 | 0.101 | 1.089 | 0.548 | 0.931 | 0.917 |
| serotonin | 1.161 | 0.820 | 1.253 | 0.724 | 0.766 | 0.419 | 0.566 | 0.272 |
| dimethylglycine | 0.765 | 0.724 | 1.249 | 0.604 | 1.126 | 0.788 | 1.029 | 0.548 |
| kynurenic acid | 0.523 | 0.443 | 2.313 | 0.054 | 0.941 | 0.633 | 1.379 | 0.820 |
| 5-adenosylhomocysteine | 0.921 | 0.520 | 1.367 | 0.494 | 1.879 | 0.027 | 0.822 | 0.633 |
| creatine | 0.849 | 0.351 | 1.575 | 0.221 | 1.499 | 0.152 | 0.690 | 0.130 |
| creatinine | 0.875 | 0.520 | 1.368 | 0.419 | 1.291 | 0.604 | 0.974 | 0.663 |
| thyroxine | 0.825 | 0.443 | 1.115 | 0.885 | 1.051 | 0.984 | 1.187 | 0.548 |
| cAMP | 0.566 | 0.033 | 1.158 | 0.950 | 1.354 | 0.788 | 0.532 | 0.141 |
| putrescine | 0.652 | 0.120 | 1.341 | 0.290 | 1.900 | 0.059 | 0.768 | 0.395 |
| arachidonyl_carnitine | 0.733 | 0.221 | 0.822 | 0.852 | 1.569 | 0.071 | 0.727 | 0.120 |
| 4-pyridoxate | 1.092 | 0.663 | 0.606 | 0.351 | 0.981 | 0.633 | 1.043 | 0.917 |
| uridine | 0.653 | 0.254 | 1.045 | 0.917 | 0.892 | 0.494 | 0.905 | 0.984 |
| taurocholate | 0.788 | 0.788 | 0.972 | 0.984 | 0.934 | 0.950 | 0.959 | 0.443 |
| glycodeoxycholate/glycochenodeoxycholate | 0.868 | 0.395 | 0.884 | 0.788 | 1.109 | 0.724 | 1.211 | 0.310 |
| 3-methyladipate/pimelate | 1.195 | 0.085 | 1.048 | 0.885 | 0.912 | 0.604 | 0.998 | 0.678 |
| arginine | 0.712 | 0.178 | 1.125 | 0.694 | 1.071 | 0.984 | 1.412 | 0.085 |
| anthranilic acid | 0.719 | 0.290 | 1.452 | 0.663 | 0.936 | 0.820 | 1.013 | 0.788 |
| hexoses (HILIC pos) | 1.255 | 0.443 | 1.141 | 0.788 | 0.976 | 0.663 | 1.679 | 0.071 |
| propionylcarnitine | 0.908 | 0.788 | 1.220 | 0.494 | 1.846 | 0.254 | 0.729 | 0.395 |

| **Metabolite** | **TBC1D8B FC** | **TBC1D8B P VALUE** | **TBC1D9 FC** | **TBC1D9 P VALUE** | **TBC1D9B FC** | **TBC1D9B P VALUE** | **TBCK FC** | **TBCK P VALUE** |
| --- | --- | --- | --- | --- | --- | --- | --- | --- |
| 1-methylnicotinamide | 2.726 | 0.468 | 0.139 | 0.120 | 0.137 | 0.071 | 0.051 | 0.004 |
| alpha-glycerophosphate | 2.036 | 0.852 | 1.593 | 0.330 | 1.518 | 0.178 | 1.493 | 0.254 |
| malonylcarnitine | 1.017 | 0.984 | 2.440 | 0.011 | 1.819 | 0.120 | 2.613 | 0.001 |
| erythrose-4-phosphate | 1.045 | 0.724 | 1.446 | 0.065 | 1.044 | 0.443 | 1.085 | 0.221 |
| N-carbamoyl-beta-alanine | 1.341 | 0.221 | 0.499 | 0.120 | 0.462 | 0.071 | 0.484 | 0.165 |
| hexanoylcarnitine | 0.800 | 0.468 | 1.806 | 0.059 | 1.792 | 0.130 | 2.335 | 0.071 |
| beta-alanine | 1.800 | 0.040 | 0.696 | 0.085 | 0.942 | 0.984 | 0.653 | 0.191 |
| 3-phosphoglycerate | 1.338 | 0.272 | 1.087 | 0.351 | 0.818 | 0.310 | 0.632 | 0.101 |
| inositol | 1.315 | 0.165 | 0.879 | 0.576 | 0.685 | 0.101 | 0.819 | 0.221 |
| phenylalanine | 0.959 | 0.663 | 1.385 | 0.663 | 1.876 | 0.029 | 0.920 | 0.520 |
| GABA | 0.966 | 0.395 | 0.226 | 0.002 | 0.995 | 0.885 | 0.321 | 0.008 |
| acetylcarnitine | 0.777 | 0.576 | 2.470 | 0.021 | 1.327 | 0.756 | 2.825 | 0.001 |
| butyrylcarnitine/isobutyrylcarnitine | 1.081 | 0.633 | 1.857 | 0.272 | 1.828 | 0.310 | 2.194 | 0.085 |
| myristoylcarnitine | 0.753 | 0.494 | 2.600 | 0.141 | 1.999 | 0.351 | 2.850 | 0.071 |
| 2-aminoadipate | 1.043 | 0.788 | 0.799 | 0.272 | 0.855 | 0.395 | 1.223 | 0.101 |
| DHAP/glyceraldehyde 3P | 1.230 | 0.237 | 0.973 | 0.917 | 1.033 | 0.984 | 0.533 | 0.071 |
| alanine | 1.255 | 0.373 | 1.755 | 0.059 | 1.622 | 0.237 | 0.808 | 0.468 |
| serine | 1.136 | 0.419 | 1.626 | 0.191 | 1.233 | 0.221 | 1.138 | 0.724 |
| glutamate | 1.551 | 0.178 | 0.527 | 0.015 | 1.321 | 0.576 | 0.681 | 0.272 |
| valine | 1.105 | 0.788 | 1.166 | 0.917 | 1.871 | 0.012 | 0.808 | 0.373 |
| lauroylcarnitine | 0.859 | 0.576 | 2.510 | 0.110 | 1.954 | 0.373 | 3.476 | 0.036 |
| cytidine | 0.903 | 0.788 | 1.603 | 0.373 | 1.122 | 0.788 | 1.689 | 0.373 |
| GMP | 1.407 | 0.110 | 1.737 | 0.012 | 1.463 | 0.054 | 1.349 | 0.272 |
| kynurenine | 0.619 | 0.101 | 0.384 | 0.002 | 0.504 | 0.085 | 1.625 | 0.120 |
| lactate | 1.432 | 0.059 | 0.881 | 0.576 | 1.199 | 0.604 | 0.529 | 0.033 |
| alpha-hydroxybutyrate | 0.782 | 0.191 | 0.593 | 0.008 | 0.880 | 0.663 | 1.278 | 0.120 |
| asparagine | 0.994 | 0.724 | 1.965 | 0.237 | 0.573 | 0.272 | 1.268 | 0.950 |
| lysine | 1.078 | 0.633 | 1.278 | 0.852 | 2.056 | 0.011 | 0.698 | 0.254 |
| tryptophan | 0.881 | 0.663 | 1.169 | 0.950 | 2.011 | 0.004 | 0.867 | 0.351 |
| 2-deoxycytidine | 0.631 | 0.221 | 1.923 | 0.330 | 1.683 | 0.351 | 2.648 | 0.120 |
| palmitoylcarnitine | 0.524 | 0.237 | 2.493 | 0.165 | 2.207 | 0.191 | 2.446 | 0.254 |
| guanosine | 1.278 | 0.290 | 1.575 | 0.065 | 0.800 | 0.351 | 2.082 | 0.033 |
| inosine | 1.023 | 0.663 | 0.813 | 0.351 | 1.646 | 0.044 | 1.774 | 0.036 |
| ribose-5-P/ribulose5-P | 1.855 | 0.065 | 1.284 | 0.419 | 1.054 | 0.852 | 0.651 | 0.310 |
| sorbitol | 1.205 | 0.330 | 1.238 | 0.633 | 1.087 | 0.820 | 0.765 | 0.272 |
| 2-hydroxyglutarate | 1.090 | 0.917 | 0.659 | 0.310 | 0.999 | 0.788 | 0.359 | 0.004 |
| threonine | 0.980 | 0.984 | 1.544 | 0.272 | 1.872 | 0.027 | 1.050 | 0.917 |
| isoleucine | 1.003 | 0.984 | 1.217 | 0.443 | 1.334 | 0.165 | 0.915 | 0.165 |
| 5-HIAA | 0.809 | 0.330 | 0.778 | 0.290 | 1.175 | 0.351 | 1.292 | 0.191 |
| trimethylamine-N-oxide | 0.800 | 0.254 | 0.814 | 0.221 | 0.831 | 0.254 | 1.196 | 0.191 |
| butyrobetaine | 0.991 | 0.950 | 1.327 | 0.494 | 1.501 | 0.165 | 1.566 | 0.024 |
| stearoylcarnitine | 0.409 | 0.221 | 2.695 | 0.178 | 1.469 | 0.548 | 2.390 | 0.221 |
| F1P/F6P/G1P/G6P | 1.056 | 0.756 | 1.731 | 0.078 | 0.942 | 0.756 | 1.040 | 0.788 |
| hexoses (HILIC neg) | 1.110 | 0.820 | 1.170 | 0.576 | 1.209 | 0.191 | 0.810 | 0.330 |
| hypoxanthine | 1.846 | 0.040 | 1.185 | 0.520 | 2.301 | 0.071 | 5.768 | 0.001 |
| NAD | 2.065 | 0.007 | 1.338 | 0.419 | 1.090 | 0.950 | 0.788 | 0.237 |
| NADP | 2.294 | 0.001 | 1.161 | 0.852 | 1.309 | 0.290 | 0.834 | 0.917 |
| methionine | 1.061 | 0.694 | 1.420 | 0.885 | 1.520 | 0.054 | 0.929 | 0.272 |
| glutamine | 1.232 | 0.984 | 1.447 | 0.694 | 1.965 | 0.011 | 1.267 | 0.885 |
| leucine | 1.116 | 0.520 | 1.221 | 0.494 | 1.519 | 0.085 | 0.828 | 0.130 |
| tyrosine | 0.909 | 0.788 | 1.404 | 0.724 | 1.457 | 0.141 | 0.972 | 0.576 |
| thiamine | 1.339 | 0.604 | 0.853 | 0.395 | 1.894 | 0.191 | 0.663 | 0.059 |
| niacinamide | 1.238 | 0.494 | 0.755 | 0.351 | 1.965 | 0.120 | 0.546 | 0.178 |
| alpha-glycerophosphocholine | 0.498 | 0.254 | 1.291 | 0.468 | 0.502 | 0.152 | 0.969 | 0.663 |
| thymidine | 0.900 | 0.885 | 0.752 | 0.494 | 1.217 | 0.373 | 1.250 | 0.468 |
| carnitine | 0.935 | 0.852 | 1.476 | 0.221 | 1.316 | 0.351 | 1.645 | 0.012 |
| oleylcarnitine | 0.513 | 0.178 | 2.217 | 0.178 | 1.296 | 0.604 | 3.161 | 0.059 |
| aconitate | 0.996 | 0.756 | 1.664 | 0.101 | 1.136 | 0.917 | 0.870 | 0.548 |
| CMP | 0.973 | 0.820 | 0.904 | 0.756 | 0.943 | 0.756 | 1.205 | 0.290 |
| cystathionine | 0.916 | 0.852 | 0.501 | 0.130 | 2.742 | 0.044 | 0.954 | 0.885 |
| fumarate/maleate/alpha-ketoisovalerate | 0.988 | 0.885 | 0.972 | 0.520 | 1.156 | 0.885 | 0.974 | 0.756 |
| glutathione oxidized | 2.804 | 0.004 | 0.941 | 0.917 | 1.461 | 0.310 | 0.842 | 0.917 |
| hippurate | 0.991 | 0.820 | 1.499 | 0.373 | 1.303 | 0.152 | 0.977 | 0.852 |
| pantothenate | 1.090 | 0.788 | 1.081 | 0.724 | 1.292 | 0.373 | 1.053 | 0.885 |
| sucrose | 0.837 | 0.885 | 1.062 | 0.663 | 1.047 | 0.351 | 0.895 | 0.468 |
| UMP | 1.166 | 0.663 | 1.048 | 0.756 | 1.258 | 0.395 | 1.350 | 0.237 |
| UDP-galactose/UDP-glucose | 2.149 | 0.065 | 1.053 | 0.419 | 0.970 | 0.604 | 0.609 | 0.152 |
| 6-phosphogluconate | 2.065 | 0.130 | 2.673 | 0.085 | 1.537 | 0.330 | 0.889 | 0.694 |
| histidine | 0.836 | 0.468 | 1.881 | 0.101 | 1.195 | 0.520 | 1.130 | 0.724 |
| proline | 1.375 | 0.206 | 1.138 | 0.663 | 1.084 | 0.950 | 0.948 | 0.520 |
| cis/trans-hydroxyproline | 1.770 | 0.130 | 0.877 | 0.756 | 0.822 | 0.820 | 0.844 | 0.950 |
| homocysteine | 1.042 | 0.984 | 0.891 | 0.576 | 0.839 | 0.520 | 0.952 | 0.984 |
| SDMA/ADMA | 1.165 | 0.395 | 1.499 | 0.093 | 1.426 | 0.152 | 0.907 | 0.724 |
| NMMA | 1.507 | 0.101 | 1.625 | 0.049 | 1.643 | 0.078 | 0.791 | 0.468 |
| carnosine | 1.478 | 0.221 | 1.351 | 0.494 | 1.206 | 0.351 | 0.757 | 0.310 |
| adenosine | 1.779 | 0.310 | 1.455 | 0.604 | 1.401 | 0.272 | 0.629 | 0.101 |
| 2-deoxyadenosine | 1.693 | 0.191 | 0.867 | 0.694 | 1.056 | 0.885 | 1.275 | 0.373 |
| cotinine | 1.426 | 0.165 | 0.746 | 0.237 | 0.584 | 0.044 | 1.113 | 0.604 |
| pipecolic acid | 1.017 | 0.788 | 0.838 | 0.694 | 0.909 | 0.917 | 1.942 | 0.029 |
| pyroglutamic acid | 1.453 | 0.206 | 1.315 | 0.724 | 1.563 | 0.101 | 0.644 | 0.141 |
| sarcosine | 0.755 | 0.254 | 0.905 | 0.756 | 0.834 | 0.468 | 0.902 | 0.520 |
| isocitrate | 1.183 | 0.694 | 1.462 | 0.221 | 0.924 | 0.633 | 0.851 | 0.310 |
| dCMP | 0.658 | 0.078 | 0.845 | 0.310 | 0.688 | 0.419 | 1.223 | 0.852 |
| glutathione reduced | 1.783 | 0.044 | 0.868 | 0.756 | 0.913 | 0.633 | 0.893 | 0.852 |
| lactose | 0.843 | 0.885 | 1.513 | 0.576 | 1.181 | 0.206 | 0.665 | 0.191 |
| oxalate | 0.865 | 0.310 | 0.823 | 0.221 | 0.893 | 0.633 | 1.041 | 0.852 |
| thymine | 0.945 | 0.756 | 1.110 | 0.852 | 1.074 | 0.694 | 1.301 | 0.576 |
| taurodeoxycholate/taurochenodeoxycholate | 2.546 | 0.290 | 0.374 | 0.917 | 0.801 | 0.494 | 0.252 | 0.272 |
| malondialdehyde | 0.969 | 0.934 | 0.888 | 0.772 | 1.060 | 0.984 | 0.854 | 0.468 |
| ornithine | 1.026 | 0.694 | 1.665 | 0.191 | 1.089 | 0.950 | 1.048 | 0.820 |
| citrulline | 1.408 | 0.237 | 1.370 | 0.310 | 1.221 | 0.351 | 1.117 | 0.820 |
| taurine | 1.172 | 0.548 | 1.572 | 0.272 | 0.894 | 0.419 | 1.386 | 0.468 |
| acetylglycine | 1.296 | 0.330 | 1.024 | 0.724 | 1.074 | 0.756 | 1.563 | 0.078 |
| allantoin | 1.108 | 0.788 | 1.082 | 0.885 | 1.293 | 0.221 | 0.769 | 0.419 |
| betaine | 0.893 | 0.604 | 1.122 | 0.694 | 1.361 | 0.272 | 1.355 | 0.071 |
| choline | 0.885 | 0.984 | 0.981 | 0.694 | 1.173 | 0.237 | 1.029 | 0.724 |
| acetylcholine | 1.359 | 0.663 | 1.095 | 0.984 | 1.910 | 0.351 | 0.891 | 0.820 |
| xanthosine | 1.273 | 0.494 | 1.067 | 0.724 | 1.356 | 0.310 | 0.634 | 0.078 |
| methionine sulfoxide | 1.173 | 0.351 | 1.310 | 0.950 | 0.896 | 0.663 | 0.842 | 0.206 |
| valerylcarnitine/isovalerylcarnitine/2-methylbutyroylcarnitine | 0.871 | 0.633 | 1.474 | 0.468 | 1.393 | 0.548 | 0.970 | 0.950 |
| heptanoylcarnitine | 0.928 | 0.820 | 1.207 | 0.351 | 1.258 | 0.520 | 1.082 | 0.820 |
| anserine | 1.265 | 0.110 | 1.255 | 0.191 | 0.949 | 0.820 | 0.871 | 0.330 |
| adenine | 1.325 | 0.178 | 1.296 | 0.178 | 0.703 | 0.206 | 1.359 | 0.310 |
| adipate | 0.938 | 0.950 | 1.019 | 0.984 | 1.014 | 0.724 | 1.161 | 0.373 |
| alpha-ketoglutarate | 1.264 | 0.373 | 1.140 | 0.756 | 1.130 | 0.852 | 0.937 | 0.984 |
| AMP | 0.999 | 0.820 | 1.049 | 0.885 | 1.232 | 0.290 | 1.249 | 0.494 |
| citrate | 1.305 | 0.604 | 1.504 | 0.221 | 1.107 | 0.756 | 1.008 | 0.984 |
| glucuronate | 1.039 | 0.852 | 0.911 | 0.917 | 1.300 | 0.191 | 0.888 | 0.788 |
| malate | 1.077 | 0.852 | 0.973 | 0.820 | 1.176 | 0.694 | 0.991 | 0.576 |
| PEP | 1.142 | 0.604 | 1.300 | 0.110 | 0.895 | 0.756 | 0.901 | 0.950 |
| succinate/methylmalonate | 1.221 | 0.756 | 1.242 | 0.272 | 1.357 | 0.093 | 0.982 | 0.694 |
| uracil | 0.948 | 0.950 | 1.417 | 0.373 | 1.302 | 0.130 | 1.267 | 0.604 |
| urate | 1.023 | 0.917 | 1.892 | 0.206 | 1.974 | 0.071 | 1.267 | 0.663 |
| xanthine | 1.083 | 0.917 | 1.395 | 0.576 | 1.165 | 0.330 | 0.989 | 0.917 |
| phosphocreatine | 1.933 | 0.310 | 1.517 | 0.724 | 1.345 | 0.724 | 1.092 | 0.820 |
| glycine | 1.334 | 0.165 | 1.089 | 0.694 | 0.992 | 0.917 | 0.880 | 0.917 |
| aspartate | 1.340 | 0.419 | 0.929 | 0.520 | 1.101 | 0.373 | 1.354 | 0.604 |
| serotonin | 0.874 | 0.494 | 1.342 | 0.373 | 1.031 | 0.724 | 1.981 | 0.191 |
| dimethylglycine | 1.116 | 0.724 | 1.345 | 0.351 | 1.159 | 0.373 | 1.034 | 0.984 |
| kynurenic acid | 0.924 | 0.852 | 1.720 | 0.548 | 1.222 | 0.272 | 0.965 | 0.756 |
| 5-adenosylhomocysteine | 1.280 | 0.443 | 1.242 | 0.419 | 1.488 | 0.221 | 1.379 | 0.310 |
| creatine | 1.247 | 0.917 | 0.853 | 0.633 | 1.060 | 0.756 | 1.018 | 0.984 |
| creatinine | 1.256 | 0.373 | 1.111 | 0.917 | 1.231 | 0.494 | 0.906 | 0.820 |
| thyroxine | 1.082 | 0.756 | 1.197 | 0.395 | 0.962 | 0.917 | 1.036 | 0.852 |
| cAMP | 0.511 | 0.093 | 0.879 | 0.191 | 1.064 | 0.984 | 1.116 | 0.724 |
| putrescine | 0.911 | 0.663 | 0.711 | 0.272 | 0.869 | 0.576 | 0.947 | 0.917 |
| arachidonyl_carnitine | 0.901 | 0.373 | 1.248 | 0.520 | 1.108 | 0.520 | 1.540 | 0.054 |
| 4-pyridoxate | 1.463 | 0.054 | 1.057 | 0.520 | 1.245 | 0.494 | 1.136 | 0.468 |
| uridine | 1.029 | 0.950 | 1.253 | 0.520 | 0.721 | 0.152 | 0.803 | 0.520 |
| taurocholate | 0.716 | 0.576 | 0.973 | 0.468 | 0.940 | 0.373 | 0.718 | 0.152 |
| glycodeoxycholate/glycochenodeoxycholate | 1.123 | 0.694 | 1.200 | 0.604 | 0.995 | 0.820 | 0.781 | 0.221 |
| 3-methyladipate/pimelate | 0.846 | 0.290 | 0.793 | 0.272 | 1.033 | 0.917 | 1.158 | 0.310 |
| arginine | 0.872 | 0.468 | 1.373 | 0.756 | 0.933 | 0.756 | 0.956 | 0.756 |
| anthranilic acid | 0.777 | 0.419 | 0.685 | 0.206 | 0.674 | 0.206 | 0.908 | 0.820 |
| hexoses (HILIC pos) | 1.750 | 0.110 | 1.236 | 0.724 | 0.995 | 0.604 | 0.842 | 0.468 |
| propionylcarnitine | 1.047 | 0.756 | 1.758 | 0.065 | 1.638 | 0.191 | 1.142 | 0.548 |

| **Metabolite** | **USP6 FC** | **USP6 P VALUE** | **USP6NL FC** | **USP6NL P VALUE** |
| --- | --- | --- | --- | --- |
| 1-methylnicotinamide | 1.954 | 0.724 | 0.566 | 0.520 |
| alpha-glycerophosphate | 0.368 | 0.049 | 1.049 | 0.604 |
| malonylcarnitine | 0.955 | 0.852 | 1.479 | 0.330 |
| erythrose-4-phosphate | 0.601 | 0.007 | 0.940 | 0.694 |
| N-carbamoyl-beta-alanine | 1.167 | 0.373 | 0.620 | 0.351 |
| hexanoylcarnitine | 1.204 | 0.633 | 1.311 | 0.290 |
| beta-alanine | 1.258 | 0.330 | 1.000 | 0.950 |
| 3-phosphoglycerate | 0.708 | 0.054 | 0.914 | 0.820 |
| inositol | 1.016 | 0.917 | 1.163 | 0.494 |
| phenylalanine | 0.758 | 0.820 | 0.817 | 0.468 |
| GABA | 3.300 | 0.033 | 1.622 | 0.443 |
| acetylcarnitine | 0.907 | 0.788 | 1.216 | 0.885 |
| butyrylcarnitine/isobutyrylcarnitine | 0.860 | 0.548 | 1.341 | 0.788 |
| myristoylcarnitine | 0.981 | 0.885 | 2.417 | 0.130 |
| 2-aminoadipate | 1.305 | 0.120 | 0.701 | 0.065 |
| DHAP/glyceraldehyde 3P | 0.728 | 0.351 | 0.865 | 0.950 |
| alanine | 0.556 | 0.071 | 1.253 | 0.663 |
| serine | 0.847 | 0.820 | 1.422 | 0.036 |
| glutamate | 0.825 | 0.548 | 0.729 | 0.101 |
| valine | 0.741 | 0.443 | 0.792 | 0.576 |
| lauroylcarnitine | 0.938 | 0.756 | 1.729 | 0.310 |
| cytidine | 1.956 | 0.191 | 2.093 | 0.221 |
| GMP | 0.821 | 0.373 | 1.318 | 0.130 |
| kynurenine | 2.355 | 0.008 | 0.717 | 0.310 |
| lactate | 0.845 | 0.633 | 0.967 | 0.788 |
| alpha-hydroxybutyrate | 1.420 | 0.120 | 0.682 | 0.036 |
| asparagine | 0.895 | 0.468 | 2.288 | 0.033 |
| lysine | 0.658 | 0.468 | 0.989 | 0.984 |
| tryptophan | 0.830 | 0.756 | 0.759 | 0.310 |
| 2-deoxycytidine | 1.167 | 0.576 | 1.177 | 0.788 |
| palmitoylcarnitine | 1.104 | 0.788 | 2.172 | 0.178 |
| guanosine | 1.995 | 0.040 | 1.083 | 0.468 |
| inosine | 1.933 | 0.011 | 1.131 | 0.520 |
| ribose-5-P/ribulose5-P | 0.591 | 0.120 | 1.049 | 0.885 |
| sorbitol | 0.648 | 0.373 | 0.928 | 0.788 |
| 2-hydroxyglutarate | 0.689 | 0.191 | 0.786 | 0.290 |
| threonine | 0.788 | 0.576 | 0.918 | 0.885 |
| isoleucine | 0.833 | 0.820 | 0.912 | 0.788 |
| 5-HIAA | 1.466 | 0.101 | 0.808 | 0.351 |
| trimethylamine-N-oxide | 1.428 | 0.019 | 0.856 | 0.419 |
| butyrobetaine | 1.036 | 0.520 | 1.275 | 0.756 |
| stearoylcarnitine | 1.628 | 0.468 | 1.403 | 0.468 |
| F1P/F6P/G1P/G6P | 0.531 | 0.029 | 0.954 | 0.663 |
| hexoses (HILIC neg) | 0.675 | 0.604 | 0.975 | 0.633 |
| hypoxanthine | 2.214 | 0.130 | 1.144 | 0.788 |
| NAD | 0.449 | 0.021 | 0.947 | 0.694 |
| NADP | 0.441 | 0.005 | 0.766 | 0.351 |
| methionine | 0.731 | 0.885 | 1.018 | 0.788 |
| glutamine | 0.826 | 0.633 | 1.050 | 0.330 |
| leucine | 0.729 | 0.724 | 0.959 | 0.950 |
| tyrosine | 0.802 | 0.917 | 0.805 | 0.395 |
| thiamine | 0.808 | 0.984 | 1.003 | 0.852 |
| niacinamide | 0.749 | 0.917 | 0.973 | 0.820 |
| alpha-glycerophosphocholine | 0.590 | 0.290 | 0.962 | 0.852 |
| thymidine | 2.427 | 0.040 | 0.597 | 0.330 |
| carnitine | 0.991 | 0.633 | 1.258 | 0.694 |
| oleylcarnitine | 1.963 | 0.221 | 2.156 | 0.120 |
| aconitate | 0.659 | 0.141 | 1.185 | 0.548 |
| CMP | 1.234 | 0.419 | 0.941 | 0.548 |
| cystathionine | 1.992 | 0.120 | 0.409 | 0.110 |
| fumarate/maleate/alpha-ketoisovalerate | 0.758 | 0.254 | 0.924 | 0.330 |
| glutathione oxidized | 0.648 | 0.110 | 0.922 | 0.724 |
| hippurate | 0.693 | 0.351 | 0.948 | 0.724 |
| pantothenate | 0.472 | 0.024 | 0.420 | 0.002 |
| sucrose | 0.788 | 0.468 | 0.876 | 0.820 |
| UMP | 1.083 | 0.984 | 1.308 | 0.290 |
| UDP-galactose/UDP-glucose | 0.475 | 0.141 | 1.028 | 0.756 |
| 6-phosphogluconate | 0.622 | 0.548 | 0.692 | 0.419 |
| histidine | 0.736 | 0.984 | 0.939 | 0.756 |
| proline | 0.776 | 0.373 | 1.171 | 0.419 |
| cis/trans-hydroxyproline | 0.872 | 0.694 | 1.879 | 0.101 |
| homocysteine | 1.009 | 0.950 | 0.602 | 0.178 |
| SDMA/ADMA | 0.732 | 0.576 | 1.260 | 0.085 |
| NMMA | 0.706 | 0.373 | 1.505 | 0.101 |
| carnosine | 0.607 | 0.221 | 1.812 | 0.017 |
| adenosine | 0.876 | 0.950 | 0.786 | 0.520 |
| 2-deoxyadenosine | 1.230 | 0.633 | 0.731 | 0.330 |
| cotinine | 1.496 | 0.152 | 0.453 | 0.008 |
| pipecolic acid | 1.611 | 0.290 | 0.872 | 0.885 |
| pyroglutamic acid | 0.606 | 0.419 | 1.122 | 0.351 |
| sarcosine | 0.921 | 0.694 | 0.785 | 0.351 |
| isocitrate | 0.570 | 0.036 | 0.948 | 0.756 |
| dCMP | 1.447 | 0.206 | 0.603 | 0.110 |
| glutathione reduced | 0.652 | 0.152 | 0.661 | 0.152 |
| lactose | 0.499 | 0.290 | 0.554 | 0.191 |
| oxalate | 1.234 | 0.254 | 0.728 | 0.040 |
| thymine | 1.400 | 0.254 | 1.305 | 0.468 |
| taurodeoxycholate/taurochenodeoxycholate | 0.803 | 0.788 | 1.551 | 0.724 |
| malondialdehyde | 1.129 | 0.395 | 0.943 | 0.917 |
| ornithine | 0.859 | 0.548 | 1.961 | 0.078 |
| citrulline | 1.003 | 0.756 | 1.090 | 0.191 |
| taurine | 0.993 | 0.950 | 1.231 | 0.820 |
| acetylglycine | 1.083 | 0.885 | 0.992 | 0.548 |
| allantoin | 0.768 | 0.788 | 1.031 | 0.221 |
| betaine | 0.997 | 0.290 | 1.172 | 0.141 |
| choline | 1.142 | 0.373 | 1.240 | 0.078 |
| acetylcholine | 0.172 | 0.036 | 1.196 | 0.852 |
| xanthosine | 0.978 | 0.984 | 1.592 | 0.044 |
| methionine sulfoxide | 0.841 | 0.756 | 1.313 | 0.419 |
| valerylcarnitine/isovalerylcarnitine/2-methylbutyroylcarnitine | 0.654 | 0.373 | 1.454 | 0.373 |
| heptanoylcarnitine | 1.246 | 0.443 | 1.312 | 0.165 |
| anserine | 0.900 | 0.788 | 1.407 | 0.014 |
| adenine | 1.112 | 0.950 | 0.738 | 0.351 |
| adipate | 1.064 | 0.373 | 0.871 | 0.395 |
| alpha-ketoglutarate | 0.708 | 0.152 | 0.871 | 0.548 |
| AMP | 0.954 | 0.576 | 1.202 | 0.443 |
| citrate | 0.569 | 0.078 | 0.890 | 0.548 |
| glucuronate | 1.273 | 0.206 | 0.959 | 0.788 |
| malate | 0.734 | 0.237 | 0.880 | 0.468 |
| PEP | 0.719 | 0.152 | 1.251 | 0.351 |
| succinate/methylmalonate | 0.762 | 0.604 | 1.153 | 0.165 |
| uracil | 0.921 | 0.756 | 1.273 | 0.141 |
| urate | 0.490 | 0.178 | 0.733 | 0.820 |
| xanthine | 0.840 | 0.852 | 1.530 | 0.120 |
| phosphocreatine | 0.490 | 0.206 | 1.940 | 0.221 |
| glycine | 0.774 | 0.330 | 1.273 | 0.206 |
| aspartate | 0.905 | 0.663 | 1.070 | 0.633 |
| serotonin | 1.726 | 0.290 | 0.912 | 0.694 |
| dimethylglycine | 0.832 | 0.984 | 1.053 | 0.152 |
| kynurenic acid | 0.844 | 0.984 | 1.403 | 0.206 |
| 5-adenosylhomocysteine | 1.003 | 0.950 | 0.817 | 0.237 |
| creatine | 1.129 | 0.494 | 1.504 | 0.130 |
| creatinine | 0.839 | 0.852 | 1.194 | 0.494 |
| thyroxine | 0.986 | 0.984 | 1.038 | 0.468 |
| cAMP | 0.928 | 0.984 | 0.636 | 0.152 |
| putrescine | 1.391 | 0.290 | 0.899 | 0.885 |
| arachidonyl_carnitine | 1.053 | 0.494 | 0.864 | 0.576 |
| 4-pyridoxate | 0.971 | 0.576 | 0.856 | 0.724 |
| uridine | 1.865 | 0.065 | 2.050 | 0.065 |
| taurocholate | 1.175 | 0.221 | 1.210 | 0.310 |
| glycodeoxycholate/glycochenodeoxycholate | 0.693 | 0.141 | 1.245 | 0.290 |
| 3-methyladipate/pimelate | 1.259 | 0.101 | 0.931 | 0.868 |
| arginine | 0.793 | 0.395 | 1.104 | 0.520 |
| anthranilic acid | 1.359 | 0.443 | 1.087 | 0.950 |
| hexoses (HILIC pos) | 0.707 | 0.290 | 1.097 | 0.885 |
| propionylcarnitine | 0.695 | 0.604 | 0.974 | 0.950 |

**Supplementary Table 8. Lactate production in MDA-MB-468 TNBC cells silenced for various TBC1D genes**

| **Gene** | **Mean normalized** | **STD** | **P_value** |
| --- | --- | --- | --- |
| TBC1D7 | 0.208 | 0.082 | 0.010 |
| TBC1D26 | 0.277 | 0.020 | 0.010 |
| EVI5 | 0.340 | 0.117 | 0.010 |
| TBC1D22A | 0.372 | 0.123 | 0.010 |
| TBC1D25 | 0.442 | 0.064 | 0.010 |
| TBC1D28 | 0.495 | 0.184 | 0.010 |
| TBC1D3B | 0.515 | 0.063 | 0.010 |
| TBC1D22B | 0.520 | 0.106 | 0.010 |
| TBC1D31 | 0.558 | 0.176 | 0.010 |
| TBC1D23 | 0.565 | 0.052 | 0.010 |
| TBC1D8B | 0.641 | 0.099 | 0.010 |
| TBC1D3F | 0.657 | 0.126 | 0.010 |
| USP6NL | 0.685 | 0.074 | 0.010 |
| SGSM1 | 0.693 | 0.421 | 0.476 |
| USP6 | 0.710 | 0.375 | 0.257 |
| SGSM2 | 0.733 | 0.172 | 0.010 |
| TBC1D13 | 0.741 | 0.380 | 0.762 |
| TBC1D2 | 0.746 | 0.081 | 0.010 |
| TBC1D14 | 0.764 | 0.255 | 0.171 |
| SGSM3 | 0.764 | 0.156 | 0.040 |
| TBC1D32 | 0.765 | 0.090 | 0.010 |
| TBC1D1 | 0.772 | 0.094 | 0.010 |
| TBC1D10C | 0.774 | 0.356 | 0.914 |
| TBC1D8 | 0.778 | 0.047 | 0.010 |
| TBC1D5 | 0.813 | 0.084 | 0.010 |
| TBC1D19 | 0.815 | 0.199 | 0.114 |
| TBC1D24 | 0.832 | 0.357 | 0.762 |
| TBC1D30 | 0.840 | 0.301 | 0.914 |
| TBC1D20 | 0.843 | 0.108 | 0.040 |
| TBC1D10B | 0.846 | 0.084 | 0.010 |
| TBC1D17 | 0.863 | 0.214 | 0.610 |
| EVI5L | 0.871 | 0.048 | 0.020 |
| CTRL | 1.000 | 0.064 | ref. |
| TBC1D21 | 1.005 | 0.581 | 1.000 |
| RABGAP1 | 1.208 | 0.200 | 0.114 |
| TBC1D15 | 1.225 | 0.362 | 0.610 |
| GRTP1 | 1.306 | 0.459 | 0.257 |
| TBC1D10A | 1.363 | 0.362 | 0.067 |
| TBC1D12 | 1.392 | 0.335 | 0.040 |
| TBC1D4 | 1.469 | 0.303 | 0.010 |
| TBCK | 1.481 | 0.824 | 1.000 |

**Supplementary Table 9. Univariate and multivariable analyses for prognostic prediction by TBC1D7, analyzed by IHC, in various BC molecular subtypes of the IEO cohort**

| **Molecular subgroup** | **Number of patients** | **Univariate** | | | **Multivariate** | | |
| --- | --- | --- | --- | --- | --- | --- | --- |
|  |  | HR | 95% CI | P | HR | 95% CI | P |
| ALL | 1398 | 1.101 | 0.807 - 1.503 | 0.545 | 1.337 | 0.973 - 1.838 | 0.074 |
| TNBC | 136 | 4.575 | 1.587 - 13.189 | 0.005 | 5.302 | 1.788 - 15.723 | 0.0026 |
| HER2+ | 163 | 1.183 | 0.601 - 2.328 | 0.626 | 1.377 | 0.686 - 2.764 | 0.369 |
| Luminal A | 242 | 3.449 | 0.437 - 27.230 | 0.24 | 3.875 | 0.433 - 34.680 | 0.226 |
| Luminal B | 815 | 0.811 | 0.552 - 1.193 | 0.287 | 0.862 | 0.582 - 1.275 | 0.456 |

**Supplementary Table 10. List of genes encoding glucose transporters, key glycolytic enzymes and lactate dehydrogenase isoforms analyzed in TBC1D7-KD MDA-MB-468 cells**

| **Category** | **Gene** | **logFC** | **P Value** | **FDR** |
| --- | --- | --- | --- | --- |
| **Glucose Transporter** | SLC2A1 | -0.375 | 0.082 | 0.309 |
|  | SLC2A9 | -0.451 | 0.058 | 0.261 |
|  | SLC2A10 | -0.491 | 0.007 | 0.089 |
|  | SLC2A11 | -0.407 | 0.024 | 0.170 |
|  | SLC2A12 | -0.202 | 0.293 | 0.571 |
|  | SLC2A13 | -0.215 | 0.410 | 0.670 |
|  | SLC2A14 | -1.325 | 0.001 | 0.031 |
| **STEP 1** | HK1 | -0.437 | 0.008 | 0.093 |
|  | HK2 | -0.671 | 7.4E-05 | 0.009 |
| **STEP 2** | GPI | -0.362 | 0.163 | 0.431 |
| **STEP 3** | PFKL | -0.686 | 0.229 | 0.508 |
|  | PFKM | -0.461 | 0.009 | 0.102 |
|  | PFKP | -1.146 | 0.001 | 0.030 |
| **STEP 4** | ALDOA | -0.869 | 0.122 | 0.374 |
| **STEP5** | TPI1 | -0.883 | 0.016 | 0.136 |
| **STEP6** | GAPDH | -0.519 | 0.151 | 0.414 |
|  | BPGM | -0.657 | 2.6E-04 | 0.018 |
| **STEP7** | PGK1 | -0.089 | 0.446 | 0.699 |
| **STEP8** | PGAM1 | -0.092 | 0.479 | 0.724 |
| **STEP9** | ENO1 | -0.416 | 0.093 | 0.329 |
|  | ENO2 | -0.483 | 0.137 | 0.394 |
| **STEP10** | PKM | -0.276 | 0.296 | 0.573 |
| **Lactate production** | LDHB | -0.711 | 1.5E-04 | 0.013 |
|  | LDHA | -0.413 | 0.008 | 0.093 |

**Supplementary Table 11. Gene ID and siRNA oligos**

| **Gene Symbol** | **GENE ID** | **Gene Accession** | **Catalogue ID** |
| --- | --- | --- | --- |
| EVI5 | 7813 | NM_005665 | L-032510-01 |
| EVI5L | 115704 | NM_145245 | L-015901-00 |
| TBC1D1 | 23216 | NM_015173 | L-014079-01 |
| TBC1D2 | 55357 | NM_018421 | L-020463-01 |
| TBC1D3B | 414059 | NM_001001417 | L-032309-01 |
| TBC1D3F | 84218 | NM_032258 | L-014821-00 |
| TBC1D4 | 9882 | NM_014832 | L-021230-01 |
| TBC1D5 | 9779 | NM_014744 | L-020775-01 |
| GRTP1 | 79774 | NM_024719 | L-014422-02 |
| TBC1D7 | 51256 | NM_016495 | L-021140-00 |
| TBC1D8 | 11138 | NM_001102426 | L-019705-02 |
| TBC1D8B | 54885 | NM_198881 | L-020747-01 |
| TBC1D10A | 83874 | NM_031937 | L-014756-01 |
| TBC1D10B | 26000 | NM_015527 | L-016723-01 |
| TBC1D10C | 374403 | NM_198517 | L-031765-02 |
| RABGAP1 | 23637 | NM_012197 | L-012803-00 |
| TBC1D12 | 23232 | NM_015188 | L-026297-02 |
| TBC1D13 | 54662 | NM_018201 | L-016946-01 |
| TBC1D14 | 57533 | NM_020773 | L-014032-01 |
| TBC1D15 | 64786 | NM_022771 | L-016209-02 |
| TBC1D17 | 79735 | NM_024682 | L-014409-02 |
| TBC1D19 | 55296 | NM_018317 | L-020732-02 |
| TBC1D20 | 128637 | NM_144628 | L-015981-01 |
| TBC1D21 | 161514 | NM_153356 | L-016748-02 |
| TBC1D22A | 25771 | NM_014346 | L-009319-01 |
| TBC1D22B | 55633 | NM_017772 | L-021109-01 |
| TBC1D23 | 55773 | NM_018309 | L-020533-01 |
| TBC1D24 | 57465 | NM_020705 | L-022880-01 |
| TBC1D25 | 4943 | NM_001006113 | L-024659-01 |
| TBC1D26 | 353149 | NM_178571 | L-018364-01 |
| TBC1D28 | 254272 | NM_001039397 | L-023035-02 |
| TBC1D30 | 23329 | XM_037557 | L-026976-00 |
| TBC1D31 | 93594 | NM_145647 | L-015606-01 |
| TBC1D32 | 221322 | NM_152730 | L-016125-02 |
| TBCK | 93627 | NM_033115 | L-005346-00 |
| SGSM2 | 9905 | NM_014853 | L-022997-01 |
| SGSM1 | 129049 | NM_001098498 | L-024557-02 |
| SGSM3 | 27352 | NM_015705 | L-018327-02 |
| USP6 | 9098 | NM_004505 | L-006096-00 |
| TSC2 | 7249 | NM_000548 | L-003029-00 |
| SLC2A1 | 6513 | NM_006516 | L-007509-02 |
| CTRL |  |  | D-001810-10 |
