# Supplementary figures and images for: TBC1 domain-containing proteins are frequently involved in triple-negative breast cancers in connection with the induction of a glycolytic phenotype
Source: Cell Death Dis. 2024 Sep 4;15(9):647. doi: 10.1038/s41419-024-07037-2 (PMC11375060; doi:10.1038/s41419-024-07037-2)

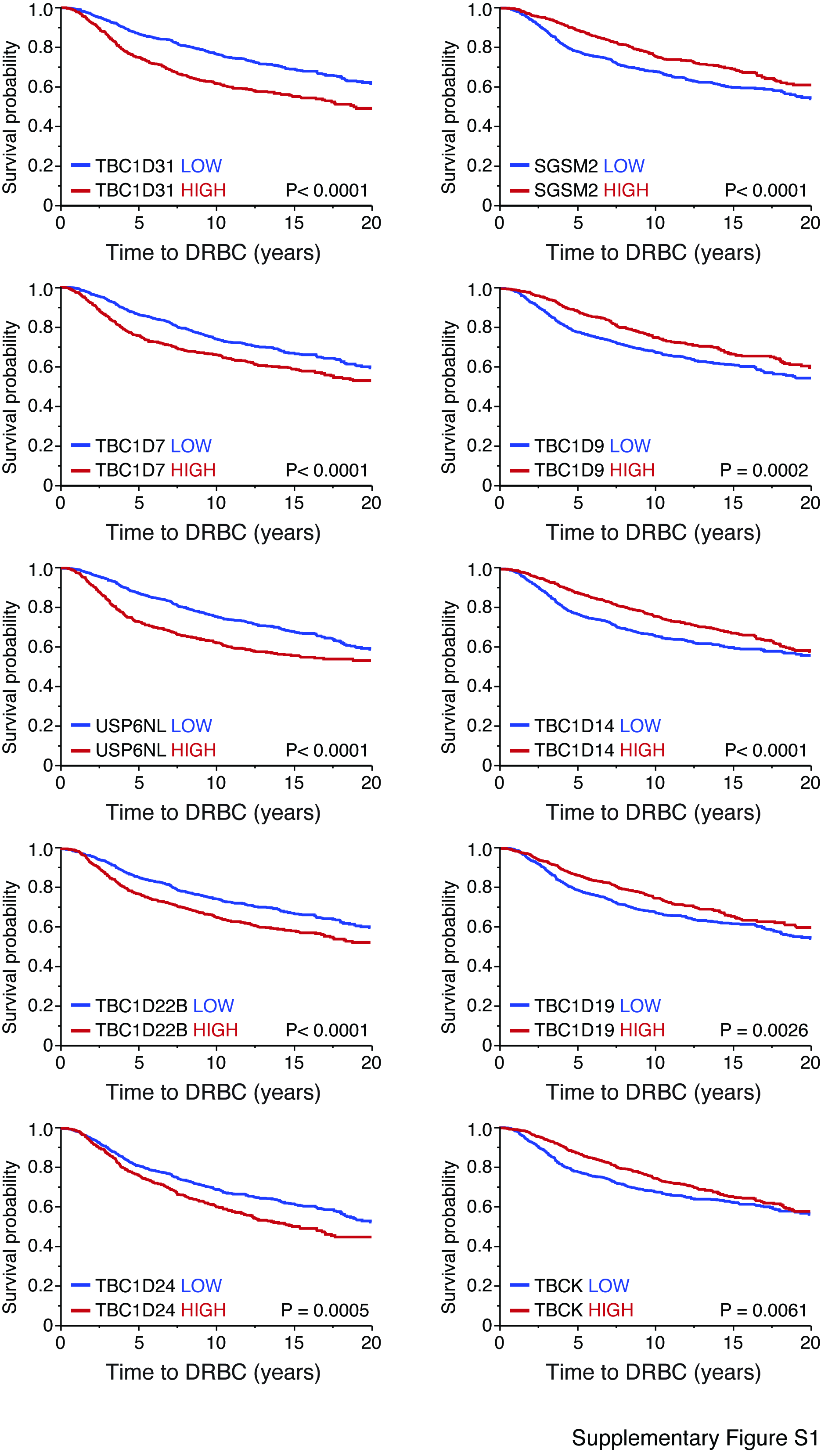

Supplement: Supplementary file 2 — Supplementary Figure S1 [file 41419_2024_7037_MOESM2_ESM.tif]

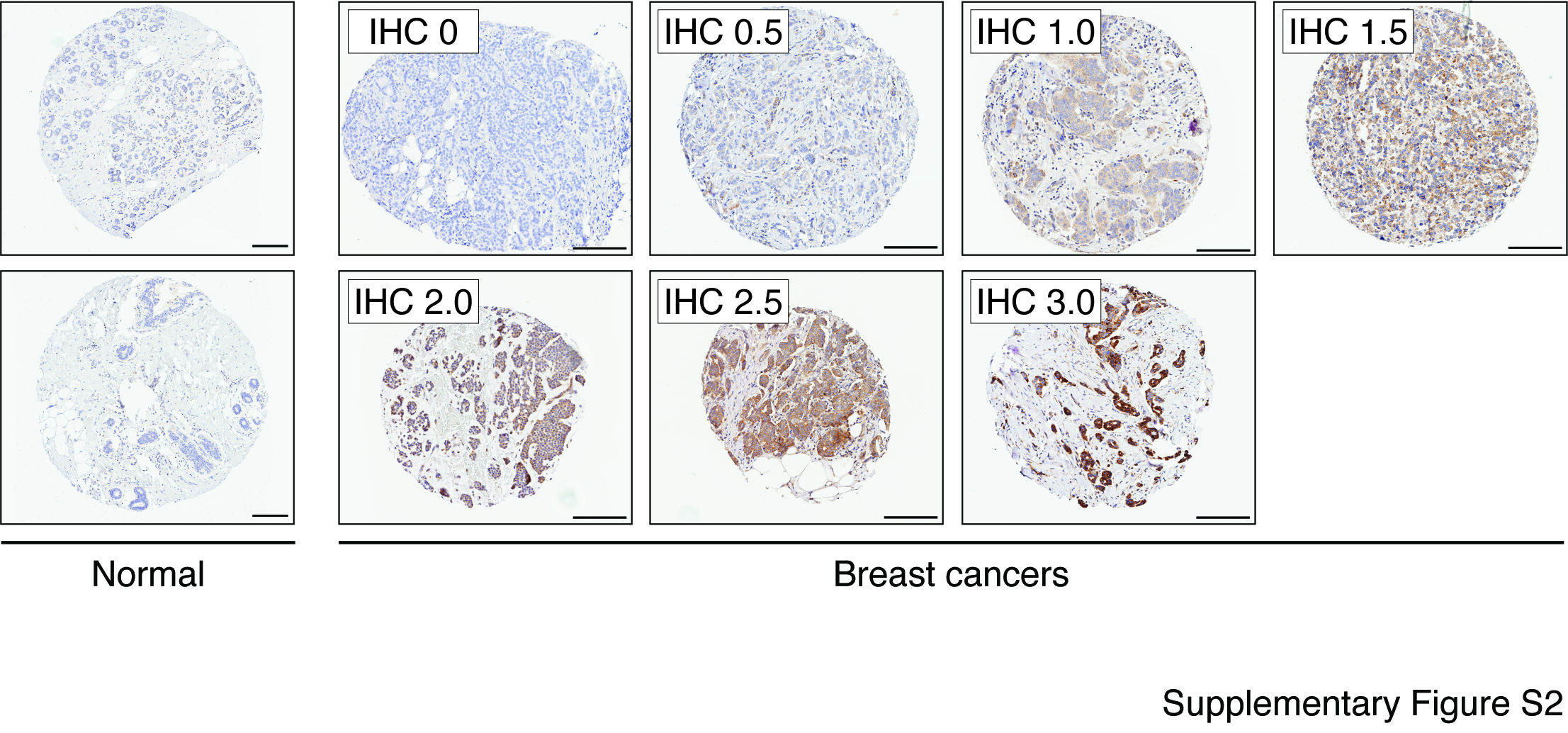

Supplement: Supplementary file 3 — Supplementary Figure S2 [file 41419_2024_7037_MOESM3_ESM.tif]

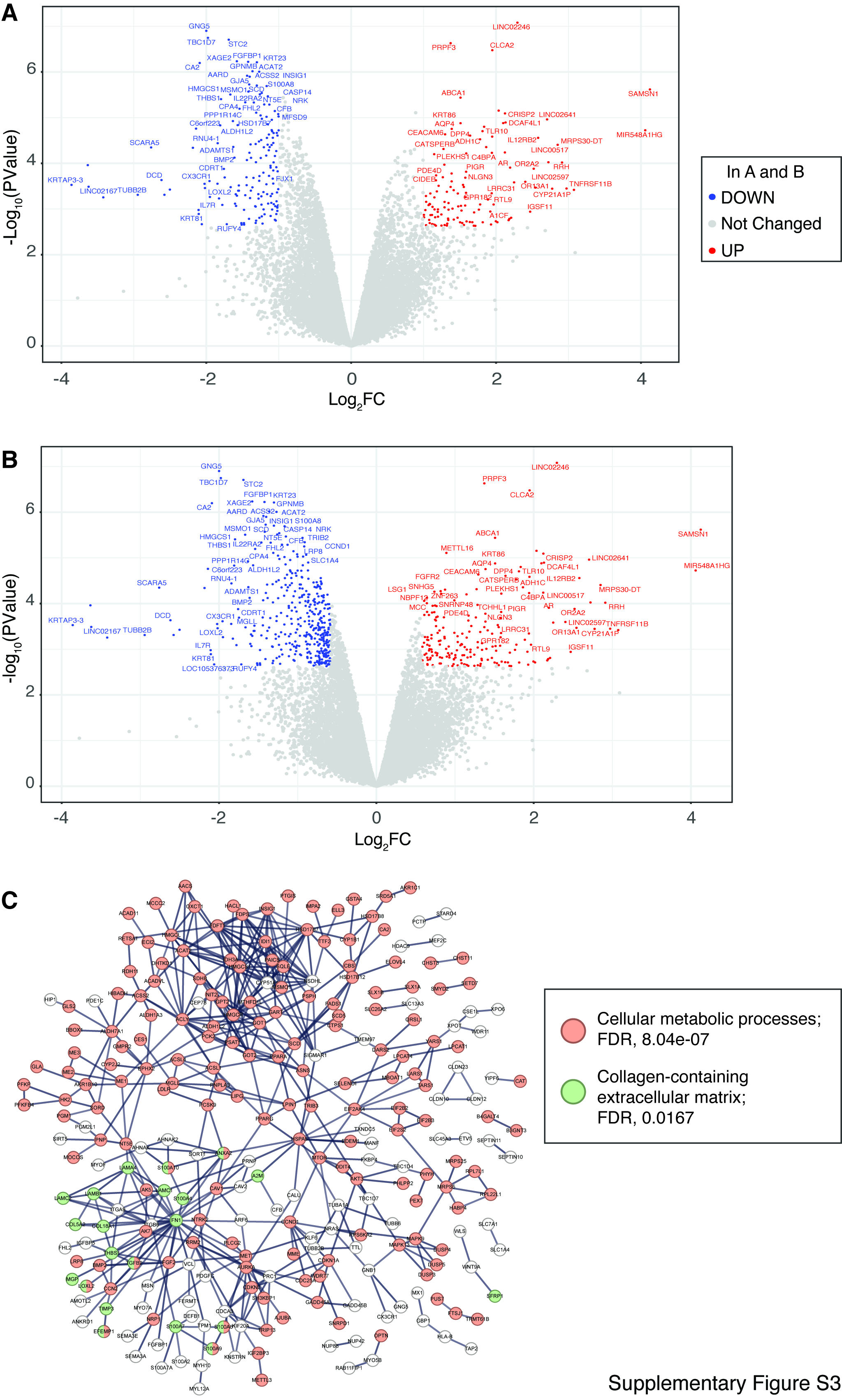

Supplement: Supplementary file 4 — Supplementary Figure S3 [file 41419_2024_7037_MOESM4_ESM.tif]

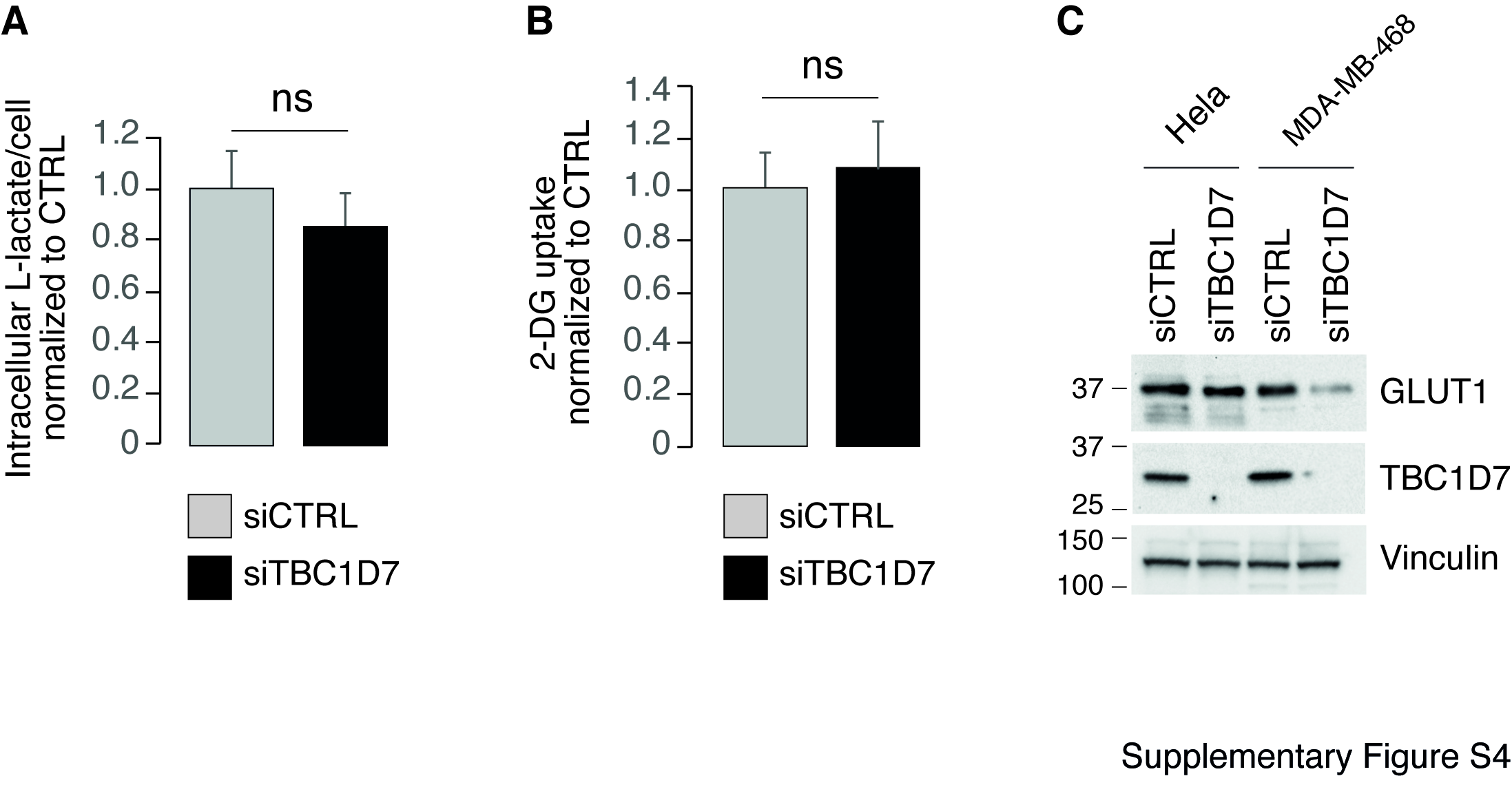

Supplement: Supplementary file 5 — Supplementary Figure S4 [file 41419_2024_7037_MOESM5_ESM.tif]

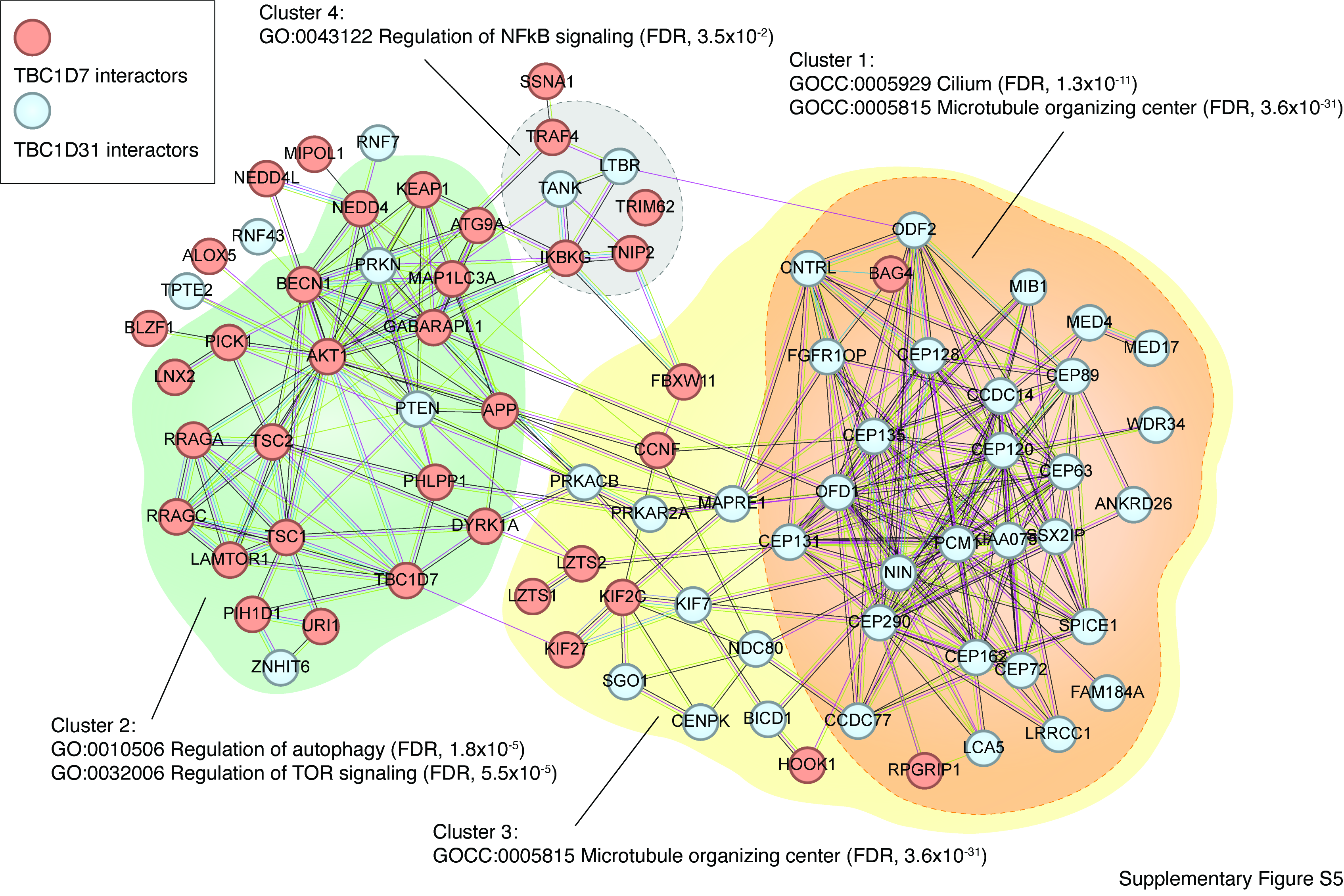

Supplement: Supplementary file 6 — Supplementary Figure S5 [file 41419_2024_7037_MOESM6_ESM.tif]
