# Supplementary material for: TBC1 domain-containing proteins are frequently involved in triple-negative breast cancers in connection with the induction of a glycolytic phenotype
Source: Cell Death Dis. 2024 Sep 4;15(9):647. doi: 10.1038/s41419-024-07037-2 (PMC11375060; doi:10.1038/s41419-024-07037-2)

Raw images of the immunoblotting experiments

In all the figures the grey panels on the right show the blot chemiluminescence.
The panels with the black frame on the left show the blot chemiluminescence overlayed on the colorimetric picture of the same membrane.

Figure 5B.
Samples were run 3 times (twice on gel 1 and once on gel 2). After transferring the proteins on nitrocellulose, the membrane corresponding to gel 1 was divided in two (B1 and B2) and the pieces were blotted with the following antibodies:

- N. B1 anti-GLUT1;
- N. B2.1 anti-GAPDH, then stripped and probed with anti-TBC1D7(B2.2).

The second membrane, corresponding to gel 2, blotted with the following antibodies:

- N. B3 anti-TBC1D22B.

Anti-GLUT1 (rabbit monoclonal)

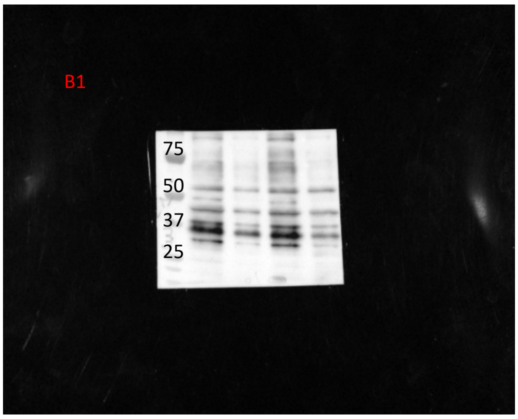

exposure of GLUT1

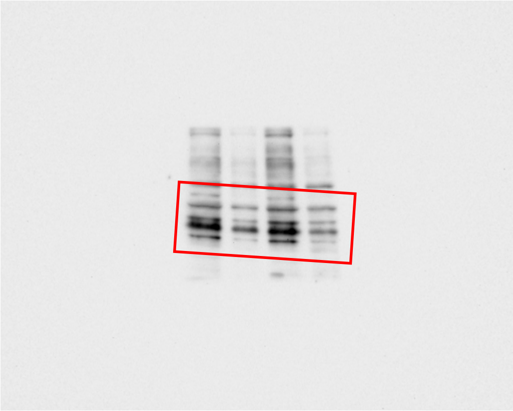

Anti-GAPDH (mouse monoclonal)

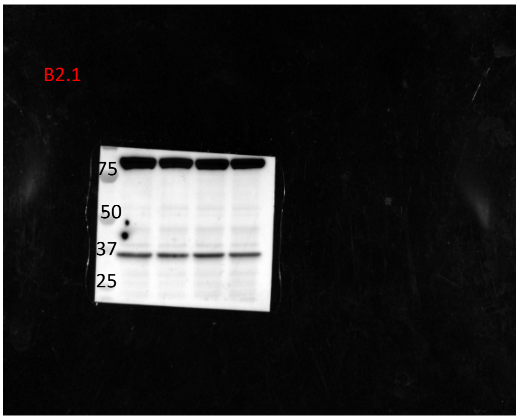

exposure of GAPDH

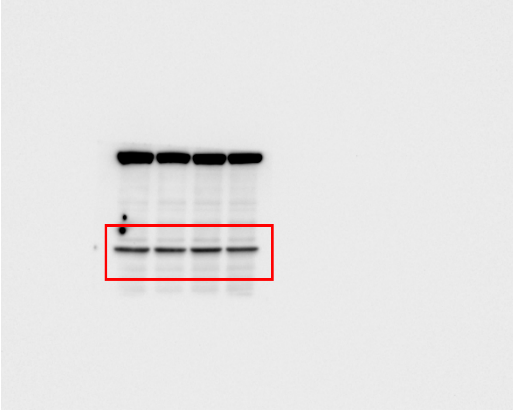

Anti-TBC1D7 (rabbit monoclonal)

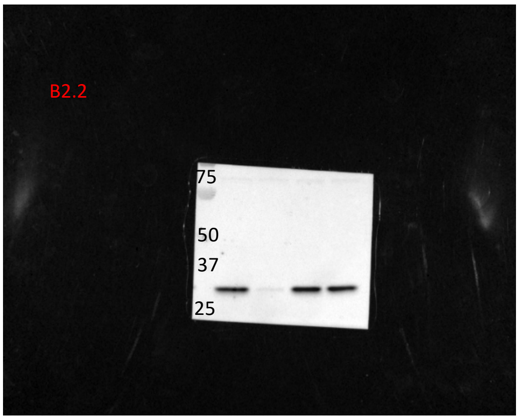

exposure of TBC1D7

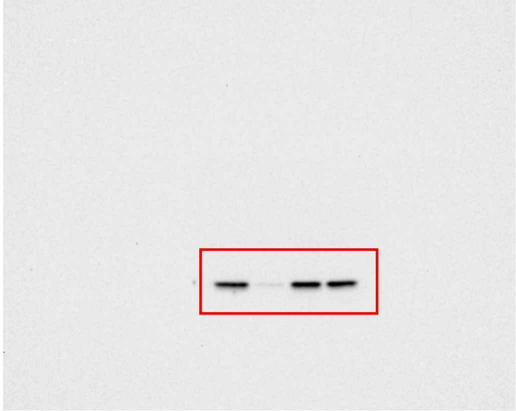

Anti-TBC1D22B (rabbit monoclonal)

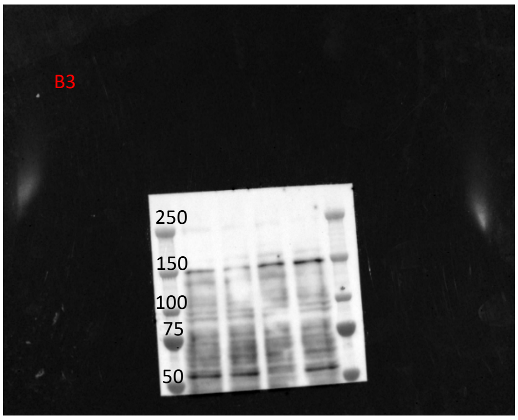

exposure of TBC1D22B

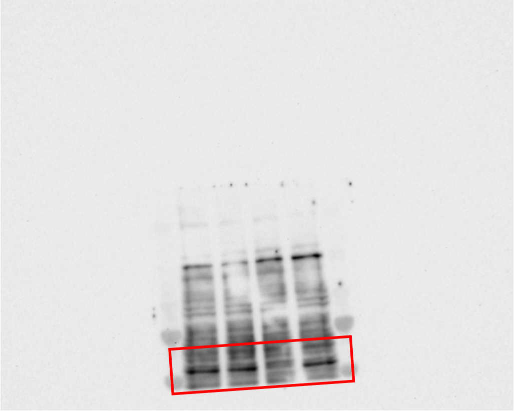

Figure 7A.
The membrane was cut in two parts, 1 and 2. Membrane 1 was first incubated with anti-TBC1D7 (1a), then stripped and probed with anti-GAPDH (1b) together with membrane 2.
The first two lanes are not relevant samples.

anti-TBC1D7 (rabbit monoclonal)

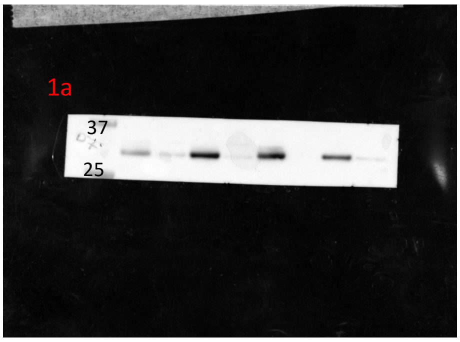

exposure of TBC1D7

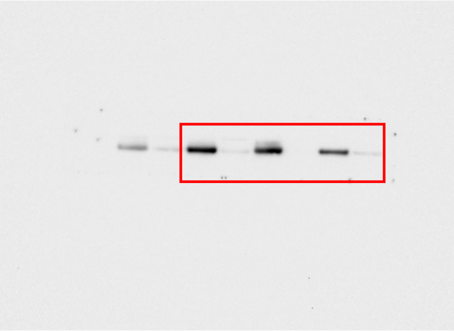

anti-GAPDH (mouse monoclonal)

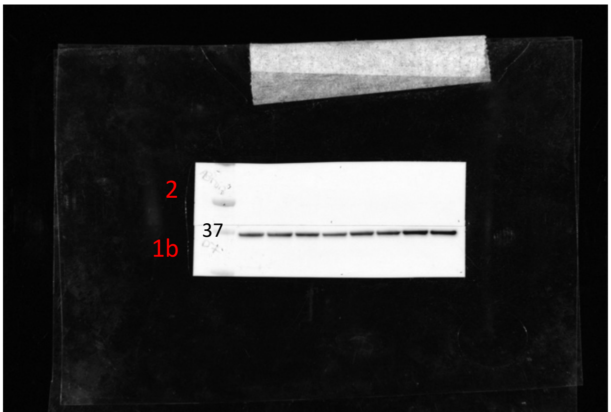

exposure of GAPDH

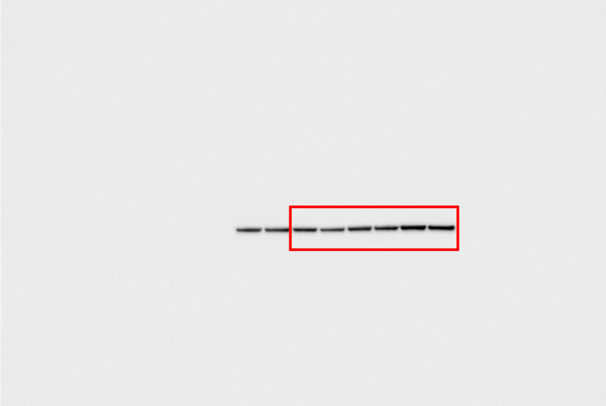

FIGURE 8A-B.

Samples in growing (GC) and serum free conditions (SF) were run on two duplicate gels. After transferring the proteins on nitrocellulose, each membrane was divided in two to obtain 4 parts (N.1, N.2 samples in GC conditions, and N.3, N.4 samples in SF condition). Each part was further cut into 3 pieces (a,b and c) and blotted with the following antibodies:

- N. 1a and 3a anti-Vinculin (1);
- N. 1b and 3b anti-pS6K;
- N. 1c and 3c anti-TBC1D7
- N. 2a and 4a anti-TSC2
- N. 2b and 4b anti-S6K
- Vinculin (2) was incubated on N. 2a and 4a, after TSC2 stripping

anti-TSC2 Ab (rabbit monoclonal) | **exposure of TSC2 in panel A** | **exposure of TSC2 in panel B**

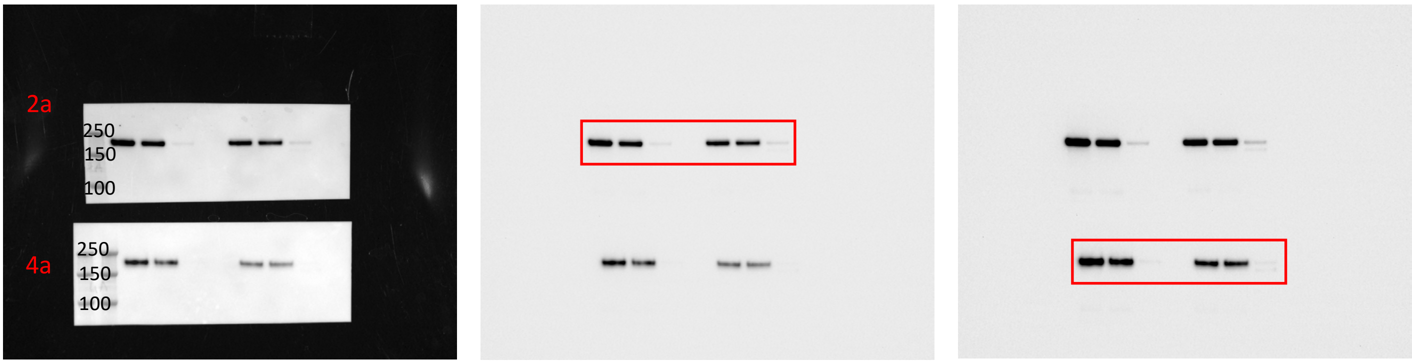

exposure of Vinculin (1)

anti-Vinculin Ab (mouse monoclonal) | **in panel A and B**

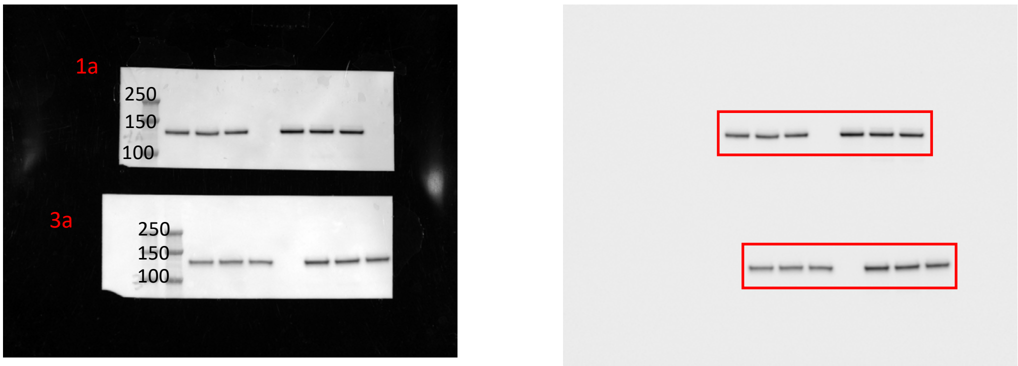

exposure of Vinculin (2) in panel A and B

anti-Vinculin Ab (mouse monoclonal)

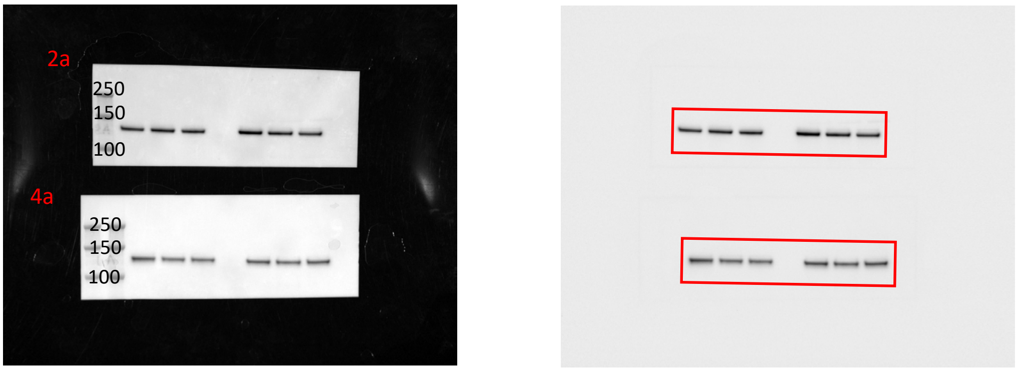

anti-TBC1D7 Ab (rabbit monoclonal)

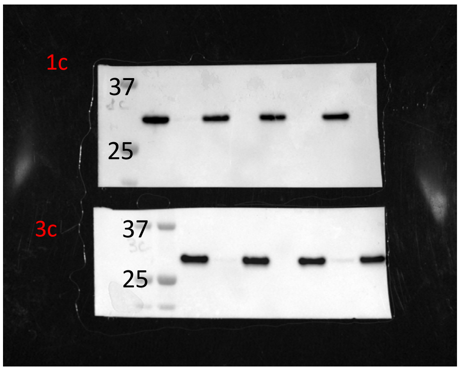

exposure of TBC1D7 in panel A and B

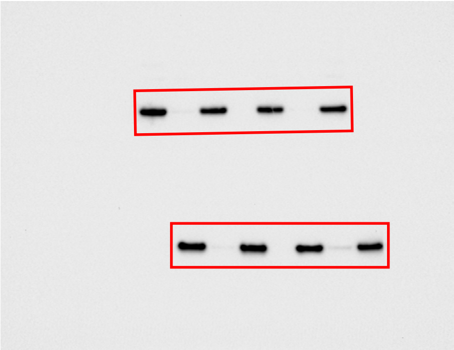

anti-phospho-p70 S6 Kinase (Thr389) (pS6K) Ab (rabbit monoclonal)

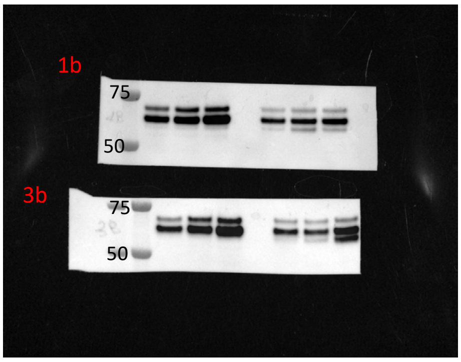

short exposure for pS6K in panel A

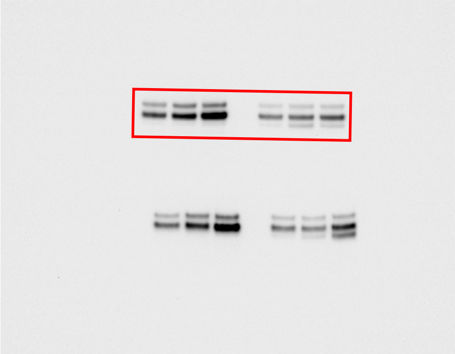

long exposure for pS6K in panel A

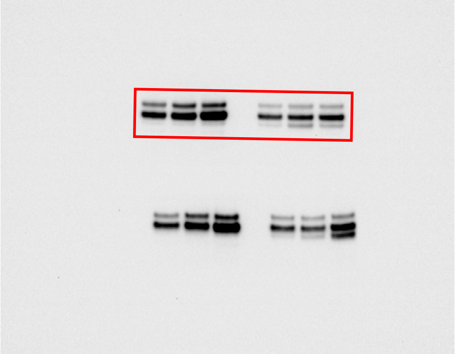

short exposure for pS6K in panel B

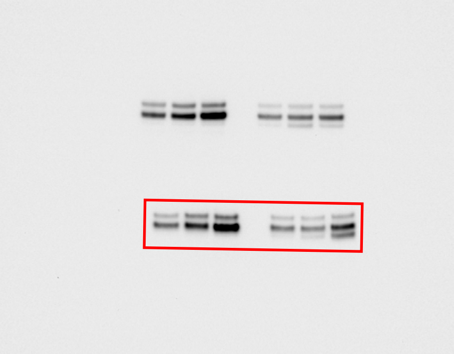

long exposure for pS6K in panel B

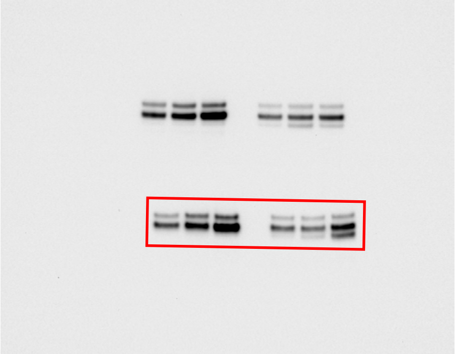

anti-p70 S6K (S6K) Ab (rabbit monoclonal)

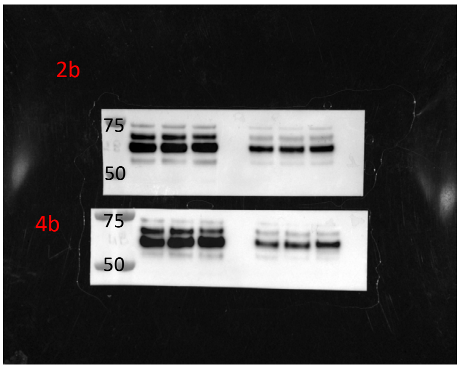

short exposure for S6K in panels A and B

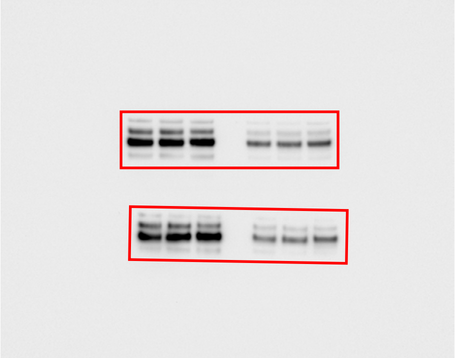

long exposure for S6K in panels A and B

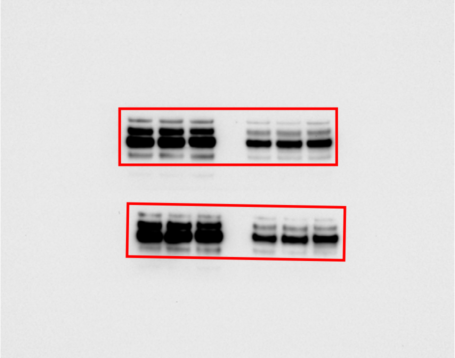

FIGURE 8C.

The membrane was divided in two parts, 1 and 2, which were incubated with different antibodies, as follow:

- N.1a anti-TSC1;
- N.1b anti-TSC2;
- N. 2 anti-HA
- Membrane 1 was first incubated with anti-TSC1 (1a), then stripped and probed with anti-TSC2 (1b)

anti-TSC1 (rabbit monoclonal)

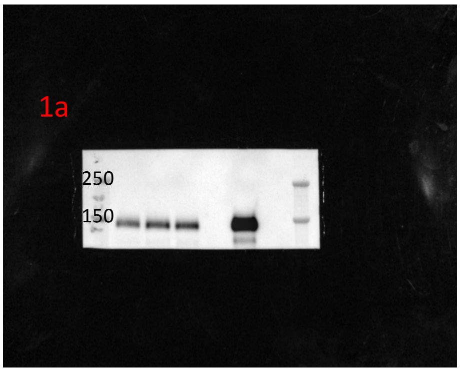

exposure of TSC1

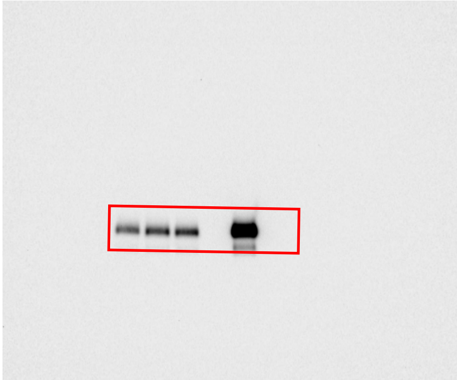

anti-TSC2 (rabbit monoclonal)

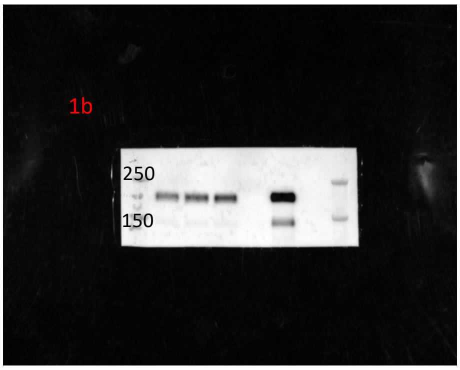

exposure of TSC2

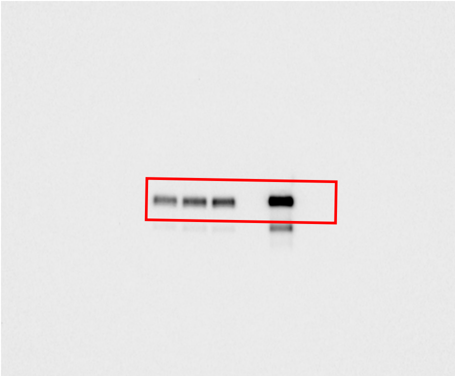

anti-HA (rabbit monoclonal)

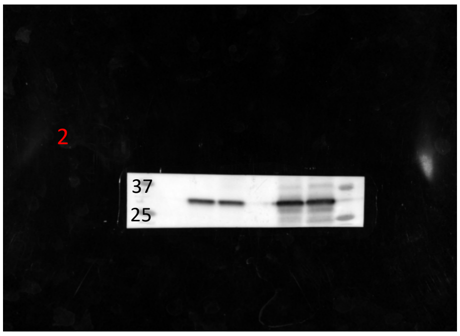

exposure of HA

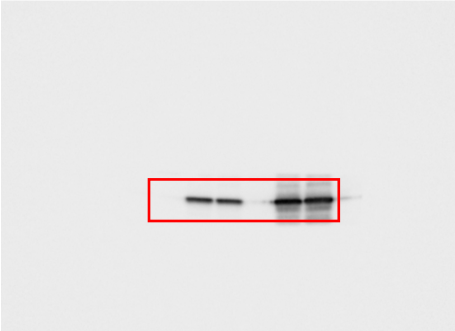

FIGURE 8D.

The membrane was divided in two parts, 1 and 2, which were incubated with different antibodies, as follow:

- N.1 anti-Vinculin;
- N. 2a anti-HA;
- N. 2b anti-TBC1D7

• Membrane 2 was probed with anti-HA (2a), then stripped and probed with anti-TBC1D7 (2b)

anti-Vinculin Ab (mouse monoclonal) **exposure of Vinculin**

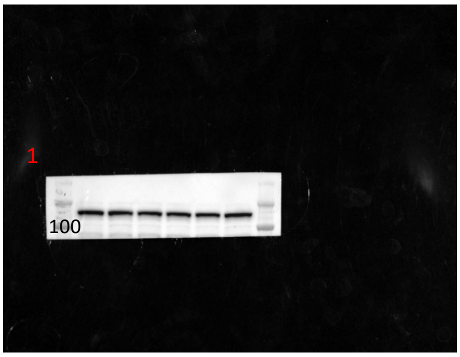

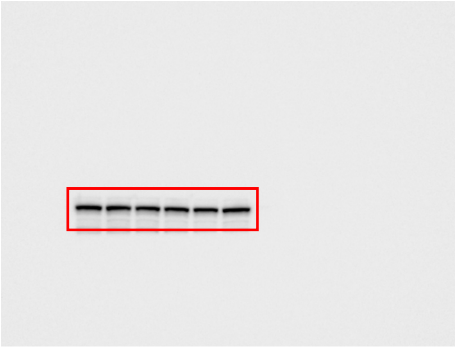

anti-HA (rabbit monoclonal) **exposure of HA**

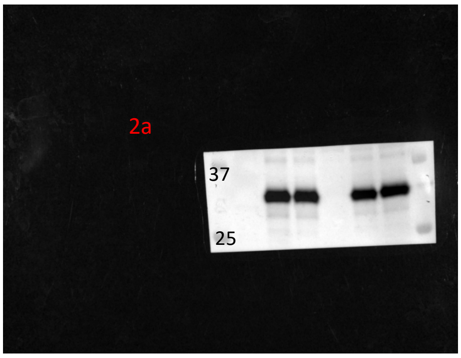

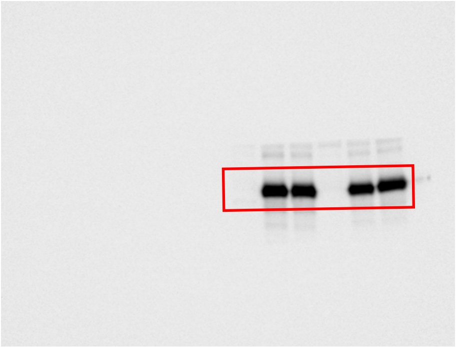

anti-TBC1D7 Ab (rabbit monoclonal) **exposure of TBC1D7**

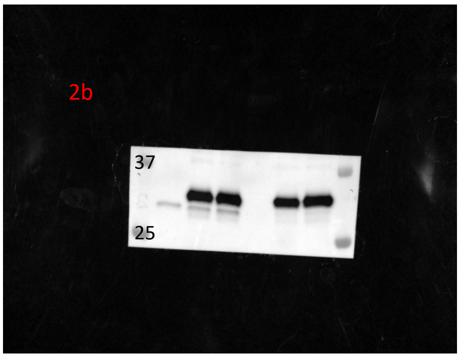

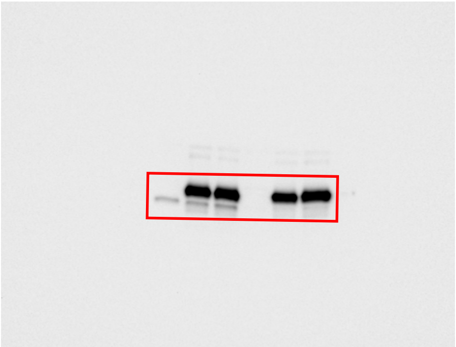

FIGURE 8F

Samples were run in duplicate on a single gel. After transferring the proteins on nitrocellulose, the membrane was divided in two to saparate the two duplicates, and each part was further cut into 3 pieces, *i.e.* a, b, c and d, e, f, and blotted with the following antibodies:

• N. a and d respectively anti-Vinculin (2) and anti-Vinculin (1);

• N. b anti-S6K;

• N. c anti-HA

- N. e anti-pS6K
- N. f anti-TBC1D7

anti-Vinculin (mouse monoclonal)

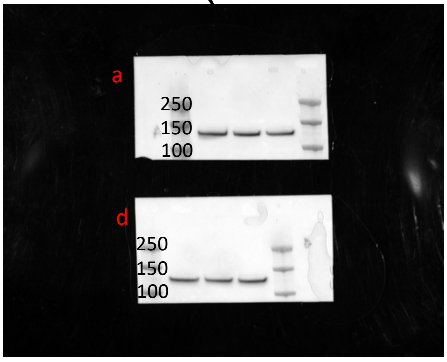

exposure of Vinculin

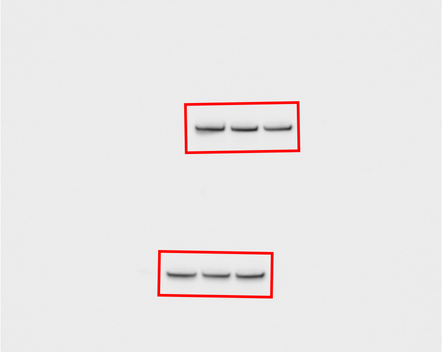

anti-TBC1D7 (rabbit monoclonal)

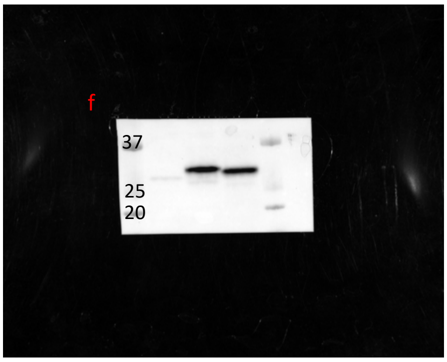

short exposure of TBC1D7

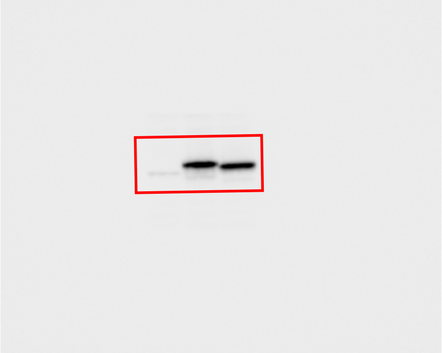

long exposure of TBC1D7

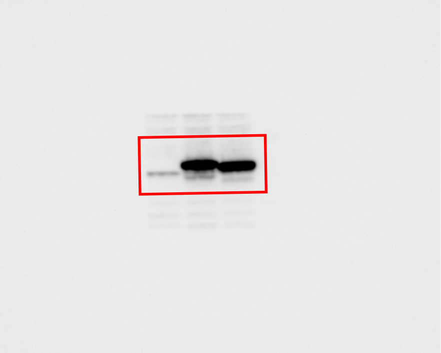

anti-HA (rabbit monoclonal)

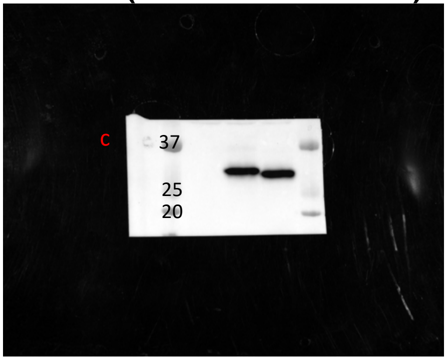

exposure of HA

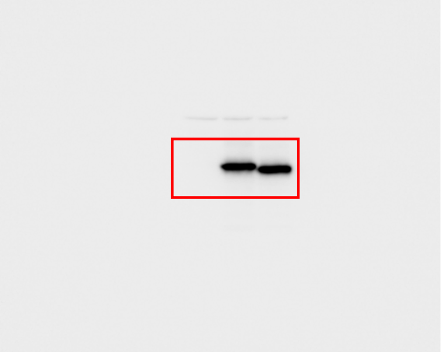

anti-phospho-p70 S6K (pS6K) (rabbit monoclonal)

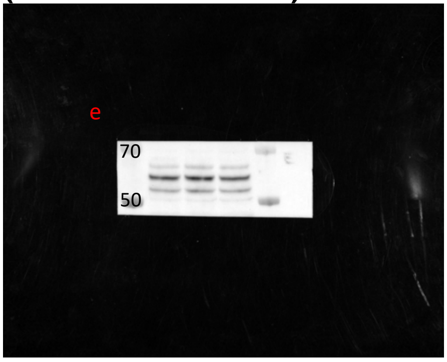

short exposure for pS6K

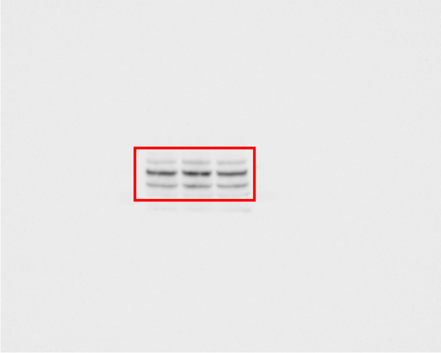

long exposure for pS6K

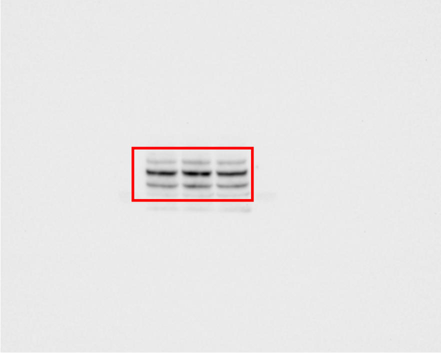

anti-p70 S6K (rabbit monoclonal) **short exposure for S6K** **long exposure for S6K**

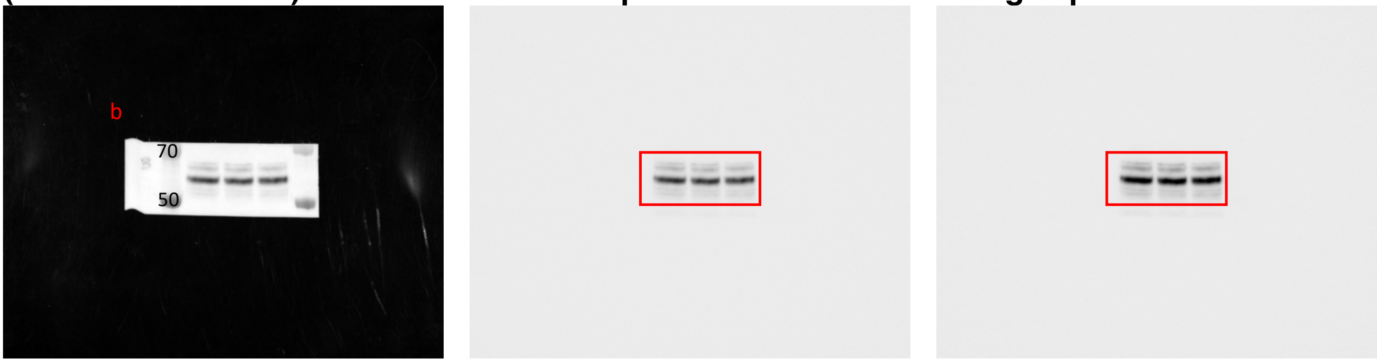

Supplementary Figure S4C

The grey panels on the right show the blot chemilumilescence. The panels with the black frame on the left show the blot chemiluminescence overlayed on the colorimetric picture of the same membrane.

Samples were run in duplicate on a single gel. After transferring the proteins on nitrocellulose, the membrane was divided in two to separate the two duplicates (a and b). The membrane b was further cut into 2 pieces (b1 and b2). All the pieces were blotted with the following antibodies:

- N. a anti-GLUT1;
- N. b1 anti-Vinculin
- N. b2 anti-TBC1D7.

Anti-GLUT1 (rabbit monoclonal) **exposure of GLUT1**

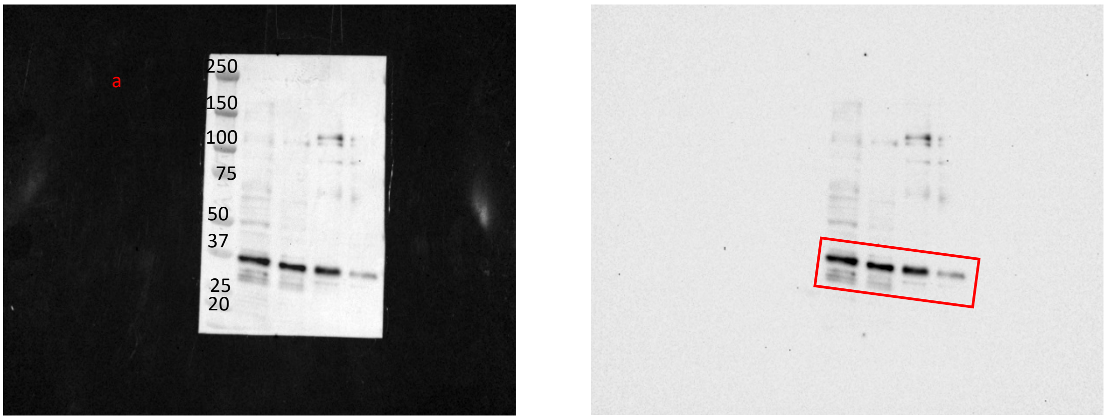

Anti-Vinculin (mouse monoclonal)

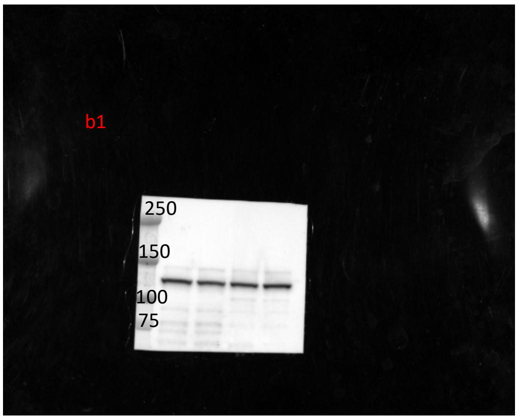

exposure of Vinculin

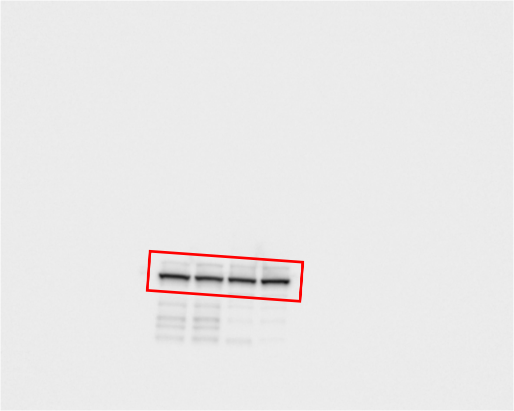

Anti-TBC1D7 (rabbit monoclonal)

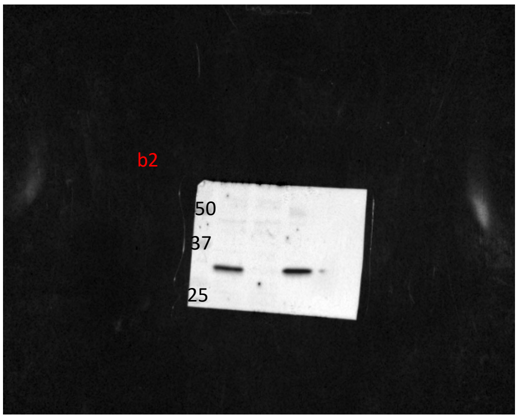

exposure of TBC1D7

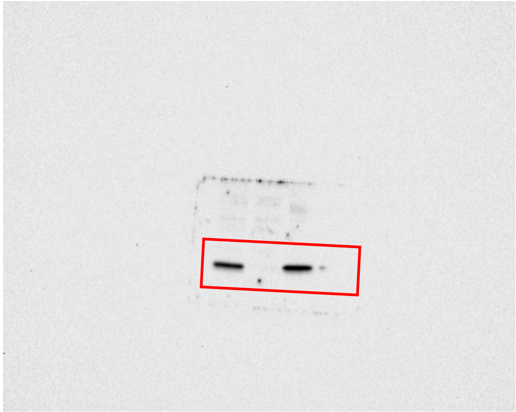

Supplement: Supplementary file 7 — Raw data western blot [file 41419_2024_7037_MOESM7_ESM.pdf]
